# Contact-dependent growth inhibition systems in *Acinetobacter*

**DOI:** 10.1038/s41598-018-36427-8

**Published:** 2019-01-17

**Authors:** Eliana De Gregorio, Raffaele Zarrilli, Pier Paolo Di Nocera

**Affiliations:** 1 Dipartimento di Medicina Molecolare e Biotecnologie Mediche, Università Federico II, Via Sergio Pansini 5, Naples, 80131 Italy; 2 Dipartimento di Sanità Pubblica, Università Federico II, Via Sergio Pansini 5, 80131 Naples, Italy

## Abstract

In bacterial contact-dependent growth inhibition (CDI) systems, CdiA proteins are exported to the outer membrane by cognate CdiB proteins. CdiA binds to receptors on susceptible bacteria and subsequently delivers its C-terminal toxin domain (CdiA-CT) into neighbouring target cells. Whereas self bacteria produce CdiI antitoxins, non-self bacteria lack antitoxins and are therefore inhibited in their growth by CdiA. In silico surveys of pathogenic *Acinetobacter* genomes have enabled us to identify >40 different CDI systems, which we sorted into two distinct groups. Type-II CdiAs are giant proteins (3711 to 5733 residues) with long arrays of 20-mer repeats. Type-I CdiAs are smaller (1900–2400 residues), lack repeats and feature central heterogeneity (HET) regions, that vary in size and sequence and can be exchanged between CdiA proteins. HET regions in most type-I proteins confer the ability to adopt a coiled-coil conformation. CdiA-CT and pretoxin modules differ significantly between type-I and type-II CdiAs. Moreover, type-II genes only have remnants of genes in their 3′ end regions that have been displaced by the insertion of novel *cdi* sequences. Type-I and type-II CDI systems are equally abundant in *A. baumannii*, whereas A. *pittii* and A. *nosocomialis* predominantly feature type-I and type-II systems, respectively.

## Introduction

Prokaryotes have developed multiple systems to secrete proteins outside the cell to promote bacterial virulence, facilitate attachment to eukaryotic cells, scavenge iron and other resources in the environment, and damage neighbouring cells. Based on their structure and function, secretion apparatuses are generally divided into six different classes. In all these systems, the formation of beta-barrel channels in the outer membrane is crucial for protein secretion^1^. The type Vb secretion system, commonly referred to as the two-partner secretion (TPS) system, is made up of two proteins: the TpsB transporter, which carries the β-barrel domain, and the secreted TpsA cargo protein^2^. TPS systems have been identified in many gram-negative bacteria and are primarily responsible for the export of large virulence proteins, such as the filamentous haemagglutinin (FHA) protein in *Bordetella pertussis* and the HMW1 and HMW2 adhesins in *Haemophilus influenzae*^2,3^.

Over the last decade, extensive research has identified and characterized a subset of TPS systems involved in the secretion of toxic proteins. One of these systems is the contact-dependent growth inhibition (CDI) system, where CdiA proteins are exported onto the outer membrane by cognate transporter CdiB proteins. Once CdiA binds to specific receptors, the C-terminal toxic domain (CdiA-CT) is clipped off and delivered to neighbouring cells, inhibiting their growth^4–6^. Characterized CDI toxins appear to either disrupt target cell membrane integrity or degrade cellular nucleic acids. Co-expressed immunity proteins, encoded by *cdi*I genes located immediately downstream of *cdi*A genes, bind CdiA-CT, neutralizing their activity in self CDI^+^ bacteria^7^.

CdiA-CT/CdiI systems are distributed in a strain-specific manner among bacterial species^6,8^. Rearrangement hotspot systems (Rhs) also express functional toxin/antitoxin (T/A) proteins and feature T/A orphan modules, although the delivery of Rhs toxins does not depend on cell-cell contact^5^. In many instances, *cdi*A-CT/*cdi*I orphan fragments, flanked by tracts identical to upstream *cdi*A gene sequences, are present at the 3′ end of *cdi* genes. Homologous recombination events with orphan sequences may lead both to the acquisition of a novel CdiA-CT/CdiI profile and to the loss of immunity against neighbouring sibling cells. Immunity would be maintained if the duplication of *cdi*A gene sequences occurred before recombination^5,9,10^. Contact-dependent inhibition is also mediated by type VI secretion systems (T6SSs), which are protein needles assembled onto bacterial outer membranes that pierce target cells to deliver toxic proteins^11^. Sibling cells survive to the attack, and T6SSs may activate a cooperative mode of growth^12^. CdiA toxin may increase bacterial fitness by increasing the number of persisting non-growing cells in high density bacterial populations exposed to antibiotic treatment^13^. CDI systems also promote social interactions between isogenic CDI^+^ cells, facilitating biofilm formation. The genes involved in the process are likely activated by contact-dependent signalling pathways^14^. Biofilm growth plays a crucial role in the persistence of pathogenic strains in infected hosts. Indeed, specific CdiA proteins are key determinants of bacterial virulence in some species^15,16^.

CDI systems are accessory genome components acquired by lateral gene transfer events and are conserved in a relatively small number of strains within a species. CDI systems that mediate growth inhibition of non-immune sister cells have recently been identified in a few strains of *Acinetobacter baumannii*^17,18^. Although *A. baumannii* is the most clinically important *Acinetobacter* species^19^ the related species 3 and 13TU now recognized as *A. pittii* and *A. nosocomialis*, respectively^20^, have also been frequently associated with nosocomial infections^21^. These three species, as well as the environmental species *A. calcoaceticus*, are closely related at the genomic level and are all referred to as components of the A. *calcoaceticus*-*A. baumannii* (ACB) complex. Furthermore, the group was recently revisited to include the pathogenic *A. seifertii*^22^ and *A. dijkshoorniae*^23^ species.

In *Acinetobacter*, several virulence factors act at the bacterial surface level^24^. Recently, we described two *A. baumannii* surface proteins that stimulate biofilm formation and adhesion to epithelial cells^25^. By wiping out non-self cells and by simultaneously stimulating the aggregation of self cells, CdiA proteins may contribute to making *Acinetobacter* a successful pathogen. Two CdiA-like proteins of 2000 (CdiA2784) and 3711 (CdiA940) aminoacids, found in the non-pathogenic *Acinetobacter baylyi* ADP1 strain, were both shown to inhibit the growth of ADP1 cells lacking the corresponding CdiI immunity proteins in a contact-dependent manner^26^.

In this study, systematic in silico analyses revealed that pathogenic *Acinetobacter* also feature CdiA proteins that significantly differ in size and structural organization. The distribution of the corresponding CDI systems differs among the species of the ACB complex.

## Results

### *Acinetobacter cdi* genes are located at different chromosomal sites

*Acinetobacter* proteins annotated as haemagglutinins using the KEGG (Kyoto Encyclopedia of Genes and Genomes) database were used as queries to search for CdiA-encoding genes in ACB complex genomes deposited in GenBank. Most of the bacterial sequenced genomes are incomplete, and many are unannotated. Moreover, giant proteins, such as CdiA, are often overlooked, with the corresponding genes annotated as pseudogenes^25^. To circumvent both problems, CdiAs were searched for using tBLASTn. All the CDI systems identified are listed in Supplementary File 1. For each *Acinetobacter* strain, the sequence type (ST), which was determined with the *Acinetobacter* Pasteur Multi Locus Sequence Typing (MLST) system^27^, is also provided. In the adopted annotation scheme, CdiA proteins are all marked by a prefix to identify the species (bau, pit, nos, cal and bay denote *A. baumannii*, *A. pittii*, *A. nosocomialis*, *A. calcoaceticus* and *A. baylyi* proteins, respectively). Thoroughly analysed CDI^+^ strains are listed in Table 1.

**Table 1:** *Acinetobacter* strains encoding type-I and type-II CdiA protein.

Strain	GenBank ID	CdiA	residues	type	Toxin domain	Reference
ATCC 19004	APQP01000005.1	pit-B5	2023	I	Tox-REase-7 (pfam15649)	this work
NIPH 146	NZ_KB849308.1	bau-B1	2014		Tox-REase-7 (pfam15649)	this work
ab031	NZ_CP009256.1	bau-B3	1920		unknown	this work
NIPH 601	APQZ01000007.1	bau-B4	2245		unknown	this work
ABBL098	LLGZ01000054.1	pit-B7	2245		unknown	this work
RUH2202	ACPK01000025.1	cal-C11	2069		Unknown	this work
PHEA-2	NC_016603	pit-C7	2071		Unknown	this work
PR320	NGDK01000053.1	pit-A4	2057		AHH endonuclease (pfam14412)	this work
TCM292	LSAK01000014.1	pit-A5	2156		nuc domain (cl00089)	this work
AP_882	NZ_CP014477.1	pit-C5	2199		unknown	this work
ab736	NZ_CP015121.1	bau-C1	2061		unknown	this work
86II/2 C	NIWI01000005.1	bau-C5	2162		unknown	this work
XH551	LYKW01000018.1	nos-C8	2174		unknown	this work
ACICU	CP000863	bau-C2	2141		MafB19-deaminase (pfam14437)	this work
ARLG1798	NGGQ01000032.1	pit-C6	2106		MafB19-deaminase (pfam14437)	this work
ZW85-1	NC_023028.1	bau-C3	2414		PT-HINT protease (cl25980)	this work
UH19608	AYFZ01000050.1	bau-C4	2181		unknown	this work
ANC3680	NZ_KB849752.1	cal-C10	2187		unknown	this work
SSA3	NZ_CP020588.1	nos-C9	2135		colicin DNase (pfam12639)	this work
ATCC19606	APRG01000014.1	bau-B2	1898		unknown	this work
IEC338SC	NZ_CP015145.1	pit-B6	1898		unknown	this work
BJAB0715	NC_021733.1	bau-A2	2146		unknown	this work
OIFC0162	AMFH01000034.1	bau-A1	2152		unknown	this work
IEC338SC	NZ_CP015145.1	pit-A3	2145		unknown	this work
A.sp. ADP1	NC_005966.1	CdiA2784	2000		Tox-REase-7 (pfam15649)	ref. ^26^.
A.sp.21871	JEWV01000004.1	nos-D2	4002	II, class 1	unknown	this work
781407	JEZS01000001.1	bau-D3	3908		colicin E5 (c113533)	this work
UH19608	AYFZ01000199.1	bau-D1	3723		unknown	this work
A.sp.FDAARGOS_131	LORV01000006.1	nos-D4	3994		Ec_869-like DNase (cd13444)	this work
A.sp.OIFC021	AMFR01000029.1	nos-D12	3840		unknown	this work
M3AC9-7	JTEC01000010.1	bau-D7	3936		toxin 47 Rnase (pfam15540)	this work
NIPH 67	APRA01000004.1	bau-D5	3983		endoU nuclease (pfam14436)	this work
TG21145	AMJH01000037.1	nos-D8	3986		unknown	this work
TG27387	ASGJ01000040.1	bau-D9	3994		unknown	this work
1035119	JEVY01000001.1	bau-D6	3983		unknown	this work
A.sp. 1542444	JEYA01000001.1	pit-D20	4269	II, class 2	unknown	this work
PHEA-2	NC_016603	pit-D6	4331		unknown	this work
Naval-72	AMFI01000006.1	bau-D13	4384		unknown	this work
GK2	LQMV01000025.1	cal-D23	4357		unknown	this work
118362	JEWB01000070.1	bau-D11	5047		unknown	this work
1267820	JEWD01000063.1	bau-D14	4989		unknown	this work
OIFC111	AMFY01000003.1	bau-D10	5181		PT-HINT protease (cl25980)	this work
232184	JEYI01000006.1	bau-D12	4910		unknown	this work
299505	JEWY01000048.1	bau-D20	4992		unknown	this work
1598530	JMOE01000001.1	bau-D17	4170	II, class 3	endoU nuclease (pfam14436)	this work
NIPH 615	APOV01000028.1	bau-D16	4940		colicin DNase (pfam12639)	this work
219_ABAU	JVPN01000006.1	bau-D21	4921		unknown	this work
99063	JEXJ01000070.1	bau-D19	4985		nuc domain (cl00089)	this work
NIPH 146	NZ_KB849308.1	bau-D15	4880		unknown	this work
A. sp. ADP1	NC_005966.1	bay-D15	3711		unknown	this work; ref. ^26^.
NIPH 2119	APOP01000003.1	nos-D18	5733		unknown	this work
PR365	NGCS01000003.1	nos-D22	5711		unknown	this work
SDF	CU468230	bau-D2	4086		unknown	this work

**Figure 1:**
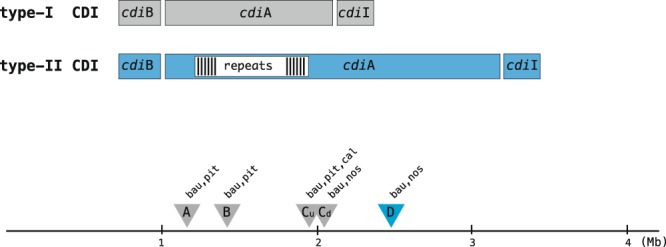
Type-I and type-II *cdi* gene clusters. Triangles denote the insertion in the *Acinetobacter* genome at sites A, B, Cu, Cd (type-I genes) and D (type-II genes). The abbreviations bau, pit, nos, and cal denote *A. baumannii*, *A. pittii*, *A. nosocomialis*, and *A. calcoaceticus* genes, respectively. Genes are not drawn to scale.

As in other gram-negative bacteria, the *Acinetobacter cdi* operons included (in the 5′−3′ order) three genes, *cdi*B*, cdi*A, and *cdi*I, which encode the transporter CdiB, CdiA, and the CdiI immunity protein that antagonizes the CdiA toxin, respectively (Fig. 1). CdiA proteins vary extensively in length and can be roughly sorted into large (~2000 amino acids) and giant (>4000 amino acids) proteins that feature long repeat arrays, herein referred to as type-I and type-II CdiA, respectively.

CDI genes identified in species of the ACB complex are located on genomic islands inserted at 5 chromosomal sites. In particular, sites A, B, Cu, and Cd host type-I genes, whereas site D hosts only type-II genes (Fig. 1). In the islands inserted at sites A and B, the *cdi* operons are flanked by genes of unknown function. Intriguingly, in the islands located at site Cu, the *cdi* operons are instead flanked by genes adjacent to the border of site D (Supplementary File 2). This observation tracks both the earlier insertion of an ancestor type-I *cdi* cluster at site D and the capture of target sequences in the excision process. Terminal repeats, corresponding to target site duplications (TSDs) mark the ends of several type-II CDI islands (Supplementary File 2). Type-I CDI islands do not feature TSDs.

### Type-I *cdi* genes

**Figure 2:**
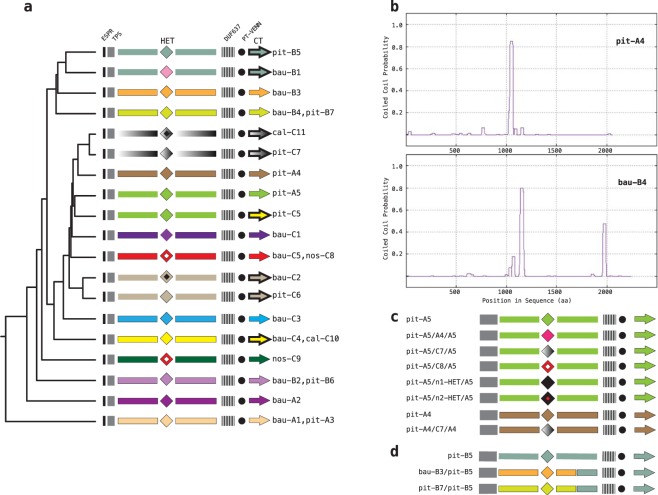
Modular organization of type-I CdiA proteins. (**a**) The cladogram was generated from a ClustalW alignment of the reported proteins. ESPR (extended signal peptide region), TPS (two-partner secretion domain), HET (Heterogeneity region), DUF637 (domain of unknown function 637), PT-VENN (pretoxin PT-VENN domain) and CT (C-terminal toxic region) are shown. Homologous protein regions are coloured similarly. Similar CT regions are outlined (**b**) Coiled-coil conformation of pit-A4 and bau-B4 CdiA proteins predicted by the MARCOIL program. (**c**) Swapping of HET modules. Chimeric pit-A5 and pit-A4 proteins are shown. (**d**) Chimeric pit-B5 CdiA proteins. In panels a, c and d the proteins are not drawn to scale.

Type-I *cdi* genes are plausibly derived from two ancestor gene clusters. This hypothesis is supported by two observations: i) the *cdi*B-*cdi*A gene distance is 24 base pairs (bp) in the A and C genes and 46 bp in the B genes; ii) the A and C transporter genes are much closer to each other than they are to B homologs (95% vs 65% similarity, respectively; Supplementary File 3). Alignments of CdiA proteins denoted a more articulated branching of type-I CDI systems (Fig. 2). Type-I CdiA proteins were marked by a prefix which identifies the species and by a letter to denote the chromosomal site of insertion of the corresponding gene cluster. All CdiA proteins display the same backbone, which is characterized by four main features: **i**) a 24 residues ESPR (extended signal peptide region, PF13018) motif at the NH2 terminus, which is recognized by the Sec-translocation machinery, and is cleaved during export through the inner membrane; **ii**) a ~140 residue region, that is recognized in the NCBI Conserved Domain Database (CDD) as a Haemagg_act (haemagglutination activity domain PF05860) domain, corresponding to the TPS domain involved in CdiA-CdiB interactions; **iii**) a domain of unknown function (DUF637, PF04830) present in a subset of CdiA proteins from other bacterial species; and **iv**) a pretoxin PT-VENN module (PFO4829) demarcating the variable CdiA-CT region (Fig. 2). In pair-wise comparisons, the homology between CdiA proteins ranges from 50 to 95% similarity. Type-I CdiA proteins are aligned in Supplementary File 4.

### Type-I CdiA proteins may adopt a coiled-coil conformation

Aside from the CT region, the primary source of variation among type-I CdiA proteins occurs in the central heterogeneity (HET) region (Fig. 2a). HET regions, the length of which ranges from 28 to 176 residues, are conserved in a few proteins but vary extensively in all others (Supplementary File 5). HET and CT regions vary independently. For instance, although similar proteins, such as cal-C11 and pit-C7 (92% identity) or bau-C2 and pit-C6 (83% identity), have the same CT region, they have different HET regions (Fig. 2). In contrast, the closely related pit-A5 and pit-C5 (87% identity) feature the same HET region but have different CT domains.

Secondary structure predictions obtained using PAIRCOIL2^28^ and MARCOIL^29^ revealed that HET regions may adopt a coiled-coil conformation (Fig. 2b). Coiled coils, which are structural protein motifs in which two or more alpha-helices are coiled together, typically contain a repeated heptameric pattern of hydrophobic and charged amino acids^30^. Most CdiA proteins may adopt a coiled-coil conformation in the HET region, as evidenced by the height of peaks in the MARCOIL profiles, which are indicative of coiled-coil formation (Supplementary File 6). Only bau-B2, pit-B6, bau-B3 and pit-C6 do not form coiled-coil conformations.

### Swapping of HET modules

We identified *A. pittii* variants of pit-A5 carrying the HET region of *A. pittii* (either pit-A4 or pit-C7) or *A. nosocomialis* (nos-C8) CdiA proteins, as well as a single pit-A4 variant carrying the HET region of pit-C7 (Fig. 2c). Variants featuring HET regions of an unknown source (n1-HET and n2-HET) were also identified (Fig. 2c). Of these variants, only pit-A5/n2-HET adopts a coiled-coil conformation (see Supplementary File 6). In most CdiA protein variants, exchanges between “donor” and “recipient” genes were limited to the HET region. Few chimeric proteins featuring the COOH region of pit-B5, and both the NH2 and the HET regions of either bau-B3 or pit-B7 were also identified in *A. pittii* and in *A. baumannii* isolates (Fig. 2c). DNA alignments revealed that switching from either bau-B3 or pit-B7 to pit-B5 sequences occurred in the same region (see Supplementary File 5).

### Multiple type-I CDI systems coexist in *A. pittii*

Most A. baumannii isolates host a single cluster of type-I cdi genes, but isolates assigned to the ST52 genotype, such as the reference ATCC 19606 strain, carry both B and C type-I genes.

In contrast, more than half of the *A. pittii* CDI^+^ strains (69/126) carry multiple *cdi* gene clusters (see Supplementary File 7). Some combinations are observed more often than others. For instance, pit-B5 genes are associated with pit-C6 genes in 39 strains, and 11 of these strains possess an additional *cdi* gene, A5/HET-n2, while other partners are present in 6 additional strains. All bau-B3/ and pit-B7/pit-B5 chimeric genes coexist with other *cdi* genes, mostly with pit-A4 and pit-C5.

Our data do not reflect an over-representation of peculiar groups of strains. Indeed, the *A. pittii* strains featuring multiple *cdi* genes belong to 20 different STs and were isolated from different geographical areas (see Supplementary File 7).

### Type-II CDI systems

Type-II c*di*B and *cdi*A genes in the ACB complex strains are separated by 63 bp, suggesting that they are derived from a single ancestor. In *A. baumannii* and *A. nosocomialis*, type-II *cdi* genes map to site D (see Fig. 1), adjacent to a tRNA-trp gene. In contrast, type-II *cdi* genes are inserted adjacent to a type-3 fimbrial gene cluster in *A. pittii*. Type-II genes present in the soil-living *A. baylyi* ADP1 strain and in the *A. baumannii* SDF strain (isolated from a louse) differ from those present in the ACB complex. In *A. baylyi* ADP1, the *cdi*B and *cdi*A genes are separated by 85 bp. In the SDF strain, the two genes are separated by *cdiC*, a gene involved in the maturation of CdiA proteins that has identified in different gamma-proteobacteria^31^. For the sake of simplicity, all type-II CdiA proteins are referred to as D proteins and numbered according to the CT profile.

**Figure 3:**
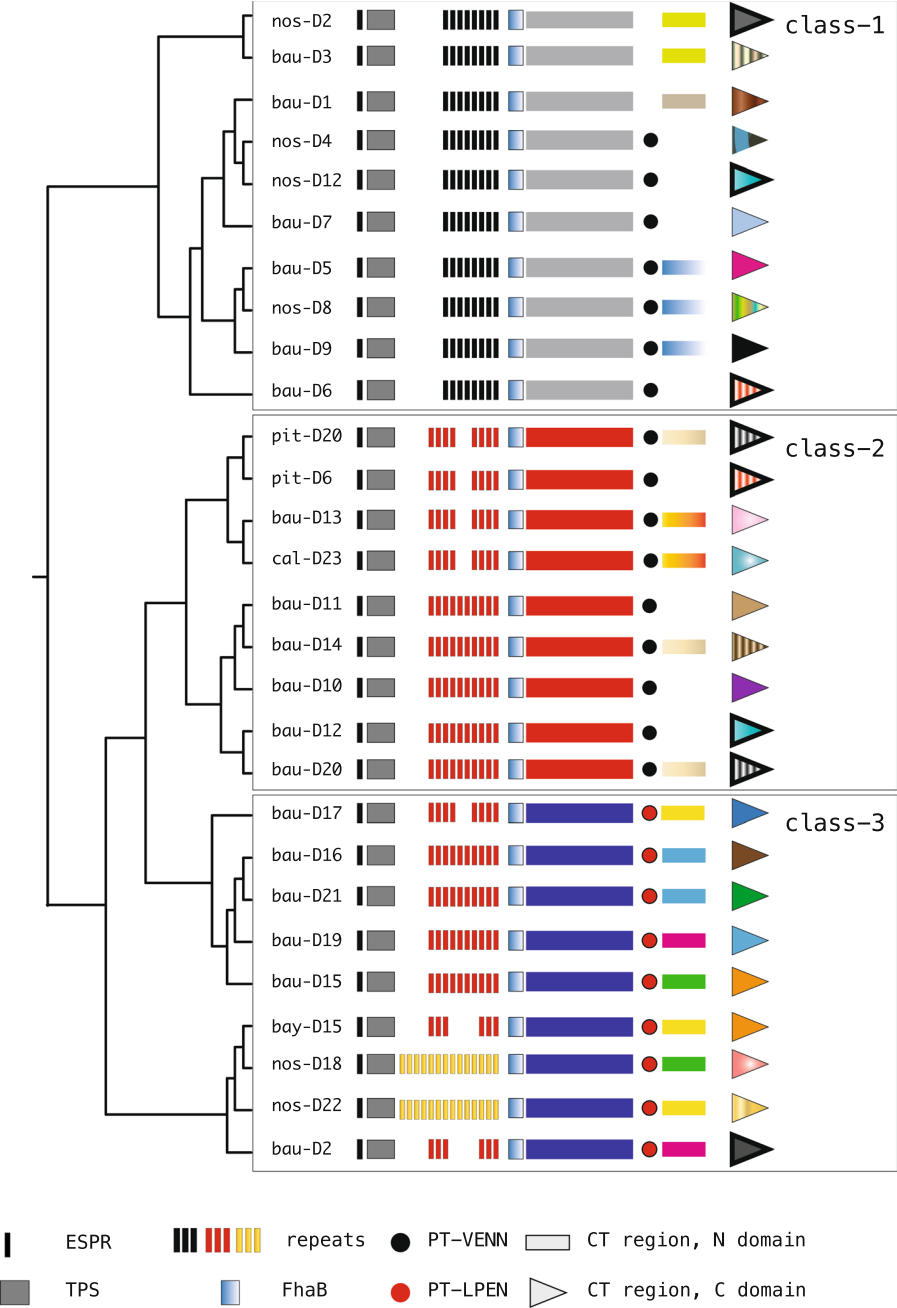
Modular organization of type-II CdiA proteins. The cladogram was generated from a ClustalW alignment of the reported proteins. Domains are indicated at the bottom. The CT regions of some CdiAs are composed of an upstream N and a downstream C domain. Proteins are not drawn to scale. Similar CT regions are coloured similarly and are outlined.

The analysed type-II CdiA proteins are shown in Fig. 3, and protein alignments are provided in Supplementary File 8. The length of these giant proteins, ranging from 3723 to 5733 residues, is correlated with the size of large repetitive (R) regions consisting of 20-mer repeats similar to those described in *B. pertussis* haemagglutinins^32^. Within repeats, branched-chain amino acids, glutamine and glycine residues are periodically reiterated in a few specific sequence combinations (Supplementary File 9). The repeat pattern is altered by indels and mutations and is discontinuous, as repeat regions occur in clusters. A detailed analysis of the R regions is out of the scope of this report. In type-II CdiAs, the R region is located between the TPS domain at the NH2 terminus and a conserved 270-residue region that is partly related to the FhaB domain (COG3210) present in many haemagglutinins (Fig. 3 and Supplementary File 9). Downstream of this domain, CdiA proteins diverge in sequence, accounting for the class 1–3 subdivision. Type-II CdiB transporters do not significantly vary and show a robust 96% similarity.

### Toxin and pre-toxin modules in type-I and type-II proteins

CT regions differ between type-I and type-II proteins. For instance, although three proteins of the two groups (*i.e*., bau-C3 and bau-D10, nos-C9 and bau-D16, and pit-A5 and bau-D19) have the same toxic activity, they are embedded within different sequence contexts. Consequently, the corresponding CT modules, as the cognate co-expressed CdiI immunity proteins, differ in all pairs. Moreover, PT-VENN modules also differ in type-I and type-II proteins, and PT-VENN modules are replaced by novel pretoxin modules called PT-LPEN in type-II proteins of class-3 (Supplementary File 9).

**Figure 4:**
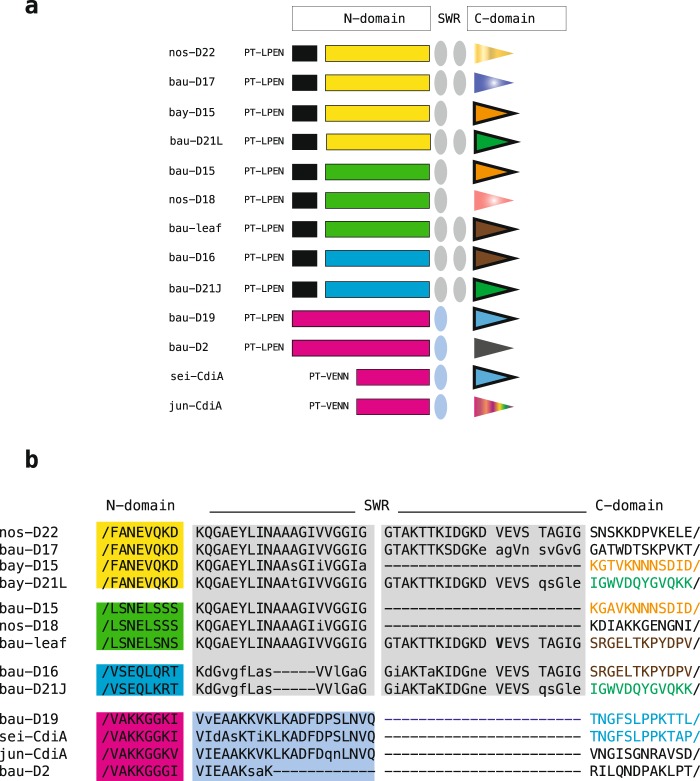
CdiA-CT regions in class 3 type-II proteins. (**a**) The different modules observed downstream of the PT-LPEN motifs are shown. Changes in the composition of the N-domain of the CT region are highlighted. SWR, switch regions at the N- and C-domain boundary are represented by ovals. (**b**) SWR sequences and immediately flanking residues of the upstream N- and downstream C-domains are shown. Predominant amino acids at each position of the SWR regions are indicated in capital letters. Sequences are coloured as in panel (**a**). Proteins marked by the prefix sei- and jun- were identified in *Acinetobacter seifertii* and *Acinetobacter junii* strains, respectively.

In most type-II CT regions, the C-toxic domains are flanked by long (150–200 residues) N-upstream modules, which are shared by multiple CT regions (Fig. 3). Upstream modules of *E. coli* CdiA-CTs regulate toxin transport in the cytoplasm of targeted cells^33^. Remarkably, upstream components are missing in the CT regions of type-I CdiAs, and only bau-B2 and pit-A4 exhibit partial homology downstream of the PT-VENN module (Supplementary File 9*)*. In class-3 type-II CdiA, 20–40 residue modules called SWR (switch regions) connect different upstream sequences to the same toxin module in proteins derived from either different species (bau-D15 and bay-D15; bau-D16 and bau-leaf, a type-II CdiA identified in the *Acinetobacter* strain Leaf130 that was isolated from *Arabidopsis thaliana*, see ref. ^34^.) or from the same species (bau-D21J and bau-D21L). SWR vary in sequence and are composed of 1 or 2 modules. Sequences homologous to N-upstream toxin and SWR modules present in bau-D19 and bau-D2 were identified in type-II CdiA proteins from *Acinetobacter junii* and *Acinetobacter seifertii* (Fig. 4). Complete CT sequences of class-3 CdiA are shown in Supplementary File 9.

### Orphan *cdi* genes

**Figure 5:**
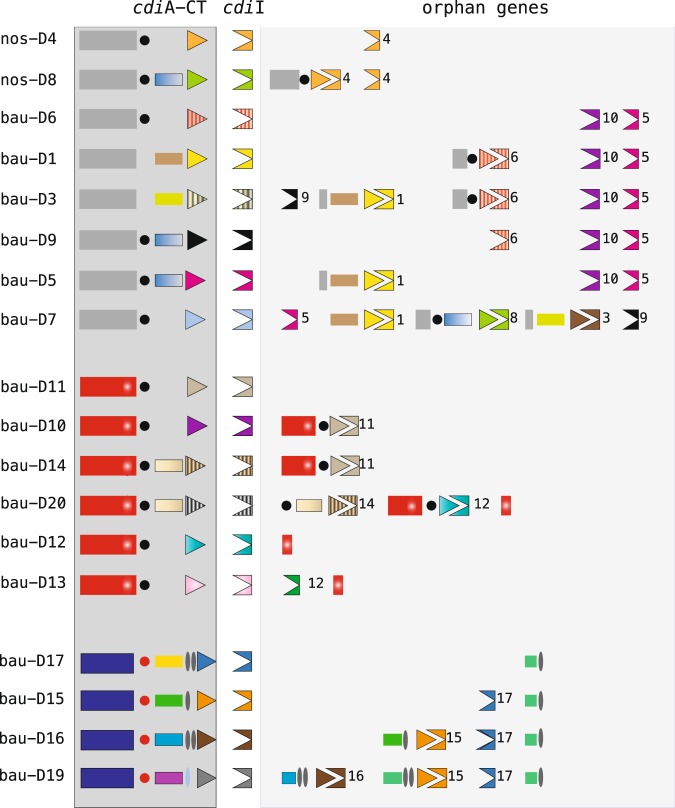
Orphan *cdi* segments. To the left, the terminal COOH regions of different type-II *cdi*A genes and the cognate coexpressed *cdi*I genes are shown. N, C and SWR domains of CT regions are denoted as in Figs 3 and 4. Vestiges of *cdi*A and *cdi*I genes, numbered and coloured as the genes from which they were derived, are shown in the orphan region to the right. Pre-toxin and toxin modules are as shown in Fig. 4.

The 3′ regions of most type-II *cdi* operons host orphan *cdi* sequences that encode CdiA-CT regions and/or immunity cdiI proteins (Fig. 5). These segments are remnants of *cdi* genes displaced by the insertion of novel *cdi* sequences. We hypothesize that the replacement of the CT region in *Acinetobacter* CDI systems takes places in a way similar to that described for the T6SS gene clusters in *Vibrio cholerae*^35^, where incoming DNA forms a heteroduplex with homologous *cdi*A sequences by promoting integration via illegitimate recombination of novel non-homologous CT sequences at the 3′ end^36^. Orphan sequences are retained, plausibly because of their potential usefulness, a hypothesis supported by the predominance of antitoxin *cdi*I genes. Accumulation of *cdi* gene remnants in the 5′−3′ direction, resulting from a step-wise remodelling of the *cdi* locus, is evident when comparing the large orphan region of bau-D19 and those from the other class 3 genes (Fig. 5). It appears that the *cdi* locus was occupied early on by bau-D17 sequences, and was subsequently remodelled by bau-D15, bau-D16 and eventually bau-D19 sequences (Fig. 5).

Some orphan genes may be derived from recombination/insertion events, as indicated by an inspection of the 3′ end region of the bau-D7 gene (Fig. 5). Instead, others may be derived from tandem duplications events, such as the *cdi* orphans in the nos-D4 and nos-D8 clusters, which are copies of the nos-D4 CT/I region. Similarly, the bau-D12 *cdi*I gene and its orphan copies, located downstream of bau-D13 and bau-D20 genes, are flanked at the 3′ end by sequences encoding a CdiA tract conserved in all class 2 proteins (Fig. 5).

### Distribution of CDI systems in *Acinetobacter*

Multilocus sequence typing (MLST) was mandatory to obtain a correct classification of CDI*-*positive strains. Several strains deposited in GenBank as *A. baumannii* were properly identified as either *A. pittii* or *A. nosocomialis* according to the MLST profile. Similarly, the completely sequenced *A. calcoaceticus* NCTC7364 strain was assigned to the *A. baumannii* ST492 profile. Isolates belonging to epidemic *A. baumannii* lineages are over-represented in GenBank. Proteins identical to the bau-C2 protein that was identified in the ACICU strain were identified in >1000 *A. baumannii* genomes. Similar to ACICU, they all belong to the epidemic ST2 genotype. The same holds true for type II proteins expressed by *A. baumannii* strains belonging to epidemic genotypes ST25 (bau-D15), ST78, and ST79 (bau-D1). In light of these results, the spread of the CDI systems in the ACB complex was assessed by evaluating the number of CDI-positive genotypes.

**Figure 6:**
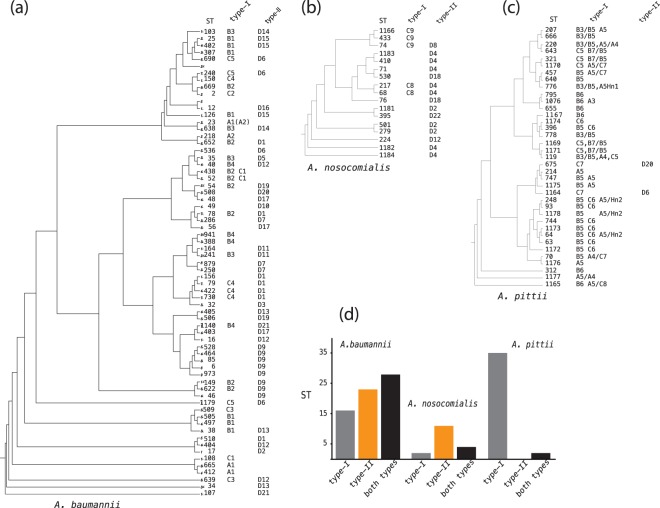
Distribution of type-I and type-II CDI systems in *A. baumannii* (panel a*), A. nosocomialis* (panel b) *and A. pittii* (panel c). The distribution ST cladograms generated from a ClustalW alignments of concatenated allele sequences of the *cpn60*, *fusA*, *gltA*, *pyrG*, *recA*, *rplB*, and *rpoB* gene segments. The frequency of STs hosting type-I, type-II or both CDI systems in the three species is shown in panel d.

Among *A. baumannii* isolates, 16 and 23 different genotypes feature only type-I or type-II CDI systems, respectively, whereas 28 genotypes feature both systems (Fig. 6). Except for a few type-II *cdi* clusters restricted to single STs, all CDI systems were observed in multiple genotypes. The distribution of CDI systems significantly differs in *A. nosocomialis and A. pittii*, which host predominantly type-II and type-I systems, respectively (Fig. 6).

Cladograms in Fig. 6 indicate that CDI systems are not restricted to subsets of *A. baumannii* strains. This result consistent with the absence of phylogenetic structuring in this species^27^. Unrooted neighbour-joining phylogenetic analyses of the entire population, unfeasible in the *A. baumannii* species which includes >1000 ST, revealed the absence of phylogenetic structuring in both *A. nosocomialis* and *A. pittii*, suggesting a random distribution of *cdi* genes in both species (Supplementary File 10).

CDI systems were also identified in other *Acinetobacter* species (Supplementary File 11). The *Acinetobacter* genus consists of >50 distinct species (http://www.bacterio.net/Acinetobacter.html). A phylogenetic tree, based on the alignment of core-genome proteins^37^, separates *Acinetobacter* species into two large groups: one group includes *A. soli*, *A. junii*, *A. baylyi* and the species of the ACB complex, while the other includes *A. lwoffii*, *A. johnsonii*, *A. gerneri*, and the closely related species, *A. guillouiae* and *A. bereziniae*^38^. The identification of type-I and type-II CdiA CDI proteins in half of the limited number of sequenced *A. guillouiae* and *A. bereziniae* strains, respectively (Supplementary File 11), suggests that CDI systems are spread throughout the entire genus.

## Discussion

Since the first description of CDI systems in *E. coli*^4^, they have been increasingly recognized as relevant accessory genome components in proteobacteria. In this study, we provide the results of a genome-wide survey of the CDI systems present in pathogenic *Acinetobacter* genomes. Although *Acinetobacter* spp. have been primarily isolated from soil, their occurrence in clinical settings has been intensively investigated as a major source of nosocomial infections. Unsurprisingly, knowledge of the organization of *Acinetobacter* genomes is mostly derived from analyses of clinical isolates. All *Acinetobacter* isolates of medical interest belong to the *A*. *calcoaceticus*-*A*. *baumannii* ACB complex. The relative abundances of sequenced *A. baumanni, A. pittii* and *A. nosocomialis* strains (2450, 157, and 72 to date, respectively) largely mirrors the frequency of infections caused by each species. Despite the predominance of *A. baumannii*, the number of *A. pittii* and *A. nosocomialis* sequenced genomes is sufficiently high to warrant meaningful comparisons. Monitoring of the distribution of CDI systems among species of the ACB complex is largely biased by both the over-representation of strains belonging to epidemic lineages and by mistakes in the species identification. Consequently, in this study, we adopted an ST-driven identification system for a coherent classification of CDI-positive clones.

Estimates based on ST profiles revealed CDI systems in approximately half of the genotypes identified as *A. nosocomialis (*17/43 genotypes) and *A. pittii* (42/97 genotypes) according to the *Acinetobacter* Pasteur MLST system. In contrast, far fewer CDI systems were identified in the *A. calcoaceticus* (2/12 genotypes) and *A. baumannii* (66/943 genotypes) strains. We speculate that the paucity of *cdi*-positive genotypes may be correlated to the limited number of sequenced *A. calcoaceticus* strains and to a reduced dissemination/maintenance of CDI systems in *A. baumannii*.

*Acinetobacter* features two distinct types of CdiA proteins that significantly differ in size and organization and exhibit limited homology (40% identity) in the ESPR and TPS domains at the NH2 terminus. Type-II CdiA proteins range in size from 3700 to 6000 residues and like most CdiA proteins characterized in other bacterial species^5–8^, these proteins feature long arrays of 20-mer repeats. The repeat region is not essential for contact-dependent growth inhibition, as supported by the analysis of type-I and type-II CdiAs in *A. bayl*yi^26^.

Body heterogeneities allowed us to sort type-II CdiA proteins into three classes. Like in many CdiA proteins from other species, in most class-1 proteins and in all class-2 proteins, CdiA-CT regions are flanked upstream by PT-VENN modules. However, in class-3 proteins, PT-VENN modules are replaced by PT-LPEN, *i.e*., novel pretoxins that are unrelated to PT-VENN and to alternative pretoxin modules identified in *Burkholderia*^39^, *Neisseria*^40^, and *Pseudomonas*^8^. Most CdiA-CT regions are bipartite and feature upstream N-modules, that are common to multiple CdiA proteins and are plausibly involved in toxin trafficking inside targeted cells^33^. CdiA-CT regions, which are located downstream of PT-LPEN pretoxins, have a more complex structure, where the profile of each region results from the combinatorial assembly of multiple modules (Fig. 4). Similar mosaic structures may not be uniquely present in *Acinetobacter*, and the construction and assay of site-directed mutants will eventually elucidate the role of the various CT modules in class 3 CdiA proteins.

As in other proteobacteria, *Acinetobacter cdi* genes are flanked by the remnants of *cdi* genes that were destroyed by the insertion of novel *cdiA/cdiI* sequences. Indeed, we observed that repeated insertions sequentially dislodged orphan modules in a 5′−3′ manner (Fig. 5). Effector (E) and immunity (I) genes were similarly displaced by the insertions of novel E/I pairs in T6SS gene clusters in *V. cholerae*^35^. Although effector genes were lost, the I module genes were retained, allowing for protection against bacteria that were still producing the old effector^35^. Orphan gene fragments retain sequences that may enable them to fuse with the upstream *cdi*A gene by homologous recombination. Orphan CT/I pairs have been reported to be able to change the toxicity profile of CdiA proteins^5,41^. CT/I reprogramming is undoubtedly harmful because of the loss of immunity against neighbouring unrearranged cells producing the old toxin. However, a few mutants equipped with the new toxin may survive and turn into predators. In contrast, recombination events driven by body or SWR orphan modules might be lethal, leading to the formation of I- strains that are rapidly eliminated.

Type-I CdiA proteins differ from type-II proteins in many respects. Repeats present in type-II proteins fold into right-handed parallel alpha-helices and may form structures protruding 40–140 nanometres from the surface of CDI^+^ bacteria^6^. Type-I proteins lack repeat sequences of any length and composition, suggesting that type-I and type-II proteins may be differently exposed on the cell surface and that their interaction with targeted cells may also differ. Type-I CdiA-CT regions lack the long upstream components present in many type II proteins. Type-I CDI systems also differ from type-II CDI systems in that they lack orphan *cdi* sequences. There is no obvious explanation for such discrepancy. It may be that type-II *cdi* genes may have recombination hotspots that make them prone to recombination events, eventually leading to the formation of orphan sequences. Alternatively, type-I CDI clusters lack orphan sequences because they have not yet experienced cycles of de novo insertions, being evolutionarily younger than type-II CDI clusters. The two CDI systems seem to have evolved independently of each other upon speciation of taxa within the ACB complex. The relative abundances of type-I and type-II systems is comparable in *A. baumannii*. In contrast, type-I systems prevail in *A. pittii*, while type-II systems prevail in *A. nosocomialis* (Fig. 6).

The different type-I CdiA proteins are largely similar, although a sequence alignment highlighted a non-homologous central region in these proteins. The HET region varies in size and sequence content and may differ in otherwise similar proteins. Secondary structure prediction showed that most HET regions can adopt a coiled-coil conformation, while analogous regions were not identified in type-II CdiA proteins. Coiled-coil domains are structural motifs used to facilitate protein oligomerization, separate functional domains, and modulate interactions with partner proteins^42^. Surprisingly, derivatives of the *A. pittii* CdiA proteins pit-A5 and pit-A4 carry HET regions that were “stolen” from other *A. pittii* or *A. nosocomialis* CdiA proteins, or even from unknown proteins. The changes occurring in all variants are due to site-specific recombination events that selectively replace HET regions. In some pit-A5 chimaeras, both the NH2 and HET regions are derived from other CdiA proteins. The need for all these changes, as well as the function of HET regions, is unknown. We hypothesize that the HET region may modulate protein-protein interactions by influencing cell surface presentation of type-I CdiA proteins and that the variety of HET regions may be associated with a similar variety of interacting protein partners.

We searched for haemagglutinin-like proteins, which are approximately 2000 amino acids in length and feature DUF367 and PT-VENN modules, in other proteobacteria. Intriguingly, proteins matching this search criterion were identified in *Neisseria meningitidis* FAM18 (GenBank AM421808, gene 444), *Serratia plymuthica* AS9 (GenBank CP002773, gene 3742), and *Moraxella catarrhalis* 25239 (GenBank CP007669, gene 760). Equally noteworthy is that all these proteins featured regions able to adopt a coiled-coil conformation. Future work is warranted to assess the occurrence of type-I CDI systems in other bacterial species, to further characterize HET modules, to identify their partners, and to ascertain the role of HET regions in the activity of CdiA proteins.

The results of this work further our knowledge of the intricate and fascinating world of CDI systems and paves the way for functional studies aimed at understanding the role of CdiA proteins in *Acinetobacter*.

## Methods

*Acinetobacter* FHA-like proteins identified in the KEGG database were used as queries for homology searches in GenBank. TBLASTn searches were carried out against both complete and draft genomes classified as *Acinetobacter* (taxid:469), *A. baumannii* (taxid:470) *A. nosocomialis* (taxid:106654), and *A. pittii* (taxid:48296). In unannotated contigs, the proteins of interest were identified with the ORFfinder (https://www.ncbi.nlm.nih.gov/orffinder/) or with the EMBOSS Sixpack (https://www.ebi.ac.uk/Tools/st/emboss_sixpack/). Protein alignments generated using MultAlin^43^, and protein domains were searched for in the NCBI Conserved Domain Database^44^.

Coiled-coils structures in type-I CdiAs were searched for with the programs Paircol2 and MARCOIL^28,29^. The sequence type of CDI^+^ strains was determined by querying either the genome or the pool of contig sequences of the strain of interest in FASTA format against the *A. baumannii* MLST database^27^.

The organization of repeat sequences in type-II CdiA was investigated with RADAR (Rapid Automatic Detection and Alignment of Repeats; https://www.ebi.ac.uk/Tools/pfa/radar/). The enrichment in particular amino acids strings was detected with COMPSEQ (http://www.hpa-bioinfotools.org.uk/pise/compseq.html). *A. baumannii* and *A. nosocomialis* ST dendrograms were generated by ClustalW alignments of concatenated allele sequences of the *cpn60*, *fusA*, *gltA*, *pyrG*, *recA*, *rplB*, and *rpoB* gene segments of the STs of interest extracted from the *Acinetobacter baumannii* MLST Pasteur database.

## Electronic supplementary material

Supplementary file

## Data Availability

All data generated or analysed during this study are included in this published article and the Supplementary Information files.
