# Supplementary material for: Contact-dependent growth inhibition systems in Acinetobacter
Source: Sci Rep. 2019 Jan 17;9:154. doi: 10.1038/s41598-018-36427-8 (PMC6336857; doi:10.1038/s41598-018-36427-8)
Supplement: Supplementary file 1 — Supplementary file [file 41598_2018_36427_MOESM1_ESM.pdf]

Contact-dependent growth inhibition systems in *Acinetobacter*

Eliana De Gregorio, Raffaele Zarrilli and Pier Paolo Di Nocera

Supplementary file 1. CDI-positive Acinetobacter strains

CDI type	species	CdiA protein	strain	GenBank	ST (sequence type)
type-I	*A. baumannii*	bau-A1	WC-348	AMZT01000004.1	**23**
type-I	*A. baumannii*	bau-A1	OIFC0162	AMFH01000034.1	**412**
type-I	*A. baumannii*	bau-A1	OCU_Ac16a	BDHK01000001.1	**412**
type-I	*A. baumannii*	bau-A1	ARLG1929	NGIG01000064.1	**412**
type-I	*A. baumannii*	bau-A1	OIFC047	AMFW01000012.1	**665**
type-I	*A. baumannii*	bau-A2	2004ZJAB5	JSCW01000063.1	**23**
type-I	*A. baumannii*	bau-A2	2004ZJAB6	JSCX01000078.1	**23**
type-I	*A. baumannii*	bau-A2	XH769	LYGN01000059.1	**23**
type-I	*A. baumannii*	bau-A2	XH683	LYJG01000011.1	**23**
type-I	*A. baumannii*	bau-A2	65	MACA01000015.1	**23**
type-I	*A. baumannii*	bau-A2	BJAB0715	NC_021733.1|	**23**
type-I	*A. baumannii*	bau-A2	XH858	NZ_CP014528.1|	**23**
type-I	*A. baumannii*	bau-A2	GB1-2	NIWO01000006.1	**23**
type-I	*A. baumannii*	bau-A2	2011BJAB9	JSDA01000044.1	**218**
type-I	*A. baumannii*	bau-A2	ABBL102	LLHC01000068.1	**ND**
type-I	*A. baumannii*	bau-B1	4190	AEPA01000201	**25**
type-I	*A. baumannii*	bau-B1	Naval-18	AFDA02000005	**25**
type-I	*A. baumannii*	bau-B1	OIFC143	AFDL01000002	**25**
type-I	*A. baumannii*	bau-B1	AB5256	AHAI01000037	**25**
type-I	*A. baumannii*	bau-B1	NIPH146	APOU01000014	**25**
type-I	*A. baumannii*	bau-B1	CI86	AVOB01000053	**25**
type-I	*A. baumannii*	bau-B1	CI79	AVOD01000028	**25**
type-I	*A. baumannii*	bau-B1	107m	CBSG010000018	**25**
type-I	*A. baumannii*	bau-B1	984213	JEVX01000144	**25**
type-I	*A. baumannii*	bau-B1	1429530	JEWM01000012	**25**
type-I	*A. baumannii*	bau-B1	RUH1486	JZBU01000028	**25**
type-I	*A. baumannii*	bau-B1	NM3	JZBV01000025	**25**
type-I	*A. baumannii*	bau-B1	LUH_6220	JZBW01000015	**25**
type-I	*A. baumannii*	bau-B1	4390	JZBY01000030	**25**
type-I	*A. baumannii*	bau-B1	741019	JZBZ01000028	**25**
type-I	*A. baumannii*	bau-B1	161/07	JZCA01000036	**25**
type-I	*A. baumannii*	bau-B1	ABBL018	LLCY01000195	**25**
type-I	*A. baumannii*	bau-B1	AB2828 LV35	LRDT01000007	**25**
type-I	*A. baumannii*	bau-B1	AB3638 LV38	LRDW01000020	**25**
type-I	*A. baumannii*	bau-B1	AB3806 LV40	LRDY01000020	**25**
type-I	*A. baumannii*	bau-B1	PR388	NGEV01000032	**25**
type-I	*A. baumannii*	bau-B1	ARLG1317	NGHO01000084	**25**
type-I	*A. baumannii*	bau-B1	HWBA8	NZ_CP020597	**25**
type-I	*A. baumannii*	bau-B1	BAuABod-3	NIWN01000020	**25**
type-I	*A. baumannii*	bau-B1	NIPH 201	APQV01000009	**38**
type-I	*A. baumannii*	bau-B1	P630	CBYG010000017	**126**
type-I	*A. baumannii*	bau-B1	233846	JMOG01000012	**126**
type-I	*A. baumannii*	bau-B1	PR337	NGEA01000060	**126**
type-I	*A. baumannii*	bau-B1	CIP70.10	NZ_LN865143.1	**126**
type-I	*A. baumannii*	bau-B1	R2091	NZ_LN997846.1	**126**
type-I	*A. baumannii*	bau-B1	DSM 30011	JJOC01000004	**738**
type-I	*A. baumannii*	bau-B1	554S	NKXP01000136.1	**307**
type-I	*A. baumannii*	bau-B1	LUH_7841	JZBX01000012	**402**
type-I	*A. baumannii*	bau-B1	ABBL007 contig-15	LLCN01000226.1	**497**
type-I	*A. baumannii*	bau-B1	ARLG1873	NGJA01000152	**505**
type-I	*A. baumannii*	bau-B2	XH730	LYHV01000053	**46**
type-I	*A. baumannii*	bau-B2	XH674	LYJP01000004	**46**
type-I	*A. baumannii*	bau-B2	XH673	LYJQ01000014	**46**
type-I	*A. baumannii*	bau-B2	XH668	LYJV01000011	**46**
type-I	*A. baumannii*	bau-B2	XH664	LYJZ01000023	**46**
type-I	*A. baumannii*	bau-B2	XH662	LYKB01000643	**46**
type-I	*A. baumannii*	bau-B2	XH661	LYKC01000016	**46**
type-I	*A. baumannii*	bau-B2	XH648	LYKH01000023	**46**
type-I	*A. baumannii*	bau-B2	XH647	LYKI01000034	**46**
type-I	*A. baumannii*	bau-B2	XH642	LYKN01000014	**46**
type-I	*A. baumannii*	bau-B2	XH641	LYKO01000034	**46**
type-I	*A. baumannii*	bau-B2	XH553	LYKU01000022	**46**
type-I	*A. baumannii*	bau-B2	XH545	LYLC01000121	**46**
type-I	*A. baumannii*	bau-B2	XH512	LYLG01000108	**46**

type–I	*A. baumannii*	bau–B2	ATCC19606	ACQB01000024.1	**52**
type–I	*A. baumannii*	bau–B2	ATCC19606, JCM841 str.	APRG01000014.1	**52**
type–I	*A. baumannii*	bau–B2	ATCC19606	JMRY01000015	**52**
type–I	*A. baumannii*	bau–B2	MSP4–16	AODW01000028.1	**52**
type–I	*A. baumannii*	bau–B2	GTC03324	BBNH01000005.1	**52**
type–I	*A. baumannii*	bau–B2	GTC03329	BBNJ01000004.1	**52**
type–I	*A. baumannii*	bau–B2	NBRC110493	BBOQ01000003.1	**52**
type–I	*A. baumannii*	bau–B2	NBRC110495	BBOR01000009.1	**52**
type–I	*A. baumannii*	bau–B2	GTC03325	BBSP01000005.1	**52**
type–I	*A. baumannii*	bau–B2	NBRC110494	BBTE01000006.1	**52**
type–I	*A. baumannii*	bau–B2	NBRC109757	BBTN01000005.1	**52**
type–I	*A. baumannii*	bau–B2	ab736	NZ_CP015121.1	**52**
type–I	*A. baumannii*	bau–B2	1062314	JFDR01000005	**54**
type–I	*A. baumannii*	bau–B2	3909	AEOZ01000177	**78**
type–I	*A. baumannii*	bau–B2	TG22142	ASFL01000019	**78**
type–I	*A. baumannii*	bau–B2	TG22146	ASFM01000029	**78**
type–I	*A. baumannii*	bau–B2	TG22150	ASFO01000048	**78**
type–I	*A. baumannii*	bau–B2	UH5207	AYFP01000011.1	**78**
type–I	*A. baumannii*	bau–B2	68SM01	FUEK01000017	**78**
type–I	*A. baumannii*	bau–B2	61SM01	FUEL01000016	**78**
type–I	*A. baumannii*	bau–B2	103SM	FUEM01000017	**78**
type–I	*A. baumannii*	bau–B2	14336	FUEN01000043	**78**
type–I	*A. baumannii*	bau–B2	MGTN	FUEO01000015	**78**
type–I	*A. baumannii*	bau–B2	96SM	FUEP01000020	**78**
type–I	*A. baumannii*	bau–B2	20C15	FUEQ01000051	**78**
type–I	*A. baumannii*	bau–B2	25C30	FUER01000050	**78**
type–I	*A. baumannii*	bau–B2	74SM01	FUES01000018	**78**
type–I	*A. baumannii*	bau–B2	MONUR	FUET01000019	**78**
type–I	*A. baumannii*	bau–B2	2RED09	FUEU01000017	**78**
type–I	*A. baumannii*	bau–B2	65SM01	FUEV01000047	**78**
type–I	*A. baumannii*	bau–B2	2MG	FUEX01000028	**78**
type–I	*A. baumannii*	bau–B2	5MO	FUEY01000055	**78**
type–I	*A. baumannii*	bau–B2	1096934	JEXM01000065	**78**
type–I	*A. baumannii*	bau–B2	831240	JEYO01000017	**78**
type–I	*A. baumannii*	bau–B2	855125	JMNT01000054	**78**
type–I	*A. baumannii*	bau–B2	UH17_52	JWYK03000038.1	**78**
type–I	*A. baumannii*	bau–B2	ABBL025	LLDF01000012	**78**
type–I	*A. baumannii*	bau–B2	ABBL026	LLDG01000302	**78**
type–I	*A. baumannii*	bau–B2	ABUH393	MSMS01000003	**78**
type–I	*A. baumannii*	bau–B2	PR308	NGBY01000015	**78**
type–I	*A. baumannii*	bau–B2	PR355	NGCI01000007	**78**
type–I	*A. baumannii*	bau–B2	PR371	NGCY01000042	**78**
type–I	*A. baumannii*	bau–B2	PR385	NGES01000073	**78**
type–I	*A. baumannii*	bau–B2	PR391	NGEY01000012.1	**78**
type–I	*A. baumannii*	bau–B2	72SM01	FUEW01000053	**78**
type–I	*A. baumannii*	bau–B2	B11911	CP021345.2	**149**
type–I	*A. baumannii*	bau–B2	1295549	JFXB01000002	**652**
type–I	*A. baumannii*	bau–B2	277047	JFXS01000010	**652**
type–I	*A. baumannii*	bau–B2	426863	JFYF01000002	**652**
type–I	*A. baumannii*	bau–B2	UH6507	AYFK01000017	**669**
type–I	*A. baumannii*	bau–B2	86II/2C	NIWI01000026	**690**
type–I	*A. baumannii*	bau–B2	Pr310	NGCA01000028	**810**
type–I	*A. baumannii*	bau–B2	RB–1399	MPPK01000005.1	**920**
type–I	*A. baumannii*	bau–B2	TG19617	AMID01000010.1	**438**
type–I	*A. baumannii*	bau–B2	IOMTU433	NZ_AP014649.1	**622**
type–I	*A. baumannii*	bau–B2	AB360	NGKM01000010	**622**
type–I	*A. baumannii*	bau–B2	XH729	LYHW01000020	**nd**
type–I	*A. baumannii*	bau–B2	XH703	LYIS01000052	**nd**
type–I	*A. baumannii*	bau–B3	1267820	JEWD01000091.1	**103**
type–I	*A. baumannii*	bau–B3	ABBL038	LLDS01000070.1	**nd**
type–I	*A. baumannii*	bau–B3	ABBL085	LLGN01000115.1	**103**
type–I	*A. baumannii*	bau–B3	AB031	NZ_CP009256.1	**638**
type–I	*A. baumannii*	bau–B3	NIPH 67	APRA01000005.1	**35**
type–I	*A. baumannii*	bau–B3	AB_2007–16–25–01–7	AMHI01000023.1	**241**
type–I	*A. baumannii*	bau–B3	AB_2007–16–27–01	AMHJ01000020.1	**241**
type–I	*A. baumannii*	bau–B3	AB_TG27339	AMIR01000011.1	**241**
type–I	*A. baumannii*	bau–B3	118362	JEWB01000054.1	**241**
type–I	*A. baumannii*	bau–B4	NIPH 601	APQZ01000007.1	**40**
type–I	*A. baumannii*	bau–B4	GTC 03322	BBNF10000060.1	**40**

type-I	*A. baumannii*	bau-B4	GTC 03323	BBNG01000061.1	**40**
type-I	*A. baumannii*	bau-B4	GTC 03328	BBNI01000064.1	**40**
type-I	*A. baumannii*	bau-B4	NBRC 110489	BBOP01000068.1	**40**
type-I	*A. baumannii*	bau-B4	NBRC 110491	BBTC01000062.1	**40**
type-I	*A. baumannii*	bau-B4	TYTH-7	AGSV01000139.1	**40**
type-I	*A. baumannii*	bau-B4	LY1	JDSV01000226.1	**40**
type-I	*A. baumannii*	bau-B4	LY2	JDSW01000052.1	**40**
type-I	*A. baumannii*	bau-B4	3.5D	MABZ01000006.1	**388**
type-I	*A. baumannii*	bau-B4	ABUH513	MSPL01000024.1	**941**
type-I	*A. baumannii*	bau-B4	ARLG1862	NGGF01000027.1	**941**
type-I	*A. baumannii*	bau-B4	219_ABAU	JVPN01000108.1	**1140**
type-I	*A. baumannii*	bau-B4	ABBL110	LLHI01000055.1	**nd**
type-I	*A. baumannii*	bau-B4	ATCC 17945	MTGG01000053.1	**1168**
type-I	*A. baumannii*	bau-B4	PR360	NGCN01000030	**494**
type-I	*A. baumannii*	bau-B4	NCTC7364	NZ_LT605059.1	**494**
type-I	*A. baumannii*	bau-C1	MSP4-16	AODW01000010.1	**52**
type-I	*A. baumannii*	bau-C1	ATCC19606, JCM841 str.	APRG01000013.1	**52**
type-I	*A. baumannii*	bau-C1	GTC03324	BBNH01000013.1	**52**
type-I	*A. baumannii*	bau-C1	GTC03329	BBNJ01000012.1	**52**
type-I	*A. baumannii*	bau-C1	NBRC110493	BBOQ01000026.1	**52**
type-I	*A. baumannii*	bau-C1	NBRC110495	BBOR01000002.1	**52**
type-I	*A. baumannii*	bau-C1	GTC03325	BBSP01000021.1	**52**
type-I	*A. baumannii*	bau-C1	NBRC110494	BBTE01000023.1	**52**
type-I	*A. baumannii*	bau-C1	NBRC109757	BBTN01000002.1	**52**
type-I	*A. baumannii*	bau-C1	ATCC 19606	JMRY01000018.1	**52**
type-I	*A. baumannii*	bau-C1	ab736	NZ_CP015121.1	**52**
type-I	*A. baumannii*	bau-C1	1417041	JEYP01000023.1	**108**
type-I	*A. baumannii*	bau-C1	TG19617	AMID01000004.1	**438**
type-I	*A. baumannii*	bau-C2	ACICU	CP000863	**2**
type-I	*A. baumannii*	bau-C3	625974	JEXD01000018.1	**509**
type-I	*A. baumannii*	bau-C3	B8342	NZ_CP021342	**dsv 509**
type-I	*A. baumannii*	bau-C3	ZW85-1	NC_023028.1	**639**
type-I	*A. baumannii*	bau-C3	ARLG1846	NGFV01000032	**509**
type-I	*A. baumannii*	bau-C4	UH19608	AYFZ01000050.1	**79**
type-I	*A. baumannii*	bau-C4	ABBL006	LLCM01000033.1	**79**
type-I	*A. baumannii*	bau-C4	ABBL017	LLCX01000003.1	**79**
type-I	*A. baumannii*	bau-C4	ABBL023	LLDD01000014.1	**79**
type-I	*A. baumannii*	bau-C4	ABBL039	LLDT01000099.1	**79**
type-I	*A. baumannii*	bau-C4	ABBL043	LLDX01000088.1	**79**
type-I	*A. baumannii*	bau-C4	ABBL049	LLED01000076.1	**79**
type-I	*A. baumannii*	bau-C4	ABBL052	LLEG01000087.1	**79**
type-I	*A. baumannii*	bau-C4	ABBL055	LLFA01000014.1	**79**
type-I	*A. baumannii*	bau-C4	ABBL056	LLFB01000081.1	**79**
type-I	*A. baumannii*	bau-C4	ABBL067a	LLFN01000302.1	**79**
type-I	*A. baumannii*	bau-C4	ABBL067b	LLFO01000337.1	**79**
type-I	*A. baumannii*	bau-C4	ABBL067j	LLFV01000119.1	**79**
type-I	*A. baumannii*	bau-C4	ABBL067k	LLFW01000018.1	**79**
type-I	*A. baumannii*	bau-C4	ABBL067l	LLFX01000008.1	**79**
type-I	*A. baumannii*	bau-C4	ABBL068	LLFY01000131.1	**79**
type-I	*A. baumannii*	bau-C4	ABBL069	LLFZ01000242.1	**79**
type-I	*A. baumannii*	bau-C4	ABBL101	LLHB01000161.1	**79**
type-I	*A. baumannii*	bau-C4	ABOB01	LLIQ01000015.1	**79**
type-I	*A. baumannii*	bau-C4	ABOB03	LLIS01000357.1	**79**
type-I	*A. baumannii*	bau-C4	ABOB04	LLIT01000019.1	**79**
type-I	*A. baumannii*	bau-C4	ABOB04_a	LLIU01000391.1	**79**
type-I	*A. baumannii*	bau-C4	ABOB06	LLIW01000172.1	**79**
type-I	*A. baumannii*	bau-C4	ABOB08	LLIZ01000033.1	**79**
type-I	*A. baumannii*	bau-C4	ABOB12	LLJD01000318.1	**79**
type-I	*A. baumannii*	bau-C4	ABOBEN	LLJG01000005.1	**79**
type-I	*A. baumannii*	bau-C4	Ab28	LMBQ01000020.1	**79**
type-I	*A. baumannii*	bau-C4	CU032113	LXYW01000100.1	**79**
type-I	*A. baumannii*	bau-C4	ABUH564	MSOI01000074.1	**79**
type-I	*A. baumannii*	bau-C4	499	MWPK01000224.1	**79**
type-I	*A. baumannii*	bau-C4	923	MWPO01000235.1	**79**
type-I	*A. baumannii*	bau-C4	ABUH505	NCXV01000134.1	**79**
type-I	*A. baumannii*	bau-C4	ABUH428	NCYV01000120.1	**79**
type-I	*A. baumannii*	bau-C4	ARLG-1775	NEQF01000053.1	**79**
type-I	*A. baumannii*	bau-C4	ARLG-1860	NEQK01000094.1	**79**
type-I	*A. baumannii*	bau-C4	AF-401	NZ_CP018254.1	**79**

type-I	*A. baumannii*	bau-C4	ABBL030	LLDK01000140.1	**79**
type-I	*A. baumannii*	bau-C4	PR307	NGBX01000017.1	**79**
type-I	*A. baumannii*	bau-C4	PR315	NGEM01000008.1	**150**
type-I	*A. baumannii*	bau-C4	ARLG1800	NGGR01000007.1	**150**
type-I	*A. baumannii*	bau-C4	ARLG1936	NGIK01000038.1	**150**
type-I	*A. baumannii*	bau-C4	TG02011	ASES01000060.1	**422**
type-I	*A. baumannii*	bau-C4	UH7607	AYFH01000090.1	**422**
type-I	*A. baumannii*	bau-C4	UH6907	AYFJ01000036.1	**422**
type-I	*A. baumannii*	bau-C4	UH22908	AYFV01000057.1	**422**
type-I	*A. baumannii*	bau-C4	UH16208	AYGB01000015.1	**422**
type-I	*A. baumannii*	bau-C4	UH12208	AYGK01000135.1	**422**
type-I	*A. baumannii*	bau-C4	CCBH15815Y_43	NLCO01000043.1	**730**
type-I	*A. baumannii*	bau-C5	86II/2C	NIWI01000005.1	**690**
type-I	*A. baumannii*	bau-C5	O1D3-2	NIWQ01000007.1	**240**
type-I	*A. baumannii*	bau-C5	PLG9P835	NIWR01000008.1	**1179**
type-I	*A. pittii*	pit-A3	IEC338SC	NZ_CP015145.1	**1076**
type-I	*A. pittii*	pit-A4	PR320	NGDK01000053.1	**119**
type-I	*A. pittii*	pit-A4	NBRC 110504	BBTQ01000063.1	**119**
type-I	*A. pittii*	pit-A4	NBRC 110507	BBTZ01000081.1	**119**
type-I	*A. pittii*	pit-A4	NBRC 110509	BBUA01000067.1	**119**
type-I	*A. pittii*	pit-A4	ABBL111	LLHJ01002199.1	**119**
type-I	*A. pittii*	pit-A4/C7/A4	St-12537-91	MDIL01000050.1	**70**
type-I	*A. pittii*	pit-A5	Ac. sp. 1294243	JFXG01000010.1	**214**
type-I	*A. pittii*	pit-A5	Ac. sp. 1264765	JMNG01000024.1	**220**
type-I	*A. pittii*	pit-A5	St-19650-90	MDHQ01000034.1	**747**
type-I	*A. pittii*	pit-A5	ABUH540	MSOC01000021.1	**1175**
type-I	*A. pittii*	pit-A5	ARLG1807	NGGW01000019.1	**1176**
type-I	*A. pittii*	pit-A5	ARLG1875	NGJC01000033.1	**nd**
type-I	*A. pittii*	pit-A5	TCM292	LSAK01000014.1	**207**
type-I	*A. pittii*	pit-A5/A4/A5	A. sp. 907131 (*)	JEXS01000005.1	**220**
type-I	*A. pittii*	pit-A5/A4/A5	FC8876	LBCP01000024.1	**643**
type-I	*A. pittii*	pit-A5/A4/A5	ABBL033	LLDN01000009.1	**1170**
type-I	*A. pittii*	pit-A5/A4/A5	ABBL098	LLGZ01000237.1	**1177**
type-I	*A. pittii*	pit-A5/C7/A5	PR301	NGBT01000013.1	**457**
type-I	*A. pittii*	pit-A5/Hn1/A5	UKK-0546	MDIJ01000089.1	**776**
type-I	*A. pittii*	pit-A5/Hn2/A5	DSM 21653	AIEK01000183.1	**63**
type-I	*A. pittii*	pit-A5/Hn2/A5	DSM 25618	BBST01000016.1	**63**
type-I	*A. pittii*	pit-A5/Hn2/A5	ATCC 19004	APQP01000005.1	**63**
type-I	*A. pittii*	pit-A5/Hn2/A5	DSM 9306	AIEF01000274.1	**64**
type-I	*A. pittii*	pit-A5/Hn2/A5	St-10592-91	MDHO01000041.1	**64**
type-I	*A. pittii*	pit-A5/Hn2/A5	St-20772-90	MDHR01000015.1	**64**
type-I	*A. pittii*	pit-A5/Hn2/A5	St-14379-92	MDIW01000070.1	**64**
type-I	*A. pittii*	pit-A5/Hn2/A5	PR386	NGET01000034.1	**248**
type-I	*A. pittii*	pit-A5/Hn2/A5	UKK-0548	MDIM01000262.1	**248**
type-I	*A. pittii*	pit-A5/Hn2/A5	UKK-0556	MDIU01000045.1	**248**
type-I	*A. pittii*	pit-A5/Hn2/A5	ARLG1839	NGHJ01000050.1	**396**
type-I	*A. pittii*	pit-A5/Hn2/A5	PR368	NGCV01000008.1	**744**
type-I	*A. pittii*	pit-A5/Hn2/A5	St-12828-92	MDIV01000078.1	**744**
type-I	*A. pittii*	pit-A5/Hn2/A5	NBRC 110508	BBTR01000017.1	**1178**
type-I	*Acb complex*	pit-A5/Hn2/A5	NIPH 817	APPF01000024.1	**88**
type-I	*A. pittii*	pit-A5/C8/A5	ARLG1933	NGII01000017.1	**1165**
type-I	*A. pittii*	pit-B5	D499	AGFH01000024.1	**63**
type-I	*A. pittii*	pit-B5	ATCC 19004 = CIP 70.29	APQP01000005.1	**63**
type-I	*A. pittii*	pit-B5	DSM 25618	BBST01000007.1	**63**
type-I	*A. pittii*	pit-B5	UKK-0539	MDIC01000039.1	**63**
type-I	*A. pittii*	pit-B5	UKK-0541	MDIE01000040.1	**63**
type-I	*A. pittii*	pit-B5	UKK-0542	MDIF01000057.1	**63**
type-I	*A. pittii*	pit-B5	UKK-0543	MDIG01000029.1	**63**
type-I	*A. pittii*	pit-B5	UKK-0547	MDIK01001418.1	**63**
type-I	*A. pittii*	pit-B5	UKK-0551	MDIP01000101.1	**63**
type-I	*A. pittii*	pit-B5	St-10592-91	MDHO01000002.1	**64**
type-I	*A. pittii*	pit-B5	St-20772-90	MDHR01000085.1	**64**
type-I	*A. pittii*	pit-B5	UKK-0327	MDHW01000161.1	**64**
type-I	*A. pittii*	pit-B5	St-14379-92	MDIW01000004.1	**64**
type-I	*A. pittii*	pit-B5	PR313	NGCD01000013.1	**64**
type-I	*A. pittii*	pit-B5	PR370	NGCX01000007.1	**64**
type-I	*A. pittii*	pit-B5	ARLG1803	NGGT01000052.1	**64**
type-I	*A. pittii*	pit-B5	St-12537-91	MDIL01000050.1	**70**
type-I	*A. pittii*	pit-B5	Ac sp. SH024	ADCH01000014.1	**93**

type-I	*A. pittii*	pit-B5	ABBL010	LLCQ01000051.1	**93**
type-I	*A. pittii*	pit-B5	ABBL047	LLEB01000028.1	**93**
type-I	*A. pittii*	pit-B5	ABBL065	LLFK01000010.1	**93**
type-I	*A. pittii*	pit-B5	ABBL103	LLHD01000155.1	**93**
type-I	*A. pittii*	pit-B5	St-11469-92	MDHP01000045.1	**93**
type-I	*A. pittii*	pit-B5	UKK-0245	MDHT01000134.1	**93**
type-I	*A. pittii*	pit-B5	UKK-0544	MDIH01000054.1	**93**
type-I	*A. pittii*	pit-B5	UKK-0555	MDIT01000139.1	**93**
type-I	*A. pittii*	pit-B5	St-14569-91	MDIX01000009.1	**93**
type-I	*A. pittii*	pit-B5	St-15559-91	MDIY01000035.1	**93**
type-I	*A. pittii*	pit-B5	St-15639-91	MDIZ01000031.1	**93**
type-I	*A. pittii*	pit-B5	ABUH403	NDFE01000196.1	**93**
type-I	*A. pittii*	pit-B5	ARLG1776	NGGH01000095.1	**93**
type-I	*A. pittii*	pit-B5	ARLG1798	NGGQ01000048.1	**93**
type-I	*A. pittii*	pit-B5	IHIT32685	NWVY01000021.1	**93**
type-I	*A. pittii*	pit-B5	IHIT32473	NWVZ01000019.1	**93**
type-I	*A. pittii*	pit-B5	UKK-0548	MDIM01000224.1	**248**
type-I	*A. pittii*	pit-B5	UKK-0556	MDIU01000067.1	**248**
type-I	*A. pittii*	pit-B5	PR386	NGET01000049.1	**248**
type-I	*A. pittii*	pit-B5	UBA2478	DDPC01000026.1	**396**
type-I	*A. pittii*	pit-B5	ARLG1839	NGHJ01000054.1	**396**
type-I	*A. pittii*	pit-B5	ARLG1768	NGHT01000057.1	**396**
type-I	*A. pittii*	pit-B5	PR301	NGBT01000034.1	**457**
type-I	*A. pittii*	pit-B5	ANC 3678	APQN01000012.1	**640**
type-I	*A. pittii*	pit-B5	UKK-0545	MDII01000018.1	**744**
type-I	*A. pittii*	pit-B5	PR368	NGCV01000022.1	**744**
type-I	*A. pittii*	pit-B5	PR339	NGEB01000042.1	**744**
type-I	*A. pittii*	pit-B5	St-19650-90	MDHQ01000073.1	**747**
type-I	*A. pittii*	pit-B5	NBRC 110508	BBTR01000005.1	**1178**
type-I	*A. pittii*	pit-B5	NBRC 110510	BBTS01000015.1	**1172**
type-I	*A. pittii*	pit-B5	ABBL042	LLDW01000017.1	**nd**
type-I	*A. pittii*	pit-B5	ABBL074	LLGE01000164.1	**nd**
type-I	*A. pittii*	pit-B5	XH765	LYGR01000024.1	**nd**
type-I	*A. pittii*	pit-B5	ABUH540	MSOC01000020.1	**1175**
type-I	*A. pittii*	pit-B5	ARLG1771	NGGG01000163.1	**1173**
type-I	*A. pittii*	pit-B5	ARLG1875	NGJC01000005.1	**nd**
type-I	*A. pittii*	pit-B5	42F	CBRO020000065.1	**nd**
type-I	*A. pittii*	pit-B5	IHIT29592	NWVA01000016.1	**nd**
type-I	*A. pittii*	pit-B5	IHIT29469	NWVB01000006.1	**nd**
type-I	*A. pittii*	pit-B5	IHIT24944	NWVC010000081	**nd**
type-I	*A. pittii*	bau-B3/pit-B5	269	JQNV01000053.1	**119**
type-I	*A. pittii*	bau-B3/pit-B5	NBRC 110507	BBTZ01000082	**119**
type-I	*A. pittii*	bau-B3/pit-B5	NBRC 110509	BBUA01000069	**119**
type-I	*A. pittii*	bau-B3/pit-B5	NBRC 110504	BBTQ01000064	**119**
type-I	*A. pittii*	bau-B3/pit-B5	CR12-42	JQNT01000051	**119**
type-I	*A. pittii*	bau-B3/pit-B5	A. sp. 2027	AMJN01000042	**119**
type-I	*A. pittii*	bau-B3/pit-B5	PR348	NGEJ01000058	**119**
type-I	*A. pittii*	bau-B3/pit-B5	A. sp. 1295259	JEWG01000043	**119**
type-I	*A. pittii*	bau-B3/pit-B5	TCM292	LSAK01000022	**207**
type-I	*A. pittii*	bau-B3/pit-B5	A. sp. 907131	JEXS01000003.1	**220**
type-I	*A. pittii*	bau-B3/pit-B5	A. sp. 883425	JFXZ01000006.1	**220**
type-I	*A. pittii*	bau-B3/pit-B5	NBRC 110506	BBTY01000009	**220**
type-I	*A. pittii*	bau-B3/pit-B5	NBRC 110505	BBTX01000009	**220**
type-I	*A. pittii*	bau-B3/pit-B5	A. sp. 809848	JEXG01000029.1	**666**
type-I	*A. pittii*	bau-B3/pit-B5	UKK-0546	MDIJ01000142	**776**
type-I	*A. pittii*	bau-B3/pit-B5	UKK-0540	MDID01000164	**778**
type-I	*A. baumannii*	bau-B3/pit-B5	NIPH 329	APQY01000009.1	**11**
type-I	*A. pittii*	bau-C3/new CT	792879	JFYC01000001	**1167**
type-I	*A. baumannii*	bau-C3/new CT	29D2	MABW01000006	**309**
type-I	*A. pittii*	PIT-B6	PR366	NGCT01000027.1	**312**
type-I	*A. pittii*	PIT-B6	ARLG1933	NGII01000026	**1165**
type-I	*A. pittii*	PIT-B6	T167	JRQZ01000033.1	**655**
type-I	*A. pittii*	PIT-B6	ARLG1942	NGIO01000041.1	**795**
type-I	*A. pittii*	PIT-B6	ARLG1869	NGIX01000002.1	**795**
type-I	*A. pittii*	PIT-B6	IEC338SC	NZ_CP015145.1	**1076**
type-I	*A. pittii*	PIT-B6	792879	JFYC01000021	**1167**
type-I	*A. pittii*	pit-B7	ABBL098	LLGZ01000054.1	**1177**
type-I	*A. pittii*	pit-B7/pit-B5	PR320	NGDK01000009.1	**119**
type-I	*A. pittii*	pit-B7/pit-B5	ABBL135	LLIF01000327.1	**119**

type-I	*A. pittii*	pit-B7/pit-B5	ABBL111	LLHJ01002637.1	**119**
type-I	*A. pittii*	pit-B7/pit-B5	IPK_TSA6.1	LWHP01000001.1	**321**
type-I	*A. pittii*	pit-B7/pit-B5	FC8876	LBCP01000088.1	**643**
type-I	*A. pittii*	pit-B7/pit-B5	ABBL046	LLEA01000010.1	**643**
type-I	*A. pittii*	pit-B7/pit-B5	ABBL024	LLDE01000039.1	**643**
type-I	*A. pittii*	pit-B7/pit-B5	UBA2484	DDOW01000009.1	**1171**
type-I	*A. pittii*	pit-B7/pit-B5	UBA2608	DDKC01000097.1	**nd**
type-I	*A. pittii*	pit-B7/pit-B5	ARLG1957	NGIS01000052.1	**1169**
type-I	*A. pittii*	pit-C5	A. sp. 1295259	JEWG01000048.1	**119**
type-I	*A. pittii*	pit-C5	A. baumannii 269	JQNV01000054.1	**119**
type-I	*A. pittii*	pit-C5	AP_882	NZ_CP014477.1	**119**
type-I	*A. pittii*	pit-C5	ABBL111	LLHJ01000059	**119**
type-I	*A. pittii*	pit-C5	ABBL015	LLCV01000009	**119**
type-I	*A. pittii*	pit-C5	PR348	NGEJ01000078	**119**
type-I	*A. pittii*	pit-C5	CR12-42	JQNT01000088	**119**
type-I	*A. pittii*	pit-C5	UKK-0550	MDIO01000081	**119**
type-I	*A. pittii*	pit-C5	UKK-0536	MDHZ01000127	**119**
type-I	*A. pittii*	pit-C5	NBRC 110506	BBTY01000007	**220**
type-I	*A. pittii*	pit-C5	IPK_TSA6.1	LWHP01000001	**321**
type-I	*A. pittii*	pit-C5	ABBL024	LLDE01000066	**643**
type-I	*A. pittii*	pit-C5	ARLG 1957	NGIS01000002	**1169**
type-I	*A. pittii*	pit-C5	ABBL033	LLDN01000069	**1170**
type-I	*A. pittii*	pit-C5	UBA 2484	DDOW01000010	**1171**
type-I	*A. pittii*	pit-C6	DSM25618	BBST01000014.1	**63**
type-I	*A. pittii*	pit-C6	UKK-0547	MDIK01001336.1	**63**
type-I	*A. pittii*	pit-C6	ATCC19004	APQP01000003.1	**63**
type-I	*A. pittii*	pit-C6	ABBL126	LLHW01000293.1	**64**
type-I	*A. pittii*	pit-C6	UKK-0327	MDHW01000178.1	**64**
type-I	*A. pittii*	pit-C6	IIF1SW-P1	MIZX01000011.1	**64**
type-I	*A. pittii*	pit-C6	PR313	NGCD01000037.1	**64**
type-I	*A. pittii*	pit-C6	PR370	NGCX01000020.1	**64**
type-I	*A. pittii*	pit-C6	ARLG1779	NGGI01000025.1	**64**
type-I	*A. pittii*	pit-C6	ARLG1803	NGGT01000062.1	**64**
type-I	*A. pittii*	pit-C6	DSM9306	AIEF01000088.1	**64**
type-I	*A. pittii*	pit-C6	ABBL128	LLHY01001554.1	**64**
type-I	*A. pittii*	pit-C6	St-10592-91	MDHO01000070.1	**64**
type-I	*A. pittii*	pit-C6	St-20772-90	MDHR01000065.1	**64**
type-I	*A. pittii*	pit-C6	St-14379-92	MDIW01000067.1	**64**
type-I	*A. pittii*	pit-C6	Ac sp. SH024	ADCH01000005.1	**93**
type-I	*A. pittii*	pit-C6	ABBL010	LLCQ01000056.1	**93**
type-I	*A. pittii*	pit-C6	ABBL047	LLEB01000058.1	**93**
type-I	*A. pittii*	pit-C6	ABBL065	LLFK01000065.1	**93**
type-I	*A. pittii*	pit-C6	ABBL096	LLGX01000066.1	**93**
type-I	*A. pittii*	pit-C6	ABBL120	LLHQ01000819.1	**93**
type-I	*A. pittii*	pit-C6	St-11469-92	MDHP01000046.1	**93**
type-I	*A. pittii*	pit-C6	UKK-0245	MDHT01000136.1	**93**
type-I	*A. pittii*	pit-C6	UKK-0544	MDIH01000005.1	**93**
type-I	*A. pittii*	pit-C6	UKK-0555	MDIT01000070.1	**93**
type-I	*A. pittii*	pit-C6	St-14569-91	MDIX01000086.1	**93**
type-I	*A. pittii*	pit-C6	St-15559-91	MDIY01000009.1	**93**
type-I	*A. pittii*	pit-C6	St-15639-91	MDIZ01000006.1	**93**
type-I	*A. pittii*	pit-C6	ABUH403	NDFE01000063.1	**93**
type-I	*A. pittii*	pit-C6	PR369	NGCW01000047.1	**93**
type-I	*A. pittii*	pit-C6	PR318	NGDI01000015.1	**93**
type-I	*A. pittii*	pit-C6	PR346	NGEH01000075.1	**93**
type-I	*A. pittii*	pit-C6	ARLG1776	NGGH01000045.1	**93**
type-I	*A. pittii*	pit-C6	ARLG1798	NGGQ01000032.1	**93**
type-I	*A. pittii*	pit-C6	ARLG1804	NGGU01000039.1	**93**
type-I	*A. pittii*	pit-C6	ABBL103	LLHD01000160.1	**93**
type-I	*A. pittii*	pit-C6	CEB-Ap	NGAB01000018.1	**93**
type-I	*A. pittii*	pit-C6	IHIT32685	NWVY01000037.1	**93**
type-I	*A. pittii*	pit-C6	IHIT32473	NWVZ01000006.1	**93**
type-I	*A. pittii*	pit-C6	IHIT29592	NWWA01000059.1	**93**
type-I	*A. pittii*	pit-C6	IHIT29469	NWWB01000013.1	**93**
type-I	*A. pittii*	pit-C6	IHIT24944	NWWC01000020.1	**93**
type-I	*A. pittii*	pit-C6	ABUH411	MSMX01000037.1	**248**
type-I	*A. pittii*	pit-C6	UKK-0548	MDIM01000175.1	**248**
type-I	*A. pittii*	pit-C6	UKK-0556	MDIU01000051.1	**248**
type-I	*A. pittii*	pit-C6	PR386	NGET01000030.1	**248**

type-I	*A. pittii*	pit-C6	UBA2478	DDPC01000006.1	**396**
type-I	*A. pittii*	pit-C6	ARLG1839	NGHJ01000073.1	**396**
type-I	*A. pittii*	pit-C6	ARLG1768	NGHT01000085.1	**396**
type-I	*A. pittii*	pit-C6	St-12828-92	MDIV01000077.1	**744**
type-I	*A. pittii*	pit-C6	PR368	NGCV01000039.1	**744**
type-I	*A. pittii*	pit-C6	PR339	NGEB01000026.1	**744**
type-I	*A. pittii*	pit-C6	NBRC 110510	BBTS01000017.1	**1172**
type-I	*A. pittii*	pit-C6	ABBL005	LLCL01000051.1	**nd**
type-I	*A. pittii*	pit-C6	XH765	LYGR01000031.1	**nd**
type-I	*A. pittii*	pit-C6	ARLG1771	NGGG01000209.1	**1173**
type-I	*A. pittii*	pit-C6	ARLG1961	NGIT01000049.1	**1174**
type-I	*A. pittii*	pit-C7	PHEA-2	NC_016603.1	**1164**
type-I	*A. pittii*	pit-C7	A. sp. 1542444	JEYA01000001.1	**675**
type-I	*A. baumannii*	pit-C7-like	8D1	MABV01000005.1	**512**
type-I	*A. nosocomialis*	nos-C8	GTC 03313	BBTJ01000037.1	**68**
type-I	*A. nosocomialis*	nos-C8	NBRC 110501	BBTO01000034.1	**68**
type-I	*A. nosocomialis*	nos-C8	NBRC 110503	BBTP01000032.1	**68**
type-I	*A. nosocomialis*	nos-C8	NBRC 110502	BBTW01000035.1	**68**
type-I	*A. nosocomialis*	nos-C8	UBA3105	DFAN01000123.1	**nd**
type-I	*A. nosocomialis*	nos-C8	UBA3966	DGCY01000018.1	**68**
type-I	*A. nosocomialis*	nos-C8	A. sp. 1245593	JFDW01000009.1	**68**
type-I	*A. nosocomialis*	nos-C8	A. sp. 1281984	JFXD01000005.1	**68**
type-I	*A. nosocomialis*	nos-C8	446_ABAU	JVGT01000017.1	**68**
type-I	*A. nosocomialis*	nos-C8	A. sp. FDAARGOS_131	LORV01000006.1	**68**
type-I	*A. nosocomialis*	nos-C8	XH796	LYGC01000035.1	**nd**
type-I	*A. nosocomialis*	nos-C8	XH762	LYGU01000043.1	**68**
type-I	*A. nosocomialis*	nos-C8	XH654	LYKD01000035.1	**68**
type-I	*A. nosocomialis*	nos-C8	XH653	LYKE01000040.1	**68**
type-I	*A. nosocomialis*	nos-C8	XH551	LYKW01000018.1	**68**
type-I	*A. nosocomialis*	nos-C8	HJ14	MADF01000010.1	**217**
type-I	*A. nosocomialis*	nos-C8	AN2605	NNSH01000193.1	**68**
type-I	*A. nosocomialis*	nos-C9	SSA3	NZ_CP020588.1	**433**
type-I	*A. nosocomialis*	nos-C9	UBA2042	DDBC01000042.1	**1166**
type-I	*A. nosocomialis*	nos-C9	TG21145	AMJH01000036.1	**74**
type-I	*A. nosocomialis*	nos-C9	NCTC 8102	AIEJ01000063.1	**74**
type-I	*A. calcoaceticus*	cal-C10	ANC3680	NZ_KB849752.1	**539**
type-I	*A. calcoaceticus*	cal-C11	RUH2202	ACPK01000025.1	**92**
type-II	*A. baumannii*	bau-D1	3909	AEOZ01000025.1	**78**
type-II	*A. baumannii*	bau-D1	TG22142	ASFL01000035.1	**78**
type-II	*A. baumannii*	bau-D1	TG22146	ASFM01000027.1	**78**
type-II	*A. baumannii*	bau-D1	TG22150	ASFO01000033.1	**78**
type-II	*A. baumannii*	bau-D1	1096934	JEXM01000056.1	**78**
type-II	*A. baumannii*	bau-D1	831240	JEYO01000006.1	**78**
type-II	*A. baumannii*	bau-D1	855125	JMNT01000076.1	**78**
type-II	*A. baumannii*	bau-D1	ABBL025	LLDF01000014.1	**78**
type-II	*A. baumannii*	bau-D1	ABBL026	LLDG01000617.1	**78**
type-II	*A. baumannii*	bau-D1	UH5207	AYFP01000025.1	**78**
type-II	*A. baumannii*	bau-D1	UH17_52	JWYK03000080.1	**78**
type-II	*A. baumannii*	bau-D1	68SM01	FUEK01000010.1	**78**
type-II	*A. baumannii*	bau-D1	61SM01	FUEL01000006.1	**78**
type-II	*A. baumannii*	bau-D1	103SM	FUEM01000001.1	**78**
type-II	*A. baumannii*	bau-D1	14336	FUEN01000014.1	**78**
type-II	*A. baumannii*	bau-D1	MGTN	FUEO01000003.1	**78**
type-II	*A. baumannii*	bau-D1	96SM	FUEP01000001.1	**78**
type-II	*A. baumannii*	bau-D1	20C15	FUEQ01000001.1	**78**
type-II	*A. baumannii*	bau-D1	25C30	FUER01000002.1	**78**
type-II	*A. baumannii*	bau-D1	74SM01	FUES01000003.1	**78**
type-II	*A. baumannii*	bau-D1	MONUR	FUET01000004.1	**78**
type-II	*A. baumannii*	bau-D1	2RED09	FUEU01000004.1	**78**
type-II	*A. baumannii*	bau-D1	65SM01	FUEV01000003.1	**78**
type-II	*A. baumannii*	bau-D1	72SM01	FUEW01000002.1	**78**
type-II	*A. baumannii*	bau-D1	2MG	FUEX01000003.1	**78**
type-II	*A. baumannii*	bau-D1	PR308	NGBY01000012.1	**78**
type-II	*A. baumannii*	bau-D1	PR355	NGCI01000010.1	**78**
type-II	*A. baumannii*	bau-D1	PR371	NGCY01000013.1	**78**
type-II	*A. baumannii*	bau-D1	PR385	NGES01000050.1	**78**
type-II	*A. baumannii*	bau-D1	ABBL030	LLDK01000155.1	**78**
type-II	*A. baumannii*	bau-D1	ABUH393	MSMS01000084.1	**78**

type−II	*A. baumannii*	bau−D1	5MO	FUEY01000001.1	**78**
type−II	*A. baumannii*	bau−D1	UH19608	AYFZ01000199.1	**79**
type−II	*A. baumannii*	bau−D1	ABBL006	LLCM01000053.1	**79**
type−II	*A. baumannii*	bau−D1	ABBL017	LLCX01000017.1	**79**
type−II	*A. baumannii*	bau−D1	ABBL023	LLDD01000202.1	**79**
type−II	*A. baumannii*	bau−D1	ABBL039	LLDT01000063.1	**79**
type−II	*A. baumannii*	bau−D1	ABBL043	LLDX01000121.1	**79**
type−II	*A. baumannii*	bau−D1	ABBL049	LLED01000019.1	**79**
type−II	*A. baumannii*	bau−D1	ABBL052	LLEG01000062.1	**79**
type−II	*A. baumannii*	bau−D1	ABBL055	LLFA01000065.1	**79**
type−II	*A. baumannii*	bau−D1	ABBL056	LLFB01000068.1	**79**
type−II	*A. baumannii*	bau−D1	ABBL067a	LLFN01000267.1	**79**
type−II	*A. baumannii*	bau−D1	ABBL067b	LLFO01000279.1	**79**
type−II	*A. baumannii*	bau−D1	ABBL067j	LLFV01000130.1	**79**
type−II	*A. baumannii*	bau−D1	ABBL067k	LLFW01000040.1	**79**
type−II	*A. baumannii*	bau−D1	ABBL067l	LLFX01000045.1	**79**
type−II	*A. baumannii*	bau−D1	ABBL068	LLFY01000087.1	**79**
type−II	*A. baumannii*	bau−D1	ABBL069	LLFZ01000171.1	**79**
type−II	*A. baumannii*	bau−D1	ABBL101	LLHB01000147.1	**79**
type−II	*A. baumannii*	bau−D1	ABOB01	LLIQ01000018.1	**79**
type−II	*A. baumannii*	bau−D1	ABOB03	LLIS01000428.1	**79**
type−II	*A. baumannii*	bau−D1	ABOB04	LLIT01000118.1	**79**
type−II	*A. baumannii*	bau−D1	ABOB04_a	LLIU01000358.1	**79**
type−II	*A. baumannii*	bau−D1	ABOB06	LLIW01000299.1	**79**
type−II	*A. baumannii*	bau−D1	ABOB08	LLIZ01000032.1	**79**
type−II	*A. baumannii*	bau−D1	ABOB12	LLJD01000055.1	**79**
type−II	*A. baumannii*	bau−D1	ABOBEN	LLJG01000034.1	**79**
type−II	*A. baumannii*	bau−D1	Ab28	LMBQ01000001.1	**79**
type−II	*A. baumannii*	bau−D1	CU032113	LXYW01000010.1	**79**
type−II	*A. baumannii*	bau−D1	ABUH564	MSOI01000045.1	**79**
type−II	*A. baumannii*	bau−D1	499	MWPK01000002.1	**79**
type−II	*A. baumannii*	bau−D1	923	MWPO01000003.1	**79**
type−II	*A. baumannii*	bau−D1	ABUH505	NCXV01000092.1	**79**
type−II	*A. baumannii*	bau−D1	ABUH428	NCYV01000132.1	**79**
type−II	*A. baumannii*	bau−D1	ARLG−1775	NEQF01000006.1	**79**
type−II	*A. baumannii*	bau−D1	ARLG−1860	NEQK01000117.1	**79**
type−II	*A. baumannii*	bau−D1	AF−401	NZ_CP018254.1	**79**
type−II	*A. baumannii*	bau−D1	UH106_440	JWUZ03000197.1	**79**
type−II	*A. baumannii*	bau−D1	UH106_429	JWVB03000032.1	**79**
type−II	*A. baumannii*	bau−D1	UH535_423	JWVC03000014.1	**79**
type−II	*A. baumannii*	bau−D1	ABBL035	LLDP01000111.1	**79**
type−II	*A. baumannii*	bau−D1	ABBL037	LLDR01000050.1	**79**
type−II	*A. baumannii*	bau−D1	ABBL050	LLEE01000153.1	**79**
type−II	*A. baumannii*	bau−D1	ABBL053	LLEH01000059.1	**79**
type−II	*A. baumannii*	bau−D1	ABBL066	LLFL01000048.1	**79**
type−II	*A. baumannii*	bau−D1	ABBL067	LLFM01000081.1	**79**
type−II	*A. baumannii*	bau−D1	ABBL067c	LLFP01000234.1	**79**
type−II	*A. baumannii*	bau−D1	ABBL067e	LLFQ01000141.1	**79**
type−II	*A. baumannii*	bau−D1	ABBL067f	LLFR01000205.1	**79**
type−II	*A. baumannii*	bau−D1	ABBL067g	LLFS01000014.1	**79**
type−II	*A. baumannii*	bau−D1	ABBL067h	LLFT01000096.1	**79**
type−II	*A. baumannii*	bau−D1	ABBL067i	LLFU01000254.1	**79**
type−II	*A. baumannii*	bau−D1	ABBL079	LLGJ01000170.1	**79**
type−II	*A. baumannii*	bau−D1	ABBL083	LLGM01000149.1	**79**
type−II	*A. baumannii*	bau−D1	ABBL105	LLHE01000176.1	**79**
type−II	*A. baumannii*	bau−D1	ABBL106	LLHF01000121.1	**79**
type−II	*A. baumannii*	bau−D1	ABBL117	LLHO01000219.1	**79**
type−II	*A. baumannii*	bau−D1	ABBL118	LLHP01000616.1	**79**
type−II	*A. baumannii*	bau−D1	ABBL122	LLHS01000277.1	**79**
type−II	*A. baumannii*	bau−D1	ABBL127	LLHX01000342.1	**79**
type−II	*A. baumannii*	bau−D1	ABOB02	LLIR01000425.1	**79**
type−II	*A. baumannii*	bau−D1	ABOB04_b	LLIV01000188.1	**79**
type−II	*A. baumannii*	bau−D1	ABOB06_a	LLIX01000047.1	**79**
type−II	*A. baumannii*	bau−D1	ABOB07	LLIY01000105.1	**79**
type−II	*A. baumannii*	bau−D1	ABOB09	LLJA01000175.1	**79**
type−II	*A. baumannii*	bau−D1	ABOB10	LLJB01000197.1	**79**
type−II	*A. baumannii*	bau−D1	Ab23	LMBO01000002.1	**79**
type−II	*A. baumannii*	bau−D1	ORABAshR	LRMG01000196.1	**79**
type−II	*A. baumannii*	bau−D1	ORABRicT	LRMH01000055.1	**79**

type-II	*A. baumannii*	bau-D1	LIM891	LUUZ01000291.1	**79**
type-II	*A. baumannii*	bau-D1	LIM994	LUVA01000276.1	**79**
type-II	*A. baumannii*	bau-D1	LIM929	LUVB01000368.1	**79**
type-II	*A. baumannii*	bau-D1	LIM997	LUVC01000003.1	**79**
type-II	*A. baumannii*	bau-D1	LIM939	LUVD01000521.1	**79**
type-II	*A. baumannii*	bau-D1	LIM1013	LUVE01001894.1	**79**
type-II	*A. baumannii*	bau-D1	LIM1011	LUVF01002386.1	**79**
type-II	*A. baumannii*	bau-D1	LIM1049	LUVG01000004.1	**79**
type-II	*A. baumannii*	bau-D1	LIM1126	LUVH01000004.1	**79**
type-II	*A. baumannii*	bau-D1	LIM1099	LUVI01000006.1	**79**
type-II	*A. baumannii*	bau-D1	LIM1018	LUVJ01001272.1	**79**
type-II	*A. baumannii*	bau-D1	LIM1029	LUVK01000007.1	**79**
type-II	*A. baumannii*	bau-D1	LIM1128	LUVL01000225.1	**79**
type-II	*A. baumannii*	bau-D1	LIM941	MANL01000869.1	**79**
type-II	*A. baumannii*	bau-D1	AR_0036	MPBY01000002.1	**79**
type-II	*A. baumannii*	bau-D1	ABUH642	MSNJ01000071.1	**79**
type-II	*A. baumannii*	bau-D1	ABUH516	MSPM01000075.1	**79**
type-II	*A. baumannii*	bau-D1	502	MWPL01000003.1	**79**
type-II	*A. baumannii*	bau-D1	891	MWPM01000003.1	**79**
type-II	*A. baumannii*	bau-D1	895	MWPN01000006.1	**79**
type-II	*A. baumannii*	bau-D1	991	MWPP01000005.1	**79**
type-II	*A. baumannii*	bau-D1	ABUH502	NCXU01000046.1	**79**
type-II	*A. baumannii*	bau-D1	ABUH363	NCZR01000137.1	**79**
type-II	*A. baumannii*	bau-D1	PR323	NGDN01000081.1	**79**
type-II	*A. baumannii*	bau-D1	ARLG1858	NGGD01000064.1	**79**
type-II	*A. baumannii*	bau-D1	ARLG1864	NGIV01000047.1	**79**
type-II	*A. baumannii*	bau-D1	AB030	NZ_CP009257.1	**79**
type-II	*A. baumannii*	bau-D1	AbH120-A2	NZ_CP009534.1	**79**
type-II	*A. baumannii*	bau-D1	11598	MSCZ01000020.1	**156**
type-II	*A. baumannii*	bau-D1	4113	MSDA01000030.1	**156**
type-II	*A. baumannii*	bau-D1	11547	MSDB01000016.1	**156**
type-II	*A. baumannii*	bau-D1	11502	MSCX01000013.1	**156**
type-II	*A. baumannii*	bau-D1	11536	MSCY01000013.1	**156**
type-II	*A. baumannii*	bau-D1	11551	MSDC01000013.1	**156**
type-II	*A. baumannii*	bau-D1	11606	MSDD01000011.1	**156**
type-II	*A. baumannii*	bau-D1	TG02011	ASES01000026.1	**422**
type-II	*A. baumannii*	bau-D1	UH7607	AYFH01000099.1	**422**
type-II	*A. baumannii*	bau-D1	UH6907	AYFJ01000032.1	**422**
type-II	*A. baumannii*	bau-D1	UH22908	AYFV01000030.1	**422**
type-II	*A. baumannii*	bau-D1	UH16208	AYGB01000074.1	**422**
type-II	*A. baumannii*	bau-D1	UH12208	AYGK01000073.1	**422**
type-II	*A. baumannii*	bau-D1	3207	NZ_CP015364.1	**422**
type-II	*A. baumannii*	bau-D1	AB_1583-8	AMHC01000214.1	**422**
type-II	*A. baumannii*	bau-D1	AB_TG27343	AMIS01000029.1	**422**
type-II	*A. baumannii*	bau-D1	UH7907	AYFE01000114.1	**422**
type-II	*A. baumannii*	bau-D1	A33405	JPXZ01000025.1	**422**
type-II	*A. baumannii*	bau-D1	1412924	JFEF01000001.1	**510**
type-II	*A. baumannii*	bau-D1	342950	JEWZ01000078.1	**512**
type-II	*A. baumannii*	bau-D1	1295549	JFXB01000001.1	**652**
type-II	*A. baumannii*	bau-D1	277047	JFXS01000001.1	**652**
type-II	*A. baumannii*	bau-D1	426863	JFYF01000001.1	**652**
type-II	*A. baumannii*	bau-D1	CCBH15815 Y_1	NLCO01000001.1	**730**
type-II	*A. baumannii*	bau-D3	ARL1814	NGGY01000078.1	**32**
type-II	*A. baumannii*	bau-D3	1525283	JEXR0100002/64.1	**32**
type-II	*A. baumannii*	bau-D3	781407	JEZS01000001.1	**32**
type-II	*A. baumannii*	bau-D5	NIPH 67	APRA01000004.1	**35**
type-II	*A. baumannii*	bau-D6	1035119	JEVY01000001.1	**536**
type-II	*A. baumannii*	bau-D6	8D1	MABV01000021.1	**512**
type-II	*A. baumannii*	bau-D6	O1D3-2	NIWQ01000001.1	**240**
type-II	*A. baumannii*	bau-D6	86II/2C	NIWI01000010.1	**690**
type-II	*A. baumannii*	bau-D6	PLG9P835	NIWR01000002.1	**1179**
type-II	*A. baumannii*	bau-D7	M3AC9-7	JTEC01000010.1	**250**
type-II	*A. baumannii*	bau-D7	UH392_659	JWTR03000141	**250**
type-II	*A. baumannii*	bau-D7	UH592_595	JZJE02000126	**250**
type-II	*A. baumannii*	bau-D7	ABBL149	LLIP01000073.1	**250**
type-II	*A. baumannii*	bau-D7	UH592_599	JZJD02000204.1	**250**
type-II	*A. baumannii*	bau-D7	ABBL122	LLHS01000277.1	**250**
type-II	*A. baumannii*	bau-D7	CCF12	LZAF01000035.1	**250**
type-II	*A. baumannii*	bau-D7	CCF50	LZBF01000052.1	**250**

type–II	*A. baumannii*	bau–D7	ABUH797	LZUU01000019.1	**250**
type–II	*A. baumannii*	bau–D7	ABUH399	MSMU01000040.1	**250**
type–II	*A. baumannii*	bau–D7	ABUH675	MSNS01000001.1	**250**
type–II	*A. baumannii*	bau–D7	ABUH631	NCZW01000042.1	**250**
type–II	*A. baumannii*	bau–D7	ABUH638	NCZZ01000005.1	**250**
type–II	*A. baumannii*	bau–D7	ABUH650	NDAD01000033.1	**250**
type–II	*A. baumannii*	bau–D7	ABUH658	NDAF01000140.1	**250**
type–II	*A. baumannii*	bau–D7	ABUH619	NDAY01000053.1	**250**
type–II	*A. baumannii*	bau–D7	ARLG1898	NGHX01000065.1	**250**
type–II	*A. baumannii*	bau–D7	ARLG1906	NGID01000040.1	**250**
type–II	*A. baumannii*	bau–D7	ARLG1891	NGJI01000045.1	**250**
type–II	*A. baumannii*	bau–D7	247	NSJA01000052.1	**286**
type–II	*A. baumannii*	bau–D7	XH681	LYJI01000001.1	**879**
type–II	*A. baumannii*	bau–D9	TG27387	ASGJ01000040.1	**6**
type–II	*A. baumannii*	bau–D9	ABBL011	LLCR01000058.1	**6**
type–II	*A. baumannii*	bau–D9	XH730	LYHV01000013.1	**46**
type–II	*A. baumannii*	bau–D9	XH674	LYJP01000037.1	**46**
type–II	*A. baumannii*	bau–D9	XH673	LYJQ01000034.1	**46**
type–II	*A. baumannii*	bau–D9	XH668	LYJV01000010.1	**46**
type–II	*A. baumannii*	bau–D9	XH664	LYJZ01000001.1	**46**
type–II	*A. baumannii*	bau–D9	XH662	LYKB01000388.1	**46**
type–II	*A. baumannii*	bau–D9	XH661	LYKC01000040.1	**46**
type–II	*A. baumannii*	bau–D9	XH648	LYKH01000001.1	**46**
type–II	*A. baumannii*	bau–D9	XH647	LYKI01000023.1	**46**
type–II	*A. baumannii*	bau–D9	XH642	LYKN01000001.1	**46**
type–II	*A. baumannii*	bau–D9	XH641	LYKO01000001.1	**46**
type–II	*A. baumannii*	bau–D9	XH553	LYKU01000207.1	**46**
type–II	*A. baumannii*	bau–D9	XH545	LYLC01000001.1	**46**
type–II	*A. baumannii*	bau–D9	XH512	LYLG01000001	**46**
type–II	*A. baumannii*	bau–D9	AE3M	FWFB01000092.1	**85**
type–II	*A. baumannii*	bau–D9	AE27M	FWWO01000046.1	**85**
type–II	*A. baumannii*	bau–D9	AC40M	FWYN01000006.1	**85**
type–II	*A. baumannii*	bau–D9	ACMH–6200	LKMA01000064.1	**85**
type–II	*A. baumannii*	bau–D9	ACMH–6201	LKMB01000010.1	**85**
type–II	*A. baumannii*	bau–D9	AB–A	LWSM01000068.1	**85**
type–II	*A. baumannii*	bau–D9	AB–B	LWSN01000050.1	**85**
type–II	*A. baumannii*	bau–D9	AB–C	LWSO01000056.1	**85**
type–II	*A. baumannii*	bau–D9	AR_0037	MPBX01000005.1	**85**
type–II	*A. baumannii*	bau–D9	AR_0033	MPCA01000001.1	**85**
type–II	*A. baumannii*	bau–D9	MBL_M4	MWTU01000196.1	**85**
type–II	*A. baumannii*	bau–D9	MBL_M6	MWTW01000060.1	**85**
type–II	*A. baumannii*	bau–D9	B11911	LFYX01000003.1	**149**
type–II	*A. baumannii*	bau–D9	PR361	NGCO01000049.1	**464**
type–II	*A. baumannii*	bau–D9	6200	NZ_CP010397.1	**464**
type–II	*A. baumannii*	bau–D9	1297549	JEVV01000001.1	**528**
type–II	*A. baumannii*	bau–D9	ARLG1326	NGHQ01000027.1	**973**
type–II	*A. baumannii*	bau–D9	ABBL067	LLFM01000081.1	**nd**
type–II	*A. baumannii*	bau–D9	AB360	NGKM01000001.1	**622**
type–II	*A. baumannii*	bau–D9	IOMTU433	NZ_AP014649.1	**622**
type–II	*A. baumannii*	bau–D9	XH729	LYHW01000042.1	**nd**
type–II	*A. baumannii*	bau–D9	XH703	LYIS01000022.1	**nd**
type–II	*A. baumannii*	bau–D9	SP1917	LFYW01000006.1	**nd**
type–II	*A. baumannii*	bau–D10	AB900	ABXK01000016.1	**49**
type–II	*A. baumannii*	bau–D10	OIFC111	AMFY01000003.1	**49**
type–II	*A. baumannii*	bau–D10	1461963	JEWQ01000007.1	**49**
type–II	*A. baumannii*	bau–D10	1293320	JFEE01000003.1	**49**
type–II	*A. baumannii*	bau–D10	ABBL070	LLGA01000031.1	**49**
type–II	*A. baumannii*	bau–D10	ABBL071	LLGB01000025.1	**49**
type–II	*A. baumannii*	bau–D11	TG22198	ASFT01000014.1	**164**
type–II	*A. baumannii*	bau–D11	AB_2007–16–25–01–7	AMHI01000047.1	**241**
type–II	*A. baumannii*	bau–D11	AB_2007–16–27–01	AMHJ01000047.1	**241**
type–II	*A. baumannii*	bau–D11	AB_TG27339	AMIR01000013.1	**241**
type–II	*A. baumannii*	bau–D11	118362	JEWB01000070.1	**241**
type–II	*A. baumannii*	bau–D12	UMB002	AEPL01000028.1	**16**
type–II	*A. baumannii*	bau–D12	268680	JEYN01000009.1	**16**
type–II	*A. baumannii*	bau–D12	1043794	JEYX01000007.1	**16**
type–II	*A. baumannii*	bau–D12	972082	JFAA01000074.1	**16**
type–II	*A. baumannii*	bau–D12	655378	JFCE02000001.1	**16**
type–II	*A. baumannii*	bau–D12	1064293_45	JFDS01000016.1	**16**

type-II	*A. baumannii*	bau-D12	FDAARGOS_123	LORJ01000003	**16**
type-II	*A. baumannii*	bau-D12	232184	JEYI01000006	**16**
type-II	*A. baumannii*	bau-D12	J65	LAKP01000059.1	**16**
type-II	*A. baumannii*	bau-D12	BAL 255	CZWB01000012.1	**16**
type-II	*A. baumannii*	bau-D12	SIUA6 17	NIUK01000031.1	**16**
type-II	*A. baumannii*	bau-D12	NIPH 601	APQZ01000007.1	**40**
type-II	*A. baumannii*	bau-D12	GTC 03322	BBNF10000040.1	**40**
type-II	*A. baumannii*	bau-D12	GTC 03323	BBNG01000002.1	**40**
type-II	*A. baumannii*	bau-D12	GTC 03328	BBNI01000006.1	**40**
type-II	*A. baumannii*	bau-D12	NBRC 110489	BBOP01000045.1	**40**
type-II	*A. baumannii*	bau-D12	NBRC 110491	BBTC01000006.1	**40**
type-II	*A. baumannii*	bau-D12	A118	AEOW01001006.1	**404**
type-II	*A. baumannii*	bau-D12	ZW85-1	NC_023028.1	**639**
type-II	*A. baumannii*	bau-D13	NIPH 60	APPM01000011.1	**34**
type-II	*A. baumannii*	bau-D13	NBRC 110492	BBTD01000008.1	**34**
type-II	*A. baumannii*	bau-D13	NIPH 201	APQV01000007.1	**38**
type-II	*A. baumannii*	bau-D13	DSM 30011	JJOC01000003.1	**738**
type-II	*A. baumannii*	bau-D13	Naval-72	AMFI01000006.1	**405**
type-II	*A. baumannii*	bau-D14	1267820	JEWD01000063.1	**103**
type-II	*A. baumannii*	bau-D14	AB031	NZ_CP009256.1	**638**
type-II	*A. baumannii*	bau-D15	AB3638 LV38	LRDW01000016.1	**25**
type-II	*A. baumannii*	bau-D15	4190	AEPA01000264.1	**25**
type-II	*A. baumannii*	bau-D15	UMB003	AEPM01000002.1	**25**
type-II	*A. baumannii*	bau-D15	Naval-18	AFDA02000003	**25**
type-II	*A. baumannii*	bau-D15	OIFC143	AFDL01000004	**25**
type-II	*A. baumannii*	bau-D15	AB5256	AHAI01000041.1	**25**
type-II	*A. baumannii*	bau-D15	NIPH146	APOU01000021.1	**25**
type-II	*A. baumannii*	bau-D15	CI86	AVOB01000072.1	**25**
type-II	*A. baumannii*	bau-D15	CI79	AVOD01000049.1	**25**
type-II	*A. baumannii*	bau-D15	107m	CBSG010000032.1	**25**
type-II	*A. baumannii*	bau-D15	984213	JEVX01000013.1	**25**
type-II	*A. baumannii*	bau-D15	1429530	JEWM01000001.1	**25**
type-II	*A. baumannii*	bau-D15	RUH1486	JZBU01000024.1	**25**
type-II	*A. baumannii*	bau-D15	NM3	JZBV01000044.1	**25**
type-II	*A. baumannii*	bau-D15	LUH_6220	JZBW01000052.1	**25**
type-II	*A. baumannii*	bau-D15	4390	JZBY01000157.1	**25**
type-II	*A. baumannii*	bau-D15	741019	JZBZ01000009.1	**25**
type-II	*A. baumannii*	bau-D15	161/07	JZCA01000061.1	**25**
type-II	*A. baumannii*	bau-D15	ABBL018	LLCY01000148.1	**25**
type-II	*A. baumannii*	bau-D15	AB2828 LV35	LRDT01000061.1	**25**
type-II	*A. baumannii*	bau-D15	AB3806 LV40	LRDY01000030.1	**25**
type-II	*A. baumannii*	bau-D15	PR388	NGEV01000035.1	**25**
type-II	*A. baumannii*	bau-D15	ARLG1317	NGHO01000017.1	**25**
type-II	*A. baumannii*	bau-D15	NIPH146	KB849308.1	**25**
type-II	*A. baumannii*	bau-D15	HWBA8	NZ_CP020597	**25**
type-II	*A. baumannii*	bau-D15	AB_2008-15-69	AMHN01000069.1	**25**
type-II	*A. baumannii*	bau-D15	R2091	NZ_LN997846.1	**126**
type-II	*A. baumannii*	bau-D15	P630	CBYG010000034.1	**126**
type-II	*A. baumannii*	bau-D15	233846	JMOG01000008.1	**126**
type-II	*A. baumannii*	bau-D15	PR337	NGEA01000014.1	**126**
type-II	*A. baumannii*	bau-D15	CIP70.10	NZ_LN865143.1	**126**
type-II	*A. baumannii*	bau-D15	LUH_7841	JZBX01000006.1	**402**
type-II	*A. baumannii*	bau-D16	NIPH 615	APOV01000028.1	**12**
type-II	*A. baumannii*	bau-D17	AB967	LRDS01000005	**48**
type-II	*A. baumannii*	bau-D17	ABUH397	NCYO01000022.1	**48**
type-II	*A. baumannii*	bau-D17	PR356	NGCJ01000033.1	**48**
type-II	*A. baumannii*	bau-D17	PR343	NGEE01000017.1	**48**
type-II	*A. baumannii*	bau-D17	ARLG1830	NGHE01000010.1	**48**
type-II	*A. baumannii*	bau-D17	1598530	JMOE01000001.1	**56**
type-II	*A. baumannii*	bau-D17	OIFC035	AMTB01000006.1	**403**
type-II	*A. baumannii*	bau-D17	Ab5	LANH01000043	**nd**
type-II	*A. baumannii*	bau-D19	1062314	JFDR01000001.1	**54**
type-II	*A. baumannii*	bau-D19	99063	JEXJ01000070	**506**
type-II	*A. baumannii*	bau-D20	299505	JEWY01000048	**508**
type-II	*A. baumannii*	bau-D21	219_ABAU	JVPN01000006.1	**1140**
type-II	*A. baumannii*	bau-D21	ABBL110	LLHI01000268.1	**nd**
type-II	*A. baumannii*	bau-D21	LIM1820	LUUY01000276.1	**107**
type-II	*A. baumannii*	bau-D2	SDF	CU468230	**17**
type-II	*A.nosocomialis*	nos-D2	Ac. sp.1424608	JFXL01000001.1	**279**

type-II	*A.nosocomialis*	nos-D2	Ac.sp. 21871	JEWV01000004.1	**279**
type-II	*A.nosocomialis*	nos-D2	Ac. sp. 25977_8	JFVJ01000007.1	**501**
type-II	*A.nosocomialis*	nos-D2	Ac. sp. 25977_7	JFVK01000003.1	**501**
type-II	*A.nosocomialis*	nos-D2	Ac. sp. 25977_6	JFVL01000006.1	**501**
type-II	*A.nosocomialis*	nos-D2	Ac. sp. 25977_4	JFVM01000009.1	**501**
type-II	*A.nosocomialis*	nos-D2	Ac. sp. 25977_3	JFVN01000007.1	**501**
type-II	*A.nosocomialis*	nos-D2	Ac. sp. 25977_2	JFVO01000008.1	**501**
type-II	*A.nosocomialis*	nos-D2	Ac. sp. 25977_1	JFVP01000009.1	**501**
type-II	*A.nosocomialis*	nos-D2	Ac. sp. 796380-1375	JMOS01000007.1	**501**
type-II	*A.nosocomialis*	nos-D2	Ac. sp. 25977_9	JMPF01000048.1	**501**
type-II	*A.nosocomialis*	nos-D2	Ac. sp. 25977_10	JFVQ01000046.1	**501**
type-II	*A.nosocomialis*	nos-D2	TG19596	AMIZ01000051.1	**501**
type-II	*A.nosocomialis*	nos-D2	UBA5656	DIJA01000032.1	**501**
type-II	*A.nosocomialis*	nos-D2	GTC 3312	BBOO01000030.1	**1181**
type-II	*A.nosocomialis*	nos-D2	NBRC 110500	BBOT01000029.1	**1181**
type-II	*A.nosocomialis*	nos-D2	UBA873	DBFT01000089.1	**nd**
type-II	*A.nosocomialis*	nos-D4	GTC 03313	BBTJ01000007.1	**68**
type-II	*A.nosocomialis*	nos-D4	NBRC 110501	BBTO01000005.1	**68**
type-II	*A.nosocomialis*	nos-D4	NBRC 110503	BBTP01000005.1	**68**
type-II	*A.nosocomialis*	nos-D4	NBRC 110502	BBTW01000005.1	**68**
type-II	*A.nosocomialis*	nos-D4	UBA3966	DGCY01000183.1	**68**
type-II	*A.nosocomialis*	nos-D4	Ac. sp. 1281984	JFXD01000001.1	**68**
type-II	*A.nosocomialis*	nos-D4	446_ABAU	JVGT01000060.1	**68**
type-II	*A.nosocomialis*	nos-D4	Ac. sp. FDAARGOS_131	LORV01000006.1	**68**
type-II	*A.nosocomialis*	nos-D4	XH762	LYGU01000023.1	**68**
type-II	*A.nosocomialis*	nos-D4	XH654	LYKD01000057.1	**68**
type-II	*A.nosocomialis*	nos-D4	XH653	LYKE01000040.1	**68**
type-II	*A.nosocomialis*	nos-D4	XH551	LYKW01000034.1	**68**
type-II	*A.nosocomialis*	nos-D4	ABBL080	LLGK01000451.1	**71**
type-II	*A.nosocomialis*	nos-D4	ABUH381	NDFD01000029.1	**71**
type-II	*A.nosocomialis*	nos-D4	259052	JEYJ01000008.1	**71**
type-II	*A.nosocomialis*	nos-D4	1592897	JEYK02000002.1	**71**
type-II	*A.nosocomialis*	nos-D4	28F	CBSD020000036.1	**71**
type-II	*A.nosocomialis*	nos-D4	HJ14	MADF01000001.1	**217**
type-II	*A.nosocomialis*	nos-D4	UBA1655	DCLF01000050.1	**410**
type-II	*A.nosocomialis*	nos-D4	UBA4562	DGKU01000019.1	**410**
type-II	*A.nosocomialis*	nos-D4	NIPH 386	APPP01000013.1	**410**
type-II	*A.nosocomialis*	nos-D4	1396970	JEWI01000055.1	**410**
type-II	*A.nosocomialis*	nos-D4	694762	JFEV01000024.1	**410**
type-II	*A.nosocomialis*	nos-D4	WC-487	AMZR01000082.1	**410**
type-II	*A.nosocomialis*	nos-D4	Ac. sp.1396970	ACQF02000020.1	**410**
type-II	*A.nosocomialis*	nos-D4	UBA2098	DCYY01000023.1	**1182**
type-II	*A.nosocomialis*	nos-D4	UBA2046	DDAY01000142.1	**1183**
type-II	*A.nosocomialis*	nos-D4	A. baumannii 1	JSAO01000067.1	**1184**
type-II	*A.nosocomialis*	nos-D4	UBA3105	DFAN01000186.1	**nd**
type-II	*A.nosocomialis*	nos-D4	XH796	LYGC01000024.1	**nd**
type-II	*A.nosocomialis*	nos-D4	UBA5077	DIAN01000118.1	**nd**
type-II	*A.nosocomialis*	nos-D4	UBA6700	DKJE01000061.1	**nd**
type-II	*A.nosocomialis*	nos-D8	Ac. sp. TG21145	AMJH01000037	**74**
type-II	*A.nosocomialis*	nos-D12	OIFC021	AMFR01000029.1	**224**
type-II	*A.nosocomialis*	nos-D12	766875	JEXX01000001.1	**224**
type-II	*A.nosocomialis*	nos-D18	NIPH 2119	APOP01000003.1	**76**
type-II	*A.nosocomialis*	nos-D18	LMG 10619	BBSR01000002.1	**76**
type-II	*A.nosocomialis*	nos-D18	216872	JFEM01000009.1	**530**
type-II	*A.nosocomialis*	nos-D18	BIDMC 57	JMUI01000011.1	**530**
type-II	*A.nosocomialis*	nos-D22	UBA3970	DGCU01000061	**ND**
type-II	*A.nosocomialis*	nos-D22	PR365	NGCS01000003.1	**395**
type-II	*13BJ*	nos-D22	NBRC 110496	BBTF01000002.1	**200**
type-II	*A. pittii*	pit-D6	PHEA-2	NC_016603.1	**1164**
type-II	*A. pittii*	pit-D20	Acinetobacter sp. 1542444	JEYA01000001.1	**675**
type-II	*A.baylyi*	bay-D15	Acinetobacter ADP1	NC_005966.1	**1185**

Supplementary file 2 CDI islands

a) Type–I *cdi* islands

Species	Strain	Site	Island	flanking orf	*cdi* B	*cdi* A	*cdi* I					flanking orf
A. baumannii	WC-348	A site	**bau-A1**	1316	1317	1318-19	1320	1321	1322			1323
A. baumannii	BJAB0715	A site	**bau-A2**	1274	1275	1276-78	-	1282	1283			1284
A. pittii	IEC338	A site	**pit-A3**	7795	7800	7805	7810	7815	7820			7825
A. pittii	PR320	A site	**pit-A4**	-	19125	19130	19135					19140
A. pittii	ABUH540	A site	**pit-A5**	13600	13605	13610	13615					13620
A. baumannii	ACICU (reference genome)	A site		1137	empty site							1138
A. baumannii	NCTC7364	B site	**bau-B4**	1427	1428	1429	1430	1431	1432			1434
A. pittii	ABBL098	B site	**pit-B7**	-	11410	11405	11415	11420	11425			11430
A. baumannii	NIPH146	B site	**bau-B1**	1824	1825	1826	1827					1828
A. pittii	ATCC1904	B site	**pit-B5**	2275	2270	2265	2260	2255				2250
A. baumannii	19606	B site	**bau-B2**	2522	2521	2520	2519	2518				2517
A. pittii	IEC338	B site	**pit-B6**	8930	8935	8940	8945	8950				8965
A. baumannii	AB031	B site	**bau-B3**	8525	8530	8535	8540	8545	8550	TPase		
A. baumannii	ACICU (reference genome)	B site		1272	alternative genomic island							1277
A. baumannii	ab736	Cd site	**bau-C1**	8290	8295	8300	8305					8310
A. baumannii	ACICU	Cd site	**bau-C2**	1913	1912	1911	1910					1908
A. baumannii	ZW85-1	Cd site	**bau-C3**	7825	7830-35	7840	7845					7850
A. nosocomialis	A. sp. FDAARGOS_131	Cd site	**nos-C8**	14385	14390	14395	14400					14405
A. nosocomialis	SSA3	Cd site	**nos-C9**	8550	8545	8540	8535					8530
A. pittii	ARLG1803	Cu site	**pit-C6**	18850	18855	18860	18865	18870	18875	18880	18885	18890
A. pittii	PHEA-2	Cu site	**pit-C7**	1272	1269	1268	1267	1266	1265	1264	1263	1262
A. calcoaceticus	ANCC3680	Cu site	**cal-C10**	2410	2411	2412	2413	2414	2415	2416	2417	2418
A. baumannii	UH19608	Cu site	**bau-C4**	833	834	835	836	838-37	840	-	-	839
A. baumannii	ACICU (reference genome)	Cu site		1891	empty site							1890

b) Type–II *cdi* islands

Species	Strain	Site	flanking orf	*cdi* B	*cdi* A	*cdi* I	flanking orfs			
A. baumannii	ACICU (reference genome)	D site	2441	empty site			2440	2439	2438	2437

c) Capture of flanking DNA

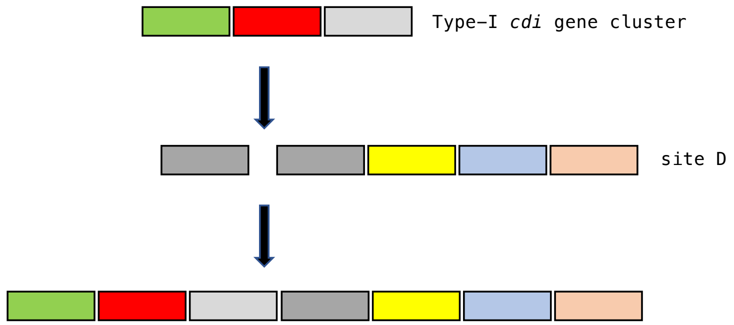

The excision of type-I *cdi* gene clusters inserted at D site is accompanied by the capture of flanking sequences (see type-I *cdi* islands at the Cu site in panel b)

d) Target site duplications (TSD) induced by type–II *cdi* islands

```
TATTGAAATA AAAAAGCTCCACAAA-AGGAGCTTTT target                              TTAGAATCTT
TATTGAAAT- AAAAAGCTCCACAAA-AGGAGCTTTT bau-D1 AAAAAGCTCCCGTAACAGGAGCTTTT TT-GAATCTT
TATTGAAAT- AAAAAGCTCCACAAA-AGGAGCTTTT bau-D9 AAAAAGCTCCATAAA-AGGAGCTTTT TTAGAATCTT
TATCGAAATA AAAAAGCTCCACAAA-AGGAGCTTTT bau-D3 AAAAAGCTCCCGTAACAGGAGCTTTT TT-GAATCTT
TATTGAAAT- AAAAAGCTCCACAAA-AGGAGCTTTT bau-D6 AAAAAGCTCCCGTAACAGGAGCTTTT TTAGAATCTT
```

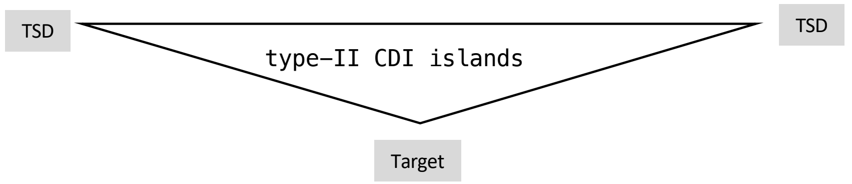

Duplications at the ends of type-II *cdi* islands found in the *A. baumannii* strains AB030 (bau-D1), IOMTU 433 (bau-D9), 781407 (bau-D6) and 1035119 (bau-D6). The empty target site is from in the *A. baumannii* strain ACICU.

Supplementary file 3. Alignment of type-I CdIB proteins

Highly and moderately homologous residues are in red and blu, respectively.

```
         1                                                                                                   100
bau-A1   MLTRNFITSS IILFAGFHQY VYAIKDVSLP SQVLQDQRLK ELNQQLQDQL AQQTPYLNTK PLQDFKHLVV EESPCVTVKE ISLIPLIGQS ESDLQQFNFV
bau-C1   MLTKNFITSS IIFFAGFHQY VYAIEDVSLP SQVLQDQRLK ELNQQLQDQL AQQTHYQNTK PLQDFKHLVV EESPCVTVKE ISLIPLIGQS ESDLKQFNFV
pit-A4   MLTKNFITSS LILFAGFHQY VYAIEDVSLP SQVLQDQRLK ELNQQLQDQL AQQTPYENTK PLQDFKHLVV EESPCVTVKE ISLIPLNGQS QSDLQQFNFV
pit-C5   MLTKNFITSS LILFAGFHQY VYAIEDVSLP SQVLQDQRLK ELNQQLQDQL AQQTPYENTK PLQDFKHLVV EESPCVTVKE ISLIPLNGQS QSDLQQFNFV
pit-C6   MLTRNFITSS IILFAGYHQY VYAIEDVSLP SQVLQDQRLK ELNQQLQDQL AQQTPYQNTK PLQDFKHLVV EESPCVAVKK ISLIPLTGHS ESDLQQFNFV
pit-A5   MLTKNFITSS IILFAGFHQY VYAIEDVSLP SQVLQDQRLK ELNQQLQDQI AQQTPYQNTK PLQDFKHLVV KESPCVVVKK ISFIPLNDQS ESELQQFNFV
bau-C3   MLTRNFITSS IILFAGFHQY VYAIEDVSLP SQVLQDQRLK ELNQQLQGQI SQQTPYQNTK PLQDFKRLVV EESPCVAVKE ITLIPLIGQS ESDLQQFNFV
cal-C10  MLTKNFITSS LILFVGFHQY VYAIEDVSLP SQVLQDQRLK ELNQQLQDQL AQQTPYQNTK PLQDFKHLVV KESPCVVVKK ISLVPLSGQS ESDLQQFNFV
bau-A2   MLTRNFITSS IIFFAGFHQY VYAIEDVSLP SQVLQDQRLK ELNQQLQDQL AQQTPYQNIK PLQDFKHLVV EESPCVTVKE ISLIPLIGQS ESDLQQFNFV
bau-C4   MLTKNFINSS IILFAGFHQY VYAIEDVSLP SQVLQDQRLK ELNQQLQDQL AQQTPYQNTK PLQDFKYLAV KESPCVAVKE ISLIPLIGQS ESDLQQFNFV
bau-C2   MLTKNFITSS IILFAGFHQY VYAIEDVSLP SQVLQDQRLK ELNQQLQDQL AQQTPYQNTK PLQDFKHLVV EESPCVTVKE ISLIPLIGQS ESDLQQFNFV
pit-C7   MLTKSFITTS IIFFTGFNQY VYAIEDVSLP SQVLQDQRLK ELNQQLQDQL AQQTPYQNTK PLQDFKHLVV DESPCVAVKE ISLIPLIGQS ESDLQRFNFV
nos-C8   MLNKNFITFS IIFFAGFNQY VYAIEDVSLP GQVLQEQRLK ELSQQLQDQL TQQTPYQNSK SSQDFKHLVV EESPCVAVKK ISLLPLKDQS VADLKQFDFV
pit-A3   MLTRNFITSS IILFAGFHQY VYAIEDVSLP SQVLQDQRLK ELNQQIQDQL AQQTPYQNNK PLQDFKHLVV DESPCVAVKK ISLIPLNDQS E--LQQFNFV
pit-B6   MLTRNFITSS IILFAGFHQY VYAIEDVSLP SQVLQDQRLK ELNQQIQDQL AQQTPYQNNK PLQDFKHLVV DESPCVAVKK ISLIPLNDQS E--LQQFNFV
cal-C11  MLAKSFITTS IIFFTGFNQY VYAIEDVSLP SQVLQEQRLK ELNQQLQDQL AQQTPYQNTK PLQDFKHLVV KESPCVAVKK IILVPLNGQS ESDLQQFNFV
nos-C9   MLTKKFINTS IIFFAGFNQY VYAIEDVSLP GQVLQEQRFK ELSQQLQDQF TQQTPYQNTK PLQDFKHLIV EESPCVAVKK ISLIPLKEQS VTDLQQFDFI
bau-B1   MQNKIFFTHA FFLLYGLATH AYAIDDISLP DRIRQEQRLK ELNQQLEAQI TQLPPKPDTT QLQDFKTIVV PEEPCVQVKN INFDSKTLQ- DPEYYKFHFM
bau-B4   MQNKIFFTHA FFLLYGLATH AYAIDDISLP DRIRQEQRLK ELNQQLEAQI TQLPPKPDTT QLQDFKTIVV PEEPCVQVKN INFDSKTLQ- DPEYYKFHFM
bau-B2   MQNKIFFTHA FFLLYGLATH AYAIDDISLP DRIRQEQRLK ELNQQLEAQI TQLPPKPVTT QLQDFKTIVV PEEPCVQVKN INFDSKTLQ- DPEYYKFHFM
bau-B3   MQNKIFFTHA FFLLYGLATH AYAIDDISLP DRIRQEQRLK ELNQQLEAQI TQLPPKPVTT QLQDFKTIVV PEEPCVQVKN INFDSKTLQ- DPEYYKFHFM
pit-B5   MQNKIFFTHA FFLLYGLATH AYAIDDISLP DRIRQEQRLK ELNQQLEEQI TQLVPKPVTT QLQDFKTIVV SEEPCVKVKN IDFDRKTLQ- DPEHYKFHFM
pit-B7   MQNKIFITHA FFLFYGLATH AYAIDDISLP DRIRQEQRLK ELNQQLEDQI TQLAPKPVDT KVQDFRTIVV AEEPCFKVQN IDFDRKTLQ- DPEYYKFHFM

         101                                                                                                 200
bau-A1   IKAIKKHPQN ILGKCIGTQS LHNIVNYAQN ELLKKGFITS QIVVSPQDLN QGNLNLSIQI GRLNKIVIQE GKISSLQLKT GLPFKAGDIV NLKRLDQGLE
bau-C1   IKAIKKHPQD ILGKCIGTQS LHNIVNYAQN ELLKKGFITS QIVVSPQDLS QGNLNLSIQI GRLNKIVIQE GKISSLQLKT GLPFKAGDIV NLKRLDQGLE
pit-A4   VKAIKKHPQN VLGKCIGTQS LHNIVNYAQN ELLKKGFITS QIVVSPQYLN QGNLNLSVQI GRLNKIIIQE GKISSLQLKT GLPFKAGDIV NLKKLDQGLE
pit-C5   VKAIKKHPQN VLGKCIGTQS LHNIVNYAQN ELLKKGFITS QIVVSPQDLN QGNLNLSVQI GRLNKIIIQE GKISSLQLKT GLPFKAGDIV NLKKLDQGLE
pit-C6   IKAIKKHPQN ILGKCIGTQS LHNIVNYAQN ELLKKGFITS QIVVSPQDLN QGNLNLSVQI GRLNKIIIQE GKISSLQLKT GLPFKAGDIV NLKKLDQGLE
pit-A5   VKAIKKHPQN VLGKCIGTQS LHNIVNYAQN ELLKKGFITS QIVVSPQDLN QGNLNLSVQI GRLNKIVIQE GKISSLQLKT GLPFKTGDIV NLKRLDQGLE
bau-C3   VKAIKKHPQN ILDKCIGTQS LHNIVNYAQN ELLKKGFITS QIVVSPQDLN QGNLNLSVQI GRLNKIVIQE GKISSLQIKT GLPFKAGDIV NLKRLDQGLE
cal-C10  VKAIKKHPQS VLGKCIGTQS LHNIVNYAQN ELLKKGFITS QIVVSPQDLN QGNLNLSVQI GRLNKIIIQE GKISSLQLKT GLPFKAGDIV NLKRLDQGLE
bau-A2   IKAIKKHPQN ILSKCIGTQS LHNIVNYAQN ELLKKGFVTS QIVVSPQDLN QGNLNLSIQI GRLNKIVIQE GKISSLQLKT GLPFKAGDIV NLKRLDQGLE
bau-C4   VKAIKKHPQN ILGKCIGTQS LHNIVNYAQN ELLKKGFITS QIVVSPQDLN QGNLNLSVQI GRLNKIIIQE GKISSLQLKT GLPFKAGDIV NLKRLDQGLE
bau-C2   IKAIKKHPQN ILGKCIGTQS LHNIVNYAQN ELLKKGFITS QIVVSPQDLN HGNLNLSIQI -RLNKIVIQE GKISSLQLKT GLPFKAGDIV NLKRLDQGLE
pit-C7   IKAIKKHPQN ILGKCIGTQS LHNIVNYAQN ELLKKGFITS QIVVSPQDLN QGNLNLSVQI GRLNKIIIQE GKISSLQLKT GLPFKASDIV NLKKLDQGLE
nos-C8   VKAIQKHPQV VLGKCIGTQS LHNIVNYAQN ELLKKGFITS QIVVSPQDLN QGNLYLSVQI GRLNKVVIQE GKISSLQLKT GLPFKAGDII NLKKLDQGLE
pit-A3   VKAIKEHPQN VLGKCIGTQS LHNIVNYAQN ELLKKGFITS QIVVSPQDLN QGNLNLSVQI GRLNKIIIQE GKISSLQLKT GLSFKAGDIV NLKSLDQGLE
pit-B6   VKAIKEHPQN VLGKCIGTQS LHNIVNYAQN ELLKKGFITS QIVVSPQDLN QGNLNLSVQI GRLNKIIIQE GKISSLQLKT GLSFKAGDIV NLKSLDQGLE
cal-C11  VKAIKKHPQN ILGKCIGTQS LHNIVNFAQN ELLKKGFITS QIVVSPQDLN QGNLNLSVQV GRLNKIIIQE GEISSLQLKA GLPFKAGDIV NLKRLDQGLE
nos-C9   VKAIQKHPQA VLGKCIGTQS LHNIVNYAQN ELLKKGFITS QIVVNPQDLN QGNLYLSVQI GRLNKVVIQE GKISSLQLKT GLPFKTGDII NLKKLDQGLE
bau-B1   LRAIWNHPQK IIGKCIGTQS LHNLVNFAQN ELIKKGFITS QVTVDPQDIQ QGSLRLGVQI GRLRKIIVEG EQLSLLQLKA ALPFKEGDVL NLQQLDQGLE
bau-B4   LRAIWNHPQK IIGKCIGTQS LHNLVNFAQN ELIKKGFITS QVTVDPQDIQ QGSLRLGVQI GRLRKIIVEG EQLSLLQLKA ALPFKEGDVL NLQQLDQGLE
bau-B2   LRAIWNHPQK IIGKCIGTQS LHNLVNFAQN ELIKKGFITS QVTVDPQDIQ QGSLRLGVQI GRLRKIIVEG EQLSLLQLKA ALPFKEGDVL NLQQLDQGLE
bau-B3   LRAIWNHPQK IIGKCIGTQS LHNLVNFAQN ELIKKGFITS QVTVDPQDIQ QGSLRLGVQI GRLRKIIVEG EQLSLLQLKA ALPFKEGDVL NLQQLDQGLE
pit-B5   LRAIWNHPQK IIGKCIGTQS LRNLVNFAQN ELIKKGFITS QVTVAPQDIQ QGSLRLGVQI GRLRKIIVEG EQLSLLQLKA SLPFKEGDVL NLQQLDQGLE
pit-B7   LRAIWKHPQK IIGKCIGTKS LHNLVNFAQN ELIKKGFITS QVTVDPQDIQ QGSLRLGVQI GRLHKIIVQG EQLSLLQLKA ALPFKEGDVL NLKQLDQGLE

         201                                                                                                 300
bau-A1   NLKRVYAVDM QITPATAQDK ELTGYSDLIL KLQALQKVNF NLSVDDSGNQ DTGTYMGNIG LGINNPFHLN DILSLNVSHS LDNFHEDLNR SYFISYQLPV
bau-C1   NLKRVYAVDM QITPATAQDK ELTGYSDLIL KLQALQKVNF NLSVDDSGNQ DTGTYMGNIG IGINNPFHLN DILSLNVSHS LDNFHEDLNR SYFISYQLPV
pit-A4   NLKRVYAVDM QITPASAQDK ELTGYSDLIL KLQPLQKFNF NLSVDDSGNQ DTGTYMGNIG IGVNNPFHLN DILSLNVSHS LDDFNEDLNR SYFISYQLPV
pit-C5   NLKRVYAVDM QITPASAQDK ELTGYSDLIL KLQPLQKFNF NLSVDDSGNQ DTGTYMGNIG IGVNNPFHLN DILSLNVSHS LDDFNEDLNR SYFISYQLPV
pit-C6   NLKRVYAVDM QITPATAQDK ELTGYSDLIL KLQPLQKVNF NLSVDDSGNQ DTGTYMGNIG IGVNNPFHLN DILSLNVSHS LDDFHEDLNR SYFISYQLPV
pit-A5   NLKRAYAVDM QITPASVQDK ELTGYSDLIL KLQPLQKVNF NLSVDDSGNQ DTGTYMGNIG LGINNPFHLN DILSLNVSHS LDDFHEDLNR SYFISYQLPV
bau-C3   NLKRVYVVDM QITPATAQDK ELTGHSDLIL KLQPLQKVNF NLSVDDSGNQ DTGIHMGNMG LGVNNPFHLN DILSLNVGHS LDDFHEDLNR SYFISYQLPA
cal-C10  NLKRVYAVDM QIAPASSQDK ELTGYSDLIL KLQTQQKVNF NLSVDDSGNQ DTGTYMGNIG IGVNNPFHLN DILSLNVGHS LDDFHEDLNR SYFISYQLPV
bau-A2   NLKRVYAVDM QITPATAQDK ELTGYSDLIL KLQPLQKVNF NLNVDDSGNQ DTGTYMGNIS LGVNNPFHLN DILSLNVSHS LDDFHESLNR SYFISYQLPV
bau-C4   NLKRVYAVDM QITPATAQDK ELTGYSDLIL KLQPLQKVNF NLSVDDSGNQ DTGTYMGNIG LGVNNPFHLN DILSLNVSHS LDDFHESLNR SYFISYQLPV
bau-C2   NLKRVYAVDM QITPATAQDK ELTGYSDLIL KLQALQKVNF NLSVDDSGNQ DTGTYMGNIG IGINNPFHLN DILSLNVSHS LDDFHESLNR SYFISYQLPV
pit-C7   NLKRVYAVDM QITPSSAQDK ELTGYSDLIL KLQPLQKFNF NLSVDDSGNQ DTGTYMGNIG IGVNNPFHLN DILSLNVSHS LDDFHESLNR SYFISYQLPV
nos-C8   NLKRVYAVDM QLTPAVSTDK ELTGFTDLIL KLQPLQKVNF NLSVDNSGNQ ETGTYMGNIS IGVNNPFHLN DILSLNVSHS LDNFHEDLNR SYFISYQLPV
pit-A3   NLKRVYAVDM QIAPASAQDK ELTGYSDLIL KLQPLQKVNF NLSVDDSGNQ DTGTYMGNIG LGVNNPFHLN DILSLNVSHS LDDFHESLNR SYFISYQLPV
pit-B6   NLKRVYAVDM QIAPASAQDK ELTGYSDLIL KLQPLQKVNF NLSVDDSGNQ DTGTYMGNIG LGVNNPFHLN DILSLNVSHS LDDFHESLNR SYFISYQLPV
cal-C11  NLKRVYAVDM QIAPANSQDK ELTGYSDLIL KLQPLQKVNF NLSVDDSGNQ DTGTYMGNIG LGVNNPFHLN DILSLNVSHS LDDFHEDLNR SYFISYQLPV
nos-C9   NLKRAYAVDM QLTPAVSTDK ELMGYTDLIL KLQPLQKVNF NLSVDNSGNQ ETGTYMGNIG VGVNNPFHLN DILSLNVSHS LDDFHENLNR SYFISYQLPF
bau-B1   NLKRAYTVDI QIVPSNESVQ DTQGYSDLVI KLQAHSKISL NLGLDNSGSK DTGKYIGSLG VSINNPFYLS DSLSFNFSHS LDNLHRDLNK NYFVSYQLPL
bau-B4   NLKRAYTVDI QIVPSNESVQ ETQGYSDLVI KLQAHSKISL NLGLDNSGSK DTGKYIGSLG VSINNPFYLS DSLSFNFSHS LDNLHRDLNK NYFVSYQLPL
bau-B2   NLKRAYTVDI QILPSNESVQ ETQGYSDLVI KLQAHSKISL NLGLDNSGSK DTGKYIGSLG VNINNPFYLS DSLSFNFSHS LDNLHRDLNK NYFVSYQLPL
bau-B3   NLKRAYTVDI QILPSNESVQ ETQGYSDLVI KLQAHSKISL NLGLDNSGSK DTGKYIGSLG VSINNPFYLS DSLSFNFSHS LDNLHRDLNK NYFVSYQLPL
pit-B5   NLKRAYTVDI QIVPSNESVQ EAKGYSDLII KLQPQSKINL NFALDNSGSK DTGRYIGSLG LNINNPFYLS DSLSFNFSHS LDNLHRDLNK NYFVSYQLPL
pit-B7   NLKRAYTVDI QIVPSNESAQ EAQGYSDLII KLQAHSKISL NLGLDNSGSE DTGKYIGSLG VNVNNPFYLS DSLSLNFSHS LDNLHRDLNK NYFVSYQVPL

         301                                                                                                 400
bau-A1   GYYDLGFSYN DYQYKQ-TVL GGNG-PLLYH GNSQQANLNL SRVISRSGQH KTSVYGKLYH KESQSFIDDI EINVLHRRTS GWNLGVQHRQ YLGNAVLDGS
bau-C1   GYYDLGFSYN DYQYKQ-TVL GGNG-PLLYH GNSQQANLNL SRVISRSGQH KTSVYGKLYH KESQSFIDDI EINVLHRRTS GWNLGVQHRQ YLGNAVLDGS
pit-A4   GYYDLGLSYN DYQYKQ-TVL GGNG-PLLYH GNSQQANLNL SRVISRSGQH KTSVYGKLYH KESQSFIDDI EINVLHRRTS GWNLGFQHRQ YLGNAVLDGS
pit-C5   GYYDLGLSYN DYQYKQ-TVL GGNG-PLLYH GNSQQANLNL SRVISRSGQH KTSVYGKLYH KESQSFIDDI EINVLHRRTS GWNLGFQHRQ YLGNAVLDGS
pit-C6   GYYDLGLSYN DYQYKQ-TVL GGNG-PLLYH GNSQQANLNL SRVISRSGQH KTSVYGKLYH KESQSFIDDI EINVLHRRTS GWNLGFQHRQ YLGNAVLDGS
pit-A5   GYYDLGLSYN DYQYKQ-TVL GGNG-PLLYH GNSQQANLNL SRVISRSGQH KTSVYGKLYH KESQSFIDDI EINVLHRRTS GWNLGFQHRQ YLGNAVLDGS
bau-C3   GYYDLGLSYN DYQYKQ-TVL GGNG-PLLYH GNSQQANLNL SRVISRSGQH KTSVYGKIYH KESQSFIDDI EINVLHRRTS GWNLGFQHRQ YLGNAVLDGS
cal-C10  SYYDLGFSYN DYQYKQ-TVL GANG-PLLYH GNSQQGNLNL SRVISRSGQH KTSVYGKLYH KESQSFIDDI EISVLHRKTS GWNLGFQHRQ YLGNAVLDGS
bau-A2   GYYDLGFSYN DYQYRQGTVA PESGYPVIYH GNSQQANLNL SRVISRSGQH KTSVYGKLYH KESQSFLNDI EINVLHRKTS GWNLGVQHRQ YLGNAVLDGS
bau-C4   GYYDLGFSYN DYQYRQGTVA PESGYPVIYH GNSQQANLNL SRVISRSGQH KTSVYGKLYH KESQSFLNDI EINVLHRKTS GWNLGVQHRQ YLGNAVLDGS
bau-C2   GYYDLGFSYN DYQYRQGTVA PESGYPVIYH GNSQQANLNL SRVISRSGQH KTSVYGKLYH KESQSFLNDI EINVLHRKTS GWNLGVQHRQ YLGNAVLDGS
pit-C7   GYYDLGFSYN DYQYRQGTVA PESGYPVIYH GNSQQANLNL SRVISRSGQH KTSVYGKLYH KESQSFLNDI EINVLHRKTS GWNLGVQHRQ YLGNAVLDGS
nos-C8   GYYDLGFSYN DYQYKQ-TVL GGNG-PLLYH GNSQQANLNL SRVISRSGQH KTSVYGKLYH KESQSFIDDI EINVLHRRTS GWNLGVQHRQ YLGNAVLDGS
pit-A3   GYYDLGFSYN DYYYRQGTVA PKSKYPVIYD GNSQQANLNL SRVISRSGQH KTSVYGKLYH KESQSFMNDI EVEVLHRKTS GWNLGFQHRQ YLGNAVLDGS
pit-B6   GYYDLGFSYN DYYYRQGTVA PKSKYPVIYD GNSQQANLNL SRVISRSGQH KTSVYGKLYH KESQSFMNDI EVEVLHRKTS GWNLGFQHRQ YLGNAVLDGS
cal-C11  GYYDLGLTYN DYQYSQRRVV SKD-YPIIYD GNSQQANLNL SRVISRSGQH KTSIYGKIYH KESQSFMNDI EVEVLHRKTS GWNLGFQHRQ YLGNAVLDGS
nos-C9   GYYDLGVSYN DYQYRQGTVT PK--YPVIYH GNSQQANLNL SRVISRSGQH KTSIYGKLYH KESQSFLNDI EINVLHRKTS GWVLGFQHRQ YLGNAVLDGS
bau-B1   GNYDFSTSYS RYQYEQ-NVL GANS-VLRYH GLSEQGNLNV SRVLSRSGQH KTTLYGKLYH KQNSNFIDDI EIEVQHRRTS GWNAGIQHRQ YLGDAVLDAG
bau-B4   GNYDFSTSYS RYQYEQ-NVL GANS-VLRYH GLSEQGNLNV SRVLSRSGQH KTTLYGKLYH KQNSNFIDDI EIEVQRRRTS GWNAGIQHRQ YLGDAVLDAG
bau-B2   GNYDFSTSYS RYQYEQ-NVL GANS-VLRYH GLSEQGNLNV SRVLSRSGQH KTSLYGKLYH KQNSNFIDDI EIEVQRRRTS GWNAGIQHRQ YLGVAVLDAG
bau-B3   GNYDFSTSYS RYQYEQ-NVL GANS-VLRYH GLSEQGNLNV SRVLSRSGQH KTSLYGKLYH KQNSNFIDDI EIEVQRRRTS GWNAGIQHRQ YLGVAVLDAG
```

```
pit-B5   GNYDFSTSYS RYQYEQ-NVL GANG-VLRYH GLSQQGNLNV SRVLSRSGQH KTSLYGKLYH KQNSNFIDDI EIEVQRRKTS GWNAGIQHRQ YLGDAVLDAG
pit-B7   GNYDFSTSYS RYQYEQ-NVL GANG-VLRYH GLSQQGNLNL SRVLSRSGQH KTSIYGKLYH KKNSNFIDDI EIEVQRRKTS GWNAGIQHRQ YLGDAVLDAG

         401                                                                                                 500
bau-A1   IDYRRGMGV- GARTAPEENI TDVYGNHLPV EGYSRAPLWS ADLRFTTPFL LLDKPAQYRF NWRGQYAPKI LVPNDRFYIG GRYSVRGFDG ELMLSGDNGQ
bau-C1   IDYRRGMGV- GARTAPEENI TDVYGNHLPV EGYSRAPLWS ADLRYITPFL LLDKPAQYRL NWRGQYAPKI LVPNDRFYIG GRYSVRGFDG ELMLSGDNGQ
pit-A4   IDYRRGMGV- GARTAPEENI TDVYGNHLPV EGYSRAPLWS ADLRYTTPFL LLEKPAQYRL NWRGQYAPKI LVPNDRFYIG GRYSVRGFDG ELMLSGDNGQ
pit-C5   IDYRRGMGV- GARTAPEENI TDVYGNHLPV EGYSRAPLWS ADLRYTTPFL LLEKPAQYRL NWRGQYAPKI LVPNDRFYIG GRYSVRGFDG ELMLSGDNGQ
pit-C6   IDYRRGMGV- GARTAPEENI TDVYGNHLPV EGYSRAPLLS ADLRYITPFL LLEKPAQYRL NWRGQYAPKI LVPNDRFYIG GRYSVRGFDG ELMLSGDNGQ
pit-A5   IDYRRGMGV- GARTAPEENI TDVYGNHLPV EGYSRAPLWS ADLRYTTPFL LLEKPAQYRL NWRGQYAPKI LVPNDRFYIG GRYSVRGFDG ELMLSGDNGQ
bau-C3   IDYRRGMGV- GARTAPEENI TDVYGNHLPV EGYSRAPLLS ADLRFSTPFL LLEKPAQYRL NWRGQYAPKI LVPNDRFYIG GRYSVRGFDG ELMLSGDNGQ
cal-C10  IDYRRGMGL- GARTAAEENI IDVNGNHLPV EGYSRAPLWS ADLRYTTPFL LLEKPAQYRL NWRGQYAPKI LVPNDRFYIG GRYSVRGFDG ELMLSGDNGQ
bau-A2   IDYRRGMGV- EARTAPEENI TDVYGNHLPI EGYSRAPLWS ADLRFTTPFL LLDKPAQYRL NWRGQYAPKI LVPNDRFYIG GRYSVRGFDG ELMLSGDNGQ
bau-C4   IDYRRGMGV- EARTAPEENI TDVYGNHLPV EGYSRAPLWS ADLRFTTPFL LLDKPAQYRF NWRGQYAPKI LVPNDRFYIG GRYSVRGFDG ELMLSGDNGQ
bau-C2   IDYRRGMGV- GARTAPEENI TDVYGNHLPV EGYSRAPLWS ADLRYTTPFL LLDKPAQYRL NWRGQYAPKI LVPNDRFYIG GRYSVRGFDG ELMLSGDNGQ
pit-C7   IDYRRGMGV- EARTAPEENI TDVYGNHLPV EGYSRAPLLS ADLRFSTPFL LLEKPAQYRL NWRGQYASKI LVPNDRFYIG GRYSVRGFDG ELMLSGDNGQ
nos-C8   IDYRRGMGV- GARTAPEENI TDVYGNHLPV EGYSRAPLWS ADLRFTTPFL LLDKPAQYRL NWRGQYAPKI LVPNDRFYIG GRYSVRGFDG ELMLSGDNGQ
pit-A3   IDYRRGMGV- EARTAAEEKI IDVNGNHLPV EGYSRAPLLS ADLRFSTPFL LLEKPAQYRL NWRGQYAPKI LVPNDRFYIG GRYSVRGFDG ELMLSGDNGQ
pit-B6   IDYRRGMGV- EARTAAEEKI IDVNGNHLPV EGYSRAPLLS ADLRFSTPFL LLEKPAQYRL NWRGQYAPKI LVPNDRFYIG GRYSVRGFDG ELMLSGDNGQ
cal-C11  IDYRRGVGV- GARTAAEENI TDNKGNHLPV EGYSRAPLLS ADLRFSTPFL LLEKPAQYRL NWRGQYAPKI LVPNDRFYIG GRYSVRGFDG ELMLSGDNGQ
nos-C9   IDYRHGMGI- NALTAPEENI TDINGKRLAV EGYSRAPLWN ADLRFTMPFL LLETPAQYRL NWRGQYAPKI LVPNDRFYIG GRYSVRGFDG ELMLSGDNGQ
bau-B1   LDYRHGTGAF DALLAPEEQI KDIDKNPLPS EGYSRAPIWS ADLRFQMPFL LLDTPAQYRL NWRGQYAPRV LVPNDRFYIG GRYSVRGFDG ELMLSGDNGQ
bau-B4   LDYRHGTGAF DALLAPEEQI KDIDKNPLPS EGYSRAPIWS ADLRFQMPFL LLDTPAQYRL NWRGQYAPRV LVPNDRFYIG GRYSVRGFDG ELMLSGDNGQ
bau-B2   LDYRHGTGAF DALLAPEEQI KDVDKNPLPP EGYSRAPIWS ADLRFQMPFQ LLDTPAQYRL NWRGQYAPRV LVPNDRFYIG GRYSVRGFDG ELMLSGDNGQ
bau-B3   LDYRHGTGAF DALLAPEEQI KDVDKNPLPP EGYSRAPIWS ADLRFQMPFQ LLVTPAQYRL NWRGQYAPRV LVPNDRFYIG GRYSVRGFDG ELMLSGDNGQ
pit-B5   LDYRHGTGAF DALLAPEEQI KDIDKNPLPS EGYSRAPIWS ADLRFQMPFL LLDTPAQYRL NWRGQYAPRV LVPNDRFYIG GRYSVRGFDG ELMLSGDNGQ
pit-B7   LDYRHGTGAF DALLAPEEQI KDIDKNPLPS EGYSRAPIWN ADLRFQMPFL LLDAPAQYRL NWRGQYAPRV LVPNDRFYIG GRYSVRGFDG ELMLSGDNGQ

         501                                                                       582
bau-A1   YLQQEISLNA PIPNTQFYMA VDQGWVNGRN SIPGQRYLLG SVLGLRTYQN SFYLDAFTGR GLIAPDSIKK DWVTGFSINL SY
bau-C1   YLQQEISLNA PIPNTQFYMA VDQGWVNGRN SIPGQRYLLG SVLGLRTYQN SFYLDAFTGR GLIAPDSIKK DWITGFSINL SY
pit-A4   YLQQEISLNA PIPNTQFYMA VDQGWVNGRN SIPGQRYLLG SVLGLRTYQN SFYLDAFTGR GLIAPDSIKK DWVTGFSINL SY
pit-C5   YLQQEISLNA PIPNTQFYMA VDQGWVNGRN SIPGQRYLLG SVLGLRTYQN SFYLDAFTGR GLIAPDSIKK DWVTGFSINL SY
pit-C6   YLQQEISLNA PIPNTQFYMA VDQGWVNGRN SIPGQRYLLG SVLGLRTYQN SFYLDAFTGR GLIAPDSIKK DWVTGFSINL SY
pit-A5   YLQQEISLNA PIPNTQFYMA IDQGWVNGRN SIPGQRYLLG SVLGLRTYQN SFYLDAFIGR GLIAPDSIKK DWVTGFSINL SY
bau-C3   YLQQEISLNA PIPNTQFYMA VDQGWVNGRN SIPGQRYLLG SVLGLRTYQN SFYLDAFTGR GLIAPDSIKK DWVTGFSINL SY
cal-C10  YLQQEISLNA SIPNTQFYMA VDQGWVNGRN SIPGQRYLLG SVLGLRTYQN SFYLDAFTGR GLIAPDSIKK DWVTGFSINL SY
bau-A2   YLQQEISLNA PIPNTQFYMA VDQGWVNGRN SIPGQRYLLG SVLGLRTYQN SFYLDAFTGR GLIAPDSIKK DWITGFSINL SY
bau-C4   YLQQEISLNA PIPNTQFYMA VDQGWVNGRN SIPGQRYLLG SVLGLRTYQN SFYLDAFTGR GLIAPDSIKK DWVTGFSINL SY
bau-C2   YVQQEISLNA PIPNTQFYMA VDQGWVNGRN SIPGQRYLLG SVLGLRTYQN SFYLDAFTGR GLIAPDSIKK DWVTGFSINL SY
pit-C7   YLQQEISLNV PIPNTQFYMA VDQGWVNGRN SIPGQRYLLG SVLGLRTYQN SFYLDAFTGR GLIAPDSIKK DWVTGFSINL SY
nos-C8   YLQQEISLNA PIPNTQFYMA VDQGWVNGRN SIPGQRYLLG SVLGLRTYQN SFYLDAFTGR GLIAPDSIKK DWVTGFSINL SY
pit-A3   YVQQEISLNA PIPNTQFYMA VDQGWVNGRN SIPGQRYLLG SVLGLRAYQN SFYLDAFTGR GLIAPDSIKK DWITGFSINL SY
pit-B6   YVQQEISLNA PIPNTQFYMA VDQGWVNGRN SIPGQRYLLG SVLGLRAYQN SFYLDAFTGR GLIAPDSIKK DWITGFSINL SY
cal-C11  YVQQEISLNA PIPNTQFYIA VDQGWVNGRN SIPGQRYLLG SVLGLRTYQN SFYLDAFTGR GLIAPDSIKK DWVTGFSINL SY
nos-C9   YLQQEISLNT PMPNTQFYMA VDQGWVNGRN SIPGQRYLLG SVLGLRTYQN SFYLDAFTGR GLIAPDSIKK DWVTGFSINL SY
bau-B1   YLQQEISVNT QIPNTQFYMA VDQGWVNGEN SIAGQRHLMG SVVGVRSYLN HFYIDAFTGR GLIAPQSIKK DWIFGFSLNA FY
bau-B4   YLQQEISVNT QIPNTQFYMA VDQGWVNGEN SIAGQRHLMG SVVGVRSYLN HFYIDAFTGR GLIAPQSIKK DWIFGFSLNA FY
bau-B2   YLQQEISVNT QLPNTQFYMA VDQGWVNGEN SIAGQRHLMG SVVGLRSYLN HFYIDAFTGR GLIAPKSIKK DWILGFSLNA FY
bau-B3   YLQQEISVNT QLPNTQFYMA VDQGWVNGEN SIAGQRHLMG SVVGLRSYLN HFYIDAFTGR GLIAPKSIKK DWILGFSLNA FY
pit-B5   YLQQEISVNT QIPNTQFYMA VDQGWVNGEN SIAGQRHLMG SVVGLRSYLN HFYIDAFTGR GLIAPKNIKK DWILGFSLNA FY
pit-B7   YLQQEISLNT KIPNTQMYVA VDQGWVNGKN SIAGQRHLMG SVVGLRSYLN HFYIDAFTGR GLIAPQSIKK DWIFGFSLNA FY
```

Supplementary file 4. Type-I CdiA alignment.

Residues color: red, high consensus value (90%); blu, moderate consensus value(50%)
CT domains at the COOH site are highlighted to evidentiate homologies.

```
        1                                                                                                     100
cal-C10 MNKNSYRIIY SKARQMFVAV AENVRSQTKA SGQSEAS--A HNQINNTESQ AFHQLWQVKA LVASMSLWMP LAPAYAAIVA DSAANAANRA VIAAGKNAAG
bau-C4  MNKNSYRIIY SKARQMFVAV AENVRSQTKT SGQSEAS--T RNNIDNNESQ AFHQLWQVKA LVASISLWMP LAPVYAGIVA DSAANAANRA VIGAGKNSAG
pit-C5  MNKNSYRIIY SKARQMFVAV AENVRSQTKT SGQSEAS--T QTNIDNTESQ AFHQLWQVKA LVASISLWMP LAPVYAGIVA DSAANAANRA VIGAGKNSAG
bau-C3  MNKNSYRIIY SKARQMFVAV AENVRSQTKA SGQSEAS--T QNNIDNTESQ AFHQLWQVKA LVASISLWMP LAPVYAGIVA DSAANAANRA VIGAGKNSAG
cal-C11 MNKNSYRIIY SKARQMFVAV AENVRSQTKA SGQSEAS--S QNQINNTESQ AFHQLWQVKA LVASMSLWMP LAPVYAGIVA DSAANAANRA VIGAGKNSAG
pit-C7  MNKNSYRIIY SKARQMFVAV AENVRSQTKT SGQSEAS--T QNNIDNTESQ AFHQLWQVKA LVASISLWMP LAPVYAGIVA DSAANAANRA VIGAGKNSAG
bau-C1  MNKNSYRIIY SKARQMFVAV AENVRSQTKT SGQSEVS--T QSNIDKTESQ AFHQLWQVKA LVASISLWMP LAPVYAGIVA DSAANAANRA VIGAGKNSAG
pit-A4  MNKNSYRIIY SKARQMFVAV AENVRSQTKT SGQSEAS--T QTNIDNTESQ AFHQLWQVKA LVASISLWMP LAPVYAGIVA DSAANAANRA VIGAGKNSAG
pit-A5  MNKNSYRIIY SKARQMFVAV AENVRSQTKT SGQSEAS--T QNNIDNTESQ AFHQLWQVKA LVASISLWMP LAPVYAGIVA DSAANAANRA VIGAGKNSAG
pit-C6  MNKNSYRIIY SKARQMFVAV AENVRSQTKA SGQSEAS--A QSNIDNTESQ AFHQLWQVKA LVASISLWMP LAPVYAGIVA DSAANAANRA VIGAGKNSAG
bau-C2  MNKNSYRIIY SKARQMFVAV AENVRSQTKT SGQSEAS--T QNNIDNTESQ AFHQLWQVKA LVASISLWMP LAPVYAGIVA DSAANAANRA VIGAGKNSAG
nos-C8  MNKNSYRIIY SKARQMFVAV AENVRSQTKA SGQSEAS--T QNNIDNTESQ AFHQLWQVKA LVASISLWMP LAPVYAGIVA DSAANAANRA VIGADKNSAG
pit-B7  MNKNLYRIVF NKARNMFMAV AENVRSQSKE AGQSTSSEIE RSGQSSGQEK SFHQLWHVKA LVVSISFLVP FAPVYAAIVA DSAANAANRA VIGAGKNSAG
bau-B4  MNKNLYRIVF NKARNMFMAV AENVRSQSKA TGQSTSSEIV SSGQLLVQEK SFHQLWHVKA LVTSMSLMLP FAPVYADIIA DSTANAANRA VIAAGKNSAG
nos-C9  MNKNSYRIIY SKVRQMFVAV AENVKSQTKT SGQSQAN--S QSPI-NHESQ VFHQLWQVKA LVASMSFWMP LAPVYADIVA DNAANAENRA VIAAGRNAQG
bau-B1  MNKNLYRIVF NKARNMFMAV AENVRSQSKA TGQSTSSEIV SSGQLLVQEK SFHQLWHVKA LVTSMSLMVP FAPVYADIIA DSAANAANRA VIAAGKNSAG
pit-B5  MNKNLYRIVF NKARNMFMAV AENVRSQSKA AGQSTSSEIV SSGQSLVQEK SFHQLWHVKA LVASISLLVP FAPVYAAIVA DSAANAANRA VIGAGKNSTG
bau-B3  MNKNLYRIIF NKARNMFMAV AENVRSQSKA AGQSSSSEIV SYGQSSGQEK SFHQLWHVKA LVTSMSLLVP FAPVYADIVA DSAANAANRA VIAAGKNSAG
bau-B2  MNKNLYRIIF NKARNMFMAV AENVRSQSKA AGQSSSSEIV SYGQSSGQEK SFHQLWHVKA LVTSMSLLVP FAPVYADIVA DSAANAANRA IIAAGKNSAG
pit-B6  MNKNLYRIVF NKARNMFMAV AENVRSQSKA AGQSTSSEIE RSGQSSGQEK SFHQLWHVKA LVASISLLVP FAPVYAAIVA DSAANAANRA VIGAGKNSAG
bau-A2  MNKNSYRIIY SKARQMFVAV AENVRSQTKT SGQSEAS--T QNKIDNTESQ AFHQLWQVKA LVASISLWMP LAPVYAGIVA DSAANAANRA VIGAGKNSAG
bau-A1  MNKNSYRIIY SKARQMFVAV AENVRSQTKT SGQSEAS--T RNNIDNNESQ AFHQLWQVKA LVASISLWMP LAPVYAGIVA DSAANAANRA VIGAGKNSAG
pit-A3  MNKNSYRIIY SKARQMFVAV AENVRSQTKA SGQSEAS--T QSNTDNTESQ AFHQLWQVKA LVASISLWMP LAPVYAGIVA DSAANAANRA VIGAGKNSAG

        101                                                                                                   200
cal-C10 TVVPVVNIQT PKNGISHNIY KQFDVLAEGA VLNNSRQGAT TKTVGNVAAN PFLATGEARV ILNEVNSSAA SRFEGNLEVA GQMADVIIAN PSGINIKGGG
bau-C4  TVVPVVNIQT PKNGISHNIY KQFDVLAEGT VLNNSRTGAT TKTVGAVGAN PFLATGEARV ILNEVNSSAA SRFEGNLEVA GQMADVIIAN PSGINIKGGG
pit-C5  TVVPVVNIQT PKNGISHNIY KQFDVLAEGA VLNNSRQGAT TKTVGNVAAN PFLATGEARV ILNEVNSSAA SRFEGNLEVA GQMADVIIAN PSGINIKGGG
bau-C3  TVVPVVNIQT PKNGISHNIY KQFDVLAEGA VLNNSRQGAT TKTVGNVAAN PFLATGEARV ILNEVNSSAA SRFEGNLEVA GQMADVIIAN PSGISIKGGG
cal-C11 TVVPVVNIQT PKNGISHNIY KQFDVLAEGA VLNNSRQGAT TKTVGNVAAN PFLATGEARV ILNEVNSSAA SRFEGNLEVA GQMADVIIAN PSGISIKGGG
pit-C7  TVVPVVNIQT PKNGISHNIY KQFDVLAEGA VLNNSRQGAT TQTVGNVAAN PFLATGEARV ILNEVNSSAA SRFEGNLEVA GQMADVIIAN PSGINIKGGG
bau-C1  TVVPVVNIQT PKNGISHNIY KQFDVLAEGA VLNNSRQGAT TKTVGNVAAN PFLATGEARV ILNEVNSSAA SRFEGNLEVA GQMADVIIAN PSGISIKGGG
pit-A4  TVVPVVNIQT PKNGISHNIY KQFDVLAEGA VLNNSRQGAT TKTVGNVAAN PFLATGEARV ILNEVNSSAA SRFEGNLEVA GQMADVIIAN PSGINIKGGG
pit-A5  TVVPVVNIQT PKNGISHNIY KQFDVLAEGA VLNNSRQGAT TKTVGNVAAN PFLATGEARV ILNEVNSSAA SRFEGNLEVA GQMADVIIAN PSGISIKGGG
pit-C6  TVVPVVNIQT PKNGISHNIY KQFDVLAEGA VLNNSRQGAT TKTVGNVAAN PFLATGEARV ILNEVNSSAA SRFEGNLEVA GQMADVIIAN PSGINIKGGG
bau-C2  TVVPVVNIQT PKNGISHNIY KQFDVLAEGA VLNNSRQGAT TKTVGNVAAN PFLATGEARV ILNEVNSSAA SRFEGNLEVA GQMADVIIAN PSGINIKGGG
nos-C8  TVVPVVNIQT PKNGISHNIY KQFDVLAEGA VLNNSRQGAT TQTVGNVAAN PFLATGEARV ILNEVNSSAA SRFEGNLEVA GQMADVIIAN PSGINIKGGG
pit-B7  VTVPVVNIQT PKNGISHNIY KQFDVLAEGA VLNNSRTGAT TKTVGAVGAN PFLATGEARV ILNEVNSTAA SRFEGNLEVA GQMADVIIAN PSGINIKGGG
bau-B4  TTVPVVNIQT PKNGISHNIY KQFDVLAEGV VLNNSRTGAI TKTVGGVGAN PFLATGEARV ILNEVNSTVA SRFEGNLEVA GQMADVIIAN PSGINIKGGG
nos-C9  AVVPVVNIQT PKNGVSHNIY QQFDVLAEGA VLNNSRQGAA TQTVGNVSAN PFLATGEARV ILNEVNSSAA SRFEGNLEVA GQMADVIIAN PSGINIKGGG
bau-B1  TTVPVVNIQT PKNGISHNIY KQFDVLAEGA VLNNSRTGAI TKTIGGVGAN PFLATGEARV ILNEVNSTVA SRFEGNLEVA GQMADVIIAN PSGINIKGGG
pit-B5  TTVPVVNIQT PKNGISHNIY KQFDVLAEGA VLNNSRTGAT TKTVGTVGAN PFLATGEARV ILNEVNSTAA SRFEGNLEVA GQMADVIIAN PSGINIKGGG
bau-B3  TTVPVVNIQT PKNGISHNIY KQFDVLAEGA VLNNSRQGAT TKTVGAVAAN PFLATGEARV ILNEVNSSAA SRFEGNLEVA GQMADVIIAN PSGINIKGGG
bau-B2  KTVPVVNIQT PKNGISHNIY KQFDVLAEGA VLNNSRQGAT TKTVGAVAAN PFLATGEARV ILNEVNSSAA SRFEGNLEVA GQMADVIIAN PSGINIKGGG
pit-B6  TTVPVVNIQT PKNGISHNIY KQFDVLAEGA VLNNSRTGAT TKTVGAVGAN PFLATGEARV ILNEVNSTTA SRFEGNLEVA GQMADVIIAN PSGINIKGGG
bau-A2  TVVPVVNIQT PKNGISHNIY KQFDVLAEGA VLNNSRQGAT TKTVGNVAAN PFLATGEARV ILNEVNSSAA SRFEGNLEVA GQMADVIIAN PSGISIKGGG
bau-A1  TVVPVVNIQT PKNGISHNIY KQFDVLAEGT VLNNSRTGAT TKTVGAVGAN PFLATGEARV ILNEVNSSAA SRFEGNLEVA GQMADVIIAN PSGINIKGGG
pit-A3  TVVPVVNIQT PKNGISHNIY KQFDVLAEGA VLNNSRTGAT TKTVGAVGAN PFLATGEARV ILNEVNSSAA SRFEGNLEVA GQMADVIIAN PSGINIKGGG

        201                                                                                                   300
cal-C10 FINANKAIFT TGKPQLNADG SIKQFTVDQG KITVSANPNS KFGLGGNNND ANYVDLYARA LELSAELRAK NDIQVIAGAN NVSADLQDVT AKTGTGTAPT
bau-C4  FINANKAIFT TGKPQLNADG SIKQFTVDQG KITVSANPNS KFGLGGNNND ANYVDLYARA LELSAELRAK NDIQVIAGAN NISADLQDVT AKTGTGTAPT
pit-C5  FINANKAIFT TGKPQLNADG SIKQFTVDQG KITVSANPNS KFGLGGNNND ANYVDLYARA LELSAELRAK NDIQVIAGAN NVSADLQDVT PKTGTGTAPT
bau-C3  FINANKAIFT TGKPQLNADG SIKQFTVDQG KITVSANPNS KFGLGGNNND ANYVDLYARA LELSAELRAK NDIQVIAGAN NVSADLQDVT AKTGTGTAPT
cal-C11 FINANKAIFT TGKPQLNADG SIKQFTVDQG KITVTANPNS KFGLGGNNND ANYVDLYARA LELSAELRAK NDIQVITGAN NVSADLQDIT AKTGTGTSPT
pit-C7  FINANKAIFT TGKPQLNADG SIKQFTVDQG KITVSANPNS KFGLGGNNND ANYVDLYARA LELSAELRAK NDIQVIAGAN NVSADLQDVT AKTGTGTAPT
bau-C1  FINANKAIFT TGKPQLNADG SIKQFTVDQG KITVSANPNS KFGLGGNNND ANYVDLYARA LELSAELRAK NDIQVIAGAN NVSADLQDVT AKTGTGTAPT
pit-A4  FINANKAIFT TGKPQLNADG SIKQFTVDQG KITVSANPNS KFGLGGNNND ANYVDLYARA LELSAELRAK NDIQVIAGAN NVSADLQDVT PKTGTGTAPT
pit-A5  FINANKAIFT TGKPQLNADG SIKQFTIDQG KITVSANPNS KFGLGGNNND ANYVDLYARA LELSAELRAK NDIQVIAGAN NVSADLQDVT AKTGTGTAPT
pit-C6  FINANKAIFT TGKPQLNADG SIKQFTIDQG KITVSANPNS KFGLGGNNND ANYVDLYARA LELSAELRAK NDIQVIAGAN NVSADLQDVT AKTGTGTAPT
bau-C2  FINANKAIFT TGKPQLNADG SIKQFTVDQG KITVSANPNS KFGLGGNNND ANYVDLYARA LELSAELRAK NDIQVIAGAN NVSADLQDVT PKTGTGTAPT
nos-C8  FINANKAIFT TGKPQLNADG SIKQFTVDQG KITVSANPNS KFGLGGNNND ANYVDLYARA LELSAELRAK NDIQVIAGAN NISTDLQGVT AKTGTGTAPT
pit-B7  FINANKAIFT TGKPQLNADG SIQQFNVSQG KVTITAPTNS TFGLGGNNNN ANYVDIYARA LDLNAKLQAT NDIQVITGAN TVSSDLKNIA PISGAGTAPT
bau-B4  FINANKAIFT TGKPQLNADG SIQQFNVSQG KVTITAPTNS ILGLGGNNNN ANYVDIYARA LDLNAQLHAT NDIQVIIGAN TVSSDLKNIA SISGTGTVPT
nos-C9  FINANKAIFT TGKPQLNADG SIKQFTVDQG KITVSANPNS KFGLGGNNND ANYIDLYARA LELSAELRAK NDIQVITGAN DISADLQDTT AKTGTGTTPT
bau-B1  FINANKAIFT TGKPQLNADG SIKQFTVDQG RITISANPNS KFGLGGNNND ANYVDLYARA LELSAELRAK NDIQVIAGAN NVSADLQDVT AKTGTGTAPT
pit-B5  FINANKAIFT TGKPQLNADG SIKQFTVDQG KITVSANPNS KFGLGGNNND ANYVDLYTRA LELSAELRAK NDIQVIAGAN NVSADLQDVT AKTGTGTAQT
bau-B3  FINANKAIFT TGKPQLNADG SIKQFTVDQG RITVSANPNS KFGLGGNNND ANYVDLYARA LELSAELRAK NDIQVIAGAN NVSADLQEVT AKTGTGTAPA
bau-B2  FINANKAIFT TGKPQLNADG SIKQFTVDQG RITVSANPNS KFGLGGNNND ANYVDLYARA LELSAELRAK NDIQVIAGAN NVSADLQEVT AKTGTGTAPA
pit-B6  FINANKAIFT TGKPQLNADG SIKQFTVDQG RITVSANPNS KFGLGGNNND TNYVDLYARA LELSAELRAK NDIQVIAGAN NVSADLQDVT SKTGTGTAPT
bau-A2  FINANKAIFT TGKPQLNADG SIKQFTVDQG KITVSANPNS KFGLGGNNND ANYVDLYARA LELSAELRAK NDIQVIAGAN NVSADLQDVT AKTGTGTAPT
bau-A1  FINANKAIFT TGKPQLNADG SIKQFTVDQG KITVSANPNS KFGLGGNNND ANYVDLYARA LELSAELRAK NDIQVIAGAN NVSADLQDVT AKTGTGTAPT
pit-A3  FINANKAIFT TGKPQLNADG SIKQFTVDQG KITVSANPNS KFGLGGNNND ANYVDLYARA LELSAELRAK NDIQVIAGAN NISTDLQGVT AKTGTGTAPT

        301                                                                                                   400
cal-C10 LAVDVKALGG MYANNIYLVG TEKGLGVTNA GTIQAVNNLV ITSAGKIENK GTIESTSPTE SWVNIETIGA ATDGDIINSG VIVSKGPLSI KSSNNINLNG
bau-C4  LAVDVKALGG MYANNIYLMG TEKGLGVTNA GTIQAVNNLV ITSAGKIENK GTLESTSPTE SWLNIETIGV ATDGDILNSG AIVSKGPLSI KSSNNINLNG
pit-C5  LAVDVKALGG MYANNIYLMG TEKGLGVTNA GTVQAVNNLV ITSAGKIEHS GTISSTSKTQ GLVNIQTTGT GAAADINSSG SINSNSALNI DSGNNLNVNA
bau-C3  LAVDVKALGG MYANNIYLMG TEKGLGVTNA GTIQAVNNLV ITSAGKIEHS GTISSTSKTQ GLVSVQTTGT GAASDINSSG SINSNSMLNI DSGNNLNVNA
cal-C11 LAVDVKALGG MYANNIYLMG TEKGLGVTNA GTIQAVNNLV ITSAGKIEHS GTISSTSKTQ GLVNIQTTGT GAAADINSSG SINSNSMINI DSGNNLNVNA
pit-C7  LAVDVKALGG MYANNIYLMG TEKGLGVTNA GTIQAVNNLV ITSAGKIEHS GTISSTSKTQ GLVNIQTTGT DAAGDINSSG SINSNSMLNI DSGNNLNVNA
bau-C1  LAVDVKALGG MYANNIYLMG TEKGLGVTNA GTIQAVNNLV ITSAGKIEHS GTISSNSKTQ GLVNIQTTGT GAAADINSSG SINSNSMLNI DSGNNLNVNA
pit-A4  LAVDVKALGG MYANNIYLMG TEKGLGVTNA GTVQAVNNLV ITSAGKIEHS GTISSTSKTQ GLVNIQTTGT GAAADINSSG SINSNSALNI DSGNNLNVNA
pit-A5  LAVDVKALGG MYANNIYLMG TEKGLGVTNA GTIQAVNNLV ITSAGKIEHS GTFSSTSKTQ GLVNIQTTGT GAVADINSSG SINSNSMLNI DSGNNLNVNA
pit-C6  LAVDVKALGG MYANNIYLMG TEKGLGVTNA GTIQAVNNLV ITSAGKIEHS GTISSTSKTQ GLVNIQTTGT GAAADINSSG SINSNSMLNI DSGNNLNVNA
bau-C2  LAVDVKALGG MYANNIYLMG TEKGLGVTNA GTIQAVNNLV ITSAGKIEHS GTISSTSKTQ GLVNIQTTGV GAAADINSSG SINSNSALNI DSGNNLNVNA
nos-C8  LAVDVKALGG MYANNIYLMG TEKGLGVTNA GTIQAVNNLV VTSAGKIEHS GTISSTNKTQ GLVNIQTTGT GAAADINSSG SINSNSALNI DSGNNLNVNA
pit-B7  LAIDVKALGG MYANNIYLMG TEKGLGVTNA GTLQAVNNLI ITSAGKIEHS GTISSTSKTQ GLVSVQTTGV GAAADINSSG SINSNGMLNI DSGNNLNVNA
bau-B4  LAVDVKALGG MYANNIYLMG TEKGLGVTNA GTIQAVNNLV ITSAGKIEHS GTISSTSKTQ GLVSVQTTGV GAAADINSSG SINSNGMLNI DSGNNLNVNA
nos-C9  LAVDVKALGG MYANNIYLVG TEKGLGVSNA GTLEALNSLV VTSAGKIENT GALYNKNPQN SLLSLSS--- GEGNDIVHTG TMITNGNLIL DSGKDIIVDH
bau-B1  LAVDVKALGG MYANNIYLMG TEKGLGVTNA GTLQAVNNLV ITSAGKIEHS GTISSTSKTQ GLVSVQTTGV GVAADINSSG SINSNGMLNI DSGNNLNVNA
pit-B5  LAVDVKALGG MYANNIYLMG TEKGLGVTNA GTLQAVNNLV ITSAGKIEHS GTISSTSKTQ GLVSLQTTGT GAVADINSSG SINSNSMLNI DSGNNLNVNA
bau-B3  LAVDVKALGG MYANNIYLMG TEKGLGVTNA GTLQAVNNLV ITSAGKIEHS GTISSTSKTQ GLVSVQTTGV GAAADINSSG SINSNSMLNI DSGNDLNVNA
bau-B2  LAVDVKALGG MYANNIYLMG TEKGLGVTNA GTIQAVNNLV ITSAGKIENN GTIKTTGSQN SVLNLSSQNN G---DFTSSG AIQSNGNIFI SSDRNLALNG
pit-B6  LAVDVKALGG MYANNIYLMG TEKGLGVTNA GTIQAVNNMV ITSAGKIENS GTIKTTGSQN SVLNLSSQNN G---DFTSSG AIQSNGNIFI SSDRNLALNG
```

```
bau-A2  LAVDVKALGG MYANNIYLMG TEKGLGVTNA GTIQAVNNLV ITSAGKIEHS GTISSTSKTQ GLVNIQTTGT GAASDINSSG SINSNSMLNI DSGNNLNVNA
bau-A1  LAVDVKALGG MYANNIYLMG TEKGLGVTNA GTIQAVNNLV ITSAGKIEHS GTISSTNKTQ GLVNIQTTGT GAAGDINNSG TISGKGIVNL DSANDLNITA
pit-A3  LAVDVKALGG MYANNIYLMG TEKGLGVTNA GTIQAVNNLV ITSAGKIEHN GTISSTSKTQ GLVNIQTTGT GVAGDINNSG TISGKGIVNL DSANDLNITA

        401                                                                                                  500
cal-C10 G-INLNGGRI QSTAIKPVPV IIEAKGDIKL SERASISNLK LGSNMYLNAN NIELDNKSSI SSN-GANSVI AKNGIVLKNN SSITSIYDLD V-SAQKIDSS
bau-C4  G-INLNGGRI QSTAIKPVPV IIEAKGDIKL SERASISNLK LGSNMYLNAN NIELENKSSI SSN-GANSVI AKNGIILKNN SSITSIYDLD V-SAQKIDSS
pit-C5  KEIIINNGSL ASS-----PL IVNAKGNINL AADTRIMDDS QGGDVYIDAA NINLAAGSEL KSNRGTATIQ VQKDLVAAKG AKLIAAKDLN VLSNGKLSLT
bau-C3  KEIIINNGSL ASS-----PL MINTKGNLNL AANSRIFNDA QSGDVYVDAA NINLAANAGI TSNRGTASIQ VQKDLVAAKG AKLIAAKDLN VLSNGKLSLT
cal-C11 KEIIINNGSL ASS-----PL IVNTKGNINL AADTRIMDDS QGGDVYIDAA NINLAAGSEL KSNRGTATIQ VQKDLVAAKG AKLIAAQDLN VLSNGKLSLT
pit-C7  KEIIINVGSL ASS-----PL IVNAKGNINL AADTRIMDDS QGGDVYIDAA NINLAAGSEL KSNRGTATIQ VQKDLVAAKG AKLIAAKDLN VLSNGKLSLT
bau-C1  KEIIINNGSL ASS-----PL MINTKGNLNL AANSRIFNDA QSGDVYVDAA NINLAANAGI TSNRGTASIQ VQKDLVAAKG AKLIAAKDLN VLSNGKLSLT
pit-A4  KEIIINNGSL ASS-----PL IVNAKGNINL AADTRIMDDS QGGDVYIDAA NINLAAGSEL KSNRGTATIQ VQKDLVAAKG AKLIAAKDLN VLSNGKLSLT
pit-A5  KEIIINNGSL ASS-----PL IVNTKGNINL AADTRIMDDS QGGDVYIDAA NINLAAGSEL KSNRGTATIQ VQKDLVAAKG AKLIAAKDLN VLSNGKLSLT
pit-C6  KEIIINVGSL ASS-----PL IVNAKGNINL AADTRIMDDS QGGDVYIDAA NINLAAGSEL KSNRGTATIQ VQKDLVAAKG AKLIAAKDLN ILSNGKLSLT
bau-C2  KEIIINNGSL ASS-----PL IVNAKGNINL AADTRIMDDS QGGDVYIDAA NINLAAGSEL KSNRGTATIQ VQKDLVAAKG AKLIAAKDLN VLSNGKLSLT
nos-C8  KEIIINNGSL ASS-----PL IVNVKGNINL AADTRIMDDS QGGDVYIDAA NINLAAGSEL KSNRGTATIQ VKKDLVAAKG AKLIAAQDLN VLSNGKLSLT
pit-B7  KEIIINNGSL ASS-----PL IISSKGNLSL AANSRIFNDA QSGELYVDAA NINLATNAGI TSNRGSAYIQ SQKDVVAAQG AKLIAAQNLN VSGKGKLSLT
bau-B4  KEIIINNGSL ASS-----PL IINTKGNLNL AANSRIFNDA QSGELYVDAA NINLATNAGI TSNRGSAYIQ SQKDVVTEQG AKLIAAQNLN VSGRGKLSLN
nos-C9  ARIEKHGADN KNI------V SLSAKNDINI KNSANIQNFG EGGDLYLDAN NINLGNDVNL GVN-GAISLQ AKQNLQGDRV RNISSMYDLN LYAGNLLSFN
bau-B1  KEIIINNGSL ASS-----PL IINTKGNLNL AANSRIFNDA QSGELYVDAA NINLATNAGI TSNRGSAYIQ SQKDVVAAQG AKLIAAQNLN VSGRGKLSLN
pit-B5  KEIIINNGSL AAS-----PL IISSKGNLNL AANSRIFNDA QSGDVYVDAA NINLATNAGI TSNRGSAYIQ SQKDVVAAQG AKLIAAQNLN VSGKGKLSLN
bau-B3  KEIIINNGSL AAS-----PL IISSKGKVNL AANSRIFNDA QSGDVYVDAA NINLATNAGI TSNRGSAYIQ SQKDVVAAQG AKLIAAQNLN VSGKGKLSLN
bau-B2  GKIDKLGTTL SS-------I FLSAKGDVSL QNDVNIQNAG VGGGIYSDSL NFNLGKDSNI LSQ-GDISLL ANNNIDIQNG KGITSNNDLS IQSKNNLNIA
pit-B6  GKIDKLGTTI SS-------I FLSAKGDVSL QNGVNVQNVG VGGGIYSDSL NFNLGKDSNI LSQ-GDISLL ANNNIDIQNG KGITSNNDLS IQSKNNLNIA
bau-A2  KEIIINNSSL ASS-----PL IVNTKGNINL AANTRIMDDS QGGDVYIDAA NINLAAGSEL KSNRGTATIQ VQKDLVAAKG AKLIAAQDLN VLSNGKLSLT
bau-A1  KEVKIDEGN- -NT-----AL ILAANGNINL KNEIQLKNSA VDGGIYLNAN NINLE-GNAT VDGKADVNIV GKESILIKKN NKIKSGKSIS ILGGG-LSLE
pit-A3  KEVKIDEGN- -NT-----AL ILAANGNINL KNEIQLKNSA VDGGIYLNAN NINLE-GNAT IDGKADVNIV GKENVLIKKN NKIKSGKSIS ILGSG-LSLE

        501                                                                                                  600
cal-C10 YSSLVANKGN INIVGSSDDR TLGNIDLYET ILNAEGDVSL YSSGNTLLKK LDITTLNDGN SKIKNFKMYS KNNL-LWDYL TKAPPQFVGK IQAEAGGKLT
bau-C4  YSSLVANKGN INIVGNSDDG NLGNINLSET ILNAVGNVSL YSSGNTLLKN LDITTLNDGN SKIKNFKLYS KNNL-LWDYL TKAPPQFVGK IQAEAGEKLT
pit-C5  ENHIQASLGS INLQANSANI Q-NLIDLQGG TIYAGKDLNL YSSGDLNLKN LGFS-LENAA TRVKNIKAYS GRDL-VWNNA DKALPLITGL VQLDAANNLT
bau-C3  ENHIQASLGS INLQANSANT Q-NLIDLQGG TIYAGKDLNL YSSGDLNLKN LGFS-LENAA TRVKNIKAYS GRDL-VWNNA DKALPLITGM VQLDAANNLT
cal-C11 ENHIQASLGS INLQANSANT Q-NLIDLQGG TIYAGKDLNL YSSGDLNLKN LGFS-LENTA TRVKNIKAYS GRNL-VWNNA DKALPLITGM VQLDAANNLT
pit-C7  ENHIQASLGS INLQANSANI Q-NLIDLQGG TIYAGKDLNL YSSGDLNLKN LGFS-LENAA TRVKNIKAYS GRDL-VWNNA DKALPLITGL VQLDAANNLT
bau-C1  ENHIQASLGS INLQANSANT Q-NLIDLQGG TIYAGKDLNL YSSGDLNLKN LGFS-LENAA TRVKNIKAYS GRDL-VWNNA DKALPLITGM VQLDAANNLT
pit-A4  ENHIQASLGS INLQANSANI Q-NLIDLQGG TIYAGKDLNL YSSGDLNLKN LGFS-LENAA TRVKNIKAYS GRDL-VWNNA DKALPLITGL VQLDAANNLT
pit-A5  ENHIQASLGS INLQANSANT Q-NLIDLQGG TIYAGKDLNL YSSGDLNLKN LGFS-LENAA TRVKNIKAYS GRDL-VWNNA DKALPLITGL VQLDAANNLT
pit-C6  ENHIQASLGS INLQANSANT Q-NLIDLQGG TIYAGKDLNL YSSGDLNLKN LGFS-LENAA TRVKNIKAYS GRDL-VWNNA DKALPLITGL VQLDAANNLT
bau-C2  ENHIQASLGS INLQADSANT Q-NIIDLQGG TIYAGKDLNL YSSGDLNLKN LGFS-LENAA TRVKNIKAYS GRDL-VWNNA DKALPLITGM VQLDAANNLT
nos-C8  ENHIQASLGS INLQANSANT Q-NLIDLQGG TIYAGKDLNL YSSGDLNLKN LGFS-LENAA TRVKNIKAYS GRDL-VWNNA DKALPLITGL VQLDAANNLT
pit-B7  GNQIQASLGS INLQADSSNT D-GLIDLQGG TIYGGKDLNL YSSGDVNLQN LGFA-LENSA TRVKNINAYS GRNL-IWDNT AKALPQISGK VVLEAENNLT
bau-B4  GNQIQASLGS INLQADSSNT D-GLIDVQGG TIYGGKDLNL YSSGDVNLQN LGFA-LENSA TRVKNINAHS GRNL-VWNNA TKVLPQITGK VVLDAENNLS
nos-C9  NTYIWANFGN LNF--NTANA NSNLI-LDWN GFNVGKDINI YSSGDLTLKN LGFV-LDNSV TRSKNFNAYS GHNL-VWDSS SRALPQINGD IKVEAGNIID
bau-B1  ENQIQASLGS INLQVDSSNT D-GLIDIRGG TIYGGKDLNL YSSGDVNLQN LGFA-LENYV TRVKNINAHS GRNL-VWNNA TKVLPQITGK VALDAENNLS
pit-B5  ENQIQASLGS INLQADSSNT D-GLIDIRGG TIYGGKDLNL YSSGDVNLQN LGFA-LENSA TRVKNINAHS GRNL-VWNNA TKVLPQITGK VALDAENNLS
bau-B3  ENQIQASLGS INLQADSSNT D-GLIDIRGG TIYGGKDLNL YSSGDVNLQN LGFA-LENSA TRVKNINAHS GRNL-VWNNA TKVLPQITGK VALDAESNLS
bau-B2  NSSIESKAGN VNL-ISLGNT ASSLLGINNT KLYAGKDFNI KGAKNIKLNS ------SIVQ ENSNNINLYA GNDIEIGNDV SPVSLKTQGT INIAAKNNLI
pit-B6  NSSIESKAGN VNL-ISLGNT ASSLLGINNT KLYAGKDFNI KGAKNIKLNS ------SIVQ ENSNNINLYA DNDIEIGNDV SPVSLKTQGT INIAAKNNLI
bau-A2  ENHIQASLGT INLQANSANT Q-NLIDLQGG TIYAGKDLNL YSTDNINMKN LSFS-IENNK NRVKNINIYS GGSL-YWDNT AFLIPDLTGK ISLRAEKDLT
bau-A1  GSELTSGQN- VNLQNSSVNK K-LLIN--DS VIKAEEDYNF YSNGDLSINK IDFS-QSKDN LKKQNIKVGS GGIL-DLGDN FTFNSKGGGA FESRGNINLG
pit-A3  GSELTSGQN- INLQSTGVNK K-LLIN--DS IIKAEENYNF YSHEDLSINK IDFS-QNKDN LRKQNIKIGS GGIL-ELGDN FTFNSKGGAN FESKSNINLG

        601                                                                                                  700
cal-C10 LSGKKLIALD GIDLQASQLN LDSELDSQKN INITSKKDDL ILEKNLNAQN DINLTALTGG VTANSLKATS SAGKISILAN KNVNLNSTQT TKAMPSADKD
bau-C4  LSGKKLIALD GIDLQASQLK LDSELDSQKN INITSKKDDL ILEKNLNTQN DINITALAGG VTANSLKATS SAGKISILAN KNLNLNSTQT TKAMPSADKD
pit-C5  VTAKEISNKD SIQLHANQIA LNSALTSQKN IDVSSEIADL VLSQALKAQG DINLTALAGG VTANSLKATS SAGEISILAN KNISLNSTQT TKAMPSADKD
bau-C3  VTAKEISNKD SIQLHANQIA LNSALTSQKN IDVSSEIADL VLSQALKAQG DINLTALAGG VTANSLKATS SAGKISILAN KNLNLNSTQT TKAMPSADKD
cal-C11 VTAKEISNKD SIQLHANQVA LNSALTSQKN IDVSSEISDL VLNQALNAQG DINLTALAGG VTANSLKATS SAGKISILAN KNINLNSTQT SKAMPSADKD
pit-C7  VTAKEISNKD SIQLHANQIA LNSALTSQKN IDVSSEIADL VLSQALKAQG DINLTALAGG VTANSLKATS SAGKISILAN KNISLNSTQT TKAMPSADKD
bau-C1  VTAKEISNKD SIQLHANQIA LNSALTSQKN IDVSSEIADL VLSQALKAQG DINLTALAGG VTANSLKATS SAGKISILAN KNINLNSTQT SKAMPSADKD
pit-A4  VTAKEISNKD SIQLHANQIA LNSALTSQKN IDVSSEIADL VLSQALKAQG DINLTALAGG VTANSLKATS SAGEISILAN KNISLNSTQT TKAMPSADKD
pit-A5  VTAKEISNKG SIQLHANQIA LNSALTSQKN IDVSSEIADL VLSQALKAQG DINLTALAGG VTANSLKATS SAGKISILAN KNINLNSTQT SKAMPSADKD
pit-C6  VTAKEISNKD SIQLHANQIA LNSALTSQKN IDVSSEIADL VLSQALKAQG DINLTALAGG VTANSLKATS SAGKISILAN KNINLNSTQT SKAMPSADKD
bau-C2  VTAKEISNKD SIQLHANQIA LNSALTSQKN IDVSSEIADL VLSQALKAQG DINLTALAGG VTANSLKATS SAGKISILAN KNINLNSTQT SKAMPSADKD
nos-C8  VTAKEISNKD SIQLHANQIA LNSALTSQKN IDVSSEIADL VLSQALKAQG DINLTALAGG VAANSLKATS SAGKISILAN KNINLNSTQT SKAVPATDKD
pit-B7  LTATGLSSKD SIQLQGAQLS LNSGLTSLNN ISLTSEIADL VLSNVLNAQN NIDVSALTGS ITTNSLQATS TGGKISLLAK KDLIINSVQT --VLPKWPTD
bau-B4  LSSQGFSSKG GIQLQADQLT LSTALTSQNN ISLTAEIADL VLDNALTAQN DINVTALAGN LKTFSLKANS IAGKISLLAN KDVNLSSIQH --FDSMASYD
nos-C9  LKGSGLSSTG DLKLQAKALN IGTDLNAGKN LSLNATQSDL NLNKTLTAQN NIDVTSLDGK ITATGLNATS LQGKLSILGA KDTLFTNAGT T---------
bau-B1  LSSQGFSSKG GIQLQADQLT LNTALTSQNN ISLTAEIADL VLDNALTAQN DINVTALAGN LKTFSLKANS IAGKISLLAN KDVNLSSMQH FDSM--ASYD
pit-B5  ISAQGVSSKD SIQLQAGQLT LNTNFTSQKD INLITETTDL FLNNVLNAQN DINISALTGG ITANSLKANS NAGKISILAN KNLVLNSLQE IESAPWADQD
bau-B3  ISAQGVSNKD SIQLRAGQLT LNTNLTSQKD INVIAETTDL FLNNGLTAQN DINISALTGG ITANSLKANS NAGKISILAN KNLVLNSLQE IESAPWADQD
bau-B2  LRT------- ---------- -DTSLLTKKD IDVQTDI--- ---------- --------GN LYAKSLNVSS SEGKVSILGN GNVNLET--- -------QND
pit-B6  LRT------- ---------- -DTSLLTKKD IDVQTDI--- ---------- --------GN LYAKSLNVSS SEGKVSILGN GNVNFET--- -------QND
bau-A2  ILGQ------ ---------- ---------- ---------- -----GGLSA DNGISLYGGN VNVSSLNLNS NIGNTLVFAN NNIDIKGVNI KSNNGNLNFS
bau-A1  EGLK------ ---------- ---------- ---------- -----LNLDG DISFISTGSD IKANTLNLTS NVGGVYFKSN GDININSKIT EKKVLNSKGV
pit-A3  EGLK------ ---------- ---------- ---------- -----LNLDE DIGVVSTGGD IKANTLNLTS NLGSVYFKSN GNIKIDSKIT NKKYSDSIGF

        701                                                                                                  800
cal-C10 ELTTDQSVIS G--LKGVTLG SIGDGTVN-L QSVQVNANQG DILVSSNNGI NLKANSDVSV KGDTGYTQIV N-NVLKGQTV SVENSKSDIK IQNTDLGSTV
bau-C4  ELATDQTVIS G--LKGVTLG SIGDGTVN-L QSVQVNANQG DILVSSNNGI NLKANSDVSV KGDTGYTKIV N-NVLKGQTV SIENSKSDIK IQNTDLGSTV
pit-C5  ELTTDQSVIS G--LKGVTLG SIGDGTVN-L QSVQVNANQG DILVSSNNGI NLKANSDVVV TGDTGRFKTV N-NVLKGQTV SIENSKSDIK IQNTDLSSTV
bau-C3  ELTTDQSVIS G--LKGVTLG SIGDGTVN-L QSVQVNASQG DILVSSNNGI NLKANNDVVV TGDTGRFKTV N-NILKGQTV SIENSKSDIK IQNTDLSSTV
cal-C11 ELTTDQTVIS G--LKGVTLG SIGDGTVN-L QSVQVNANQG DILVSSNNGI NLKANTDVVV TGDTGRFKTV N-NILKGQTV SIENSRSDIK IQNTDLGSTV
pit-C7  ELTTDQSVIS G--LKGVTLG SIGDGTVN-L QSVQVNANQG DILVSSNNGI NLKANSDVVV TGDTGRFKTV N-NVLKGQTV SIENSKSDIK IQNTDLSSTV
bau-C1  ELTTDQTVIS G--LKGVTLG SIGDGTVN-L QSVQVNASQG DILVSSNNGI NLKANNDVVV TGDTGRFKTV N-NVLKGQTV SIENSKSDIK IQNTDLGSTV
pit-A4  ELTTDQSVIS G--LKGVTLG SIGDGTVN-L QSVQVNANQG DILVSSNNGI NLKANSDVVV TGDTGRFKTV N-NVLKGQTV SIENSKSDIK IQNTDLSSTV
pit-A5  ELTTDQTVIS G--LKGVTLG SIGDGTVN-L QSVQVNANQG DILVSSNNGI NLKANNDVVV TANKGQVKTV N-NVLKGQTV SIENSKSDIK IQNTDLGSTV
pit-C6  ELTTDQTVIS G--LKGVTLG SIGDGTVN-L QSVQVNANQG DILVSSNNGI NLKANNDVVV TANKGQVKTV N-NVLKGQTV SIENSKSDIK VQNTDLGSTV
bau-C2  ELTTDQTVIS G--LKGVTLG SIGDGIVN-L QSVQVNASQG DILVSSNNGI NLKANNDVVV TGDTGRFKTV N-NVLKGQTV SIENSKSDIK IQNTDLSSTV
nos-C8  ELTTDQIVIS G--LKGVTLG SIGDGTVN-L QSVQVNANQG DILVSSNNGI NLKANTDVIV TGNNGQFKTV N-NVLKGQIV SIENSKSDIK IQNTDLSSTV
pit-B7  ELTTVVTHIK G--QKGIDLG SIGDGGVS-I ISSGLEAQQG DVQIISGNGL NILPNYDVEV NADDWYERSI S-STLTAQNI GINNKKGDLR IEDATLTATF
bau-B4  VITTDKTNIN G--AKGVVVG STGEGALS-I SATDIMANQG DVQLISNNGL KILANNDVSM HNDDNGRSVL SPTTITAQNI NINNKKNDLV VDNGTSFIAA
nos-C9  -----NTVLK G--SKGVTIG TTGIGDLT-V QNAILESQSG NIVATSG--- ---------- ---------- ---------- ---------- ----------
bau-B1  VITTNKTNIN G--AKGVVVG STGEGALS-I SATDIMANQG DVQLISNNGL KILANNDVSM HNDDNGRSVL SPTTITAQNI NINNKKNDLV VDNGTSFIAA
pit-B5  GATTDMTFIN G--NKGVTLG SIGDGNVQ-L KSTRITAEQG DIQLVAGNGV SLQANTDVTI -SGDSGYDNV RKNVLQGQSL QIQNKKADIV IDSTNLTTSV
bau-B3  GATTDMTFIN G--NKGVTLG SIGDGNVQ-L KSTRITAEQG DIQLVAGNGI SLQANTDVTI -RGDHGYDDI RKNTLQGQSL QIQNKKADVV IDNTTLTTSV
bau-B2  GWYTLKNRIN A--KNGIILG SKGENAITKI NTVDLKSTDG NVLLLSGGDL TLDGNNGYT- ---TGMKAAV ASGFINAKDV TLWSKTGVLD ISSGVINASN
pit-B6  GWYTLKNRIN A--KNGIVLG SKGENAITKI NTVDLKSTDG NVLLLSGGDL TLDGNNGYT- ---TGMKAAV ASGFINAKDV ILWSKTGVLD ISSGVINASN
```

```
bau-A2  AENGYIKIAD SNLSSEKNKL FLISKDKQIF NNTSLLSG-G DLILNT---- -ISNKGDVEI NQDGGLFSSP IKSNNGNVII DSKGMLNITT KDLNKFVDIS
bau-A1  ETGAYFQNQEVS ENYINAFNDI SIDSNIGVYL NGANFKTNFG DFVVKG---- -KDLNIDVSK YGYHTRFDYM -----GQELY SYKNSNNKNN FEANNILFLV
pit-A3  GGAYFDQEVS ENYINSQKDL SIYSKNNIYI NGADFKSNSG DILIGS---- -RSVDFDAYR YDSAR----L -----GGGIL GKKN-NNTNR FDANNISILA

        801                                                                                                  900
cal-C10 GKLAINSRAG MSTIIDSVLT SKGNTELYAK DLLTLQGVNA TSDQHLAVSS GRTVYSNAEY TPAT---KWI ADKVTNLTSK GVTSVTATGN QVLQNTNLTG
bau-C4  GKLAINSRAG MSTIIDSVLT SKGNTELFAK DLLTLQGVNA TSDQHLAVSS GRTVYSNAEY TPAT---KWI ANKVTNLTSK GVTSVTATGN QVLQNTNLTG
pit-C5  GKLAINSRAG MSTIIDSVLT SKGNTELYAK DLLTLQGVNA TSDQHLAVSS GRTVYSNAEY TPAT---KWI ADKVTNLTSK GVTSVTATGN QVLQNTNLTG
bau-C3  GKLAINSRAG MSTIIDSVLT SKGNTELYAK DLLTLQGVNA TSDQHLAVSS GRTVYSNAEY TPAT---KWI ADKVTNLTSK GVTSVTATGN QVLQNTNLTG
cal-C11 GKLAINSRAG MSTIIDSVLT SKGNTELYAK DLLTLQGVNA TSDQHLAVSS GRTVYSNAEY TPAT---KWI ADKVTNLTSK GVTSVTATGN QVLQNTNLTG
pit-C7  GKLAINSRAG MSTIIDSVLT SKGNTELYAK DLLTLQGVNA TSDQHLAVSS GRTVYSNAEY TPAT---KWI ADKVTNLTSK GVTSVTATGN QVLQNTNLTG
bau-C1  GKLAINSRAG MSTIIDSVLT SKGNTELYAK DLLTLQGVNA TSDQHLAVSS GRTVYSNAEY TPAT---KWI ANKVTNLTSK GVTSVTATGN QVLQNTNLTG
pit-A4  GKLAINSRAG MSTIIDSVLT SKGNTELYAK DLLTLQGVNA TSDQHLAVSS GRTVYSNAEY TPAT---KWI ADKVTNLTSK GVTSVTATGN QVLQNTNLTG
pit-A5  GKLAVNSRAG MSTIIDSVLT SKGNTELYAK DLLTLQGVNA TSDQHLAVSS GRTVYSNAEY TPAT---KWI ADKVTNLTSK GVTSVTATGN QVLQNTNLTG
pit-C6  GKLAVNSRAG MSTIIDSVLT SKGNTELYAK DLLTLQGVNA TSDQHLAVSS GRTVYSNAEY TPAT---KWI ADKVTNLTSK GVTSVTATGN QVLQNTNLTG
bau-C2  GKLAINSRAG MSTIIDSVLT SKGNTELYAK DLLTLQGVNA TSDQHLAVSS GRTVYSNAEY TPAT---KWI ADKVTNLTSK GVTSVTATGN QVLQNTNLTG
nos-C8  GKLAINSRAG MSMITDSVLT SKGNTELYAK DLLTLQGVNA TSDQHLAVSS GLSVYSNREN TPSP---LLI ADKVTNLTSK GVTSVTATGN QVLKNTNLTG
pit-B7  GDLSINSE-G KSSIKAVSLN SKGNTELSAK DHLTMHAVTA NADKHMALSS KKNIYLNMGY GSTTTPVILD SYSSVNLKAN GILSIASAG- HAAYRTNYTG
bau-B4  NELKINSE-G KLNFKNSILN SKGNTEISSK DHLTLLGVTA NSDKHLALSS KKNIYINGEY GTTT---VWT PYVESNLTAK GILSMTSGGS HAAQNTTYTG
nos-C9  ---------A KNTLTDSTLT AKNNIEIFAT DDLTLDGVKS TSQQHTALNS KKNIYINS-H VNAYGVADFS STKSSELNST GVLSLVGGGS LNVQNTKLTG
bau-B1  NELKINSE-G KLNFKNSILN SKGNTEISSK DHLTLLGVTA NSDKHLALSS KKNIYINGEY G---TTTVWT PYVESNLTAK GILSMTSGGS HAAQNTTYTG
pit-B5  GGLSINGE-G KTTLSNSELN SKGNIELSAK DHLTLQNIEA NADKHMALNS QKNIYLNSEQ GPTVTSTIWE SYSLVNLKAA GLLSLKSKGS QALNSTSIKG
bau-B3  GGLSINGE-G KTTLSNSELN SKGNIELSAK DHLTLRNIEA NADKHMAVSS KKNIYFNSKY GSTATPVVWD SSSSVNLKAN GILSMV-GMD HIANQTTYTG
bau-B2  GGISIRAG-N NAQVHDIDLN STKNIEINSD KDLILERTNT RANQHIALSS KGNINAYQNY I--------- ------LDAK GVLSAISNGS IDGQGY---G
pit-B6  GGISIRAG-N NAQVHDIDLN STKNIEINSD KDLILERTNT RANQHIALSS KGNINAYQNY I--------- ------LDAK GVLSAISSGS IDGQGY---G
bau-A2  GE-------- KIEISSSNNM KLDGINVYSN GNLSLHA--- DKDIYLNTVL PNSWT-GGGF GSEIIRSQKH IS-ISSKEGS VKIGLENQAL NAPVVGLKAN
bau-A1  DN-------- SLRAVSGKYI AKKNINLFSN GSFDFYVNDL NSNGHTNIQA KNKLNIRDGF NGKNFFDNQY ISGSNNFKSD GLISILGSRV EVDGFLNAKG
pit-A3  ED-------- RLKTVAGNYN AKNNINLFSN GDVDFYTGYI NSGGHTNIQA KNKLIIRGGI GGENFFNNKY ISGVNSFKSD GLILILGDSV ELDGFLNARG

        901                                                                                                  1000
cal-C10 GAVLLEAGGF IL--GQTGLN L------NAV GSDLLKNDTK LNSLNGDLTI QTNSNLTIDP ----KVYSLK AVGDIELVSK TGTLTL---K GYEGTKGNGS
bau-C4  GAVLLEAGGF IT--GQTGLN L------NAV GSDLLKNDTK LNSLNGDLTI QTNSNLTIDP ----KVYSLK AVGDIELVSK AGTLTL---K GYEGAKGNGS
pit-C5  GAVLLEAGGF IL--GQTGLN L------NAV GSDLLKNDTK LNSLNGDLTI QTNSNLTIDP ----KVYSLK AVGDIELVSK NGTLTL---K GYGGTAGNGS
bau-C3  GAVLLEAGGF IA--GQTGLN L------NAV GSDLLKNDTK LNSLNGDLTI QTNSNLTIDP ----KVYSLK AVGDIELVSK TGTLTL---K GYEGAKGNGS
cal-C11 GAVLLEAGGF IL--GQTGLN L------NAV GSDLLKNDTK LNSLNGDLTI QTNSNLTIDP ----KAYSLK AVGDIELVSK NGTLTL---K GYGGTIGNGS
pit-C7  GAVLLEAGGF IL--GQTGLN L------NAV GSDLLKNDTK LNSLNGDLTI QTNSNLTIDP ----KVYSLK AVGDIELVSK TGTLTL---K GYGGTTGNGS
bau-C1  GAVLLEAGGF IL--GQTGLN L------NAV GSDLLKNDTK LNSLNGDLTI QTNSNLTIDP ----KVYSLK AVGDIELVSK TGTLTL---K GYGGTAGNGS
pit-A4  GAVLLEAGGF IL--GQTGLN L------NAV GSDLLKNDTK LNSLNGDLTI QTNSNLTIDP ----KVYSLK AVGDIELVSK NGTLTL---K GYGGTTGNGS
pit-A5  GAVLLEAGGF IL--GQTGLN L------NAV GSDLLKNDTK LNSLNGDLTI QTNSNLTIDP ----KVYSLK AVGDIELVSK TGTLTL---K GYGGTTGNGS
pit-C6  GAVLLEAGGF IL--GQTGLN L------NAV GSDLLKNHTK LNSLNGDLTI QTNSNLTIDP ----KVYSLK AVGDIELVSK TGTLTL---K GYGGTAGNGS
bau-C2  GAVLLEAGGF IL--GQTGLN L------NAV GSDLLKNDTK LNSLNGDLTI QTNSNLTIDP ----KAYSLK AVGDIELVSK TGTLTL---K GYGGTAGNGS
nos-C8  GAVLLEAGGF IT--GQTGLN L------NAV GSDLLKNDTK LNNLNGNLSI QSNSNLTIDP ----KAYNLK AVGDIELVSK AGTLTL---K GYGGTAGNGS
pit-B7  GAVSLEANNL IS-PSDGTLS F------NTV DSTFLKNDPV LKDLNGDLTI QTNGALTIDP ----KIHKFN AIGDIELISK NGTLTL---K GYEGIAGNGS
bau-B4  GAVLLEAGNV LTTPSNATLT F------NTV DSTFLKNDPV LKDLNGDLTI QTNGALTIDP ----KIHKFN AIGDIELISK NGTLTL---K GYEGTSGNGS
nos-C9  GAVLIEGDAL NT---AKSVD L------NAT GSDLLKNDTK LNNLNGNLSI QTNSNLTIDP ----KIHNLK AAGDIELISK NGTLTL---K GYEGTAGNGS
bau-B1  GAISLEAGNV LTTPSNATLT L------NAV DSAFLRSDPA LKDLNGDLTI QTNGALTIDP ----KIHTLN AKGDVQLISK NGALNL---N GYGGKNGNGS
pit-B5  GAINIETDGS FNINKDVQLN SARNIFSNQE SDKFLASDPQ LQKIQGNLSI QTNNTLVLDP ----EKIKIQ GDNNIELISK NGDLTL---L GYGGSKGNGS
bau-B3  GAISLEANNL IS-PSAGTLT F------NAM DSSVLTNDPA LKNLEGDLTI QTNGALTLDP ----KIHKLG AAGDYELISK NGKLTL---L GYGGTNGNFS
bau-B2  GAVIVEANQL ----SNNGID F------RAT GSELLQLDTK LKNINGNLSI QLNKDFVIKP THGHDTITLV AENDIDVRSK QGAIRI---E GENFAPNLNE
pit-B6  GAVIVEANQL ----SNNGID F------RAT GSELLQLDTK LKNINGNLSI QLDKDFVIKP THGHDTITLV AENDIDVRSK QGAIRI---E GENFAPNLNE
bau-A2  GVISIKSKLD QS-YQNTSLN A----GAITL HSD---AGSI NNSKNFRAIA NTTFFLESD- -----SELKK INGNLSLYAK KDIQLDAINS QGRAFDPNYT
bau-A1  GAILINSLEN YS-NSDNGLH L----FLTST KNDFLENNSL LKNHNGSIDI RSTGGVDFK- -----TNKIE AYGDINLYSK GGVTLN---- KIIDFSKDDV
pit-A3  GAILINSLKN YN-N--NGVN L----ILTSS KSDFLEGISI LQKYNGSIDI LSKENLNFS- -----WNNIK ADGDINLYSK GGMVFN---- RGANYSGEEA

    1001                                                                                                     1100
cal-C10 EQVVKLDTAN GGINLEGAK- VDIQGSQLIA K-------KD IQVVSTKDDL LIDGVRNSFS NIKFPDKAN- REKIVQADFI QELNALKATQ EYLNYQQALK
bau-C4  EQVVKLDTAN GGINLEGTK- VDIQGSQLIA K-------KD IQVVSTKDDL LIDGVRNSFS NIKFPDKAN- REKIVQTDFI QELNVLKATQ EYLNYQQALK
pit-C5  EQVVKLDTAN GGINLEGAK- VDIQGSQLTA Q-------KD IKIVSSKDDV LVDGVKNSFA KRKKEEKFT- EANNLKNSIL SSLDLLKQQQ FFTDYDNMVT
bau-C3  EQVVKLDTAN GGINLEGAK- VDIQGSQLTA Q-------KD IKIVGTKDDV LISGIKNTLN KQSSTKYLN- NLEIVKTDLQ KENDKLAADP EYI---QLLD
cal-C11 EQVVKLDTAN GGINLEGAK- VDIQGSQLIA Q-------KD IKIVSSKDDV LVDGVKNSLE NIRSLKYKE- DFIKELYSLR AVLEKEEINL VNKPRYNDYK
pit-C7  EQFVKLDTAN GGINLEGAK- VDIQGSQLTA Q-------KD IKIVSSKEDV LIDGLKNNIT NKISISKLE- YSKKELENIN NIIQQFKDTE NFKLYKNSID
bau-C1  EQVVKLDTAN GGINLEGAK- VDIQGSQLTA Q-------KD IKIVSSKDEV LIDGIKNSLK EVVSLKYTK- ELNDELKDLN NSLIKEESEL INKARYNDYK
pit-A4  EQVVKLDTAN GGINLEGAK- VDIQGSQLTA Q-------KD IKIVSSKDDV LIDGVKNNFS SLQKLNLAK- DYTLKIEEMN KERLKLQSQD YFNDYSNLVK
pit-A5  EQVVKLDTAN GGINLEGAK- VDIQGSQLTA Q-------KD IKIVSSKDDV LIDGMKNSFI NRKKQEKLN- EANNLKKNIS DSLALLKSQQ FFTDYDNMVT
pit-C6  EQVVKLDTAN GGINLEGAK- VELQGSNLKA A-------KD ISIVSTNGDL VVDGVRNTIT NQH----TD- EYKAKIIKLD EEIKILQKS- --SDYEAIIA
bau-C2  EQVVNLDTAN GSINLEGAK- VDIQGSQLTA Q-------KD IKIVSSKDDV LIDGVKNKFS GLQKLNLAQ- DYSLELDRLN KERLKLQSQE YFNDFSNMQK
nos-C8  EQVVKLDTAN GGINLEGAK- VDIQGSQLTA Q-------KD IKIVSSKDDV LVDGIKNTLN NISADSKNIL NIEKNIKEID NSINILSNSK EFQERNTLID
pit-B7  EQIVKLNTVG GGISLEGTK- IDLQGSQLTA Q-------KD IKLISSKEDI LIDGIKNKFT NIASIKHIA- SLNQNLADLN AKINIIKNDP TYLSTKDKIA
bau-B4  EQVVKLNTLG GGISLEGTK- IDLQGAQLTA Q-------KD IKLISSKEDI FIDGIKNKFT NFASIKHIV- SLNQNLADLN AKINIIKNDP SYLSTKDKIA
nos-C9  EQVVKLDTAN GSINLEGNK- VDIQGSQLIA Q-------KD IKIVSSKDDL LVDGIKNILN NRTADTKNII NIENNIKEID NNINILSNSK EFQERKILLD
bau-B1  EQVVKIQTTD GNINLIGTK- VHINGAKLQA SSSNAFIPRD INITATDGDV VISGVKNTAV DYVTQKRIQE IGIELSALDA QIERYKQDQA NYKAQT--FE
pit-B5  ERNFTISGT- GDIHVEGKK- VDIQGAFISG --------RN INIFSSSGDI NIDGIKNNFN NYASKKYSSE LFEKIFNL-T MILSNTESLK KYSASR--IS
bau-B3  ETVFRLTNNM GGSHLEGNKG VEIQGTVIRN M-------KD LKIIAPDGDI KIEGVKNTFN NSTYSKHSDY LFKQRLYYEN KIQEINRLGV LNTAQKKLVA
bau-B2  AGFVGIISRK GGLSLEGTS- VDIKGTKINV Q-------KD INIVSTKGDL VIDGIADKVN GVSKKKDLI- ---------- ---------- ----------
pit-B6  AGFVGIISRK GGLSLEGTS- VDIKGTKINV Q-------KD INIVSTKGDL VIDGIADKVN GVSKKKDLI- ---------- ---------- ----------
bau-A2  RMIKPVLSSQ GLMDIRADG- V----FQVNG K-------AP PAQTAMWDQL KMDRAYFTSN DGINILAG-- -TVKMYAGDL KNTSNTAPIN IISTGDIVLD
bau-A1  KNNKEISSEN GSINISADY- VDLNAARLKS K-------NG ISIVANKGDI SLNSKKETLN S-INVMGK-- -FTESYGDPY SEFELFKKNN --PEYKKKLD
pit-A3  KTSKDISSEN GVINISADY- IDLNAVRLKS K-------NG ISIVANKGDI GFNSKKVTLD D-VNVMGK-- -FTETYGDPY SEFELFKKNN --PEYKKKLD

        1101                                                                                                 1200
cal-C10 DANDQIK--- ---------- -AGEDW---- ---------- ----MRTAQG ---SIALTAK RLKDEGERKL ---------- ---------- -----AQVNE
bau-C4  DANDQIK--- ---------- -AGEDW---- ---------- ----MRTAQG ---SIALTAK RLKDEGERKL ---------- ---------- -----AQVNE
pit-C5  AYEDYAS--- ---------- -SNYDYTLYV KHYNLYKDFI SKYPVRPNPR ---LLIFGSD VPLFHSIIAV ---------- ---------- -----PQRSS
bau-C3  ILKKRLH--- ---------- -SGNELGFNV SE-------- ---AKKLAES ---LERKYLI KIEISGTTNG ---------- ---------- -----PMLPQ
cal-C11 NELDEILK-- ---------- GRKKIEEMMK YSL--TRSVG QKLLQESINK ---QNQLEEK YPEI------ ---------- ---------- -----NGLSN
pit-C7  NINNSINS-- ---------- KRKEWERAVS DDLHSKKRAL SDQLKKLEEE ---REDIRNK FKDI------ ---------- ---------- -----IDQEN
bau-C1  SELDSILE-- ---------- KRKKIEQMMK LPF--TKGVG QKLLDEITIM ---QNNLEEK YPEI------ ---------- ---------- -----NGPSN
pit-A4  KYRNYNDMF- ---------- LKYWDYELLD QTKNKNSEIW VNAYKEYERD ---FNVFKDK YPIKINYPVP GQLPFLL--- ---------- -----GGNNT
pit-A5  AYEDYTS--- ---------- -SNYDYSLYM KHYNLYNDFI SKYPVRPNPR ---LLIFGSD KPLFHSI--- ----FSE--- ---------- -----PQRMT
pit-C6  FFMKEALKV- ---------- YARISSGELI EGSPEMDREL QKALQKTTKE ---FPEYVSK YGP------- ---------- ---------- -----LRDQK
bau-C2  KYQYYNQVI- ---------- NKYWDRSTLD QSKNENAVLW VNAYNDYHKN ---YNEFKNK YMVKINYPIL GEPPILI--- ---------- -----NLMDGS
nos-C8  NIKKTKNSLE AALSYLFDGS NGEYSAGALS NVDVSNIFFY ENAYIQSFSS ---EGA-SDT YAMVINNDIV GFLPSVDSIG TLLSSYKAEI KKYKNLNESE
pit-B7  SLGSQASQIN QLLNDALSAY TSGVDPLNAS IGYDSENGNY IYVSNDDGGR ---AYFVSIP SGLDLSRYRQ IVLNEL---- ---------- -----PAENQ
bau-B4  SLGSQASQIN QLLNDALSAY TSGVDPLNAS IGYDSENGNY IYVSNDDGGR ---AYFVSIP SGLDLSRYRQ IILNEL---- ---------- -----PAENQ
nos-C9  NIKKAKNNLE ASMSYLFNAS NGEYGAGALS NVDVSNTLFY ENAYIQSISS EGASDTYIIV IPTGSYGNIQ GFLPSLGYVE VLLNSYKTEI KKYKILNESK
bau-B1  SMVQQRN--- ---------- ---------- ---------- ---------- ---------- ---------- ---------- ---------- ------IMSQ
pit-B5  QMQEELA--- ---------- ---------- ---------- ---------- ---------- ---------- ---------- ---------- ------DLKD
bau-B3  QYTEEIN--- ---------- ---------- ---------- ---------- ---------- ---------- ---------- ---------- ------DITK
bau-B2  ---------- ---------- ---------- ---------- ---------- ---------- ---------- ---------- ---------- ----------
pit-B6  ---------- ---------- ---------- ---------- ---------- ---------- ---------- ---------- ---------- ----------
bau-A2  AMNYDVNI-- ---------- -GQLDLMLPA QKLRDELEAS GKKDSVTLNT INQLNEEIAF YLSRSLNGT- ---------- ---------- -----RSQSS
```

```
bau-A1  DLNYHIYL-- ---------- -TRYGVSGKG AGQKRIDYAN NEINNAEKEI VNLNNTYGVT NLYNRYKLL- ---------- ---------- -----FSEYA
pit-A3  DLNYHIYL-- ---------- -TRWGVSGKG AGQKRIDYAN SEINNAEKEI VNLNNAYGVT NLYNRYKIL- ---------- ---------- -----FSEYA

        1201                                                                                              1300
cal-C10 TYKTLIANLN GVQRNIDQS- GANINFYEQS LKGQQHASVN IKSSGGNINL VSAKGLSLSG SKVN-AQQGE VQIEAAGTLT NNIYNIQGQY DSATENSVKQ
bau-C4  TYKTLITNLN GLQRNIDQS- GANISFYEQN LKGQQHASVN IKSIDGNINL VSAKGLSLSG SKVN-AQQGE VRIEAAGTLT NNIYNIQGQY DSATENSVKQ
pit-C5  RFDALYSFEP KYDENYLRSL QAEVDFYNTE INGSEHAEAK LTSKSGNINI TSAKGLSISG GNIS-AQLGQ VNLEASGVLA E-----QYKS TTTTETNPQP
bau-C3  LGAWVLGKIT ILYPQEINDI DKAITLYSQS LTGYEHAQPI LRSNNKDINI AAAKGVSISG AMID-STKGE VNIEAMGTLN GETYNIQGIY NSDKENSVKQ
cal-C11 KIKLS----- ------IQDI EDNLKYFEEE VNGSEHSEVN FSALSGNINI TSAKGLSISG GNIS-AQSGQ VNLEASGVLA E-----QYKS SISSGVNQPP
pit-C7  KLNNL----- ------LDER NNYFIYFDSN VSGSEHSEAK IISKLGNVNI TSAKGLSISG GNIS-AQSGQ VNLEASGVLA E-----QYKS SISSGANQPP
bau-C1  KLKVI----- ------IKDV EANISFFKNS INGSEHTETK LTSKSGNINI TSAKGLSISG GNIS-AQTGQ VNLEASGVLA E-----QYKS TTTTETNSQP
pit-A4  PLAVDYVFGF KYDNDYINDV EKNELFYKES LNGSEHAETK LTSKSGNINI TSAKGLSISG GNIS-AQLGQ VNLEASGVLA E-----QYKS SISSGVNQPP
pit-A5  RFDALYSFEP KYDENYLRSL QAEVDFYNTG INGSEHAEAK LNSKSGNINI TSVKGLSISG GNIS-AQTGQ VNLEASGVLA E-----QYKS TINNGANQPP
pit-C6  DFYSEMI--- ---------- ---KLFNVGY VNGEVHSGSK IISNTGNIQL VSNKGLSISG SSMD-AKDGK VNLEAAGALD GEGYNIQGKY NNPTENSVKK
bau-C2  PFSADYVFSF KYDDNYLNDV KRNELFYKSN LNGSEHSETK LNSKNGNINV TSAKGLSISG GNIS-AQLGQ VNLEASGILA E-----QYKS TTITESNPQP
nos-C8  KVKDLQKLIS KLESDKQSEI KSKELLTKG- VNGSEHTEAK LTSKSGNINI TSAKGLSISG GDIS-AQSGQ VNLEASAALS E-----QYKS TTTTNTNPQP
pit-B7  KLNNFNNKIT AFEQDKAR-V NQTITFMNNK TNGYEHAKST LTSKAGNISL TAARGISISG ANLT-ATAGQ ANIEARAPLV S-----QYTS STINSGQTLP
bau-B4  KLNNFNNKIT VFEQDKAR-V NQTISFMNNK TNGYEHAEST LTSKAGNISL TAARGISISG ANLT-ATAGQ ANIEARSPLL S-----QYTS STINSGQTLP
nos-C9  AIKDLESMIN KLQSQKQIEL KTKE-FLASN INGNEHAEAN LISKSGNINI TSAKGLTISG GNIS-SQLGQ VNLEANGALS E-----QYKT TTTIGTNAQP
bau-B1  GLEML----- ---KTKGKAI EHSYAEILPW TKAAAIGTGT PYWAKGNVNI LSNGGIKLEG TQVD-GLSVL IRAKGLLSQD PD----VGNK TEILPNSVVI
pit-B5  ASTFL----- ---ENGTNGF EHAETVL--- --RTSLGDGI -------INL ISKKGIKITG ATIQAGYDGK VNIEAQGGSD K-----IYNS TVLNKNNQPI
bau-B3  QINFL----- ---SLGTNGA EHAGSLL--- --RSETGK-- -------VNI VSGKGIMITG ADIM-AIDGQ VNIEAQGIGE K-----IYTS TALDKNSQPM
bau-B2  ---------- -----NSQDD QEKKNFIANT ITGVENFNSE LSTNTGNINI SSKKGVSITG ANID-AKQGI VNIQAQGVLN G-----KYRA TAKKEGTTA-
pit-B6  ---------- -----NSQDD QEKKNFIANT ITGVENFNSE LSSNAGNINI SSKKGVSITG ANID-AKQGI VNIQAQGVLN G-----KYRA TAKKEGTAA-
bau-A2  HIDSAQNINL LSKKGILIRA SELYAKNEVN IEAQGLLARD LTG-AQVTDY IDTSILVDGV HDIY--KNGE ATNSNY---- ------EERS DFHNSIVSGD
bau-A1  VILGKKRFNY YFED--FKQV NENIDYSDLN FSGYLHRGVD LLS-DGNINL ISNNGIILNS ANVN--TLGD FT-VYA---- ------KGKL DKKKTAELVE
pit-A3  VILGKKRFNY YFED--FKQV NENIDYSDLN FSGYLHRGGD LQS-DGNINL ISNNGIILNS ANVN--TLGD FT-VYA---- ------KGKL DKKKTAELVE

        1301                                                                                              1400
cal-C10 GAIAGSIIID ANQDSYEFGN ESEATYAWRS PVDAPSIYGK KGVKIKATGT NTTDNLILQG VDIRSE-GDV NIEAHKNIIF DVAVESGYDK STTTETKKKW
bau-C4  GAIASSIIID ANQDSYEFGN ETDTTYAWRS PVDAPSIYGK KGVKIKATGT NTTDNLVLQG VDIRSE-GDV NIEAHKNIIF DVAVESGYDK STTTETKKKW
pit-C5  RILNASIIVD GHTDFYDKGS ENDQNYSMRT LVSPSIINGD KGVNIRTVGK TKDDNLVLQA TGITSKNGDV KIESNKSILF DAAIEQSYDR SITTEKKKSW
bau-C3  GTIQSSIIID ALQDSYEFAK PTDTNYSWRS PVNPTEINGY KGVKIKATGQ QKTDNLVLQG VRIDSA-NSV NIEANKNIIF DVAIDNSYDK SEQTETQKKW
cal-C11 KSLNASVIID GHTDFYEKGT EGEGNYSFRT LVSPTIINGD KGVNIRTVGK TKDDNLVLQA IGITSKNGDV KIESNKSILF DAAIEQSYDL TTTTEKKKSW
pit-C7  KSLNASIIID GHTDFYSKGT EGEGNYSFRT LVSPTIINGD KGVNIRTVGK TKDDNLVLQA TGITSKNGDV KIESNKSILF DAAIEQSYDL TTTTEKKKSW
bau-C1  RILNTSIIID GHTDFYDKGN ENDDNYSMRT LVSPTIINGD KGVNIRTVGK TKDDNLVLQA TGITSKNSDV KIESNKSILF DAAVEQSYDR TITTEKKKSW
pit-A4  KSLNASIIVD GHTDFYEKGT ESEGNYSFRT LVSPTIINGD KGVNIRTVGK TKDDNLVLQA TGITSKNGDV KIESNKSILF DAAIEQSYDL TTTTEKKKSW
pit-A5  KSLNASIIID GHTDFYEKGT ESEGNYSFRT LVSPTIINGD KGVNIRTVGK TKDDNLVLQA TGITSKNGDV KIESNKSILF DAAIEQSYDL TTTTEKKKSW
pit-C6  GVIKGSIIID ANQDSYEQGS PSDNDYRWFS PVSPTTINGT KGVSIKVIGK QSTDNLILQG VAITSEN-DV NIEAYKNIIF DVAINNSFNK SQKTETKSKW
bau-C2  RILNASIIID GHTDFYDKGN ENDDNYSMRT LVSPTIINGD KGVNIRTVGK TKDDNLVLQA TGITSKNGDV KIESNKSILF DAAVEQSYDR TTTTEKKKSW
nos-C8  RILNASIIID GHTDFYDKGS ENDQNYSMRT LVSPSIINGD KGVNIRTVGK SKDDNLVLQA TGITSKNGDV KIESNKSILF DAAVEQSYDR SITTEKKKSW
pit-B7  RTLSASIIID GHTDFYDKGN EADANYSMRT FISPTIINGT KGVNIKTIGN TASDNLVLQA TGITASQGDV KIEANKSMLF DAAVEQSYDR STSTTTKKSW
bau-B4  RTISASIIID GHTDFYDKGN EADTNYSMRT FISPTIINGT KGVNIKTVGN TASDNLVLQA TGITASQGDV KIEANKSMLF DAAVEQSYDR STSTTTKKSW
nos-C9  RILNASVIID GHVNFYDKGN ENDQNYSMRT LISPTIINGD KGVSIRTVGK SQSDNLVLQA TGVTSKNGDV KIESNKSILF DAAIEQSYDR SLTTEKKKSW
bau-B1  QKSPTSIYIA GLLDEFEYGV AGANNYGYAS FNTPSYINGS KSVKIETLGG NNGLTIVSSS LN-GGIDG-V NLAASGDLRL EATQEELYKY STKTYKTGNW
pit-B5  NMN-ASIIIA GHNDFYDKGN DTDSNYKAIS FVSPTTIEGA -DINIKTVGN SAGDNLVLQG VGISAKNQ-V KIEAKKNILF DVALEQIFDR STKVQKKKSW
bau-B3  SMN-AGVILA AHNDFYDRGN ETDQNYQAIS LLTPTLVEGK RGVNIKTTGA QEGSNLILQA VGISSSEGDV KIESNKNILF DVALEQIYDR STSIEKKKSW
bau-B2  KELSASIIID GNLDVYEKGN ETDQNYSYRE YVNPTNINGN KGVNIRAIGK TVNDNLVLQG VGINSAQGDV NIEADKSILF DTALETLYDR STKTETKRSW
pit-B6  KEISASIIID GNLDVYEKGK DTDQNYSYRE YVNPTNINGN KGVNIRAIGK TVNDNLVLQG VGINSAQGDV NIEADKSILF DTALETLYDR STKTETKRSW
bau-A2  NGINIKSTGS AKLNWYDNPN LLSNY-ATVT SAKPIQLK-K K------IND GKN-NIVFNG AELISSGGDV NIQSNANIVL ESSQDEIYKH STSQVIKKTW
bau-A1  NQLDSSIVFA NVKDQYELGR DNENYYQKID ITKPNNIN-A KNVNL-IAND QSGLNLILQG TD-IKAADTV NIVGSGNIDF RHNVDNIYTK TQQTTTKESL
pit-A3  NQLDSSIVFA NVKDQYELGR DNENYYQKID ITKPNNIN-A KNVNL-IAND QSGLNLIIQG TD-IKAADTI NIVGSGNIDF RHNVDNIYTK TQQTTTKESL

        1401                                                                                              1500
cal-C10 YG-KKTTTTT VNTSNKSEGV SVNINAKNIS IKSKEQNTKE MTGKNR---- TSIDMYSSQF TATGGKISIQ AGGDLNFLTA LDVEQNTDDI TKKSSFLGV-
bau-C4  YG-KKTTTTT VNTSNKSEGV SVNINAKNIN IKSKEQNTKE MTGKNR---- TSIDMYSSQF TATGGKISIQ AGGDLNFLTA LDVEQNTDDI TKKSSFLGV-
pit-C5  GGLKKKYITT VSENNGTNAA SVDISAKNIS IETKKLDPSV DAKTPD---- NNIDIYSGRF TAEGGTISIN SGGNLNLYTV EESSRSDVDI TKKSSFAGI-
bau-C3  YG-KKTRITT VKTSNKSLGI SVDIVAKDIN IRSEGKNTAE MKGKDR---- TSIDMYSSNL TANGGKVTIQ AGGDLNFLTA DDVRQETTDI TKKKTWVGI-
cal-C11 GGLKKKYITT VSENNDTNAA SVDISAKNIS IETKKLDPSV DAKTPD---- NNIDIYSGRF TAEGGTISIK SGGNLNFYTV EESSSSTVDV TKKTSYSKLI
pit-C7  GGLKKKYITT VSENNDANAA SVDISAKNIS IETKKLDPSV DAKTPD---- NNIDIYSGRF TAEGGTISIK SGGNLNFYTV EESSSSTVDV TKKTSYSKLI
bau-C1  GGLKKKYITT VAESNDTNAA SVDISGKNIF IETKKLDPSV DAKNPD---- NNIDIYSGRF TADGGKITIN SGGNLNFYTV EESSDSNVDI TKKSSFAGI-
pit-A4  GGLKKKYITT VSENNDTNAA SVDISAKNIS IETKKLDPSV DAKTPD---- NNIDIYSGRF TAEGGTISIK SGGNLNFYTV EESSSSAVDV TKKTSYSKLI
pit-A5  GGLKKKYITT VSENNDTNAA SVDISAKNIS IETKKLDTSV NVKTPD---- NNIDIYSGRF TAIDGGTISIK SGGNLNFYTV EESSSSTVDV TKKTSYSKLI
pit-C6  YG-KKTKITT TQTDEKSGGL SVDIDAKNIN IKSQAKNTPA MSGQNR---- TSIDMYSSQL TAIDGKINIQ AGGDLNFLTA DDVSLNTTDI SKKSSFLGV-
bau-C2  GGLKKKYITT VAENNDTNAA SVDISGKNIF IETKKLDPSV DVKNPD---- NNIDIYSGRF TADGGKITIN SGGNLNFYTV EESSDSNVDI TKKSTFAGI-
nos-C8  GGLKKKYITT VAESNSTNAA SVDISGKNIF IETKKLDPSV NVSTPD---- NNIDIYSGRF TAEGGQIKIN SGGNLNFYTV EESSSSNVDI TKKSSFAGI-
pit-B7  GGLKKKITTT IEETNITNAA SVDISGKNIY IETKKLATDV DPTKPD---- NNIDIYSGRF TAEGGQISII SGGNLNFYTA EETSSSKVDI TKKSSFAGI-
bau-B4  GGLKKKITTT IEETNITNAA SVDIAGKNIY IETKKLAAGV DPTKPD---- NNIDIYSGRF TAEGGQISIK SGGNLNFYTA EETSSSKVDI TKKSSFAGI-
nos-C9  GGLKKKYITT IAESNNANAA SVDISGKNIF IETKKLDPSL N--TPD---- NNIDIYSGRF TADGGQISIN SGGNLNFYSV EESSSSNVDI TKKSSFAGI-
bau-B1  YKRKTVTETE TYNTNTPTPT SI-ISNRDIK ISSN------ ---------- GSLTLVSTDL QSHDGNINLN AGGYLRLWTK PTSQFSQIDV SKKSKLFGV-
pit-B5  GGLKKKTITT VMENELIRPA SVDIDAKNIY IESKEVN--- ----PQ---- NSIDIYGGKL TANGGMISVR SGGNLNFYTA PESSKTTIDV NSKSSIMGI-
bau-B3  GGLKKKTITT FSENESTRPA SVDISGKNIY IESKELN--- ----KN---- NKIDIYSGQF IAEGGDVFIR SGGDLFFNTV NEISKNQLEV NKKSSILGI-
bau-B2  YG-KKKTTTT TTIGDVVTPA SVDIYGKNIN VISQFKNPLR ADGTRDPALN TSVDIYSARF NANGGKISIR SGGDLNFLTV AAVNNSNVNQ VKKSSWLGI-
pit-B6  YG-KKKTTTT TTIGDVVTPA SVDIYGKNIN VISQFKNPLR ADGTRDPALN TSVDIYSARF NANGGKISIR SGGDLNFLTV AAVNNSNVNQ VKKSSWLGI-
bau-A2  YGKKKSTETY TQSQYGTAVP TTILAKN-IS LKA-A----- ---------- GDIDFYSTLM KANSGNVNI- TGENLYFLTS NNYANSTSTT KKNSSILGI-
bau-A1  FGLKKSSTTE TRILEGVIGK SDVLKANKIN IESLG----- ---------- GSISLYGTQI QAPKDQVKLK AAKDIYMVTF QDQINEVNTI KKDAGFLGL-
pit-A3  FGLKKSSTTE TKILEGVIGK SDVLKANKIN IESLG----- ---------- GNISLYGIQI QAPKDQVKLK AAKDIYMITF QDQINEVNTI KKDAGFLGL-

        1501                                                                                              1600
cal-C10 ---KLNKSKT TATRNIKAEL PATLKADY-I GTKSGLDTRL KGPEFEYLSG -ATIEAG--- -----GVISI ESASNVVEQT LKRDKNSVVW QSMQDKGSIT
bau-C4  ---KLNKSKT TATRNIKAEL PATLKADY-I GTKSGLDTRL KGPEFEYLSG -ATIEAG--- -----GVISL ESASNIVEQT LKRDKNSVVW QSMQDKGSIT
pit-C5  ---KYNTSKT NATRTQVTEI PATLKADY-I GTKSVYDTRL VGTEFEYLIG -STLEAG--- -----GKLEL IAAKTSITDL LKKEKNSVVW QSMQDKGSIT
bau-C3  ---KYNDSKS TNTRDIKSEI PAVLTADY-I TTKSGFDTRL KGTEFNYLEG -ATIEAG--- -----GKIQL EGASSTVSET LKKEKNSVVW QSMQDKGSIT
cal-C11 SLLASSKTTT NTTRTQISEL PATLKADY-I GTKSGFDTKL VGTEFEYLKD -ATIETG--- -----GTLEL IAAKTSITEL LKKEKSSLAW QGMQDKGSIT
pit-C7  SLLASSKTTT NTTRTQISEL PATLKADY-I GTKSGFDTKL VGTEFEYLKD -ATIETG--- -----GTLEL IAAKTSITEL LKKEKSSLAW QAMQDKGSIT
bau-C1  ---KYNTSKT NATRTQVTEL PAILKADY-I GTKSGFDTRL VGTEFEYLKD -ATIEAG--- -----GKLDL IAAKTSITDL VKKEKNSVVW QSMQDKGSIT
pit-A4  SLLGSSKTTT NTTRTQISEL PANLKADY-I NTQAVDKTKL VGTEFDYLKN -ATIETG--- -----GTLEL IAAKTSITEL LKKEKSSLAW QSMQDKGSIT
pit-A5  SLLGSSKTTT NTTRTQISEL PANLKADY-I NTQAVDKTKL VGTEFDYLKN -ATIETG--- -----GTLEL IAAKTSITEL LKKEKSSLAW QSMQDKGSIT
pit-C6  ---KLNKSHT TNTRNVKSEL PAELNADY-I GTKSGFDTRL KGTVFNYLEG -AEIQAG--- -----GTITL ESASTTVTET LKKESNSVVW QSMQDKGSIT
bau-C2  ---KYNTSKT NATRTQVTEL PATLKADY-I GTKSGFDTRL VGTEFEYLKD -ATIEAG--- -----GKLDF IAAKTSITDL VKKEKNSVVW QSMQDKGSIT
nos-C8  ---KYNTSKT NATRTQVSEL PATLKADY-I GTKSGFDTRL VGTEFEYLKD -ATIEAG--- -----GKLEL IAAKTSITDL VKKEKNSVVW QSMQDKGSIT
pit-B7  ---KYNDSKT NATRTQISEL PAVLKADY-I GTKSGFDTKL VGTEFEYLKG -AHIEAG--- -----GKIEL LAATKQITDL LKKEKNSVVW QSMQDKGSIT
bau-B4  ---KYNDSKT NATRTQISEL PAVLKADY-I GTKSGFDTRL VGTEFEYLKG -AQIEAG--- -----GKIEL LAATKQITDL VKKEKNSVVW QSMQDKGSIT
nos-C9  ---KYNTSKT NATRTQVSEL PATLKADY-I GTKSGFDTRL VGTEFEYLKG -ASIEAG--- -----GKLEL IAAKTSITDL VKKEKNSVVW QSMQDKGSIT
bau-B1  ---KYNTSTV SSTRSVESEL PAVLVAQRNT NLSSGNTTWI RGSEFENKLG HAEIKAGVGE NAVEGAQIIL QDIVTKITET KKRDSNSVVW QSMQDKGSIT
pit-B5  ---KYNSSKT NSTHNVVTQI PTKLVADY-I GTKSGFDTRL VGTDFEYLKG -ATIESG--- -----GKIFI EPAITNISTK LKKESNSVVW QSMQDKGSIT
bau-B3  ---KYNTSKT NSSYQVVTQV PSTLTASY-I GTKSGFDTHL AGTEFNYLKE -ATIESG--- -----GKIFL EPSVTSINNT IKKEKNSVVW QSMQDKGSIT
bau-B2  ---NLGTSKT NATRNQTTEI PAKLKADY-I GTKSGYDTRL EGTEFTYLKS -ATIEAG--- -----GTITL VPAKNLVEES LNRKKNSVVW QSMQDKGSIT
pit-B6  ---NLGTSKT NATRNQTTEI PAKLKADY-I GTKSGYDTRL EGTEFTYLKS -ATIEAG--- -----GTITL VPAKNLVEES LNRKKNSVVW QSMQDKGSIT
bau-A2  ---PLNKSKT TSSRSQISQL PVKLVGDY-L STESKNDTVF VGTEFDYMKN -ASVKAG--- -----GQIAF LGASETISQA LKVEKSNIVW QSMQDKGSIT
bau-A1  ---EYRDSNS VGSKIVNNQI PTVITGRY-I ASSSGGSTYL EGTQFNFTEG -VSIKVGDGP NPSEDAKLVI NSAKGLYSNS QDNNKKNTFW QSMESRGTVE
pit-A3  ---EYRDSNS VGSKIVNNQI PTVITGRY-I ASSSGGSTYL EGTQFNFTEG -VSIKVGDGP NPSEDAKLVI NSAKGLYSNS QDNNKKNTFW QSMESKGTVE
```

```
        1601                                                                                                 1700
cal-C10 ETAKLPSFNG PVLPTFKATG GLSVQVPISE KDANKVELRD EILKLANHPG NTYLKELVNR KD--VDWQTV LLTQKDWDYK SQGLTAAGAA IIVIIVTIVT
bau-C4  ETAQLPSFNG PVLPTFKAAG GLSVQVPISE KDVNKVELRD EILKLANQPG NAYLKELVNR KD--VDWQKV LLAQKDWDYK SQGLTAAGAA LIVIIVTIAT
pit-C5  ETAQLPSFNG PVLPTFKAAG GLSVQVPISE KDVNKVELRD AILDLSKKPG NEYLKELVNR KD--VDWQTV LLTQKDWDYK SQGLTAAGAA IIVIIVTILT
bau-C3  ETATLPSFNG PALPTFKAAG GLSVQVPISE KDANKVELRD EILKLANQPG NAYLKELVNR KD--VDWQQV LLTQKDWDYK SQGLTAAGAA LIVIIVTIAT
cal-C11 ETAQLPSFNG PVLPTFKAAG GLSIQVPISE KDANKIELRD AILELSKQPG NEYLKEFVNR KD--VDWQMV LLTQKDWDYK SQGLTGAGAA IIAIVVTMVT
pit-C7  ETAQLPSFNG PVLPTFKAAG GLSVQVPISE KDANKIELRD AILELSKQPG NEYLKEFVNR KD--VDWQMV LLTQKDWDYK SQGLTGAGAA IIAIVVTMVT
bau-C1  ETAQLPSFNG PVLPTFKAAG GLSVQVPISE KDANKVELRD EILKLANQPG NAYLKELVNR KD--VDWQTV LLTQKDWDYK SQGLTGAGAA IIAIIVAVLT
pit-A4  ETAQLPSFNG PVLPTFKAAG GLSVQVPISE KDANKVELRD AILDLSKKPG NEYLKEFVNR KD--VDWQIV LLTQKDWDYK SQGLTGAGAA IIAIVVTMVT
pit-A5  ETAQLPSFNG PVLPTFKATG GLSVQVPISE KDANKVELRD AILDLAKQPG NEYLKEFVNR KD--VDWQKV LLTQKDWDYK QQGLTAAGAA IIVIIITIVT
pit-C6  ETAQLPSFNG PVLPTFIAAG GLSVQVPISE KDVNKVELRN EILKLANQPG NAYLKELVNR KD--VDWQTV LLTQKDWDYK SQGLTGAGAA IIVIIVTIVT
bau-C2  ETAQLPSFNG PVLPTFKAAG GLSVQVPISE KDANKVELRD EILKLANQPG NAYLKELVNR KD--VDWQTV LLTQKDWDYK SQGLTAAGAA LIVIIVTIAT
nos-C8  ETAQLPSFNG PVLPTFKAAG GLSVQVPISE KDANKVELRD EILKLANQPG NAYLKELVNR KD--VDWQQI LLTQKDWDYK SQGLTGAGAA IIVIIVTIVT
pit-B7  ETAKLPSFNG PVAPTFKAAG GLLVQVPISE VDANKVELRD EILKLANQPG NAYLKSFVNR ND--VNWQQI LLAQKEWDYK SQGLTGAGAA IIAIIVAIAT
bau-B4  ETAKLPSFNG PVAPTFKAAG GLVVQVPISE IDANKVELRN EILKLANQPG NAYLKDFINR ND--VNWQQI LLAQKDWDYK SQGLTGAGAA IIAIIVAIAT
nos-C9  ETAQLPRFNG PVLPTINAAG GLSVQVPISE TDANKAELRD EILKLANQPG NAYLKELVNR KD--VDWQQI LLTQKDWDYK SQGLTAAGAA LIVIIVTIAT
bau-B1  ETAKLPSFNG PTPPIFKATG GLSVQIPISE KDANKIVLRD EILKLANQSG NTYLKDLVNR ND--VDWQKV LLTQKDWDYK SQGLTGAGAA IIAIIVVAAT
pit-B5  ETAKLPNFNG PVAPTFKAAG GLSVQIPISE KDANKIVLRD EILKLANQPG NTYLKDLVNR ND--VDWQKV LLTQKDWDYK SQGLTGAGAA LIVIIVTIAT
bau-B3  ETAKLPSFNG PVAPIFKAAG GLAVQVPVKD D------VRS ALDTLVNQQG TTYLKDLVLR ND--VDWQAV KLAQQQWDYK SQGLTPAAAA IIVIIITVAT
bau-B2  ETAQLPNFNG PQPPVFNAAG GLSVQVPISE KDVNKVELRD EILKLANQPG SAYLKDLVNR KD--IDWQTV LLTQKDWDYK SQGLTAAGAA IIVIIVTIAT
pit-B6  ETAQLPNFNG PQPPVFNAAG GLSVQVPISE KDVNKVELRD AILKLANQPG SAYLKDLVNR KD--VDWQTV LLTQKDWDYK SQGLTAAGAA IIVIIVTIAT
bau-A2  ETTKLPNFNG PTPPTFKASG GLIVQVPIIS NKNN--DIRT EVINLANQPG NEYLKGFIAR KD--VNWEAV KLAQENWDYK SQGLTAAGAA IIVIIVTIAT
bau-A1  ELANLPKFNG PVN-NIKIDG NLSVQVPIKQ GENS--EVID IINNLSKEKG YEYLKTLIDN KDKNVNWTAL ELTKKNWDYK QQGLTPAGAA ILTIIVTALT
pit-A3  ELANLPKFNG PVN-NIKIDG NLSVQVPIKQ GENS--EVID IINNLSKEKG YEYLKTLIDN KDKNVNWTAL ELTKKNWDYK QQGLTPAGAA ILTIIVTALT

        1701                                                                                                 1800
cal-C10 MGSGTAAAA- ------GAAG GTAASGTTVG LGASMIGSA- GVTTATVGGV TTITGVSTLG AMANAAITSL ATQASVGLIN NGGDIGKTLK DLGSKDSIKN
bau-C4  MGSGTAAAA- ------GAAG GTAASGTTVG LGASMIGSA- GVTTATVGGV TTITGVSTLG AMANAAITSL ATQASVGLIN NGGDIGKTLK DLGSKDSVKN
pit-C5  MGSGTAAAA- ------GAAG GTAASGTTVG LGASMIGSA- GVTTATVGGV TTITGVSTLG AMANAAITSL ATQASVGLIN NGGDIGKTLK DLGSKDSVKN
bau-C3  MGSGTAAAA- ------GAAG GTAASGTTVG LGASMIGSA- GITTTAAGAI VP----STLG AMANAAITSL ATQASVSLIN NGGNIGKTLK ELGSKDAVKS
cal-C11 AGAGAAVVG- ---------- ---------- ALGGSAATL- GGSAIA---- ---------- -MTQAALTSL ATQASVSLIN NGGNIGKTLK ELGSKDSIKN
pit-C7  AGAGAAVVG- ---------- ---------- ALGGSAATL- GGSAIA---- ---------- -MTQAALTSL ATQASVSLIN NGGNIGKTLK ELGSKDSIKN
bau-C1  YGAGTALIGT TAAAGAGTTT AAVGTTTVAA AGGGTITTF- GGMTLATTTA AGVTTYTTTG IMVNAALTSI ATQASIGLVN NQGNVTQTLK DLGSKDTVRN
pit-A4  AGAG-AAVV- ------GALG GSAAT----- LGGSAI---- ---------- ---------- AMTQAALTSL ATQASVSLIN NGGNIGKTLK ELGSKDSVKN
pit-A5  MGTGTAAAA- ------GAAG GTAASGTTVG LGASMIGSA- GVTTATVGGV TTITGVSTLG AMANAAITSL ATQAGVSLIN NGGDIGKTLK DLGSKDSIKS
pit-C6  MGSGTAAAA- ------GAAG GAAASGTTVG LGASMLGAA- GVTTTAAGAI VP----SALG AMANAAITSL AAQASVGLIN NGGDIGKTLK DLGSKDSIKN
bau-C2  MGSGTAAAA- ------GAAG GTAASGTTVG LGASMIGSA- GITTTAAGAI VP----STLG AMANAAITSL ATQASVGLIN NGGDIGKTLK DLGSKDSIKN
nos-C8  MGSGTAAAA- ------GAAG GAAASGTTVG LGASMLGAA- GVTTTAAGAI VP----STLG AMANAAVTSL ATQASVSLIN NGGNIGKTLK DLGSKDSIKN
pit-B7  YGAGVAALGT TTVAASGGAT TVTVGTTTVA VGAGGSATAF GGTVLATTTA AGVTTYTTTG IMINAALTSL ASQASVSLVN NQGNVAQTLK DLGSKDTVKN
bau-B4  YGAGVAALGT TTVAASSGAT TVTVGTTTVA VGAGGSATAF GGTVLATTTA AGVTTYTTTG IMINAALTSL ASQASVSLVN NQGDVVQTLK DLGSKDTVKN
nos-C9  MGSGTAAAA- ------GAAG GTAASGTTVG LGASMIGSA- GVTTATVGGV TTITGVSTLG AMANAAITSL ATQASVSLIN NGGDIGKTLK ELGSKDSAKN
bau-B1  AGAG-ATVV- ------GALG GTAST----- LGGSAV---- ---------- ---------- AMSQAAITTI ASQASISLIN NGGDIGKTLN DLGSKESIRN
pit-B5  MGTGTAAAA- ------GAAG GTAASGTTVG LGASMLGTAG VTTTAAGAIV P-----STLG AMANAAVTSL AAQASIGLIN NGGDIGKTLK EMGSKDSIKN
bau-B3  YGYGAQGAG- ------AALF QTSSA----- ---------- ---------- -------TSS AMANAAVASM ASQAAISLAN NGGDPLKALK SLGSSEYIKS
bau-B2  MGSGTAAAA- ------GAAG GTAASGTTVG LGASMLGTA- GVTTTAAGAI VP----STLG AMANAAVTSL AAQASIGLIN NGGDIGKTLK EMGSKDSIKN
pit-B6  MGSGTAAAA- ------GAAG GTAASGTTVG LGASMLGTA- GVTTTAAGAI VP----STLG VMANAAVTSL AAQASIGLIN NGGDIGKTLK EMGSKDSIKN
bau-A2  MGSGTAAAA- ------GAAG GTAASGTTVG LGASMIGTA- GVTTTAAGAI VP----STLG VMANAAVTSL ATQASISLIN NGGDISKTLK DLGSKETVKN
bau-A1  AGAGSSAA-- ---------- ---------- -GALGFSTT- GVASS----- ---------- -MAQAAITSL STQASISLIN NGGNVAATLR ELGSDQSIRN
pit-A3  AGAGSSAA-- ---------- ---------- -GALGFSTT- GVASS----- ---------- -MAQAAITSL STQVSISLIN NGGNVAATLR ELGSDQSIRN

        1801                                                                                                 1900
cal-C10 LAASVVTAGL LNGVATNLNI SS-------- NAIVN---DI ANNMATGMIQ GVGSTLIDSA VNGRDLSEGL EKALLAGLAN SLQAPLAGAI GDT---LGMS
bau-C4  LAASVVTAGL LNGVATNLNI SS-------- NAIVN---DI ANNMATGMIQ GVGSTLIDSA VNGRDLSEGL EKALLAGLAN SLQAPLAGAI GDT---LGMS
pit-C5  LAASVVTAGL LNGVATNLNI SS-------- NAIVN---DI ANNMATGMIQ GVGSTLIDSA VNGRNLSEGL EKALLAGLAN SLQAPLAGAI GDT---LGMS
bau-C3  LATSVVTAGV LSQLSNTELM SDL-SKMTKQ GEFMK---EV VGRTTQGIIN AGASSLVTTA INGGSLSENL SNALLTNTAM ALQASLSGQI --K---LLES
cal-C11 LAASVVTAGL LSQVSTALNL KPDSTVFS-- DRLVN---NF TSSVGSTLVQ T----AIN-- --GGDLEENL KVALLSGLAG ALQGQMAQNI KGL---EDI-
pit-C7  LAASVVTAGL LSQVSTALNL KPDSTVFS-- DRLVN---NF TTSVSSTLVQ T----AIN-- --GGDLEENL KVALLSGLAG ALQGQMAQNI KGL---EDI-
bau-C1  LATAVVTAGL LDKVGGTATM QSLKALGNTP DVLMNLAAKT ATAIIETTIR T----GINST ITGESFSDGL EDLLYQSLGT AIQGTMANQI HLN---KEII
pit-A4  LAASVVTAGL LSQVSTALNL KP-DSTVL-S DRLIN---NF TTSVGSTLVQ --------TA IKGGDLEENL KVALLAGLAG ALQGELASQI GDS---LYKA
pit-A5  LATSVVTAGV LSKIASIDQM KALQETTK-S GEFFT---NL SGRVGQALVN STVSASVDTA VNGGSLSEKL SASLVAGLSV ALQGSLAGEI GEN---LDLG
pit-C6  LAASVVTAGL MSQVGSALNL KP-DSTLL-P DRLAN---NF ASSVGSTLVQ --------TA IKGGDLGDNL QVALLAGLAG ALQGELAQNL ---------K
bau-C2  LAASVVTAGL MSQVGSALNL KP-DSTLL-P DRLAN---NF ASSVGSTLVQ --------TA IKGGDLGDNL QVALLAGLAG ALQGELAQNL ---------K
nos-C8  LATSVVTAGL MSQVGSALNL KP-DSTML-P DRLIN---NI SSSVGSTLIQ --------TA IKGGDLEENL KVALLSGLAG ALQGELASQI GDS---LYKS
pit-B7  LATSVVTAGL LDKIGGSNWM QQFNGTGV-T DRIVT---NL VNSTGSALVS --------TA INGGDLNDNL QTALLGGLAT AVQGYLSNNI SG----IQQG
bau-B4  LATSVVTAGL LDKIGGSNWM QQFNGTGV-T DRIVT---NL VNSTGSALVS --------TA INGGDLNDNL QTALLGGLAT AVQGYLSNNI SG----IQQG
nos-C9  LAASVVTAGL MSQVGSSALNL NLNDMPRL-- EKLAN---NF VQGVGSNIIS --------SS IQGENLSDNL DSIILSGLTS ALHAEIAQGI GENFSPLLKD
bau-B1  LAASVVTAGL LSQVS-TAL- ----NLKPDS TLFP----DR LMNNFTTSVG STL---VQTA INGGNLGDNL QVALLAGLAG ALQGELASQI GTS---LDKV
pit-B5  LAASVVTAGL LSQVG-TAL- ----NLKPDS TLLS----DR LVNNFTTSVG STL---VQTA INGGNLGDNL QVALLAGLAG ALQGELASQI GTS---LDKV
bau-B3  LAVSVLSAGA LSAVGGTAMM QNLEKLKQSG DLVQ----KI VSNLTAQALVN STIGAGINTA VSGNSLSENL ENYLASGAMT ALQGTMAQYI -KP---LESQ
bau-B2  LAASVVTAGL LSQVGTALNL KPDSTLL--S DRLAN---NF ASSVGSTLVQ --------TA INGGDLGDNL QVALLAGLAG ALQGELASQI GTS---LDKV
pit-B6  LAASVVTAGL LSQVGTALNL KPDSTLL--S DRLAN---NF ASSVGSTLVQ --------TA INGGDLGDNL QVALLAGLAG ALQGELASQI GTS---LDKV
bau-A2  LATSVVTAGA LSTVGGLDWM EKLKDMGTDP NLLV----NL AGRTGGAIVE AGLSAGISSS INGGSLTDNL QGMLLSSFAT ALQGSLASKI GKE---LGGM
bau-A1  LVASIATAGA LKSISVQFNL PETSTAFND- ---------- --RLYVAFVN STSSSIINSA INGGSLSDNL EASLLAGLSN SLQAEFAQNI GLS---LDIK
pit-A3  LVASIATAGA LKSISVQFNL PETSTAFND- ---------- --RLYVAFVN STSSSIINSA INGGSLSDNL EASLLAGLSN SLQAEFAQNI GLS---LDIK

        1901                                                                                                  2003
cal-C10 SNAFVSKVVA IAAHAAAGC- ATGVIDKECQ SRALGAALGE VIAENM-FKP A-NGIEYTEA E-----KSKI LNITKLTTGV VAAYAGYDVT AAANAADTAVSNN
bau-C4  SNAFVSKVVA IAAHAAAGC- ATGVVDNECK SRALGAALGE VIAENM-FKP A-NGIEYTEA E-----KSKI LNITKLTTGV VAAYAGYNVT AAANAADIAVSNN
pit-C5  SNAFVSKVVA IAAHAAAGC- ATGVIDKECQ SRALGAALGE VIAENM-FKP A-NGIEYTEA E-----KSKI LNITKLTTGV VAAYVGYDVT AAANAADTAFSNN
bau-C3  NELDMDYVLH KVAHAAAGCV AASISRSECE SGAIGAAIGE IVAEHM-DKS I-DSKSFNND NDIQNYQQKV RDVSKLVASS VATLAGYDPN TAANSAEIAILNN
cal-C11 -----NYIAH KLAHALAGCI AGAIQG-QCQ AGAIGGAVGE IVAGFT--PP S-SGLLYTEE E-----KQKI LMVGQLTSGI IAAYAGYDVN VASNSANIALTNN
pit-C7  -----NYIAH KLAHALAGCI AGAIQG-QCQ AGAIGGAVGE IVADFI--PP S-SGLLYTEE E-----KQKI LMVGQLTSGV IAAYAGYDVN VASNSANIALTNN
bau-C1  SDGLMKNILS QVAHATAGCT GAAIGGGECI AGAIGAAVGE SVAEYM--KQ Y-SS------ D-----QKLI TNISNAMAVT IAAATGYDTA IAQQASETAVTNN
pit-A4  DPTVLQYTIH KIAHAAAGCA VAAATKQSCE AGAIGGAVGE IIAELMIPYG K-TALDLDDN E-----INRI KGVSKIVAGS ISAYAGYDVN TAANSADTAISNN
pit-A5  SKELSDQILH KLAHAAAGC- IASSLQEKCE AGAIGAVVGE MVAGSFDMEP E-NDGDYSQA Q-----LNKV KNVGQLISGV VAASAGYDVT VASQSASTAIENN
pit-C6  GLEDVNYILH KIAHAAVGCA AAAATKQSCE AGAIGAGVGE IIAQEM-LHG R-NPTFVSAE E-----KAKI EAYSKLIAGT ISAYAGYDVN TAANSATIAIQNN
bau-C2  GLEDVNYILH KIAHAAVGCA AAAATKQSCE AGAIGAGVGE IIAQEM-LHG R-NPTFVSAE E-----KAKI EAYSKLIAGT ISAYAGYDVN TAANSATIAIQNN
nos-C8  DPTILQYTIH KIAHAAAGCA AAAATKSSCE AGAIGAGIGE IVASLM-PKP I-NGIEYNDE E-----KVKI KNVGKVVAGV VSAYAGYDVN TAANSADIAIQNN
pit-B7  SNFDLDYILH KVAHAAAGCL AGSIQK-DCA AGAIGAALGE VFADTI---T G-NKYDYTDA Q-----LNAI KEGSKALAAT VAAYAGYDVN IAISSADTALTNN
bau-B4  SNLDLDYILH KVAHAAAGCL AGSIQK-DCA AGAIGAALGE VFADTI---T G-NKYDYTDA Q-----LKAI KEGSKALAAT VAAYAGYDVN IAISSADTALTNN
nos-C9  NPDIFDKVLH KIIHIAAGCA IGSIQK-QCE AGAIGAGVGE FVAEFMPAAS GNGVYSDDEK -------ERV LAVGKILAAG VAGITGYDVN VASSSANTALLNN
bau-B1  DPNIFEYTIH KIAHAAVGCA TAAATKSSCE AGAIGAGVGE IVAGLMPEPA --NGIEYTDA E-----KLKI RNIGKLVSGT VAAYTGYDVN TAANSADTAIQNN
pit-B5  DPNIFEYTIH KIAHAAVGCA AAAATKSSCE AGAIGAGVGE IVAGLMPEPA --NGIEYTDA E-----KLKI RNIGKLVSGT VAAYTGYDVN TAANSADTAIQNN
bau-B3  GFSI-EYVLH KIAHAAAGCG AAAATKQSCE AGAIGAGVSE VAAEYLRKPR VFESVDDALA Y-----KNQI VEVSKGIAGA VAIVTGYDSN TAMASADNALKNN
bau-B2  DPNIFEYTIH KIAHAVVGCA TAAATKQSCE AGAIGAGVGE IVAELMIPAG K-TALDLTDA E-----RTKI KDTSKLIAGT TAAFAGYDVN TAANSANIAVENN
pit-B6  DPNIFEYTIH KIAHAVVGCA TAAATKQSCE AGAIGAGVGE IVAELMIPAG K-TALDLTDA E-----RTKI KDTSKLIAGT TAAFAGYDVN TAANSANIAVENN
bau-A2  NKTDFENILH KIAHLAAGCV AGAIQK-QCE SGAIGAGIGE FVAEFMPAAS GNGVYSDDEK -------ERV LAVGKILAAG VAGITGYDVN VASSSANTALLNN
bau-A1  DPTVIDSLLH KIAHIASGCV TGAIQK-QCE AGAIGAGVGE IVAGMIEQPN KDATVEDISK Y-----QEKV INTSKLISGS LASLAGYDAG YAASSASLAVSNN
pit-A3  DPTVIDSLLH KIAHIASGCV TGAIQK-QCE AGAIGAGVGE IVAGMIEQPN KDATAEDISK Y-----QEKV INTSKLISGS LASLAGYDAG YAASSASLAVSNN
```

CT domains

```
            2004                                                                                              2104
cal-C10     YLSHAQEKQK SQDFKNCNVL TCGGVTVKWA AISAGQDANY AVGFAAGIPT SIAESVVGLA QMALSPIQTY KAIKEVINSE NALGKISDSI KQEYTNRINK
bau-C4      YLNHVQEKQK SQELKDCNVL TCGGVVVKWA AISAGQDANY AAGFAVGIPT SVAESVVGLA KMALSPIQTY KAIKEVINSE NALGNISDAI KQDYTNRINK
pit-C5      YLNHVQEKQK SQELKDCNVL TCGGVVVKWA AISAGQDANY AAGFAVGIPT SVAESVVGLA KMALSPIQTY KAIKEVINSE NALGNISDAI KQDYTNRINK
bau-C3      RQLNQSEIIK IRMLAAGNAN KEARYNIAAC ALVRCAEGYE GTTEYAFLKR IQDAGNSAEF KGERDILVKQ NFRTPIMGSM TTNQKLFEKS SSNFRELNGK
cal-C11     AFNEKKVKQL LEQAKGYMGE KGGQALDAVS RGIAKGDINA LKNAQTQIAE YLLKKADSGG LSQAEMVTFG TLYALNETLF PTNILDVIPG AGKAIGKGND
pit-C7      AFNEKKVKEL LDQAKGYMGE KGGQALDAVS RGIAKGDINA LKNAQTQIAE YLLKKADSGG LSQAEMVTFG TLYALNETLF PTNILDVIPG AGKAIGKGND
bau-C1      AFWVPLIAAV MIADRAYSAY QIYRDVQDVV DGKKSLEQLA IEKGTAYVER AILGNLTSKG LGIVSEGGKK VIKRVTYIAP EKEIAELFTK TNSRNTLTIA
pit-A4      SLPKLATSGA KIAKKVLDQI KKWPSNKPLE PNDVTKMFKD AGVQEIVDVA NNIKTLVSPT STWFEKISAA VDLAVGIDIK AGKDVALAMK DKHIIQLKDP
pit-A5      WGKVLSSTGK AAYKIFKKAT ELQKAGKNLN DTNVWKDILK DTATGEYQDM LNSFKTAFSA GSTPLERTWA LIDLATGIDK KDVKIVQDLL KDRNTIAKRL
pit-C6      AFKDKALLKL EATRKYLDDK SKAALDGLIN AYKKGDIEAA KKFKNQLDDS VTTWANSSSS YLNIETKSAL GEIVIVVSDL VIPTNYVEIL PVGKLSKVAK
bau-C2      AFKDKALLKL EATRKYLDDK SKAALDGLIN AYKKGDIEAA KKFKNQLDDS VTTWANSSSS YLNIETKSAL GEIVIVVSDL VIPTNYVEIL PVGKLSKVAK
nos-C8      YLTHYSKAKF VADLAKCKVT SSNCDTIIQN YQNLSKTNDL ELATVCNNTA RSRDCNNMLK AALDYAGNDF NQIYGINSPA MANLKADQIR SRELVLKNIF
pit-B7      WLGTQQKKQR ELEYIKANAL GDAAIFAKYG GISVLQDRLT QGGFAAGVAA GAGSDIWNVG KAILSPIDTL NGLKAIIADS DVRAQLTQSA ITAFNTKIQR
bau-B4      WLGTQQKRQR DLEYIKANAL GDVAIFAKYG GISVLQDRLT QGGFASGIAA GAGTDIWNIG KAILNPIDTL NGLKAILTDS EVRAQLAQST ITAFNTKIQR
nos-C9      AFVSAKVKLE GAKRSCTEVG FAYGSTGCKE FLSKKYEMQP ALRNALVQSK VSEFIFETLE DVTPIWGNIK AFRDAETKTD YFVAGITVFP IAKWVNSGKK
bau-B1      SLVRLATTTG KVLVKTLDKY NALRKTGKQV TKDDIVAS-L KEQSADELIG IADDLLTVFG RSTSPFDRAM AAIDLVVGTD LKPGKGESLK LAKSELDKLK
pit-B5      SLVRLATTTG KLLVKAAEIY GDIKKANKGI VDSKVFVQKL KDQGVSELVG IADDLVTVFG PASTPFDRAL AAFDLIIGTD LKPGKAESLK LAQAELDKLK
bau-B3      FVFLAAVPTA GEALVGLTLG AIRTCATNAA CVSRAAQLGI SVSATLAANS NSTDDKKVVK PVPNTSGSET GTPNNGDDQN DGKQAKKLSN NDIVDLTKNP
bau-B2      TLGKLATTAG KAAYRVAKRI TDMPASVRAN LKPDDVIKIF KEEGVQGVID IGDNIKTLIS PTSTPGDRAF AAIDLLIGID LKAGKNVDIL KSEKNIIGKN
pit-B6      TLGKLATTAG KAAYRVAKRI TDMPASVRAN LKPDDVIKIF KEEGVQGVID IGDNIKTLIS PTSTPGDRAF AAIDLLIGID LKAGKNVDIL KSEKNIIGKN
bau-A2      ALSKTDVDVL IQNLKSCKNE SQCRNEIQKA VLKSNYNQSN AVNCLSLLCS DNLEQAISGY NQLRGFIASG KFSGDNLKLL NNLLTQSTKD ITLQINRLPQ
bau-A1      YLTSKNYRDL KTNYDTCIKG GGSVGKCSGA MIAAAETISK TNDTRLRADC VNATSNACIN GIKDLFEGER TRNELNKVNK IFYNSEYSKF NSWPKFNSLS
pit-A3      YLTSKNYRDL KTNYDTCIKG GGSVGKCSGA MIAAAETISK TNDTRLRADC VNATSNACIN GIKDLFEGER TRNELNKVNK IFYNSEYSKF NSWPKFNSLS

cal-C10     LESEYQKAGI KGSYNAGLEA GKLTADVASL FAGGAGLAKG GVILTEKVAA KVGYKVLKDP IVISDKGIPV TLDGHKIYDP QFTPLSTNPR ALYRFSDFNH
bau-C4      LESEYEKAGI KGSYNAGLEA GKLTADVASL FAGGASIAKG GVILTEKVAA KVGYKVLKDP IIISDKGIPV TLDGHKIYDP QFTPLSTNPG ATYRFSDPTH
pit-C5      LESEYEKAGI KGSYNAGLEA GKLTADVASL FAGGASLAKG GVILTEKVAA KVGYKVLKDP IIISDKGIAV NLDGHKIYDP QFTPLSTNPR ALYRFSDFNH
bau-C3      YSILDRNLDY QITTRAGGAV MVVGGGVGLA GSTALGSTCV TGVGCLVALG GFVSSADLVM AGAKQAWSGK SIDTIGAQVI SATTGLSTSQ AEMIYGMLSM
cal-C11     LLKGIRAAAP ASAASATARL GLREDLAKLA NIPRNMEIQP ANIWGKTIQD IKYSFEMDGA VLTLSPPKSG TSGLAQVYSV KNSATGIKEF EYHPGGGEHS
pit-C7      LLKGIRAAAP ASAASATARL GLREDLAKLA NIPRNMEIQP ANIWGKTIQD IKYSFEMDGA VLTLVPPKSS TSGLAQVYSV KNSATGIKEF EYHPGGGTHS
bau-C1      GLDIKKGYNP VNSQTTVILD SQKLTSNNIK EYAQQLAGTV PLKAVGNGDK QIYVATLGNG QQIRLRSVSS SASQTRARWT IDIQGNPGIN NSLGLSPKEK
pit-A4      GPIHHICTNK NCLSTARGGP WTRQFQKYFD GAGLDINKSV ENLVALADHK GPHPKEYHAY VEKELRLAVS GKQANTAEYR NAVIATLSRI KSEALVSGSQ
pit-A5      PNIKNGQFFD DKKIEELANR GDVKKIHQID VGAKGGWAKQ ANGKLEANSA YLLPNGAAYV TDNSGNVKEV TANLRNILMD RNNYQQSIAG KSGISGDQGG
pit-C6      ILKIGEDGTK SAGRLAEELA ELQKVDIKFG KTLPGAKAPI TVTAESNIGG KHMFDTNQTA RPEVNRTNTP TLAAGNAKID PSNPNLTMKN AHAEIALIQR
bau-C2      ILKIGEDGTK SAGRLAEELA ELQKVDIKFG KTLPGAKAPI TVTAESNIGG KHMFDTNQTA RPEVNRTNTP TLAAGNAKID PSNPNLTMKN AHAEIALIQR
nos-C8      KDVNNWYGMI NDIDLRADFF SAMYAKTGAE WFRVANETSR AAIVGLGAEG AYSGATFAIG GVISSITSAG SVNLLDVYNW RSEAGYALMK AGYNNFKQLY
pit-B7      IENNYNTGGL KEATQLGYDL GTLTWEVVGI LTVVKGAATA TAKMGSAGLK ITTTIAETAK QSAKYQAPTT SIISAAQTQK VISDVKTIPV VTFNGVVLDP
bau-B4      IESGFNTGGI QTAIQVGYDL GTLAWEVAGI LTVVKGAATA TAKLGSAGLK FSSSIADAAK QSAKYQAPIT SIISTTQTQK IISEVKAIPV VTFNGVVLDT
nos-C9      AITLILKDGS EVEAIISRAG TGYSIYVPQV GKDIVEQMAK GKTVTSTTFK PGARQTINNI KGLDKSGNNW SVNLIGDGTK MNKAAQVTYV DVSPAGVKTT
bau-B1      IDRSYVQTVY QNRVDVVTRN RMNGKEFETS VASKLGVKRN TDRQMITIKT ERDGQINIIP DAFGNGGTLV EFKNVQYLTD TKQLRGYAAT GKPVSLVVNM
pit-B5      VDQAYTLKVL QVKKDVVTRN RLNGKEFEMS AASKLGVKRN TDRQMITIKT ERDGQINIIP DAFGNGGTLV EFKNVQYLTD TQQLRGYGAT GKPVTLVVNM
bau-B3      NWHKTSLKSR IAAAYARELR GSKNFDFYRD PKTGDIFIKG NQSNNMVKID ISRFLK*
bau-B2      YSAGLNREIL NKAGDFYPNV PDLRTGRTIS FPVGDLKKVP ELSRVSWGAQ ERGKFIQEWY KRGYETPRGG WSEYDIHHIK PREFGGTNEF WNLTPVQRKT
pit-B6      YSAGLNREIL NKAGDFYPNV PDLRTGRTIS FPVGDLKKVP ELSRVSWGAQ ERGKFIQEWY KRGYETPRGG WSEYDIHHIK PREFGGTNEF WNLTPVQRKT
bau-A2      VQAYLNSPWT PPTKQQMAQI VIDILPMTSD PSAIYSIITG KAVVTQEEVS RFYAAVGLIL PITIGRSLTK AEIAGLKQSS QNPLSGTVSI KDLPALPKSA
bau-A1      KADTNIRDLG SLYSTFEKID FWQKTNCNGI STAACQTKYK QYLDANEATR VQIAGGLDRV KSMPVIGNTV TVGDAVITLI TGTSLSTNDP SRLVALAEIA
pit-A3      KADTNIRDLG SLYSTFEKID FWQKTNCNGI STAACQTKYK QYLDANEATR VQIAGGLDRV KSMPVIGNTV TVGDAVITLI TGTSLSTNDP SRLVALAEIA

cal-C10     RSTGGDIYFG ENIATSYFEVRQAVNGKSLFV GQVEVKNMLD LTDPKILKQM GIDQKKLTEV APDGYSNVEK AKKEAIYAYT NRIANQAYDK GYTGIIYNSS
bau-C4      RKTGGDVYFG ENIATSYFEV RQAVNGKSLF VGQVEVKNML DLTDPKILKQ MGIDQKKLTQ IVNTS----- -TEQDFVYAY TNKIANQAYD KGYSGIIYNS
pit-C5      RSTGGDIYFG ENIATSYFEV RQAVNGKSLF VGQVEVKNML DLTDPKILKQ MGIDQKKLTE IAPDGYSNAE KAKKEAIYAY TNRIANQAYD KGYTGIIYNS
bau-C3      ASVTNSVRTG AKQAVQEQRV LINKPTITNC VNGTTCFVAG TLIETKNGLK PIEEFNGNEL VWSRNDLTLE YGYKPVIAKK ITHNQPIFEV VVQNALKLDK
cal-C11     KSGDMYYKIV MNDGTQFRII DPKSAFNPRT ITSNQIYLNP N-GQKLKYQN GKWEIWK*
pit-C7      SDGVRYYKIV MNDGTQFRII DPNKGFKPGT ITSNQIYLNP N-GQKLKYEN GKWEIWK*
bau-C1      IEIKFR*
pit-A4      VNKWLTKK*
pit-A5      HLIAASLGGS GDRINLVPMA KTLNNGSWKA MESELANAVK LGKDVNMKVA VGYPTGSSRP NKFIVTATIN GKSTNYQFSQ*
pit-C6      AYDAGLTKGE TMQVLVRGKE VCDHCGQVMK TMYERSGLAK LIIHDTTSGS TTTYYKVIDA KTNIATTKIV VSPIFGH*
bau-C2      AYDAGLTKGE TMQVLVRGKE VCDHCGQVMK TMYERSGLSK LIIHDTTSGT TTTYYKVIDA KTKIATTKIE VAPIFGH*
nos-C8      DGKVTDIVAW NISQMQQEQK LLQPIHEKYF KNSKLFVPQT WVPTKFPNNP VNLLDYNSRV KYGCIEMGYT IAQGCKP*
pit-B7      RLPAPVAGYA YKPKVIDSPN PNIANSHVNG YMGELRLANE VAAQPNQAVL KFGGDLTHHG SDIISVDVKT GGVYLWDSKY RSANSKLGAS TTFTNSTTRT
bau-B4      RLPVPAAGYT YKPKVLDSTN PNIANSHVNG YKAELRLANE VVAQPNQVVL KYGDAIGRNG SDIISVDIKT GNVYLWDSKY RSSNSKLGAS TTFTNSTTRT
nos-C9      KIANIPYDSR GLPIFDDIAK FTTTISAHKA ESTQFKYASL SLWEAIQKGQ VNSSQFTSEQ LKQIQAGASK IKGYTWHHNA QSGPNNMQLL PEVVHNAVKH
bau-B1      NTKISETVKN NILSNKGTIQ RFDPIKQTLH PY*
pit-B5      NTKISETVKN SILSNKGTIQ RFDPIKNTLH SY*
bau-B2      HQQEFNSFWR NM*
pit-B6      HQQEFNSFWR NM*
bau-A2      QNFVIEQTAQ SKYTKLLYEA KNDPSLGTWS TSKIGQKGEE IAVQLVKGSG FTDVISIQNA SGNGIDIIAK NPQGNYVYLE VKTSAVGKIG SLSKRQEDMN
bau-A1      SVGITRYIPQ VGPVVHVAQP KYAGQRYTTA SLTSNKDATI NPDTIRAALV NNRSWLKASY VPEKVSIAVA KVETKSGTDN ILAVSGPAWN GNAPTTGKNT
pit-A3      SVGITRYIPQ VGPVVHVAQP KYAGQRYTTA SLTSNKDATI NPDTIRAALV NNRSWLKASY VPEKVSIAVA KVETKSGTDN ILAVSGPAWN GNAPTTGKNT

cal-C10     -RNTGSNNNR AVVLFGGRYD AAKIKPVLDK PILKK*
bau-C4      SRNTGSSNNR AVILFGGRYD AEKIKPVLDK PILKK*
pit-C5      SRNTGSNNNR AVVLFGGRYD AAKIKPVLDK PILKK*
bau-C3      NAIDLDLSVV TRGQVKQIAK NGDPLGGERE TFKTTEEHPF WVKNIGWQKA SILSEGMTLL DRSNNELTVI SQKLLAKLDT VYNIEVKDYH TYHVGKLGVW
pit-B7      NAVNEALTTI RQSNLPESVK QVALKNLNDG TFTTYTVATG NSKNSVIFSC VNKVCK*
bau-B4      NAVNEALTTI RQSNLPESVK QVALKNLNDG TFTTYTVATG NSKNSVIFSC VNKVCK*
nos-C9      IGEDSLKKGV IIK*
bau-A2      FFVNDILTNA AAKTGRYVNI SDTVNAQAKI MLTKYKQQKA VGNIYGAAIG VDLKNAQILI SSWTNK*
bau-A1      DVVINGVKYK VVTTDSHSVQ PITYTVDKNG KPITNNNHAE AKLFSYIKDS YSSTPSKVTM SIQNTASEPA GMCNGCLFSSG KYFTNEVPFM RLSIFHG*
pit-A3      DVVINGVKYK VVTTDSHSVQ PITYTVDKNG KPITNNNHAE AKLFSYIKDS YSSTPSKVTM SIQNTASEPA GMCNGCLFSSG KYFTNEVPFM RLSIFHG*

bau-C3      VHNAQCCDLI YQNIVKETKY GVISNRVKTE ALFENVVKEQ GGKVLSGGKY GSNNGYDHVI IFKDAQGNTN LTMVVDSKQLG QKGIKLDPKAAGGNMQMS
bau-C3      SEWDRAVLAK LDPNSEAYKA VFAARQNGTL VKGAAYVDKS AEKLMLVRID PATKK*
```

Supplementary file 5. A) HET regions (blu residues)

**bau-A1**	GDISLNSKKE	TLNSINVMGKFTESYGDPYSEFELFKKNNPEYKKKLDDLNYHIYLTRYGVSGKGAGQKRIDYANNEINNAEKEIVNLNNTYGVTNLYNRYKLLFSEYAVILGKKRFNYYFEDFKQVNENIDYSDLNF	SGYLHRGVDLLSDG
**pit-A3**	GDIGFNSKKV	TLDDVNVMGKFTETYGDPYSEFELFKKNNPEYKKKLDDLNYHIYLTRWGVSGKGAGQKRIDYANSEINNAEKEIVNLNNAYGVTNLYNRYKILFSEYAVILGKKRFNYYFEDFKQVNENIDYSDLNF	SGYLHRGGDLQSDG
**bau-A2**	VKMYAGDLKN	TSNTAPINIISTGDIVLDAMNYDVNIGQLDLMLPAQKLRDELEASGKKDSVTLNTINQLNEEIAFYLSRSL	NGTRSQSSHIDSAQ
**pit-A4**	DDVLIDGVKN	NFSSLQKLNLAKDYTLKIEEMNKERLKLQSQDYFNDYSNLVKKYRNYNDMFLKYWDYELLDQTKNKNSEIWVNAYKEYERDFNVFKDKYPIKINYPVPGQLPFL–LGGNNTPLAVDYVFGFKYDNDYINDVEKNELFYKESL	NGSEHAETKLTSKS
**pit-A5**	DDVLIDGMKN	SFINRKKQEKLNEANNLKKNISDSLALLKSQQFFTDYDNMVTAYEDYTSSNYDYSLYMKHYNLYNDFISKYPVRPNPRLLIFGSDKPLFHSIFSEPQRMTRFDALYSFEPKYDENYLRSLQAEVDFYNTGI	NGSEHAEAKLNSKS
**bau-B1**	GDVVISGVKN	TAVDYVTQKRIQEIGIELSALDAQIERYKQDQANYKAQTFESMVQQRNIMSQGLEMLKTKGKAIEHSYAEILPWT	KAAAIGTGTPYWAK
**bau-B4**	EDIFIDGIKN	KFTNFASIKHIVSLNQNLADLNAKINIIKNDPSYLSTKDKIASLGSQASQINQLLNDALSAYTSGVDPLNASIGYDSENGNYIYVSNDDGGRAYFVSIPSGLDLSRYRQIILNELPAENQKLNNFNNKITVFEQDKARVNQTISFMNNKT	NGYEHAESTLTSKA
**pit-B7**	EDILIDGIKN	KFTNIASIKHIASLNQNLADLNAKINIIKNDPTYLSTKDKIASLGSQASQINQLLNDALSAYTSGVDPLNASIGYDSENGNYIYVSNDDGGRAYFVSIPSGLDLSRYRQIVLNELPAENQKLNNFNNKITAFEQDKARVNQTITFMNNKT	NGYEHAKSTLTSKA
**pit-B5**	GDINIDGIKN	NFNNYASKKYSSELFEKIFNLTMILSNTESLKKYSASRISQMQEELADLKDASTFLENGTNG	FEHAETVLRTSLGD
**bau-C1**	DEVLIDGIKN	SLKEVVSLKYTKELNDELKDLNNSLIKEESELINKARYNDYKSELDSILEKRKKIEQMMKLPFTKGVGQKLLDEITIMQNNLEEKYPEINGPSNKLKVIIKDVEANISFFKNSI	NGSEHTETKLTSKS
**bau-C3**	DDVLISGIKN	TLNKQSSTKYLNNLEIVKTDLQKENDKLAADPEYIQLLDILKKRLHSGNELGFNVSEAKKLAESLERKYLIKIEISGTTNGPMLPQLGAWVLGKITILYPQEINDIDKAITLYSQSL	TGYEHAQPILRSNN
**bau-C4**	DDLLIDGVRN	SFSNIKFPDKANREKIVQTDFIQELNVLKATQEYLNYQQALKDANDQIKAGEDWMRTAQGSIALTAKRLKDEGERKLAQVNETYKTLITNLNGLQRNIDQSGANISFYEQNL	KGQQHASVNIKSID
**cal-C10**	DDLLIDGVRN	SFSNIKFPDKANREKIVQADFIQELNALKATQEYLNYQQALKDANDQIKAGEDWMRTAQGSIALTAKRLKDEGERKLAQVNETYKTLIANLNGVQRNIDQSGANINFYEQSL	KGQQHASVNIKSSG
**pit-C5**	DDVLVDGVKN	SFAKRKKEEKFTEANNLKNSILSSLDLLKQQQFFTDYDNMVTAYEDYASSNYDYTLYVKHYNLYKDFISKYPVRPNPRLLIFGSDVPLFHSIIAVPQRSSRFDALYSFEPKYDENYLRSLQAEVDFYNTEI	NGSEHAEAKLTSKS
**pit-C7**	EDVLIDGLKN	NITNKISISK–LEYSKKELENINNIIQQFKDTENFKLYKNSIDNINNSINSKRKEWERAVSDDLHSKKRALSDQLKKLEEEREDIRNKFKDIIDQENKLNNLLDERNNYFIYFDSNV	SGSEHSEAKIISKL
**cal-C11**	DDVLVDGVKN	SLENIRSLKYKEDFIKELYSLRAVLEKEEINLVNKPRYNDYKNELDEILKGRKKIEEMMKYSLTRSVGQKLLQESINKQNQLEEKYPEINGLSNKIKLSIQDIEDNLKYFEEEV	NGSEHSEVNFSALS
**nos-C8**	DDVLVDGIKN	TLNNISADSKNILNIEKNIKEIDNSINILSNSKEFQERNTLIDNIKKTKNSLEAALSYLFDGSNGEYSAGALSNVDVSNIFFYENAYIQSFSSEGASDTYAMVINND––––IVGFLPSVDSIGTLLSSYKAEIKKYKNLNESEKVKDLQKLISKLESDKQSEIKSKELLTKGV	NGSEHTEAKLTSKS
**bau-C5**	DDVLIDGIKN	TLKNSSADATNIISIENNIKEIDKSINILSNSKEFQERKTLLDNIKKTKNSLEAAISYLFDGSNGEYSAGALSNVDVSNIFFYENAYIQSFSSEGASDTYAMVINND––––IVGFLPSVGSIGTLLSSYKAEIKKYKNLNESAKVQELEKLISKLEAEKQSEIKSKELLTTGV	SGSEHTEAKLTSKS
**nos-C9**	DDLLVDGIKN	ILNNRTADTKNIINIENNIKEIDNNINILSNSKEFQERKILLDNIKKAKNNLEASMSYLFNASNGEYGAGALSNVDVSNTLFYENAYIQSISSEGASDTYIIVIPTGSYGNIQGFLPSLGYVEVLLNSYKTEIKKYKILNESKAIKDLESMINKLQSQKQIELKTKEFLASNI	NGNEHAEANLISKS
**bau-B3**	GDIKIEGVKN	TFNNSTYSKHSDYLFKQRLYYENKIQEINRLGVLNTAQKKLVAQYTEEINDITKQINFLSLGT	NGAEHAGSLLRSET
**pit-B7**	EDILIDGIKN	KFTNIASIKHIASLNQNLADLNAKINIIKNDPTYLSTKDKIAKLNNFNNKITAFEQDKARVNQTITFMNNKT	NGYEHAKSTLTSKA
**bau-C2**	DDVLIDGVKN	KFSGLQKLNLAQDYSLELDRLNKERLKLQSQEYFNDFSNMQKKYQYYNQVINKYWDRSTLDQSKNENAVLWVNAYNDYHKNYNEFKNKYMVKINYPILGEPPILINLMDGSPFSADYVFSFKYDDNYLNDVKRNELFYKSNL	NGSEHSETKLNSKN
**bau-B2**	GDLVIDGIAD	KVNGVSKKKDLINSQDDQEKKNFIANTI	TGVENFNSELSTNT
**pit-B6**	GDLVIDGIAD	KVNGVSKKKDLINSQDDQEKKNFIANTI	TGVENFNSELSSNA
**pit-C6**	GDLVVDGVRN	TITNQHTDEYKAKIIKLDEEIKILQKSSDYEAIIAFFMKEALKVYARISSGELIEGSPEMDRELQKALQKTTKEFPEYVSKYGPLRDQKDFYSEMIKLFNVGYV	NGEVHSGSKIISNT
**pit-n1**	DDVLVDGVKN	TILDSTKITSFERLAAVTSEKIDLEIQHEKMRKDPDYLFLINELDKGRGLKYEDKVDVEKMYALRDALEKRYLVKIDIEAGAGRRGKKVFFIDAYEKYTDKISELDQSINFYNKKF	TGNEHRGSSFTSKT
**pit-n2**	DDVLIDGIKN	SLKNLQSKKYSQNSIDDFNNIKTTLESLKTSEEYQAYTKDIGTMKSMRGYAQLVEFSGNHDFAMHLIEESNKIEKNLSKYSTLLEAIELNEKSLNDAEILKKMFDSNV	EGSEHAEAKLNSKS

B) HET region replacement. HET changes in pit-A5 derivatives are highlighted

Strains: pit-A5/C8, ARLG1933; pit-A5, ABUH540; pit-A5/A4, A.sp.907131; pit-A5/Hn2, NBRC 110508;
pit-A5/Hn1, UKK-0546;pit-A5/C7, PR301;nos-C8, XH551; pit-A4, PR320;pit-A7,PHEA-2

```
            1                                                                                                          120
pit-A5/C8   MNKNSYRIIY SKARQMFVAV AENVRSQTKT SGQSEASTQN NIDNTESQAF HQLWQVKALV ASISLWMPLA PVYAGIVADS AANAANRAVI GAGKNSAGTV VPVVNIQTPK NGISHNIYKQ
pit-A5      MNKNSYRIIY SKARQMFVAV AENVRSQTKT SGQSEASTQN NIDNTESQAF HQLWQVKALV ASISLWMPLA PVYAGIVADS AANAANRAVI GAGKNSAGTV VPVVNIQTPK NGISHNIYKQ
pit-A5/A4   MNKNSYRIIY SKARQMFVAV AENVRSQTKT SGQSEASTQT NIDNTESQAF HQLWQVKALV ASISLWMPLA PVYAGIVADS AANAANRAVI GAGKNSAGTV VPVVNIQTPK NGISHNIYKQ
pit-A5/Hn2  MNKNSYRIIY SKARQMFVAV AENVRSQTKA SGQSEASAQS NIDNTESQAF HQLWQVKALV ASISLWMPLA PVYAGIVADS AANAANRAVI GAGKNSAGTV VPVVNIQTPK NGISHNIYKQ
pit-A5/Hn1  MNKNSYRIIY SKARQMFVAV AENVRSQTKT SGQSEASTQN NIDNTESQAF HQLWQVKALV ASISLWMPLA PVYAGIVADS AANAANRAVI GAGKNSAGTV VPVVNIQTPK NGISHNIYKQ
pit-A5/C7   MNKNSYRIIY SKARQMFVAV AENVRSQTKT SGQSEASTQN NIDNTESQAF HQLWQVKALV ASISLWMPLA PVYAGIVADS AANAANRAVI GAGKNSAGTV VPVVNIQTPK NGISHNIYKQ
nos-C8      MNKNSYRIIY SKARQMFVAV AENVRSQTKA SGQSEASTQN NIDNTESQAF HQLWQVKALV ASISLWMPLA PVYAGIVADS AANAANRAVI GADKNSAGTV VPVVNIQTPK NGISHNIYKQ
pit-A4      MNKNSYRIIY SKARQMFVAV AENVRSQTKT SGQSEASTQT NIDNTESQAF HQLWQVKALV ASISLWMPLA PVYAGIVADS AANAANRAVI GAGKNSAGTV VPVVNIQTPK NGISHNIYKQ
pit-C7      MNKNSYRIIY SKARQMFVAV AENVRSQTKT SGQSEASTQN NIDNTESQAF HQLWQVKALV ASISLWMPLA PVYAGIVADS AANAANRAVI GAGKNSAGTV VPVVNIQTPK NGISHNIYKQ
            121                                                                                                        240
pit-A5/C8   FDVLAEGAVL NNSRQGATTQ TVGNVAANPF LATGEARVIL NEVNSSAASR FEGNLEVAGQ MADVIIANPS GINIKGGGFI NANKAIFTTG KPQLNADGSI KQFTVDQGKI TVSANPNSKF
pit-A5      FDVLAEGAVL NNSRQGATTK TVGNVAANPF LATGEARVIL NEVNSSAASR FEGNLEVAGQ MADVIIANPS GISIKGGGFI NANKAIFTTG KPQLNADGSI KQFTIDQGKI TVSANPNSKF
pit-A5/A4   FDVLAEGAVL NNSRQGATTK TVGNVAANPF LATGEARVIL NEVNSSAASR FEGNLEVAGQ MADVIIANPS GINIKGGGFI NANKAIFTTG KPQLNADGSI KQFTVDQGKI TVSANPNSKF
pit-A5/Hn2  FDVLAEGAVL NNSRQGATTK TVGNVAANPF LATGEARVIL NEVNSSAASR FEGNLEVAGQ MADVIIANPS GINIKGGGFI NANKAIFTTG KPQLNADGSI KQFTIDQGKI TVSANPNSKF
pit-A5/Hn1  FDVLAEGAVL NNSRQGATTK TVGNVAANPF LATGEARVIL NEVNSSAASR FEGNLEVAGQ MADVIIANPS GISIKGGGFI NANKAIFTTG KPQLNADGSI KQFTVDQGKI TVSANPNSKF
pit-A5/C7   FDVLAEGAVL NNSRQGATTQ TVGNVAANPF LATGEARVIL NEVNSSAASR FEGNLEVAGQ MADVIIANPS GISIKGGGFI NANKAIFTTG KPQLNADGSI KQFTVDQGKI TVSANPNSKF
nos-C8      FDVLAEGAVL NNSRQGATTQ TVGNVAANPF LATGEARVIL NEVNSSAASR FEGNLEVAGQ MADVIIANPS GINIKGGGFI NANKAIFTTG KPQLNADGSI KQFTVDQGKI TVSANPNSKF
pit-A4      FDVLAEGAVL NNSRQGATTK TVGNVAANPF LATGEARVIL NEVNSSAASR FEGNLEVAGQ MADVIIANPS GINIKGGGFI NANKAIFTTG KPQLNADGSI KQFTVDQGKI TVSANPNSKF
pit-C7      FDVLAEGAVL NNSRQGATTQ TVGNVAANPF LATGEARVIL NEVNSSAASR FEGNLEVAGQ MADVIIANPS GINIKGGGFI NANKAIFTTG KPQLNADGSI KQFTVDQGKI TVSANPNSKF
            241                                                                                                        360
pit-A5/C8   GLGGNNNDAN YVDLYARALE LSAELRAKND IQVIAGANNV SADLQDVTAK TGTGTAPTLA VDVKALGGMY ANNIYLMGTE KGLGVTNAGT IQAVNNLVVT SAGKIEHSGT ISSTSKTQGL
pit-A5      GLGGNNNDAN YVDLYARALE LSAELRAKND IQVIAGANNV SADLQDVTAK TGTGTAPTLA VDVKALGGMY ANNIYLMGTE KGLGVTNAGT IQAVNNLVIT SAGKIEHSGT FSSTSKTQGL
pit-A5/A4   GLGGNNNDAN YVDLYARALE LSAELRAKND IQVIAGANNV SADLQDVTPK TGTGTAPTLA VDVKALGGMY ANNIYLMGTE KGLGVTNAGT VQAVNNLVIT SAGKIEHSGT ISSTSKTQGL
pit-A5/Hn2  GLGGNNNDAN YVDLYARALE LSAELRAKND IQVIAGANNV SADLQDVTAK TGTGTAPTLA VDVKALGGMY ANNIYLMGTE KGLGVTNAGT IQAVNNLVIT SAGKIEHSGT ISSTSKTQGL
pit-A5/Hn1  GLGGNNNDAN YVDLYARALE LSAELRAKND IQVIAGANNV SADLQDVTAK TGTGTAPTLA VDVKALGGMY ANNIYLMGTE KGLGVTNAGT IQAVNNLVIT SAGKIEHSGT ISSTSKTQGL
pit-A5/C7   GLGGNNNDAN YVDLYARALE LSAELRAKND IQVIAGANNV SADLQDVTAK TGTGTAPTLA VDVKALGGMY ANNIYLMGTE KGLGVTNAGT IQAVNNLVIT SAGKIEHSGT ISSTSKTQGL
nos-C8      GLGGNNNDAN YVDLYARALE LSAELRAKND IQVIAGANNI STDLQGVTAK TGTGTAPTLA VDVKALGGMY ANNIYLMGTE KGLGVTNAGT IQAVNNLVVT SAGKIEHSGT ISSTNKTQGL
pit-A4      GLGGNNNDAN YVDLYARALE LSAELRAKND IQVIAGANNV SADLQDVTPK TGTGTAPTLA VDVKALGGMY ANNIYLMGTE KGLGVTNAGT VQAVNNLVIT SAGKIEHSGT ISSTSKTQGL
pit-C7      GLGGNNNDAN YVDLYARALE LSAELRAKND IQVIAGANNV SADLQDVTAK TGTGTAPTLA VDVKALGGMY ANNIYLMGTE KGLGVTNAGT IQAVNNLVIT SAGKIEHSGT ISSTSKTQGL
            361                                                                                                        480
pit-A5/C8   VNIQTTGTGA AADINSSGSI NSNSMLNIDS GNNLNVNAKE IIINNGSLAS SPLIVNAKGN INLAADTRIM DDSQGGDVYI DAANINLAAG SELKSNRGTA TIQVQKDLVA AKGAKLIAAK
pit-A5      VNIQTTGTGA VADINSSGSI NSNSMLNIDS GNNLNVNAKE IIINNGSLAS SPLIVNTKGN INLAADTRIM DDSQGGDVYI DAANINLAAG SELKSNRGTA TIQVQKDLVA AKGAKLIAAK
pit-A5/A4   VNIQTTGTGA AADINSSGSI NSNSMLNIDS GNNLNVNAKE IIINVGSLAS SPLIVNAKGN INLAADTRIM DDAQGGDVYI DAANINLAAG SELKSNRGTA TIQVQKDLVA AKGAKLIAAK
pit-A5/Hn2  VNIQTTGTGA AADINSSGSI NSNSMLNIDS GNNLNVNAKE IIINVGSLAS SPLIVNAKGN INLAADTRIM DDSQGGDVYI DAANINLAAG SELKSNRGTA TIQVQKDLVA AKGAKLIAAK
pit-A5/Hn1  VSVQTTGTGA AADINSSGSI NSNSMLNIDS GNNLNVNAKE ILINVGSVAS SPLIVNAKGN INLAADTRIM DDSQGGDVYI DAANINLAAG SELKSNRGTA TIQVQKDLVA AKGAKLIAAK
pit-A5/C7   VNIQTTGTGA AADINSSGSI NSNSMLNIDS GNNLNVNAKE IIINNGSLAS SPLIVNAKGN INLAADTRIM DDSQGGDVYI DAANINLAAG SELKSNRGTA TIQVQKDLVA AKGAKLIAAK
nos-C8      VNIQTTGTGA AADINSSGSI NSNSALNIDS GNNLNVNAKE IIINNGSLAS SPLIVNVKGN INLAADTRIM DDSQGGDVYI DAANINLAAG SELKSNRGTA SIQVKKDLVA AKGAKLIAAQ
pit-A4      VNIQTTGTGA AADINSSGSI NSNSALNIDS GNNLNVNAKE IIINNGSLAS SPLIVNAKGN INLAADTRIM DDSQGGDVYI DAANINLAAG SELKSNRGTA TIQVQKDLVA AKGAKLIAAK
pit-C7      VNIQTTGTDA AGDINSSGSI NSNSMLNIDS GNNLNVNAKE IIINVGSLAS SPLIVNAKGN INLAADTRIM DDSQGGDVYI DAANINLAAG SELKSNRGTA TIQVQKDLVA AKGAKLIAAK
            481                                                                                                        600
pit-A5/C8   DLNVLSNGKL SLTENHIQAS LGSINLQANS ANSQNIIDLQ GGTIYAGKDL NLYSSGDLNL KNLGFSLENA ATRVKNIKAY SGRNLVWNNA DKALPLITGM VQLDAANNLT VTAKEISNKD
pit-A5      DLNVLSNGKL SLTENHIQAS LGSINLQANS ANTQNLIDLQ GGTIYAGKDL NLYSSGDLNL KNLGFSLENA ATRVKNIKAY SGRDLVWNNA DKALPLITGL VQLDAANNLT VTAKEISNKD
pit-A5/A4   DLNVLSNGKL SLTENHIQAS LGSINLQANS ANIQNLIDLQ GGTIYAGKDL NLYSSGDLNL KNLGFSLENA ATRVKNIKAY SGRDLVWNNA DKALPLITGL VQLDAANNLT VTAKEISNKD
pit-A5/Hn2  DLNILSNGKL SLTENHIQAS LGSINLQANS ANTQNLIDLQ GGTIYAGKDL NLYSSGDLNL KNLGFSLENA ATRVKNIKAY SGRDLVWNNA DKALPLITGL VQLDAANNLT VTAKEISNKD
pit-A5/Hn1  DLNVLSNGKL SLTENHIQAS LGSINLQANS ANTQNIIDLQ GGTIYAGKDL NLYSSGDLNL KNLGFSLENA ATRVKNIKAY SGRNLVWNNA DKALPLITGM VQLDAANNLT VTAKEISNKD
pit-A5/C7   DLNVLSNGKL SLTENHIQAS LGSINLQANS ANTQNLIDLQ GGTIYAGKDL NLYSSGDLNL KNLGFSLENA ATRVKNIKAY SGRDLVWNNA DKALPLIIGM VQLDAANNLT VTAKEISNKD
nos-C8      DLNVLSNGKL SLTENHIQAS LGSINLQANS ANTQNLIDLQ GGTIYAGKDL NLYSSGDLNL KNLGFSLENA ATRVKNIKAY SGRDLVWNNA DKALPLITGL VQLDAANNLT VTAKEISNKD
pit-A4      DLNVLSNGKL SLTENHIQAS LGSINLQANS ANIQNLIDLQ GGTIYAGKDL NLYSSGDLNL KNLGFSLENA ATRVKNIKAY SGRDLVWNNA DKALPLITGL VQLDAANNLT VTAKEISNKD
pit-C7      DLNVLSNGKL SLTENHIQAS LGSINLQANS ANIQNLIDLQ GGTIYAGKDL NLYSSGDLNL KNLGFSLENA ATRVKNIKAY SGRDLVWNNA DKALPLITGL VQLDAANNLT VTAKEISNKD
            601                                                                                                        720
pit-A5/C8   SIQLHANQIT LNSALTSQKN IDVSSEIADL VLSQVLKAQG DINLTALAGG VTANSLKTTS SAGKISILAN KNINLNSTQT SKAMPSADKD ELTTDQTVIS GLKGVTLGSI GDGTVNLQSV
pit-A5      SIQLHANQIA LNSALTSQKN IDVSSEIADL VLSQALKAQG DINLTALAGG VTANSLKATS SAGKISILAN KNINLNSTQT SKAMPSADKD ELTTDQTVIS GLKGVTLGSI GDGTVNLQSV
pit-A5/A4   SIQLHANQIA LNSALTSQKN IDVSSEIADL VLSQALKAQG DINLTALAGG VTANSLKATS SAGKISILAN KNINLNSTQT TKAMPSADKD ELTTDQSVIS GLKGVTLGSI GDGTVNLQSV
pit-A5/Hn2  SIQLHANQIA LNSALTSQKN IDVSSEIADL VLSQALKAQG DINLTALAGG VTANSLKATS SAGKISILAN KNINLNSTQT SKAMPSADKD ELTTDQTVIS GLKGVTLGSI GDGTVNLQSV
pit-A5/Hn1  SIQLHANQIA LNSALTSQKN IDVSSEIADL VLSQALKAQG DINLTALAGG VTANSLKATS SAGKISILAN KNINLNSTQT SKAMPSADKD ELTTDQSVIS GLKGVTLGSI GDGTVNLQSV
pit-A5/C7   SIQFHANQIA LNSALTSQKN IDVSSEIADL VLSQALKAQG DINLTALAGG VTANSLKATS SAGKISILAN KNLNLNSTQT TKAMPSADKD ELTTDQSVIS GLKGVTLGSI GDGTVNLQSV
nos-C8      SIQLHANQIA LNSALTSQKN IDVSSEIADL VLSQALKAQG DINLTALAGG VAANSLKATS SAGKISILAN KNINLNSTQT SKAVPATDKD ELTTDQIVIS GLKGVTLGSI GDGTVNLQSV
pit-A4      SIQLHANQIA LNSALTSQKN IDVSSEIADL VLSQALKAQG DINLTALAGG VTANSLKATS SAGEISILAN KNISLNSTQT TKAMPSADKD ELTTDQSVIS GLKGVTLGSI GDGTVNLQSV
pit-C7      SIQLHANQIA LNSALTSQKN IDVSSEIADL VLSQALKAQG DINLTALAGG VTANSLKATS SAGKISILAN KNISLNSTQT TKAMPSADKD ELTTDQSVIS GLKGVTLGSI GDGTVNLQSV
            721                                                                                                        840
pit-A5/C8   QVNASQGDIL VSSNNGINLK ANNDVVVTAN KGQVKTVNNV LKGQTVSIEN NKSDIKIQNT DLSSTVGKLA INSRAGMSTI IDSVLTSKGN TELYAKDLLT LQGVNATSDQ HLAVSSGRTV
pit-A5      QVNANQGDIL VSSNNGINLK ANNDVVVTAN KGQVKTVNNV LKGQTVSIEN GKSDIKIQNT DLGSTVGKLA VNSRAGMSTI IDSVLTSKGN TELYAKDLLT LQGVNATSDQ HLAVSSGRTV
pit-A5/A4   QVNANQGDIL VSSNNGINLK ANSDVVVTGD TGRFKTVNNV LKGQTVSIEN SKSDIKIQNT DLSSTVGKLA INSRAGMSTI IDSVLTSKGN TELYAKDLLT LQGVNATSDQ HLAVSSGRTV
pit-A5/Hn2  QVNANQGDIL VSSNNGINLK ANNDVVVTAN KGQVKTVNNV LKGQTVSIEN SKSDIKVQNT DLGSTVGKLA VNSRAGMSTI IDSVLTSKGN TELYAKDLLT LQGVNATSDQ HLAVSSGRTV
pit-A5/Hn1  QVNAGQGDIL VSSNNGINLK ANNDVVVTGD TGRFKTVNNV LKGQTVSIEN SKSDIKIQNT DLSSTVGKLA INSRAGMSTI IDSVLTSKGN TELYAKDLLT LQGVNATSDQ HLAVSSGRTV
pit-A5/C7   QVNANQGDIL VSSNNGINLK ANTDVVVTGD TGRFKTVNNV LKGQTVSIEN SKSDIKIQNT DLSSTVGKLA INSRAGMSTI IDSVLTSKGN TELYAKDLLT LQGVNATSDQ HLAVSSGRTV
nos-C8      QVNANQGDIL VSSNNGINLK ANTDVIVTGN NGQFKTVNNV LKGQIVSIEN SKSDIKIQNT DLSSTVGKLA INSRAGMSMI TDSVLTSKGN TELYAKDLLT LQGVNATSDQ HLAVSSGLSV
pit-A4      QVNANQGDIL VSSNNGINLK ANSDVVVTGD TGRFKTVNNV LKGQTVSIEN SKSDIKIQNT DLSSTVGKLA INSRAGMSTI IDSVLTSKGN TELYAKDLLT LQGVNATSDQ HLAVSSGRTV
pit-C7      QVNANQGDIL VSSNNGINLK ANSDVVVTGD TGRFKTVNNV LKGQTVSIEN SKSDIKIQNT DLSSTVGKLA INSRAGMSTI IDSVLTSKGN TELYAKDLLT LQGVNATSDQ HLAVSSGRTV
            841                                                                                                        960
pit-A5/C8   YSNAEYTPAT KWIADKVTNL TSKGVTSVTA TGNQVLQNTN LTGGAVLLEA GGFILGQTGL NLNAVGSDLL KNDTKLNSLN GDLTIQTNSN LTIDPKVYSL KAVGDIELVS KTGTLTLKGY
pit-A5      YSNAEYTPAT KWIADKVTNL TSKGVTSVTA TGNQVLQNTN LTGGAVLLEA GGFILGQTGL NLNAVGSDLL KNDTKLNSLN GDLTIQTNSN LTIDPKVYSL KAVGDIELVS KTGTLTLKGY
pit-A5/A4   YSNAEYTPAT KWIADKVTNL TSKGVTSVTA TGNQVLQNTN LTGGAVLLEA GGFILGQTGL NLNAVGSDLL KNDTKLNSLN GDLTIQTNSN LTIDPKVYSL KAVGDIELVS KNGTLTLKGY
pit-A5/Hn2  YSNAEYTPAT KWIADKVTNL TSKGVTSVTA TGNQVLQNTN LTGGAVLLEA GGFILGQTGL NLNAVGSDLL KNDTKLNSLN GDLTIQTNSN LTIDPKVYSL KAVGDIELVS KTGTLTLKGY
pit-A5/Hn1  YSNAEYTPAT KWIADKVTNL TSKGVTSVTA TGNQVLQNTN LTGGAVLLEA GGFILGQTGL NLNAVGSDLL KNDTKLNSLN GDLTIQTNSN LTIDPKVYSL TAVGDIELVS KTGTLTLKGY
pit-A5/C7   YSNAEYTPAT KWIADKVTNL TSKGVTSVTA TGNQVLQNTN LTGGAVLLEA GGFILGQTGL NLNAVGSDLL KNDTKLNSLN GDLTIQTNSN LTIDPKVYSL KAVGDIELVS KTGTLTLKGY
nos-C8      YSNRENTPSP LLIADKVTNL TSKGVTSVTA TGNQVLKNTN LTGGAVLLEA GGFITGQTGL NLNAVGSDLL KNDTKLNSLN GDLTIQSNSN LTIDPKAYNL KAVGDIELVS KAGTLTLKGY
pit-A4      YSNAEYTPAT KWIADKVTNL TSKGVTSVTA TGNQVLQNTN LTGGAVLLEA GGFILGQTGL NLNAVGSDLL KNDTKLNSLN GDLTIQTNSN LTIDPKVYSL KAVGDIELVS KNGTLTLKGY
pit-C7      YSNAEYTPAT KWIADKVTNL TSKGVTSVTA TGNQVLQNTN LTGGAVLLEA GGFILGQTGL NLNAVGSDLL KNDTKLNSLN GDLTIQTNSN LTIDPKVYSL KAVGDIELVS KTGTLTLKGY
            961
pit-A5/C8   GGTTGNGSEQ VVKLDTANGG INLEGAKVDI QGSQLTAQKD IKIVSSKDDV LIDGIKNTLK NSSADSKNIL NIENNIKEID KSINILSNSK EFQERKTLLD NIKKTKNSLE AAMSYLFDGA
pit-A5      GGTTGNGSEQ VVKLDTANGG INLEGAKVDI QGSQLTAQKD IKIVSSKDDV LIDGMKNSFI NRKKQEKLNE ANNLKKNISD SLALLKSQQF FTDYDNMVTA YE-------- ----------
pit-A5/A4   GGTAGNGSEQ VVKLDTANGG INLEGAKVDI QGSQLIAQKD IKIVSSKDDA LIDGVKNNFS SLQKLNLAKD YTLKIEEMNK ERLKLQSQDY FNDYSNLVKK YR-------- ----------
pit-A5/Hn2  GGTTGNGSEQ VVKLDTANGG INLEGAKVDI QGSQLTAQKD IKIVSSKDDV LIDGIKNSLK NLQSKKYSQN SIDDFNNIKT TLESLKTSEE YQAYTKDIGT MK-------- ----------
pit-A5/Hn1  GGTTGNGSEQ VVKLDTANGG INLEGAKVDI QGSQLTAQKD IKIVSSKDDV LVDGVKNTIL DSTKITSFER LAAVTSEKID LEIQHEKMRK DPDYLFLINE LD-------- ----------
pit-A5/C7   GGTTGNGSEQ FVKLDTANGG INLEGAKVDI QGSQLTAQKD IKIVSSKDDV LIDGLKNNIT NKISISKLEY SKKELENINN IIQKFKDTEN FKLYKNSIDN IN-------- ----------
nos-C8      GGTAGNGSEQ VVKLDTANGG INLEGAKVDI QGSQLTAQKD IKIVSSKDDV LVDGIKNTLN NISADSKNIL NIEKNIKEID NSINILSNSK EFQERNTLID NIKKTKNSLE AALSYLFDGS
pit-A4      GGTTGNGSEQ VVKLDTANGG INLEGAKVDI QGSQLTAQKD IKIVSSKDDV LIDGVKNNFS SLQKLNLAKD YTLKIEEMNK ERLKLQSQDY FNDYSNLVKK YR-------- ----------
pit-C7      GGTTGNGSEQ FVKLDTANGG INLEGAKVDI QGSQLTAQKD IKIVSSKEDV LIDGLKNNIT NKISISKLEY SKKELENINN IIQQFKDTEN FKLYKNSIDN IN-------- ----------

pit-A5/C8   NGEYSAGALS NVDVSDIFFY ENAYIKTYSA SEG-DSGSST FIVIPRTPWL HDDDSIRGFI SSENYKVIP- LLDSYKAEIK KYKNLNESEK VKELEKLISK LESDKQSEIK SK--------
pit-A5      ---------- ---------- ---------- ---------- -DYTSSNYDY SLYMKHYNLY NDFISKYPVR PNPRLLIFGS DKPLFHSIFS EPQRMTRFDA LYSFEPKYDE NYLRSLQAEV
pit-A5/A4   NYNDMFLKYW DYELLDQTKN KNSEIWVNAY KEYERDFNVF KDKYPIKINY PVPGQLPFLL GGNNTPLAVD YVFGFKYDND YINDVEKNE- ---------- ---------- ----------
pit-A5/Hn2  ---------- ---------- ---------- ---------- ---------- ---------- ----SMRGYA QLVEFSGNHD FAMHLIEESN KIEKNLSKYS TLLEAIELNE KSLNDAEILK
pit-A5/Hn1  ---------- ---------- ---------- ---------- ---------- ------KGRG LKYEDKVDVE KMYALRDALE KRYLVKIDIE AGAGRRGKKV FFIDAYEKYT DKISELDQSI
pit-A5/C7   NSINSKRKEW ERAVSDDLHS KKRALSDQLK KLEEEREDIR NKFKDIIDQE NKLNNLLDER NNYF------ ---------- ---------- ---------- ---------- ----------
nos-C8      NGEYSAGALS NVDVSNIFFY ENAYIQSF-S SEG ASDTYA MVINNDIVGF LPSVDSIGT- ---------- LLSSYKAEIK KYKNLNESEK VKDLQKLISK LESDKQSEIK SK--------
pit-A4      NYNDMFLK-W DYELLDQTKN KNSEIWVNAY KEYERDFNVF KDKYPIKINY PVPGQLPFLL GGNNTPLAVD YVFGFKYDND YINDVEKNE- ---------- ---------- ----------
pit-C7      NSINSKRKEW ERAVSDDLHS KKRALSDQLK KLEEEREDIR NKFKDIIDQE NKLNNLLDER NNYF------ ---------- ---------- ---------- ---------- ----------
           1201                                                                                                       1320
pit-A5/C8   ELLTKGVNGS EHTETKLTSK SGNINITSVK GLSISGGNIS AQMGQVNLEA SGVLAEQYKS SISTGANQPP KSLNASIIID GHTDFYSNGT ESEGNYSFRT LVSPTIINGD KGVNIRTVGK
pit-A5      DFYNTGINGS EHAEAKLNSK SGNINITSVK GLSISGGNIS AQTGQVNLEA SGVLAEQYKS TINNGANQPP KSLNASIIID GHTDFYEKGT ESEGNYSFRT LVSPTIINGD KGVNIRTVGK
pit-A5/A4   LFYKESLNGS EHAETKLTSK SGNINITSAK GLSISGGNIS AQLGQVNLEA SGVLAEQYKS SISSGVNQPP KSLNASIIVD GHTDFYEKGT ESEGNYSFRT LVSPTIINGD KGVNIRTVGK
pit-A5/Hn2  KMFDSNVEGS EHAEAKLNSK SGNINITSVK GLSISGGNIS AQMGQVNLEA SGVLAEQYKS SISSGANQPP KSLNASIIID GHTDFYEKGT ESEGNYSFRT LVSPTIINGD KGVNIRTVGK
pit-A5/Hn1  NFYNKKFTGN EHRGSSFTSK TGNINIISSK GGSISGSIID AQKGSVSIEA SGALAEQYKS TINSGANQPP KSLNASVIID GHTDFYENGT EGEGNYSFRT LVSPTIINGD KGVNIRTVGK
pit-A5/C7   IYFDSNVSGS EHSETKLNSK SGNINVTSAK GLSISGGNIS AQSGQVNLEA NGVLAEQYKS SINSGANQPP KSLNASIIID GHTDFYEKGT ESEGNYSFRT LVSPTIINGD KGVNIRTVGK
nos-C8      ELLTKGVNGS EHTEAKLTSK SGNINITSAK GLSISGGDIS AQSGQVNLEA SAALSEQYKS TTTTNTNPQP RILNASIIID GHTDFYDKGS ENDQNYSMRT LVSPSIINGD KGVNIRTVGK
pit-A4      LFYKESLNGS EHAETKLTSK SGNINITSAK GLSISGGNIS AQLGQVNLEA SGVLAEQYKS SISSGVNQPP KSLNASIIVD GHTDFYEKGT ESEGNYSFRT LVSPTIINGD KGVNIRTVGK
pit-C7      IYFDSNVSGS EHSEAKIISK LGNVNITSAK GLSISGGNIS AQSGQVNLEA SGVLAEQYKS SISSGANQPP KSLNASIIID GHTDFYSKGT EGEGNYSFRT LVSPTIINGD KGVNIRTVGK
            1321                                                                                                      1440
pit-A5/C8   TKDDNLVLQA TGITSKNGDV KIESNKSILF DAAIEQSYDL TTTTEKKKSW GGLKKKYITT VSENNDTNAA SVDISAKNIS IETKKLDPSV AAQTPDNNID IYSGRFTAEG GTISIKSGGN
pit-A5      TKDDNLVLQA TGITSKNGDV KVESNKSILF DAAIEQSYDL TTTTEKKKSW GGLKKKYITT VSENNDTNAA SVDISAKNIS IETKKLDTSV NVKTPDNNID IYSGRFTAEG GTISIKSGGN
pit-A5/A4   TKDDNLVLQA TGITSKNGDV KIESNKSILF DAAIEQSYDL TTTTEKKKSW GGLKKKYITT VSENNDTNAA SVDISAKNIS IETKKLDPSV DAKTPDNNID IYSGRFTAEG GTISIKSGGN
pit-A5/Hn2  TKDDNLVLQA TGITSKNGDV KIESNKSILF DAAIEQSYDL TTTTEKKKSW GGLKKKYITT VSENNDTNAA SVDISAKNIS IETKKLDTSV NVKTPDNNID IYSGRFTAEG GTISIKSGGN
pit-A5/Hn1  TKDDNLVLQA TGITSKNGDV KIESNKSILF DAAIEQSYDL TTTTEKKKSW GGLKKKYITT VSENNDTNAA SVDISGKNIS IETKKLDTSV NAKTPDNNID IYSGRFTAEG GIISIKSGGN
pit-A5/C7   TKDDNLVLQA TGITSKNGDV KIESNKSILF DAAIEQSYDL TTTTEKKKSW GGLKKKYITT VSENNDTNAA SVDISAKNIS IETKKLDPSV AAQTPDNNID IYSGRFTAEG GTISIKSGGN
nos-C8      SKDDNLVLQA TGITSKNGDV KIESNKSILF DAAVEQSYDR SITTEKKKSW GGLKKKYITT VAESNSTNAA SVDISGKNIF IETKKLDPSV NVSTPDNNID IYSGRFTAEG GQIKINSGGN
pit-A4      TKDDNLVLQA TGITSKNGDV KIESNKSILF DAAIEQSYDL TTTTEKKKSW GGLKKKYITT VSENNDTNAA SVDISAKNIS IETKKLDPSV DAKTPDNNID IYSGRFTAEG GTISIKSGGN
pit-C7      TKDDNLVLQA TGITSKNGDV KIESNKSILF DAAIEQSYDL TTTTEKKKSW GGLKKKYITT VSENNDANAA SVDISAKNIS IETKKLDPSV DAKTPDNNID IYSGRFTAEG GTISIKSGGN
            1441                                                                                                      1560
pit-A5/C8   LNFYTVEESS SSTVDVTKKT SYSKLISLLG SSKTTTNTTR TQISELPANL KADYINTQAV DKTKLVGTEF EYLKGATIET GGTLELIAAK TSITELLKKE KSSLAWQAMQ DKGSITETAQ
pit-A5      LNFYTVEESS SSTVDVTKKT SYSKLISLLG SSKTTTNTTR TQISELPANL KADYINTQAV DKTKLVGTEF DYLKNATIET GGTLELIAAK TSITELLKKE KSSLAWQSMQ DKGSITETAQ
pit-A5/A4   LNFYTVEESS SSTVDVTKKT SYSKLISLLG SSKTTTNTTR TQISELPANL KADYINTQAV DKTKLVGTEF EYLKGATIET GGTLELIAAK TSITELLKKE KSSLAWQAMQ DKGSITETAQ
pit-A5/Hn2  LNFYTVEESS SSTVDVTKKT SYSKLISLLG SSKTTTNTTR TQISELPANL KADYINTQAV DKTKLVGTEF DYLKNATIET GGTLELIAAK TSITELLKKE KSSLAWQSMQ DKGSITETAQ
pit-A5/Hn1  LNFYTVEESS SSTVDVTKKT SYSKLISLLG SSKTTTNTTR TQISELPANL KADYINTQAV DKTKLVGTEF DYLKNATIET GGTLELIAAK TSITELLKKE KSSLAWQSMQ DKGSITETAQ
pit-A5/C7   LNFYTVEESS SSTVDVTKKT SYSKLISLLG S-KTTTNTTR TQISELPANL KADYINTQAV DKTKLVGTEF DYLKNATIET GGTLELIAAK TSITEVLKKE KSSLAWQSMQ DKGSITETAQ
nos-C8      LNFYTVEESS SSNVDITKKS SFAGI----K YNTSKTNATR TQVSELPATL KADYIGTKSG FDTRLVGTEF EYLKDATIEA GGKLELIAAK TSITDLVKKE KNSVVWQSMQ DKGSITETAQ
pit-A4      LNFYTVEESS SSAVDVTKKT SYSKLISLLG SSKTTTNTTR TQISELPANL KADYINTQAV DKTKLVGTEF DYLKNATIET GGTLELIAAK TSITELLKKE KSSLAWQSMQ DKGSITETAQ
pit-C7      LNFYTVEESS SSTVDVTKKT SYSKLISLLA SSKTTTNTTR TQISELPATL KADYIGTKSG FDTKLVGTEF EYLKDATIET GGTLELIAAK TSITELLKKE KSSLAWQAMQ DKGSITETAQ
            1561                                                                                                      1680
pit-A5/C8   LPSFNGPVLP TFKAAGGLSV QVPISEKDAN KVELRDAILD LAKQPGNEYL KEFVNRKDVD WQKVLLTQKD WDYKQQGLTA AGAAIIVIII TIVTMGTGTA AAAGAAGGTA ASGTTVGLGA
pit-A5      LPSFNGPVLP TFKATGGLSV QVPISEKDAN KVELRDAILD LAKQPGNEYL KEFVNRKDVD WQKVLLTQKD WDYKQQGLTA AGAAIIVIII TIVTMGTGTA AAA--AGGTA ASGTTVGLGA
pit-A5/A4   LPSFNGPVLP TFKAAGGLSV QVPISEKDVN KVELRDAILD LSKKPGNEYL KELVNRKDVD WQKVLLTQKD WDYKQQGLTA AGAAIIVIII TIVTMGTGTA AAAGAAGGTA ASGTTVGLGA
pit-A5/Hn2  LPSFNGPVLP TFKAAGGLSV QVPISEKDTN KVELRDAILD LAKQPGNEYL KEFVNRKDVD WQKVLLTQKD WDYKQQGLTA AGAAIIVIII TIVTMGTGTA AAAGAAGGTA ASGTTVGLGA
pit-A5/Hn1  LPSFNGPVLP TFKAAGGLSV QVPISEKDAN KVELRDAILD LAKQPGNEYL KEFVNRKDVD WQKVLLTQKD WDYKQQGLTA AGAAIIVIII TIVTMGTGTA AAAGAAGGTT ASGTTVGLGA
pit-A5/C7   LPSFNGPVLP TFKAAGGLSV QVPISEKDAN KVELRDAILD LAKQPGNEYL KEFVNRKDVD WQKVLLTQKD WDYKQQGLTA AGAAIIVIII TIVTMGTGTA AAAGAAGGTA ASGTTVGLGA
nos-C8      LPSFNGPVLP TFKAAGGLSV QVPISEKDAN KVELRDEILK LANQPGNAYL KELVNRKDVD WQQILLTQKD WDYKSQGLTG AGAAIIVIIV TIVTMGSGTA AAAGAAGGAA ASGTTVGLGA
pit-A4      LPSFNGPVLP TFKAAGGLSV QVPISEKDAN KVELRDAILD LSKKPGNEYL KEFVNRKDVD WQIVLLTQKD WDYKSQGLTG AGAAIIAIVV TMVTAGAG-A AVVGALGGSA AT-----LGG
pit-C7      LPSFNGPVLP TFKAAGGLSV QVPISEKDAN KIELRDAILE LSKQPGNEYL KEFVNRKDVD WQMVLLTQKD WDYKSQGLTG AGAAIIAIVV TMVTAGAG-A AVVGALGGSA AT-----LGG
            1681                                                                                                       1800
pit-A5/C8   SMIGSAGVTT ATVGGVTTIT GVSTLGAMAN AAITSLATQA GVSLINNGGD IGKTLKDLGS KDSIKSLATS VVTAGVLSKI ASIDQMKALQ ETTKSGEFFT NLSGRVGQAL VNSTVSASVD
pit-A5      SMIGSAGVTT ATVGGVTTIT GVSTLGAMAN AAITSLATQA GVSLINNGGD IGKTLKDLGS KDSIKSLATS VVTAGVLSKI ASIDQMKALQ ETTKSGEFFT NLSGRVGQAL VNSTVSASVD
pit-A5/A4   SMIGSAGVTT ATVGGVTTIT GVSTLGAMAN AAITSLATQA GVSLINNGGD IGKTLKDLGS KDSIKSLATS VVTAGVLSKI ASIDQMKALQ ETTKSGEFFT NLSGRVGQAL VNSTVSASVD
pit-A5/Hn2  SMIGSAGVTT ATVGGVTTIT GVSTLGAMAN AAITSLATQA GVSLINNGGD IGKTLKDLGS KDSIKSLATS VVTAGVLSKI ASIDQMKALQ ETTKSGEFFT NLSGRVGQAL VNSTVSASVD
pit-A5/Hn1  SMIGSAGVTT ATVGGVTTIT GVSTLGAMAN AAITSLATQA GVSLINNGGD IGKTLKDLGS KDSIKSLATS VVTAGVLSKI ASIDQMKALQ ETTKSGEFFT NLSGRVGQAL VNSTVSASVD
pit-A5/C7   SMIGSAGVTT ATVGGVTTIT GVSTLGAMAN AAITSLATQA GVSLINNGGD IGKTLKDLGS KDSIKSLATS VVTAGVLSKI ASIDQMKALQ ETTKSGEFFT NLSGRVGQAL VNSTVSASVD
nos-C8      SMLGAAGVTT TAAGAIVP-- --STLGAMAN AAVTSLATQA SVSLINNGGN IGKTLKDLGS KDSIKNLATS VVTAGLMSQV GSALNLKP-D STMLPDRLIN NISSSVGSTL IQ--------
pit-A4      SAI------- ---------- ------AMTQ AALTSLATQA SVSLINNGGN IGKTLKELGS KDSVKNLAAS VVTAGLLSQV STALNLKP-D STVLSDRLIN NFTTSVGSTL VQ--------
pit-C7      SAI------- ---------- ------AMTQ AALTSLATQA SVSLINNGGN IGKTLKELGS KDSIKNLAAS VVTAGLLSQV STALNLKP-D STVFSDRLVN NFTTSVSSTL VQ--------
            1801                                                                                                       1920
pit-A5/C8   TAVNGGSLSE KLSASLVAGL SVALQGSLAG EIGENLDLGS KELSDQILHK LAHAAAGCIA SSL-QEKCEA GAIGAVVGEM VAGSFDMEPE NDGDYSQAQL NKVKNVGQLI SGVVAASAGY
pit-A5      TAVNGGSLSE KLSASLVAGL SVALQGSLAG EIGENLDLGS KELSDQILHK LAHAAAGCIA SSL-QEKCEA GAIGAVVGEM VAGSFDMEPE NDGDYSQAQL NKVKNVGQLI SGVVAASAGY
pit-A5/A4   TAVNGGSLSE KLSASLVAGL SVALQGSLAG EIGENLDLGS KELSDQILHK LAHAAAGCIA SSL-QEKCEA GAIGAVVGEM VAGSFDMEPE NDGDYSQAQL NKVRNVGQLI SGVVAASAGY
pit-A5/Hn2  TAVNGGSLSE KLSASLVAGL SVALQGSLAG EIGENLDLGS KELSDQILHK LAHAAAGCIA SSL-QEKCEA GAIGAVVGEM VAGSFDMEPE NDGDYSQAQL NKVRNVGQLI SGVVAASAGY
pit-A5/Hn1  TAVNGGSLSE KLSASLVAGL SVALQGSLAG EIGENLDLGS KELSDQILHK LAHAAAGCIA SSL-QEKCEA GAIGAVVGEM VAGSFDMEPE NDGDYSQAQL NKVKNVGQLI SGVVAASAGY
pit-A5/C7   TAVNGGSLSE KLSASLVAGL SVALQGSLAG EIGENLDLGS KELSDQILHK LAHAAAGCIA SSL-QEKCEA GAIGAVVGEM VAGSFDMEPE NDGDYSQAQL NKVRNVGQLI SGVVAASAGY
nos-C8      TAIKGGDLEE NLKVALLSGL AGALQGELAS QIGDSLYKSD PTILQYTIHK IAHAAAGCAA AAATKSSCEA GAIGAGIGEI VA-SLMPKPI NGIEYNDEEK VKIKNVGKVV AGVVSAYAGY
pit-A4      TAIKGGDLEE NLKVALLAGL AGALQGELAS QIGDSLYKAD PTVLQYTIHK IAHAAAGCAV AAATKQSCEA GAIGAGVGEI IAELMIPYGK TALDLDDNEI NRIKGVSKIV AGSISAYAGY
pit-C7      TAINGGDLEE NLKVALLSGL AGALQGQMAQ NIKGL----- -EDINYIAHK LAHALAGC-I AGAIQGQCQA GAIGGAVGEI VADFIPP--S SGLLYTEEEK QKILMVGQLT SGVIAAYAGY
```

```
            1921                                                                                                                   2040
pit-A5/C8   DVTVASQSAS TAIENN---- WGKVLSSTGK AAYKIFKKAT E-LQKAGKNL NDTNVWKDIL KDTATGEYQD MLNSFKTAFS AGSTPLERTW ALIDLATGID KKDVKIVQDL LKDRNTIAKR
pit-A5      DVTVASQSAS TAIENN---- WGKVLSSTGK AAYKIFKKAT E-LQKAGKNL NDTNVWKDIL KDTATGEYQD MLNSFKTAFS AGSTPLERTW ALIDLATGID KKDVKIVQDL LKDRNTIAKR
pit-A5/A4   DVTVASQSAS TAIENN---- WGKVLSSTGK AAYKIFKKAT E-LQKAGKNL NDTNVWKDIL KDTATGEYQD MLNSFKTAFS AGSTPLERTW ALIDLATGID KKDVKIVQDL LKDRNTIAKR
pit-A5/Hn2  DVTVASQSAS TAIENN---- WGKVLSSTGK AAYKIFKKAT E-LQKAGKNL NDTNVWKDIL KDTATGEYQD MLNSFKTAFS AGSTPLERTW ALIDLATGID KKDVKIVQDL LKDRNTIAKR
pit-A5/Hn1  DVTVASQSAS TAIENN---- WGKVLSSTGK AAYKIFKKAT E-LQKAGKNL NDTNVWKDIL KDTATGEYQD MLNSFKTAFS AGSTPLERTW ALIDLATGID KKDVKIVQDL LKDRNTIAKR
pit-A5/C7   DVTVASQSAS TAIENN---N WGKVLSSTGK AAYKIFKKAT E-LQKAGKNL NDTNVWKDIL KDDMC
nos-C8      DVNTAANSAD IAIQNNYLTH YSKAKFVADL AKCKVTSSNC DTIIQNYQNL SKTNDLELAT VCNNTARSRD CNNMLKAALD YAGNDFNQIY GINSPAMA-- ----NLKADQ IRSRELVLKN
pit-A4      DVNTAANSAD TAISNN---S LPKLATSGAK IAKKVLDQIK K--WPSNKPL EPND-VTKMF KDAGVQEIVD VANNIKTLVS PTSTWFEKIS AAVDLAVGID IKAGKDVALA MKDKHIIQLK
pit-C7      DVNVASNSAN IALTNN---A FNE------K KVKELLDQAK G--YMGEKGG QALDAVSRGI AKGDINALKN AQTQIAEYLL KKADSGGLSQ AEMVTFGTLY ALNETLFPTN ILDVIPGAGK
            2041                                                                                                                    2160
pit-A5/C8   LPNIKNGQFF DDKKIEELAN RGDVKKIHQI DVGAKGGWAK QANGKLEANS AYLLPNGA-- AYITDNSGNV KEVTANLRNI LMDRNNYQQS IAGKSGISGD QGGHL-IAAS LGGSGDRINL
pit-A5      LPNIKNGQFF DDKKIEELAN RGDVKKIHQI DVGAKGGWAK QANGKLEANS AYLLPNGA-- AYVTDNSGNV KEVTANLRNI LMDRNNYQQS IAGKSGISGD QGGHL-IAAS LGGSGDRINL
pit-A5/A4   LPNIKNGQFF DDKKIEELVN RGDVKKIHQI DVGAKGGWAK QANGKLEANS AYLLPNGA-- AYVTDNSGNV KEVTANLRNI LMDRNNYQQS IAGKSGISGD QGGHL-IAAS LGGSGDRINL
pit-A5/Hn2  LPNIKNGQFF DDKKIEELAN RGDVKKIHQI DVGAKGGWAK QANGKLEANS AYLLPNGA-- AYVTDNSGNV KEVTANLRNI LMDRNNYQQS IAGKSGISGD QGGHL-IAAS LGGSGDRINL
pit-A5/Hn1  LPNIKNGQFF DDKKIEELAN RGDVKKIHQI DVGAKGGWAK QANGKLEANS AYLLPNGA-- AYVTDNSGNV KEVTANLRNI LMDRNNYQQS IAGKSGISGD QGGHL-IAAS LGGSGDRINL
nos-C8      IFKDVNNWY- --GMINDIDL RADFFSAMYA KTGAE--WFR VANETSRAAI VGLGAEGAYS GATFAIGGVI SSITSAGSVN LLDVYNWRSE AGYALMKAGY NNFKQ-LYDG KVTDIVAWNI
pit-A4      DPGPIHHICT NKNCLSTARG GPWTRQFQKY FDGAGL-DIN KSVENLVALA DHKGPHPKEY HAYVEKELRL AVSGKQANTA EYR-NAVIAT LSRIKSEALV SGSQVNKWLT KK
pit-C7      AIGKGNDLLK GIRAAAPASA ASATARLGLR EDLAKLANIP RNMEIQPANI WGKTIQDIKY SFEMDGAVLT LVPPKSSTSG LAQVYSVKNS ATGIKEFEYH PGGGTHSSDG VRYYKIVMND
            2161                                                      2215
pit-A5/C8   VPMAKTLNNG SWKAMESELA NAVKLGKDVN MKVAVGYPTG SSRPNKFIVT ATINGKSTNY QFSQ
pit-A5      VPMAKTLNNG SWKAMESELA NAVKLGKDVN MKVAVGYPTG SSRPNKFIVT ATINGKSTNY QFSQ
pit-A5/A4   VPMAKTLNNG SWKAMESELA NAVKLGKDVN MKVAVGYPTG SSRPNKFIVT ATINGKSTNY QFSQ
pit-A5/Hn2  VPMAKTLNNG SWKAMESELA NAVKLGKDVN MKVAVGYPTG SSRPNKFIVT ATINGKSTNY QFSQ
pit-A5/Hn1  VPMAKTLNNG SWKAMESELA NAVKLGKDVN MKVAVGYPTG SSRPNKFIVT ATINGKSTNY QFSQ
nos-C8      SQMQQEQKLL QPIHEKYFKN SKLFVPQTWV PTKFPNNPVN LLDYNSRVKY GCIEMGYTIA QGCKP
pit-C7      GTQFRIIDPN KGFKPGTITS NQIYLNPNGQ KLKYENGKWE IWK
```

C)CdiA chimerae

Strains: B3/B5, TCM292; B5, CIP 70.29;

```
       1                                                                                                              120
B3/B5  MNKNLYRIVF NKARNMFMAV AENVRSQSKA AGQSTSSEIE RSGQSSGQEK SFHQLWHVKA LVASISLLVP FAPVYAAIVA DSAANAANRA VIGAGKNSAG VTVPVVNIQT PKNGISHNIY
B5     MNKNLYRIVF NKARNMFMAV AENVRSQSKA AGQSTSSEIV SSGQSLVQEK SFHQLWHVKA LVASISLLVP FAPVYAAIVA DSAANAANRA VIGAGKNSTG TTVPVVNIQT PKNGISHNIY
B7/B5  MNKNLYRIVF NKARNMFMAV AENVRSQSKA AGQSTSSEIV SSGQSLVQEK SFHQIWHVKA LVASISLLVP FAPVYAAIVA DSAANAANRA VIGAGKNSAG TTVPVVNIQT PKNGISHNIY
B7     MNKNLYRIVF NKARNMFMAV AENVRSQSKE AGQSTSSEIE RSGQSSGQEK SFHQLWHVKA LVVSISFLVP FAPVYAAIVA DSAANAANRA VIGAGKNSAG VTVPVVNIQT PKNGISHNIY
B3     MNKNLYRIIF NKARNMFMAV AENVRSQSKA AGQSSSSEIV SYGQSSGQEK SFHQLWHVKA LVTSMSLLVP FAPVYADIVA DSAANAANRA VIAAGKNSAG TTVPVVNIQT PKNGISHNIY

       121                                                                                                            240
B3/B5  KQFDVLAEGA VLNNSRTGAT TKTVGAVGAN PFLATGEARV ILNEVNSTAA SRFEGNLEVA GQMADVIIAN PSGINIKGGG FINANKAIFT TGKPQLNADG SIQQFNVSQG KVTITAPTNS
B5     KQFDVLAEGA VLNNSRTGAT TKTVGTVGAN PFLATGEARV ILNEVNSTAA SRFEGNLEVA GQMADVIIAN PSGINIKGGG FINANKAIFT TGKPQLNADG SIKQFTVDQG KITVSANPNS
B7/B5  KQFDVLAEGA VLNNSRTGAT TKTVGTVGAN PFLATGEARV ILNEVNSTAA SRFEGNLEVA GQMADVIIAN PSGINIKGGG FINANKAIFT TGKPQLNADG SIKQFTVDQG KITVSANPNS
B7     KQFDVLAEGA VLNNSRTGAT TKTVGAVGAN PFLATGEARV ILNEVNSTAA SRFEGNLEVA GQMADVIIAN PSGINIKGGG FINANKAIFT TGKPQLNADG SIQQFNVSQG KVTITAPTNS
B3     KQFDVLAEGA VLNNSRQGAT TKTVGAVAAN PFLATGEARV ILNEVNSSAA SRFEGNLEVA GQMADVIIAN PSGINIKGGG FINANKAIFT TGKPQLNADG SIKQFTVDQG RITVSANPNS

       241                                                                                                            360
B3/B5  TFGLGGNNNN ANYVDIYARA LDLNAQLQAT NDIQVITGAN TVSSDLKNIA SITGTGAVPT LAVDVKALGG MYANNIYLMG TEKGLGVTNA GTIQAVSNLV ITSAGKIEHS GTISSTSKTQ
B5     KFGLGGNNND ANYVDLYTRA LELSAELRAK NDIQVIAGAN NVSADLQDVT AKTGTGTAQT LAVDVKALGG MYANNIYLMG TEKGLGVTNA GTLQAVNNLV ITSAGKIEHS GTISSTSKTQ
B7/B5  KFGLGGNNND ANYVDLYTRA LELSAELRAK NDIQVIAGAN NVSADLQDVT AKTGTGTAPT LAVDVKALGG MYANNIYLMG TEKGLGVTNA GTLQAVNNLV ITSAGKIEHS GTISSTSKTQ
B7     TFGLGGNNNN ANYVDIYARA LDLNAKLQAT NDIQVITGAN TVSSDLKNIA PISGAGTAPT LAIDVKALGG MYANNIYLMG TEKGLGVTNA GTLQAVNNLI ITSAGKIEHS GTISSTSKTQ
B3     KFGLGGNNND ANYVDLYARA LELSAELRAK NDIQVIAGAN NVSADLQEVT AKTGTGTAPA LAVDVKALGG MYANNIYLMG TEKGLGVTNA GTLQAVNNLV ITSAGKIEHS GTISSTSKTQ

       361                                                                                                            480
B3/B5  GLVSVQTTGV GVAADINSSG SINSNGMLNI DSGNNLNVNA KEIIINNGSL ASSPLIINAK GNLNLAANSR IFNDAQSGEL YVEAADLNLA TNAGITSNRG SAYIQSQKDI VAAQGAKLIA
B5     GLVSLQTTGT GAVADINSSG SINSNSMLNI DSGNNLNVNA KEIIINNGSL AASPLIISSK GNLNLAANSR IFNDAQSGDV YVDAANINLA TNAGITSNRG SAYIQSQKDV VAAQGAKLIA
B7/B5  GLVSLQTTGT GAVADINSSG SINSNSMLNI DSGNNLNVNV KEIIINNGSL ASSPLMINTK GNLNLAANSR IFNDAQSGDV YIDAANINMA TNAGITSNRG SAYIQSQKDV VAAQGAKLIA
B7     GLVSVQTTGV GAAADINSSG SINSNGMLNI DSGNNLNVNA KEIIINNGSL ASSPLIISSK GNLSLAANSR IFNDAQSGEL YVDAANINLA TNAGITSNRG SAYIQSQKDV VAAQGAKLIA
B3     GLVSVQTTGV GAAADINSSG SINSNSMLNI DSGNDLNVNA KEIIINNGSL AASPLIISSK GKVNLAANSR IFNDAQSGDV YVDAANINLA TNAGITSNRG SAYIQSQKDV VAAQGAKLIA

       481                                                                                                            600
B3/B5  AQNLNVSGKG KLSLNGNQIQ ASLGSINLQA DSSNTDGLID IQGGTIYAGK DLNLYSSGDV NLQNLGFSLE NSATRVKNIN AHSGRNLVWN NATKVLPQIT GKVALDAENN LSISAQGVSS
B5     AQNLNVSGKG KLSLNENQIQ ASLGSINLQA DSSNTDGLID IRGGTIYGGK DLNLYSSGDV NLQNLGFALE NSATRVKNIN AHSGRNLVWN NATKVLPQIT GKVALDAENN LSISAQGVSS
B7/B5  AQNLNVSGKG KLSLNGNQIQ ASLGSINLQA DSSNTDGLID IQGGTIYAGK DLNLYSSGDV NLQNLGFALE NSATRVKNIN GYSGRNLIWD NTAKALPQIS GKVVLEAENN LALTTTGLSS
B7     AQNLNVSGKG KLSLTGNQIQ ASLGSINLQA DSSNTDGLID IQGGTIYGGK DLNLYSSGDV NLQNLGFALE NSATRVKNIN AYSGRNLIWD NTAKALPQIS GKVVLEAENN LTLTATGLSS
B3     AQNLNVSGKG KLSLNENQIQ ASLGSINLQA DSSNTDGLID IRGGTIYGGK DLNLYSSGDV NLQNLGFALE NSATRVKNIN AHSGRNLVWN NATKVLPQIT GKVALDAESN LSISAQGVSN

       601                                                                                                            720
B3/B5  KDSIQLQAGQ LTLNTNFTSQ KDINLITETA DLFLNNVLNA QNDIKISALT GGITANSLKA NSNAGKISIL ATKNLVLNSL QEIESAPWAD QDGATTDMTF INGNKGVTLG SIGDGNVQLK
B5     KDSIQLQAGQ LTLNTNFTSQ KDINLITETT DLFLNNVLNA QNDINISALT GGITANSLKA NSNAGKISIL ANKNLVLNSL QEIESAPWAD QDGATTDMTF INGNKGVTLG SIGDGNVQLK
B7/B5  KDSIQLQGAQ LSLNSGLTSL NNISLTSEIA DLVLSNVLNA QNSIDVAALT GSITTKSLQA TSTGGKISLL AKKDLIINSV QTV--VPKWP TDELTTVVTH IKGQKGIDLG SIGDGGVSII
B7     KDSIQLQGAQ LSLNSGLTSL NNISLTSEIA DLVLSNVLNA QNNIDVSALT GSITTNSLQA TSTGGKISLL AKKDLIINSV QTV--LPKWP TDELTTVVTH IKGQKGIDLG SIGDGGVSII
B3     KDSIQLRAGQ LTLNTNLTSQ KDINVIAETT DLFLNNGLTA QNDINISALT GGITANSLKA NSNAGKISIL ANKNLVLNSL QEIESAPWAD QDGATTDMTF INGNKGVTLG SIGDGNVQLK

       721                                                                                                            840
B3/B5  STRITAEQGD IQLIAGNGIS LQANTDVTIR GDHGYDDIRK NILQGQSLQI QNKKADVVID NTTLTTSVGG LSINSEGKTT LSNSELNSKG NTELSAKDHL TLQNIEANAD KHMALSSKKN
B5     STRITAEQGD IQLVAGNGVS LQANTDVTIS GDSGYDNVRK NVLQGQSLQI QNKKADIVID STNLTTSVGG LSINGEGKTT LSNSELNSKG NIELSAKDHL TLQNIEANAD KHMALNSQKN
B7/B5  SSGLEAQQGD IQIISGNGLS ILPNYDVDVS ADDWYETSIS SALTAQNIGI NNKKGDLRIE DATLKATLGN LSISSDGKNT IRNVSLNSKG NMELFSKDHL TLQVVNANAD KHMALNSKKN
B7     SSGLEAQQGD IQIISGNGLN ILPNYDVEVN ADDWYERSIS STLTAQNIGI NNKKGDLRIE DATLTATFGD LSINSEGKSS IKAVSLNSKG NTELSAKDHL TMHAVTANAD KHMALSSKKN
B3     STRITAEQGD IQLVAGNGIS LQANTDVTIR GDHGYDDIRK NTLQGQSLQI QNKKADVVID NTTLTTSVGG LSINGEGKTT LSNSELNSKG NIELSAKDHL TLRNIEANAD KHMAVSSKKN

       841                                                                                                            960
B3/B5  IYINGEYG-- -TTTLWKPNI QSKLTSQGSL SMISAGSHAA QNTTYTGGAI LLEAGNVLTT PSNATLSF-- ----NAVDSA FLRIDPALKD LNGDLTIQTN GTLSLDPKIH KLSAAGDLEL
B5     IYLNSEQGPT VTSTIWESYS LVNLKAAGLL SLKSKGSQAL NSTSIKGGAI NIETDGSFNI NKDVQLNSAR NIFSNQESDK FLASDPQLQK IQGNLSIQTN NTLVLDPEKI KIQGDNNIEL
B7/B5  IYLNSGYGST TTPVVWDSYG LVNLTAKGIL STTSAD-HAA YRTTYTGGAV SLEANNLIS- PSDGTLSF-- ----NTVDST FLKNDPVLKD LNGDLTIQTN GALTIDPKIH KLNAIGDIEL
B7     IYLNMGYGST TTPVILDSYS SVNLKANGIL SIASAG-HAA YRTNYTGGAV SLEANNLIS- PSDGTLSF-- ----NTVDST FLKNDPVLKD LNGDLTIQTN GALTIDPKIH KFNAIGDIEL
B3     IYFNSKYGST ATPVVWDSSS SVNLKANGIL SMVGMD-HIA NQTTYTGGAI SLEANNLIS- PSAGTLTF-- ----NAMDSS VLTNDPALKN LEGDLTIQTN GALTLDPKIH KLGAAGDYEL

       961                                                                                                            1080
B3/B5  ISKNGKLSLL GYGGTNGNFS ETIFMIVN-G GGSHLEGNKG VEIQGAGIRS IKDFKIVAPD GDIKIEGVKN TFKNSSYAKH ALDLAHQRLY YENRIQALNS LGTLTAAQKK ----------
B5     ISKNGDLTLL GYGGSKGNGS ERNFTISG-T GDIHVEGKK- VDIQGAFI-S GRNINIFSSS GDINIDGIKN NFNNYASKKY SSELFEKIF- --NLTMILSN TESLKKYSAS ----------
B7/B5  ISKNGALTLK GYEGTVGNGS EQVVKLNTVG GGISLEGTK- VDLQGAQLTA QKDIKLISSK EDIFIDGIKN KFTNFASIKY IASLNQNLAD LNAKINIIKN DPSYLSTKDK IASLGSQASQ
B7     ISKNGTLTLK GYEGIAGNGS EQVIKLNTVG GGISLEGTK- IDLQGSQLTA QKDIKLISSK EDILIDGIKN KFTNIASIKH IASLNQNLAD LNAKINIIKN DPTYLSTKDK IASLGSQASQ
B3     ISKNGKLTLL GYGGTNGNFS ETVFRLTNNM GGSHLEGNKG VEIQGTVIRN MKDLKIIAPD GDIKIEGVKN TFNNSTYSKH SDYLFKQRLY YENKIQEINR LGVLNTAQKK ----------

       1081                                                                                                           1200
B3/B5  ---------- ---------- ---------- ---------- ---------- ---------- ---------- -------LVA QYTEEINDIT QQVNFLRSGT NGAEHKGSLL TSETGK--VN
B5     ---------- ---------- ---------- ---------- ---------- ---------- ---------- -------RIS QMQEELADLK DASTFLENGT NGFEHAETVL RTSLGDGIIN
B7/B5  INQLLNDALS AYTSGVDPLN ASIGYDTENG NYIYVSNDDG GRAYFVSIPS GLDLTRYRQI VLNELPAENQ KLKNFNNKIN AFEQDKARVN QTINFMNNKT NGYEHAESTL TSKAGN--IS
B7     INQLLNDALS AYTSGVDPLN ASIGYDSENG NYIYVSNDDG GRAYFVSIPS GLDLSRYRQI VLNELPAENQ KLNNFNNKIT AFEQDKARVN QTITFMNNKT NGYEHAKSTL TSKAGN--IS
B3     ---------- ---------- ---------- ---------- ---------- ---------- ---------- -------LVA QYTEEINDIT KQINFLSLGT NGAEHAGSLL RSETGK--VN

       1201                                                                                                           1320
B3/B5  LVSGKGIMIT GADIRA-VDG EVNIESQGIG EKVYSSTALD KNGQ-PMIMN AGAILAAHND FYDRGNETDQ NYQAISLLTP TLVEGRRGVN IKTTGTQEGS HLVLQAVGIS SSNGDVKIES
B5     LISKKGIKIT GATIQAGYDG KVNIEAQGGS DKIYNSTVLN KNNQ-PINMN ASIIIAGHND FYDKGNDTDS NYKAISFVSP TTIEGA-DIN IKTVGNSAGD NLVLQGVGIS AKN-QVKIEA
B7/B5  LTGARGVSIS GANLTA-AAG QVNIEARSPL LSQYTSSTIN SGQTLPRTLS ASIIIDGHTD FYDKGNETDA NYSMRTFISP TIINGTKGVN IKTIGNTASD NLILQATGIT ASQGDVRIEA
B7     LTAARGISIS GANLTA-TAG QANIEARAPL VSQYTSSTIN SGQTLPRTLS ASIIIDGHTD FYDKGNEADA NYSMRTFISP TIINGTKGVN IKTIGNTASD NLVLQATGIT ASQGDVKIEA
B3     IVSGKGIMIT GADIMA-IDG QVNIEAQGIG EKIYTSTALD KNSQ-PMSMN AGVILAAHND FYDRGNETDQ NYQAISLLTP TLVEGKRGVN IKTTGAQEGS NLILQAVGIS SSEGDVKIES

       1321                                                                                                           1440
B3/B5  NKNILFDVAL EQSYDRSTSV EKKKSWGGLK KKTITTFSEN ESSRPASVDI SGNNIYIESK EL-------N KNHKVDIYSG QFIAEGGDVF IRSGGDLYFN TVNEISKNQL EINKKSSILG
B5     KKNILFDVAL EQIFDRSTKV QKKKSWGGLK KKTITTVMEN ELIRPASVDI DAKNIYIESK EV-------N PQNSIDIYGG KLTANGGMIS VRSGGNLNFY TAPESSKTTI DVNSKSSIMG
B7/B5  NKSMLFDAAV EQSYDRSTST TTKKSWGGLK KKITTTIEET NITNAASVDI SGKNIYIETK KLVAGVDPTK PDNNIDIYSG RFTAEGGQIS IKSGGNLNFY TAEETSSSKV DITKKSSFAG
B7     NKSMLFDAAV EQSYDRSTST TTKKSWGGLK KKITTTIEET NITNAASVDI SGKNIYIETK KLATDVDPTK PDNNIDIYSG RFTAEGGQIS IISGGNLNFY TAEETSSSKV DITKKSSFAG
B3     NKNILFDVAL EQIYDRSTSI EKKKSWGGLK KKTITTFSEN ESTRPASVDI SGKNIYIESK EL-------N KNNKIDIYSG QFIAEGGDVF IRSGGDLFFN TVNEISKNQL EVNKKSSILG

       1441                                                                                                           1560
B3/B5  IKYNTSKTNS SYQVVTQIPS TIAAAYIGTK SGFDTHLAGT EFNYLKEATI ESGGKIFLEP SVTSINNTIK KEKNSVVWQS MQDKGSITET AKLPSFNGPT PPIFKATGGL SVQIPISEKD
B5     IKYNSSKTNS THNVVTQIPT KLVADYIGTK SGFDTRLVGT DFEYLKGATI ESGGKIFIEP AITNISTKLK KESNSVVWQS MQDKGSITET AKLPNFNGPV APTFKAAGGL SVQIPISEKD
B7/B5  IKYNDSKTNA TRTQISELPA VLKADYIGTK SGFDTRLVGT EFEYLKGAQI EAGGKIELLA ATKQITDLVK KEKNSVVWQS MQDKGSITET AKLPSFNGPV APTFKAAGGL VVQVPISETD
B7     IKYNDSKTNA TRTQISELPA VLKADYIGTK SGFDTKLVGT EFEYLKGAHI EAGGKIELLA ATKQITDLLK KEKNSVVWQS MQDKGSITET AKLPSFNGPV APTFKAAGGL LVQVPISEVD
B3     IKYNTSKTNS SYQVVTQVPS TLTASYIGTK SGFDTHLAGT EFNYLKEATI ESGGKIFLEP SVTSINNTIK KEKNSVVWQS MQDKGSITET AKLPSFNGPV APIFKAAGGL AVQVPVKD--

                                         |------------switch region---------------------|

       1561                                                                                                           1680
B3/B5  ANKIVLRDEI LKLANQPGNT YLKDLVNRND VDWQKVLLTQ KDWDYKSQGL TGAGAALIVI IVTIATMGTG TAAAAGAAGG TAASGTTV-- ---------G LGASMLG-TA GVTTTAAGAI
B5     ANKIVLRDEI LKLANQPGNT YLKDLVNRND VDWQKVLLTQ KDWDYKSQGL TGAGAALIVI IVTIATMGTG TAAAAGAAGG TAASGTTV-- ---------G LGASMLG-TA GVTTTAAGAI
B7/B5  ANKVELRNEI LKLANQPGNA YLKDFINRND VNWQQILLAQ KDWDYKSQGL TGAGAAILVI IVTVVTMGAG TAAGAALFGG T--------- ---------- ---------- ----------
B7     ANKVELRDEI LKLANQPGNA YLKSFVNRND VNWQQILLAQ KEWDYKSQGL TGAGAAIIAI IVAIATYGAG VAALGTTTVA ASGGATTVTV GTTTVAVGAG GSATAFGGTV LATTTAAGVT
B3     ----DVRSAL DTLVNQQGTT YLKDLVLRND VDWQAVKLAQ QQWDYKSQGL TPAAAAIIVI IITVATYGYG AQGAGAALF- T--------- ---------- ---------- ----------

       1681                                                                                                           1800
B3/B5  VPSTLGAMAN AAVTSLAAQA SIGLINNGGD IGKTLKEMGS KDSIKNLAAS VVTAGLLSQV GTALNLKP-D STLLSDRLVN NFTTSVGSTL VQ-------- TAINGGNLGD NLQVALLAGL
B5     VPSTLGAMAN AAVTSLAAQA SIGLINNGGD IGKTLKEMGS KDSIKNLAAS VVTAGLLSQV GTALNLKP-D STLLSDRLVN NFTTSVGSTL VQ-------- TAINGGNLGD NLQVALLAGL
B7/B5  TTTTTTAMAN AFVTSLTSQA SVSLVNNGGD IGKTLKELGS KNSIRNLAAS VVTAGLLSQV STALNLKP-D STLLSDRLIN NFTTSVGSTL VQ-------- TAINGGDLQD NLEKALLAGL
B7     TYTTTGIMIN AALTSLASQA SVSLVNNQGN VAQTLKDLGS KDTVKNLATS VVTAGLLDKI GGSNWMQQFN GTGVTDRIVT NLVNSTGSAL VS-------- TAINGGDLND NLQTALLGGL
B3     SSATSSAMAN AAVASMASQA AISLANNGGD PLKALKSLGS SEYIKSLAVS VLSAGALSAV GGTAMMQNLE KLKQSGDLVQ KIVGNTAQAL VNSTIGAGIN TAVSGNSLSE NLENYLASGA

       1801                                                                                                           1920
B3/B5  AGALQGELAS QIGTSLDKVD PNIFEYTIHK IAHAAVGCAA AAATKSSCEA GAIGAGVGEI VAGLMPEPA- -NGIEYTDAE KLKIRNIGKL VSGTVAAYTG YDVNTAANSA DTAIQNNSLV
B5     AGALQGELAS QIGTSLDKVD PNIFEYTIHK IAHAAVGCAA AAATKSSCEA GAIGAGVGEI VAGLMPEPA- -NGIEYTDAE KLKIRNIGKL VSGTVAAYTG YDVNTAANSA DTAIQNNSLV
B7/B5  AGALQGELAS QIGTNLDKVD PNIFEYTIHK IAHAAVGCAA AAVTKSSCEA GAIGAGVGEI VAGLMPEPA- -NGIEYTDAE KLKIRNIGKL VSGTVAAYTG YDVNTAANSA DTAIQNNSLV
B7     ATAVQGYLSN NI-SGIQQGS NFDLDYILHK VAHAAAGCLA GSIQKD-CAA GAIGAALGEV FADTIT--G- -NKYDYTDAQ LNAIKEGSKA LAATVAAYAG YDVNIAISSA DTALTNNWLG
B3     MTALQGTMAQ YIKPLESQG- -FSIEYVLHK IAHAAAGCGA AAATKQSCEA GAIGAGVSEV AAEYLRKPRV FESVDDALAY KNQIVEVSKG IAGAVAIVTG YDSNTAMASA DNALKNNFVF

       1921                                                                                                           2040
B3/B5  RLATTTGKLL VKAAEIYGDI KKANKGIVDS KVFVQKLKDQ GVSELV--GI ADDLVTVFGP ASTPFDRALA AFDLIIGTDL KPGKAESLKL A-QAELDKLK --VDQAYTLK VLQVKKDVVT
B5     RLATTTGKLL VKAAEIYGDI KKANKGIVDS KVFVQKLKDQ GVSELV--GI ADDLVTVFGP ASTPFDRALA AFDLIIGTDL KPGKAESLKL A-QAELDKLK --VDQAYTLK VLQVKKDVVT
B7/B5  RLATTTGKLL VKAAEIYGDI KKANKGIVDS KVFVQKLKDQ GVSELV--GI ADDLVTVFGP ASTPFDRALA AFDLIIGTDL KPGKAESLKL A-QAELDKLK --VDQAYTLK VLQVKKDVVT
B7     TQQKKQRELE YIKANALGDA AIFAK-YGGI SVLQDRLTQG GFAAGVAAGA GSDIWNVGKA ILSPIDTLNG LKAIIADSDV RAQLTQSAIT AFNTKIQRIE NNYNTGGLKE ATQLGYDLGT
B3     LAAVPTA--- --GEALVGLT LGAIRTCATN AACVSRAAQL GISVSAT-LA ANSNSTDDKK VVKPVPNTSG SETGTPNNGD DQNDGKQAKK LSNNDIVDLT KNPNWHKTSL KSRIAAAYAR

       2041                                                                                                           2160
B3/B5  RNR-----LN GKEFEMSAAS KLG-VKRNTD RQMITIKTER DGQINIIPDA FGNGGTLVEF KNVQYLTDTQ QLRGYGATGK PVTLVVNMNT KISETVKNSI LSNKGTIQRF DPIKNTLHSY
B5     RNR-----LN GKEFEMSAAS KLG-VKRNTD RQMITIKTER DGQINIIPDA FGNGGTLVEF KNVQYLTDTQ QLRGYGATGK PVTLVVNMNT KISETVKNSI LSNKGTIQRF DPIKNTLHSY
B7/B5  RNR-----LN GKEFEMSAAS KLG-GKRNTD RQMITIKTER DGQINIIPDA FGNGGTLVEF KNVQYLTDTQ QLRGYGATGK PVTLVVNMNT KISETVKNSI LSNKGTIQRF DPIKNTLHSY
B7     LTWEVVGILT VVKGAATATA KMGSA-GLKI TTTIAETAKQ SAKYQAPTTS IISAAQTQKV ISDVKTIPVV TFNGVVLDPR LPAPVAGYAY KPKVIDSPNP NIANSHVNGY MGELRLANEV
B3     ELR------G SKNFDFYRDP KTGDIFIKGN QSNNMVKIDI SRFLK

       2161                                                                                                    2275
B7     AAQPNQAVLK FGGDLTHHGS DIISVDVKTG GVYLWDSKYR SANSKLGAST TFTNSTTRTN AVNEALTTIR QSNLPESVKQ VALKNLNDGT FTTYTVATGN SKNSVIFSCV NKVCK
```

Supplementary Fig. 6. Coiled-coil conformation of type-I CdiA proteins predicted by the MARCOIL program

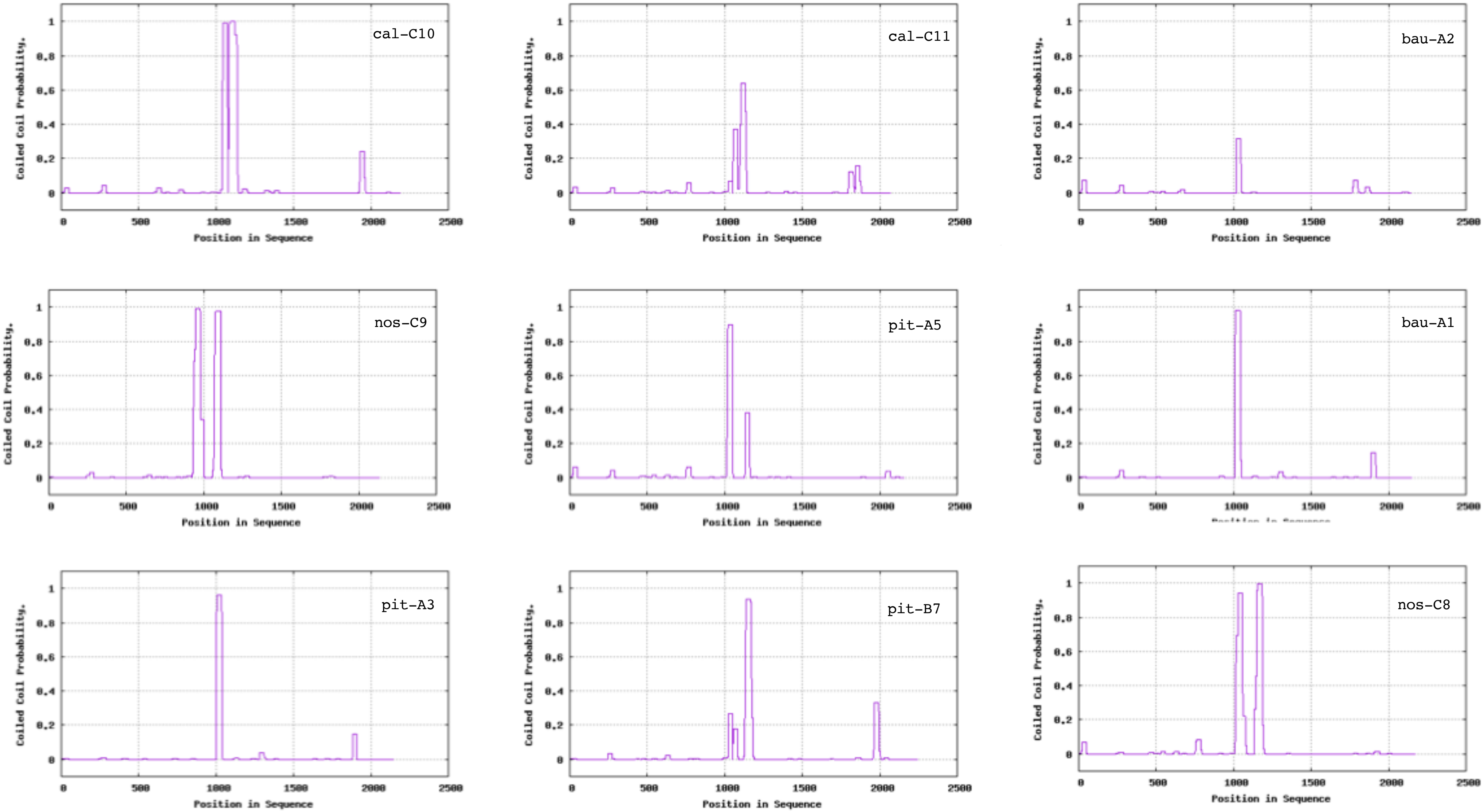

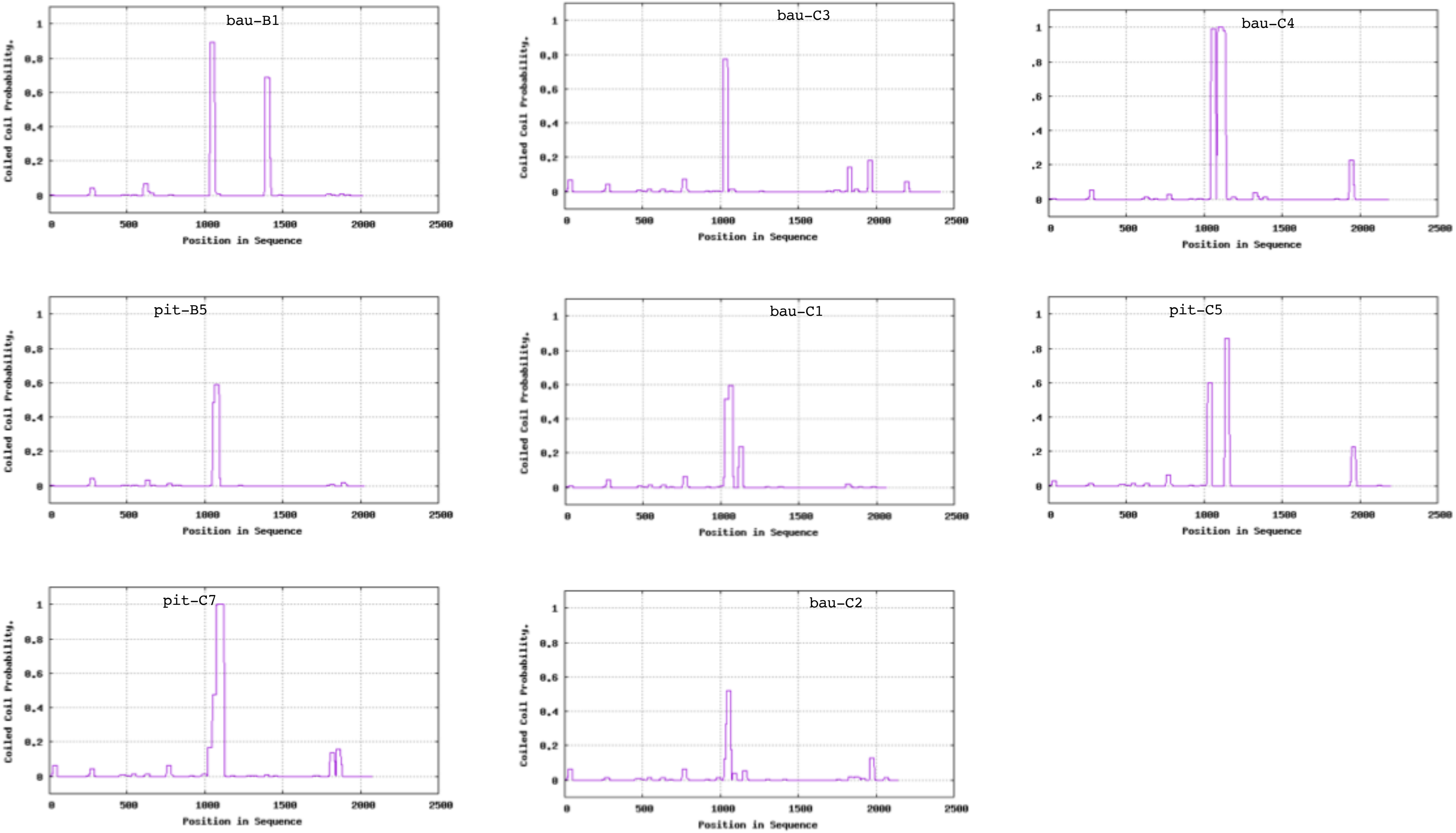

bau-B1
bau-C3
bau-C4
pit-B5
bau-C1
pit-C5
pit-C7
bau-C2
Coiled Coil Probability.
Position in Sequence
0
500
1000
1500
2000
2500
0
0,2
0,4
0,6
0,8
1

Supplementary file 7. Acinetobacter pittii strains carrying multiple *cdi*A genes

pit-A3	pit-A4	pit-A5	pit-A4/C7/A4	pitA5/A4/A5	pitA5/C7/A5	pitA5/C8/A5	pit-A5/n2/A5	pit-B7/pit-B5	pit-B5	pit-C6	pit-C5	bau-B3/B5	pit-B7	pit-B6	strain	ST	Place of isolation
							●		●	●					DSM 25618	63	unknown
							●		●	●					ATCC19004	63	unknown
							●		●	●					St-10592-91	64	Germany Cologne
							●		●	●					St-20772-90	64	Germany Cologne
							●		●	●					St-14379-92	64	Germany Cologne
							●		●	●					DSM 9306	64	unknown
							●		●	●					UKK-0548	248	Germany
							●		●	●					UKK-0556	248	Germany Bochum
							●		●	●					PR386	248	unknown
							●		●	●					ARLG 1839	396	unknown
							●		●	●					PR368	744	unknown
									●	●					UKK-0547	63	Germany Dresden
									●	●					UKK-0327	64	Germany mainz
									●	●					PR313	64	unknown
									●	●					PR370	64	unknown
									●	●					ARLG 1803	64	unknown
			●						●	●					St-12537-91	70	Germany Cologne
									●	●					SH024	93	unknown
									●	●					ABBL10	93	USA Chicago
									●	●					ABBL047	93	USA Chicago
									●	●					ABBL065	93	USA Chicago
									●	●					ABBL103	93	USA Chicago
									●	●					St-11469-92	93	Germany Cologne
									●	●					UKK-0245	93	Germany Berlin
									●	●					UKK-0544	93	Germany Braunschweig
									●	●					UKK-0555	93	Germany
									●	●					St-15639-92	93	Germany Cologne
									●	●					St-15559-91	93	Germany Cologne
									●	●					St-14569-91	93	Germany Cologne
									●	●					ABUH403	93	USA
									●	●					ARLG 1776	93	unknown
									●	●					ARLG 1798	93	unknown
									●	●					IHIT32685	93	Germany
									●	●					IHIT32473	93	Germany
									●	●					UBA2478	396	USA New York
									●	●					ARLG 1768	396	unknown
									●	●					NBRC 110510	1172	Japan Hyogo
									●	●					PR339	744	unknown
									●	●					XH765	nd	China Hangzhou
									●	●					ARLG 1771	1173	unknown
		●							●						St-19650-90	747	Germany Cologne
		●							●						ARLG 1875	nd	unknown
		●							●						ABUH540	1175	USA Ohio
		●							●						ARLG 1950	nd	unknown
							●		●						NBRC 110508	1178	Japan Osaka
					●				●						PR301	457	unknown
											●	●			NBRC 110506	220	Japan Gifu
											●	●			1295259	119	USA
											●	●			PR348	119	unknown
											●	●			CR12-42	119	Australia
											●	●			269	119	Malaysia
	●											●			NBRC 110504	119	Japan Osaka
	●											●			NBRC 110507	119	Japan Osaka
	●											●			NBRC 110509	119	Japan Hyogo
	●							●			●				ABBL111	119	USA Chicago
	●							●							PR320	119	unknown
								●			●				IPK_TSA6.1	321	South Korea
								●			●				UBA2484	1171	USA New York
								●			●				ARLG 1957	1169	unknown
								●			●				ABBL024	643	USA Chicago
				●								●			TCM292	207	China Hangzhou
				●								●			907131	220	USA
				●				●							FC8876	643	Rio de Janeiro
							●			●					St-12828-92	744	Germany Cologne
		●										●			UKK-0546	776	Germany Munich
●														●	IEC338SC	1076	Brazil
				●							●				ABBL103	93	USA Chicago
				●									●		ABBL033	1170	USA Chicago
						●								●	ARLG 1933	1165	unknown

Supplementary file 8. Type-II CdIA alignment.

Residues color: red, high consensus value (90%); blu, moderate consensus value(50%)

PT-VENN motifs, PT-LPEN motifs and the FhaB domain are highlighted in green,turquoise and grey,respectively.

```
         1                                                                                                                           120
 bau-D1  MNKNRYRIIF SHARGMFIAV AEIVKSKTKQ AGQSQGQVGT DSTIDSVLPI -------HYK KLNPLNFAVI GCLGALVISL PMSSVAETQI IADKGAPTSQ QPTILNSANG TTQVNIQTPS
 nos-D2  MNKNRYRIIF SHARGMFIAV AEIVKSKTKQ AGQSQGQIGT DSTIDSVLPI -------HYK KLNPLNFAVI GCLGALVISL PMSSVAETQI IADKGAPTSQ QPTILNSANG TTQVNIQTPS
 bau-D3  MNKNRYRIIF SHARGMFIAV AEIVKSKTKQ AGQSQGQVGT DSTIDSVLPI -------HYK KLNPLNFAVI GCLGALVISL PMSSVAETQI IADKGAPTSQ QPTILNSANG TTQVNIQTPS
 nos-D4  MNKNRYRIIF SHARGMFIAV AEIVKSKTKQ AGQSQGQIGT DSTIDSVLPI -------HYK KLNPLNFAVI GCLGALVISL PMSSVAETQI IADKGAPTSQ QPTILNSANG TTQVNIQTPS
 bau-D7  MNKNRYRIIF SHARGMFIAV AEIVKSKTKQ AGQSQETMET GVVTSSVPTI -------HYK KLNPLNFAVI GCLGALVISL PMSSVAGTQI IADKGAPTSQ QPTILNSANG TTQVNIQTPS
 bau-D5  MNKNRYRIIF SHARGMFIAV AEIVKSKTKQ AGQSQGQVGT DSTIDSVLPI -------HYK KLNPLNFAVI GCLGALVISL PMSSVAETQI IADKGAPTSQ QPTILNSANG TTQVNIQTPS
 nos-D8  MNKNRYRIIF SHARGMFIAV AEIVKSKTKQ AGQSQGQIGT DSTIDSVLPI -------HYK KLNPLNFAVI GCLGALVISL PMSSVAETQI IADKGAPTSQ QPTILNSANG TTQVNIQTPS
 bau-D9  MNKNRYRIIF SHARGMFIAV AEIVKSKTKQ AGQSQGMMEI DSVTSSVRLI -------HYK KLNPLNFAVI GCLGALVISL PMSSVAETQI IADKGAPTSQ QPTILNSANG TTQINIQTPS
 bau-D6  MNKNRYRIIF SHARGMFIAV AEIVKSKTKQ AGQSQGQVGT DSTIDSVLPI -------HYK KLNPLNFAVI GCLGALVISL PMSSVAETQI IADKGAPTSQ QPTILNSANG TTQVNIQTPS
nos-D12  MNKNRYRIIF SHARGMFIAV AEIVKSKTKQ AGQSQGQVGT DSTIDSVLPI -------HYK KLNLLNFAVI GCLGALVISL PMSSVAETQI IADKGAPTSQ QPTILNSANG TTQVNIQTPS
bau-D10  MNKNRYRIIF SHARGMFIAV AEIVKSKTKQ AGQSQGMMET DSVTSSVRPI -------HYK KLNPLNFAVI GCLGALVISL PMSSVAETQI IADKGAPTSQ QPTILNSANG TTQVNIQTPS
bau-D11  MNKNRYRIIF SHARGMFIAV AEIVKSKTKQ AGQSQGMMET DSVTSSVRPI -------HYK KLNPLNFAVI GCLGALVISL PMSSVAETQI IADKGAPTSQ QPTILNSANG TTQVNIQTPS
bau-D14  MNKNRYRIIF SHARGMFIAV AEIVKSKTKQ AGQSQGQVGT DSTIDSVLPI -------HYK KLNPLNFAVI GCLGALVISL PMSSVAETQI IADKGAPTSQ QPTILNSANG TTQVNIQTPS
bau-D12  MNKNRYRIIF SHARGMFIAV AEIVKSKTKQ AGQSQGMMET DSVTSSVRPI -------HYK KLNPLNFAVI GCLGALVISL PMSSVAETQI IADKGAPTSQ QPTILNSANG TTQVNIQTPS
bau-D20  MNKNRYRIIF SHARGMFIAV AEIVKSKTKQ AGQSQGMMET DSVTSSVRPI -------HYK KLNPLNFAVI GCLGALVISL PMSSVAETQI IADKGAPTSQ QPTILNSANG TTQVNIQTPS
bau-D15  MNKNRYRIIF SHARGMFIAV AEIVKSKTKQ AGQSQGQVGT DSTIDSVLPI -------HYK KLNPLNFAVI GCLGALVISL PMSSVAETQI IADKGAPTSQ QPTILNSANG TTQVNIQTPS
bau-D16  MNKNRYRIIF SHARGMFIAV AEIVKSKTKQ AGQSQGQVGT DSTIDSVLPI -------HYK KLNPLNFAVI GCLGALVISL PMSSVAETQI IADKGAPTSQ QPTILNSANG TTQVNIQTPS
bau-D21  MNKNRYRIIF SHARGMFIAV AEIVKSKTKQ AGQSQGQVGT DSTIDSVLPI -------HYK KLNPLNFAVI GCLGALVISL PMSSVAETQI IADKGAPTSQ QPTILNSANG TTQVNIKTPS
bau-D19  MNKNRYRIIF SHARGMFIAV AEIVKSKTKQ AGQSQGQVGT DSTIDSVLPI -------HYK KLNPLNFAVI GCLGALVISL PMSSVAETQI IADKGAPTSQ QPTILNSANG TTQVNIQTPS
bau-D13  MNKNRYRIIF SHARGMFIAV AEIVKSKTKQ AGQSQGQVGT DSTIDSVLPI -------HYK KLNPLNFAVI GCLGALVISL PMSSVAETQI IADKGAPTSQ QPTILNSANG TTQVNIQTPS
cal-D23  MNKNRYRIIF SHARGMFIAV AEIVKSKTKQ AGQSQGTMET GSVTSSVPPI -------HYK KLNPLNFAVI GYLGALVISL PMSSVAGTQI IADKGAPTSQ QPTILNSANG TTQVNIQTPS
pit-D20  MNKNRYRIIF SHARGMFIAV AEIVKSKTKQ AGQSQGTMET GSITSSVSPI -------HYK KLNPLNFAVI GCLGALVISL PMSSVAGTQI IADKGAPTSQ QPTILNSANG TTQVNIQTPS
 pit-D6  MNKNRYRIIF SHARGMFIAV AEIVKSKTKQ AGQSQGTMET GSITSSVSPI -------HYK KLNPLNFAVI GCLGALVISL PMSSVAGTQI IADKGAPTSQ QPTILNSANG TTQVNIQTPS
bau-D17  MNKNRYRIIF SHARGMFIAV AEIVKSKTKQ AGQSQGTMET GVVTSSVPTI -------HYK KLNPLNFAVI GCLGALVISL PMSSVAGTQI IADKGAPTSQ QPTILNSANG TTQVNIQTPS
nos-D18  MNKNRYRIIF SHARGMFIAV AEIVKSKTKQ AGQSQGVSGT DSTTVSVLPI -------HYK KLNPLNFAVI GCLGALVISL PMSSVAETQI SADKGAPTSQ QPTILNSANG TTQVNIQTPS
nos-D22  MNKNRYRIIF SHARGMFIAV AEIVKSKTKQ AGQSQGVSGT DSTTVSVLPI -------HYK KLNPLNFAVI GCLGALVISL PMSSVAETQI SADKGAPTSQ QPTILTSANG TTQVNIQTPS
 bau-D2  MNKNRYRVIF SQARGMFIAV AEIVKSRTKM AGQSIAAGNE TVTNSLTPNS --------YK KLNPLHFSVV TLLGAVVYTI PLSSIANTQI HADKSAPSNQ QAQILNSANG TVQVNIQTPS
bay-D15  MNKNRYRIIF SQARGMFIAV AEIVKSKTKQ AGQSQADGHD NKACSASDSD SMGFDSSHLK KLKQLNFAVI SVLGASIFTL PLNTIADSQI IADKGAPTSQ QPTILNSSNG VTQVNIQTPS

         121                                                                                                                         240
 bau-D1  AGGVSRNTYT QFDVGQEGAI LNNSRNNTQT QLGGWVQGNP WLAKGEAKVI LNEVNSNNPS QLKGYIEVAG KQAQVVIANP SGLICDGCGV INADRFTLTT GQAVMNQGYL ESFRVREGQV
 nos-D2  AGGVSRNTYT QFDVGQEGAI LNNSRNNTQT QLGGWVQGNP WLAKGEAKVI LNEVNSNNPS QLKGYIEVAG KQAQVVIANP SGLICDGCGV INADRFTLTT GQAVMNQGYL ESFRVREGQV
 bau-D3  AGGVSRNTYT QFDVGQEGAI LNNSRNNTQT QLGGWVQGNP WLAKGEAKVI LNEVNSNNPS QLKGYIEVAG KQAQVVIANP SGLICDGCGV INADRFTLTT GQAVMNQGYL ESFRVREGQV
 nos-D4  AGGVSRNTYT QFDVGQEGAI LNNSRNNTQT QLGGWVQGNP WLAKGEAKVI LNEVNSNNPS QLKGYIEVAG KQAQVVIANP SGLICDGCGV INADRFTLTT GQAVMNQGYL ESFRVREGQV
 bau-D7  AGGVSRNTYT QFDVGQEGAI LNNSRNNTQT QIGGWVQGNP WLATGEAKVI LNEVNSSNPS QLKGYIEVAG KQAQVVIANP SGLICDGCGV INADRFTLTT GQAVMNQGYL ESFRVREGQV
 bau-D5  AGGVSRNTYT QFDVGQEGAI LNNSRNNTQT QLGGWVQGNP WLAKGEAKVI LNEVNSNNPS QLKGYIEVAG KQAQVVIANP SGLICDGCGV INADRFTLTT GQAVMNQGYL ESFRVREGQV
 nos-D8  AGGVSRNTYT QFDVGQEGAI LNNSRNNTQT QLGGWVQGNP WLAKGEAKVI LNEVNSNNPS QLKGYIEVAG KQAQVVIANP SGLICDGCGV INADRFTLTT GQAVMNQGYL ESFRVREGQV
 bau-D9  AGGVSRNTYT QFDVGQEGAI LNNSRNNTQT QIGGWVQGNP WLATGEAKVI LNEVNSSNPS QLKGYIEVAG KQAQVVIANP SGLICDGCGV INADRFTLTT GQAVMNQGYL ESFRVREGQV
 bau-D6  AGGVSRNTYT QFDVGQEGAI LNNSRNNTQT QLGGWVQGNP WLAKGEAKVI LNEVNSNNPS QLKGYIEVAG KQAQVVIANP SGLICDGCGV INADRFTLTT GQAVMNQGYL ESFRVREGQV
nos-D12  AGGVSRNTYT QFDVGQEGAI LNNSRNNTQT QLGGWVQGNP WLAKGEAKVI LNEVNSNNPS QLKGYIEVAG KQAQVVIANP SGLICDGCGV INADRFTLTT GQAVMNQGYL ESFRVREGQV
bau-D10  AGGVSRNTYT QFDVGQEGAI LNNSRNNTQT QIGGWVQGNP WLATGEAKVI LNEVNSSNPS QLKGYIEVAG KQAQVVIANP SGLICDGCGV INADRFTLTT GQAVMNQGYL ESFRVREGQV
bau-D11  AGGVSRNTYT QFDVGQEGAI LNNSRNNTQT QIGGWVQGNP WLATGEAKVI LNEVNSSNPS QLKGYIEVAG KQAQVVIANP SGLICDGCGV INADRFTLTT GQAVMNQGYL ESFRVREGQV
bau-D14  AGGVSRNTYT QFDVGQEGAI LNNSRNNTQT QLGGWVQGNP WLAKGEAKVI LNEVNSNNPS QLKGYIEVAG KQAQVVIANP SGLICDGCGV INADRFTLTT GQAVMNQGYL ESFRVREGQV
bau-D12  AGGVSRNTYT QFDVGQEGAI LNNSRNNTQT QIGGWVQGNP WLATGEAKVI LNEVNSSNPS QLKGYIEVAG KQAQVVIANP SGLICDGCGV INADRFTLTT GQAVMNQGYL ESFRVREGQV
bau-D20  AGGVSRNTYT QFDVGQEGAI LNNSRNNTQT QIGGWVQGNP WLATGEAKVI LNEVNSSNPS QLKGYIEVAG KQAQVVIANP SGLICDGCGV INADRFTLTT GQAVMNQGYL ESFRVREGQV
bau-D15  AGGVSRNTYT QFDVGQEGAI LNNSRNNTQT QLGGWVQGNP WLAKGEAKVI LNEVNSNNPS QLKGYIEVAG KQAQVVIANP SGLICDGCGV INADRFTLTT GQAVMNQGYL ESFRVREGQV
bau-D16  AGGVSRNTYT QFDVGQEGAI LNNSRNNTQT QLGGWVQGNP WLAKGEAKVI LNEVNSNNPS QLKGYIEVAG KQAQVVIANP SGLICDGCGV INADRFTLTT GQAVMNQGYL ESFRVREGQV
bau-D21  AGGVSRNTYT QFDVGQEGAI LNNSRNNTQT QLGGWVQGNP WLAKGEAKVI LNEVNSNNPS QLKGYIEVAG KQAQVVIANP SGLICDGCGV INADRFTLTT GQAVMNQGYL ESFRVREGQV
bau-D19  AGGVSRNTYT QFDVGQEGAI LNNSRNNTQT QLGGWVQGNP WLAKGEAKVI LNEVNSNNPS QLKGYIEVAG KQAQVVIANP SGLICDGCGV INADRFTLTT GQAVMNQGYL ESFRVREGQV
bau-D13  AGGVSRNTYT QFDVGQEGAI LNNSRNNTQT QIGGWVQGNP WLATGEAKVI LNEVNSSNPS QLKGYIEVAG KQAQVVIANP SGLICDGCGV INADRFTLTT GQAVMNQGYL ESFRVREGQV
cal-D23  AGGVSRNTYT QFDVGQEGAI LNNSRNNTQT QIGGWVQGNP WLATGEAKVI LNEVNSSNPS QLKGYIEVAG KQAQVVIANP SGLICDGCGV INADRFTLTT GQAVMNQGYL ESFRVREGQV
pit-D20  AGGVSRNTYT QFDVGQEGAI LNNSRNNTQT QIGGWVQGNP WLATGEAKVI LNEVNSSNPS QLKGYIEVAG KQAQVVIANP SGLICDGCGV INADRFTLTT GQAVMNQGYL ESFRVREGQV
 pit-D6  AGGVSRNTYT QFDVGQEGAI LNNSRNNTQT QIGGWVQGNP WLATGEAKVI LNEVNSSNPS QLKGYIEVAG KQAQVVIANP SGLICDGCGV INADRFTLTT GQAVMNQGYL ESFRVREGQV
bau-D17  AGGVSRNTYT QFDVGQEGAI LNNSRNNTQT QIGGWVQGNP WLATGEAKVI LNEVNSSNPS QLKGYIEVAG KQAQVVIANP SGLICDGCGV INADRFTLTT GQAVMNQGYL ESFRVREGQV
nos-D18  AGGVSRNTYT QFDVGQEGAI LNNSRNNTQT QLGGWVQGNP WLAKGEAKVI LNEVNSNNPS QLKGYIEVAG KQAQVVIANP SGLICDGCGV INADRFTLTT GQAVMNQGYL ESFRVRKGQV
nos-D22  AGGVSRNTYT QFDVGQEGVI LNNSRNNTQT QLGGWVQGNP WLAKGEAKVI LNEVNSNNPS QLKGYIEVAG KQAQVVIANP SGLICDGCGV INADRFTLTT GQAVMNQGYL ESFRVREGQV
 bau-D2  AGGVSRNTYS QFDVGQQGAI LNNSRNNVQT QIGGWVQGNA NLARGEAKVI LNEVNSSNPS QLRGYLEVAG KSAQVVIANP SGLVCDGCGV INADRFTLTT GQAVMNQGYL ESFRVREGQV
bay-D15  AGGVSRNTYS QFDVGQEGAI LNNARNNTQT QLGGWIQGNP WLAKGEAKVI LNEVNSSNPS QLKGYIEVAG KQAQVVIANP SGLVCDGCGV INADRFTLTT GQAVMNQGYL ESFRVREGQI

         241                                                                                                                         360
 bau-D1  TIEGKGLNGS LTPYTDIYAR ALKVNAGLYA NELNTVLGQN DIQVKDQVAP QIT-ATTAAT TTPPPNFALD VGQLGGMYAG KIFLVGTEQG LGVRNAGTIN STQSTLTLNA NGDLVNNGNL
 nos-D2  TIEGKGLNGS LTPYTDIYAR ALKVNAGLYA NELNTVLGQN DIQVKDQVAP QIT-ATTAAT TTPPPNFALD VGQLGGMYAG KIFLVGTEQG LGVRNAGTIN STQGTLTLNA NGDLVNNGNL
 bau-D3  TIEGKGLNGS LTPYTDIYAR ALKVNAGLYA NELNTVLGQN DIQVKDQVAP QIT-ATTAAT TTPPPNFALD VGQLGGMYAG KIFLVGTEQG LGVRNAGTIN STQSTLTLNA NGDLINNGNL
 nos-D4  TIEGKGLNGS LTPYTDIYAR ALKVNAGLYA NELNTVLGQN DIQVKDQVAP QIT-ATTGPT TTPPPNFALD VGQLGGMYAG KIFLVGTEQG LGVRNAGTIN STQSTLTLNA NGDLVNNGNL
 bau-D7  TIEGKGLNGS LTPYTDIYTR ALKVNAGLYA NELNTVLGQN DIQVKDQVAP QIT-ATTAAT TTPQPNFALD VGQLGGMYAG KIFLVGTEQG LGVRNAGTIN STQSTLTLNA NGDLINNGNL
 bau-D5  TIEGKGLNGS LTPYTDIYAR ALKVNAGLYA NELNTVLGQN DIQVKDQVAP QIT-ATTAAT PTPQPNFALD VGQLGGMYAG KIFLVGTEQG LGVRNAGTIN STQSTLTLNA NGDLINNGNL
 nos-D8  TIEGKGLNGS LTPYTDIYAR ALKVNAGLYA NELNTVLGQN DIQVKDQVAP QIT-ATTGPT TTPPPNFALD VGQLGGMYAG KIFLVGTEQG LGVRNAGTIN STQSTLTLNA NGDLVNNGNL
 bau-D9  TIEGKGLNGS LTPYTDIYAR ALKVNAGLYA NELNTVLGQN DIQVKDQIVP QIK-AIKDST STPQPNFALD VGQLGGMYAG KIFLVGTEQG LGVRNAGTIN STQSTLTLNA NGDLINNGNL
 bau-D6  TIEGKGLNGS LTPYTDIYAR ALKVNAGLYA NELNTVLGQN DIQVKDQVAP QIT-ATTAAT TTPQPNFALD VGQLGGMYAG KIFLVGTEQG LGVRNAGTIN STQSTLTLNA NGDLINNGNL
nos-D12  TIEGKGLNGS LTPYTDIYAR ALKVNAGLYA NELNTVLGQN DIQVKDQVAP QIT-ATTGPT TTPPPNFALD VGQLGGMYAG KIFLVGTEQG LGVRNAGTIN STQSTLMLNA NGDLVNNGNL
bau-D10  TIEGKGLNGS LTPYTDIYAR ALKVNAGLYA NELGTVLGQN DIQVKDQVTP KIT-ATTGPT STPQPNFALD VGQLGGMYAG KIFLVGTEQG LGVRNAGSIN STQSTLTLNA NGDLVNNGNL
bau-D11  TIEGKGLNGS LTPYTDIYAR ALKVNAGLYA NELNTVLGQN DIQVKDQIAP QIK-AIKDST STPQPNFALD VGQLGGMYAG KIFLVGTEQG LGVRNAGAIN STQSTLTLNA NGDLVNNGNL
bau-D14  TIEGKGLNGS LTPYTDIYAR ALKVNAGLYA NELNTVLGQN DIQVKDQVAP QIT-AMTAAT TTPQPNFALD VGQLGGMYAG KIFLVGTEQG LGVRNAGTIN STQSTLTLNA NGDLINNGNL
bau-D12  TIEGKGLNGS LTPYTDIYAR ALKVNAGLYA NELNTVLGQN DIHVKDQIAP QIK-AIK--D STPQPNFALD VGQLGGMYAG KIFLVGTEQG LGVRNAGSIN STQSTLTLNA NGDLVNNGNL
bau-D20  TIEGKGLNGS LTPYTDIYAR ALKVNAGLYA NELNTVLGQN DIQVKDQVAP QIT-ATTAAT TTPQPNFALD VGQLGGMYAG KIFLVGTEQG LGVRNAGAIN STQSTLTLNA NGDLVNNGNL
bau-D15  TIEGKGLNGS LTPYTDIYAR ALKVNAGLYA NELNTVLGQN DIQVKDQVAP QIT-ATTAAT TTPQPNFALD VGQLGGMYAG KIFLVGTEQG LGVRNAGTIN STQSTLTLNA NGDLINNGNL
bau-D16  TIEGKGLNGS LTPYTDIYAR ALKVNAGLYA NELNTVLGQN DIQVKDQVAP QIT-ATTAAT TTPQPNFALD VGQLGGMYAG KIFLVGTEQG LGVRNAGTIN STQSTLTLNA NGDLINNGNL
bau-D21  TIEGKGLNGS LTPYTDIYAR ALKVNAGLYA NELNTVLGQN DIQVKDQVAP QIT-ATTAAT TTPQPNFALD VGQLGGMYAG KIFLVGTEQG LGVRNAGTIN STQSTLTLNA NGDLINNGNL
bau-D19  TIEGKGLNGS LTPYTDIYAR ALKVNAGLYA NELNTVLGQN DIQVKDQVAP QIT-ATTAAT TTPQPNFALD VGQLGGMYAG KIFLVGTEQG LGVRNAGTIN STQSTLTLNA NGDLINNGNL
bau-D13  TIEGKGLNGS LTPYTDIYAR ALKVNAGLYA NELNTVLGQN DIQVKDQVTP KIT-ATTGPT STPQPNFALD VGQLGGMYAG KIFLVGTEQG LGVRNAGSIN STQSTLTLNA KGDLVNSGNL
cal-D23  TIEGKGLNGS LTPYTDIYAR ALKVNAGLYA NELNTVLGQN DIQVKDQVTP KIT-ATTGST STPQPNFALD VGQLGGMYAG KIFLVGTEQG LGVRNAGSIN STQSGFTLNA NGDLVNSGNL
pit-D20  TIEGKGLNGS LTPYTDIYAR ALKVNAGLYA NELGTVLGQN DIQVKDQVTP QIK-ATTGST STPQPNFALD VGQLGGMYGG KIFLVGTEQG LGVRNAGSIN STQSSLTLNT NGDLVNSGNL
 pit-D6  TIEGKGLNGS LTPYTDIYAR ALKVNAGLYA NELGTVLGQN DIQVKDQVTP QIK-ATTGST STPQPNFALD VGQLGGMYGG KIFLVGTEQG LGVRNAGSIN STQSSLTLNT NGDLVNSGNL
bau-D17  TIEGKGLNGS LTPYTDIYTR ALKVNAGLYA NELNTVLGQN DIQVKDQITP KIT-ATTGPT STPQPNFALD VGQLGGMYAG KIFLVGTEQG LGVRNAGSIN STQSTLTLNA KGDLVNSGNL
nos-D18  TIEGKGLNGS LTPYTDIYAR ALKVNAGLYA NELNMVLGQN DIQVKDQVAL QIT-ATTGPT TTPPPNFALD VGQLGGMYAG KIFLVGTEQG LGVRNAGTIN STQGTLTLNA NGDLVNNGNL
nos-D22  TIEGKGLNGS LTPYTDIYAR ALKVNAGLYA NELNTVLGQN DIQVKDQVAP QIT-ATTGPI TTPPPNFALD VGQLGGMYAG KIFLVGTEQG LGVRNAGTIN STQGTLTLNA NGDLVNNGNL
 bau-D2  TIEGKGLNGS LTPYTDIYAR ALKVNAGLYA NELKTVLGQN DISIQDPSAP QIAKATTGTN TTPQPDFALD VGQLGGMYAG KIYLVGTEQG LGVRNAGSMN ATTGQLSLNA NGDLINTGNM
bay-D15  TIQGKGLNGS LTPFTDVYAR ALQVNAGLYA KELNVVLGQN DINVNDPTTS NVSPIQSNTS ANNKPQFALD VGQLGGMYAG KIFLVGTEQG VGVRNAGSMN STQSTLTLNA NGDLTNTGNL

         361                                                                                                                         480
 bau-D1  IANQDQVQLK AQNTKNTGNV SSATSQISIE SQSLDNSGLI SSADELRLNQ QNSLSNS-GT LNAARLVIDA GSLKNS-GSI EQTGLQGLDL KSGSMTNLGG KIGIAKNTTG GGSGGSTGGS
 nos-D2  IANKDQAQLK AQNIQNTGNV SSATSQISIE SQSLDNSGLI SSADELRLNQ QNSLSNS-GT LNAARLVIDA GSLKNS-GSI EQTGLQGLDL KSGSMTNLGG KIGIAKNTTG GGSGGSTGGS
 bau-D3  IANKDQVQLK AQNIQNTGNV SSATSQISIE SQSLDNSGLI SSADELQLNQ QNSLSNS-GT LNATRVVIDA GSLKNS-GSI EQTGLQGLDL KSGSMTNLGG KIGIAKNTTG GGTGGSTGGS
 nos-D4  IANKDQVQLK AQNTKNTGNV SSATSQISIE SQNLDNSGLI SSADELRLNQ QNSLSNS-GT LNAARLVIDV GSLKNS-GSI EQTGLQGLDL KSGSMTNLGG KIGIAKNTTG GGTGGSTGGS
 bau-D7  IANKDQVQLK AQNIQNTGNV SSATSQISIE SQSLDNSGLI SSADELQLNQ QNSLSNS-GT LNAARVVIDA GSLKNS-GSI EQTGLQGLDL KSGSMTNLGG KIGIAKNTTG GGTGGSTGGS
 bau-D5  IANKDQVQLK AQNIQNTGNV SSATSQISIE SQRLDNSGLI SSADELQLNQ QNSLSNS-GT LNAARVVIDA GSLKNS-GSI EQTGLQGLDL KSGSMTNLGG KIGIAKNTTG GGTGGSTGGS
 nos-D8  IANKDQVQLK AQNIQNTGNV SSATSQISIE SQSLDNSGLI SSADELRLNQ QNSLSNS-GT LNAARLVIDA GSLKNS-GSI EQTGLQGLDL KSGSMTNLGG KIGIAKNTTG GGSGGSTGGS
 bau-D9  IANKDQVQLK AQNIQNTGNV SSATSQISIE SQSLDNSGLI SSADELQLNQ QNSLSNS-GT LNAARVVIDA GSLKNS-GSI EQTGLQGLDL KSGSMTNLGG KIGIAKNTTG GGTGGSTGGS
 bau-D6  IANKDQVQLK AQNIQNTGNV SSATSQISIE SQSLDNSGLI SSADELQLNQ QNSLSNS-GT LNAARVVIDA GSLKNS-GSI EQTGLQGLDL KSGSMTNLGG KIGIAKNTTG GGTGGSTGGS
nos-D12  IANKDQVQLK AQNTKNTGNV SSATSQISIE SQSLDNSGLI SSADELRLNQ QNSLSNS-GT LNAARLVIDA GSLKNS-GSI EQTGLQGLDL KSGSMTNLGG KIGIAKNTTG GGSGGSTGGS
bau-D10  IANKDQVQLK AQNIQNTGNI SSATSQISVE SQNLNNSGLI SSADELHLQN QNTITNS-GT LNAARIAINS KNLKNR-GSI EQTGIQGLDL KSGSMTNLGG KIGIAKSNTG GGTGGSTGGS
bau-D11  IANKDQVQLK AQNIQNTGNI SSATSQILVE SQNLNNSGLI SSADELHLQN QNTITNS-GT LNAARIAINS KNLKNS-GSI EQTGIQGLDL KSGSMTNLGG KIGIAKSNTG GGTGGSTGGS
bau-D14  IANKDQVQLK AQNIQNTGNI SSATSQILVE SQNLNNSGLI SSADELHLQN QNTITNS-GT LNAARIAINS KNLKNS-GSI EQTGIQGLDL KSGSMTNLGG KIGIAKSNTG GGTGGSTGGS
bau-D12  IANKDQVQLK AQNIQNTGNI SSATSQILVE SQNLNNSGLI SSVDELHLQN QNTITNS-GT LNAARIAINS KNLKNS-GSI EQTGIQGLDL KSGSMTNLGG KIGIAKSNTG GGTGGSTGGS
bau-D20  IANKDQVQLK AQNIQNTGNI SSATSQILVE SQNLNNSGLI SSADELHLQN QNTITNS-GT LNAARIAINS KNLKNR-GSI EQTGIQGLDL KSGSMTNLGG KIGIAKSNTG GGTGGSTGGS
bau-D15  IANKDQVQLK AQNIQNTGNI SSATSQILVE SQNLNNSGLI SSADELHLQN QSTITNS-GT LNAARIAINS KNLKNS-GSI EQTGIQGLDL KSGSMTNLGG KIGIAKSNTG GGTGGSTGGS
bau-D16  IANKDQVQLK AQNIQNTGNI SSATSQILVE SQNLNNSGLI SSADELHLQN QSTITNS-GT LNAARIAINS KNLKNS-GSI EQTGIQGLDL KSGSMTNLGG KIGIAKSNTG GGTGGSTGGS
bau-D21  IANKDQVQLK AQNIQNTGNI SSATSQILVE SQNLNNSGLL SSADELHLQN QNTITNS-GT LNAARIAINS KNLKNS-GSI EQTGIQGLDL KSGSMTNLGG KIGIAKSNTG GGTGGSTGGS
bau-D19  IANKDQVQLK AQNIQNTGNI SSATSQILVE SQNLNNSGLI SSADELHLQN QSTITNS-GT LNAARIAINS KNLKNS-GSI EQTGIQGLDL KSGSMTNLGG KIGIAKSNTG GGTGGSTGGS
```

```
bau-D13   IANKDQVQLK AQNIQNTGNI SSATSQISVE SQNLNNSGLI SSADELHLQN QNTITNS-GT LNAARIAINS TKLKNS-GSI EQTGLQGLDL KSGSMTNLGG KIGIAKSNTG GGTGGSTGGS
cal-D23   IANKDQVQLK AQNIQNTGNI SSATSQVSIA SQNLNNSGLI SSADELHLQN QNTITNS-GT LNAARIVINS TKLKNS-GSI EQTGLQGLDL KSGSMTNLGG KIGIAKSNTG GGTGGSTGES
pit-D20   IANKDQVQLK AQNIQNTGNI SSATSQISVE SQNLNNSGLI SSADELHLQN QSIVTNS-GT LNAARIAINS KNLKNS-GSI EQTGIQGLDL KSGSMTNLGG KIGIAKSNTG GGTGGSTGGS
 pit-D6   IANKDQVQLK AQNIQNTGNI SSATSQISVE SQNLNNSGLI SSADELHLQN QNIVTNS-GT LNAARIAINS KNLKNS-GSI EQTGIQGLDL KSGSMTNLGG KIGIAKSNTG GGTGGSTGGS
bau-D17   IANKDQVQLK AQNIQNTGNI SSATSQISVE SQNLNNSGLI SSADELHLQN QNTITNS-GT LNAARIAINS TKLKNS-GSI EQTGLQGLDL KSGSMTNLGG KIGIAKSNTG G-TGGSTGGS
nos-D18   IANKDQVQLK AQNIQNTGNV SSATSQISIE SQSLDNSGLI SSADELRLNQ QNSLSNS-GT LNAARLVIDA GSLKNS-GSI EQTGLQGLDL KSGSMTNLGG KIGVAKSNTS GGTAGNSSGN
nos-D22   IANKDQVQLK AQNIQNTGNV SSATSQISIE SQSLDNSGLI SSADELRLNQ QNSLSNS-GT LNAARLVIDA DSLKNS-GSI EQTGLQGLDL KSGSMTNLGG KIGVAKSNTS GGTAGNSSGN
 bau-D2   IANKDQIAIQ AQNIKNSGNI SSTQHNIQIK ADNIQNSGLI ATNDEIQLNT QDKIDNNDGV INAGRIDFTA QTLSNDKGKI EQTGQQQLNI SAKTLDNTQG LIGQATKESS GTSGGTTTPS
bay-D15   IANQGQLQLN AENIKNTGNI SSATSQVSLH SQNLDNSGLI SSADELNIQQ QGHVGNR-GT LNAARIVVDA GSLANS-GSI EQTGAQALDL KAGSMSNIGG KIGIAKTSST GGNGSESGGG

          481                                                                                                                      600
 bau-D1   ------VPTV PTDPSKDGGS LEVATPID-- TTPKTYDQGY IHVKEQLNND QGAIIANGGV DLDSQNGLDN QGGQLNLGAI HIKGNSFNNN QGELTVKSAD IQTSSFSNQQ GLLQSLTSLD
 nos-D2   ------VPTV PTDPSRDGGS LEVATPID-- TTPKTYDQGY IHVKEQLNND QGAIIANGGV DLDSQNGLDN QGGQLNLGAI HIKGNSFNNN QGELTVKSAD IQTSSFSNQQ GLLQSLTSLD
 bau-D3   ------VPTV PTDPSKDGGS LEVATPID-- TTPKTYDTGF IHVSDVLNND QGAIIANGGV DLDSQNGLDN QGGQLNLGAI HIKGNSFNNN QGELTVKSAD IQTSSFSNQQ GLLQSLTSLD
 nos-D4   ------VPTV PTDPSKDGGS LEVATPID-- TTPKTYDQGY IHVKEQLNND QGAIIANGGV DLDSQNGLDN QGGQLNLGAI HIKGNSFNNN QGELTVKSAD IQTSSFSNQQ GLLQSLTSLD
 bau-D7   ------VPTV PTDPSKDGGS LEVATPID-- TTPKTYDQGY IHVQEQLNND QGAIIANGGV DLDSQNGLDN QGGQLNLGAI HIKGNSFNNN QGELTVKSAD IQTSSFSNQQ GLLQSLTSLD
 bau-D5   ------VPTV PTDPSKDGGS LEVATPID-- TTPKTYDQGY IHVQEQLNND QGAIIANGGV DLDSQNGLDN QGGQLNLGAI HIKGNSFNNN QGELTVKSAD IQTSSFSNQQ GLLQSLTSLD
 nos-D8   ------VPTV PTDPSKDGGS LEVATPID-- TTPKTYDQGY IHVKEQLNND QGAIIANGGV DLDSQNGLDN QGGQLNLGAI HIKGNSFNNN QGELTVKSAD IQTSSFSNQQ GLLQSLTSLD
 bau-D9   ------VPTV PTDPSKDGGS LEVATPID-- TTPKTYDQGY IHVQEQLNND QGAIIANGGV DLDSQNGLDN QGGQLNLGAI HIKGNSFNNN QGELTVKSAD IQTSSFSNQQ GLLQSLTSLD
 bau-D6   ------VPTV PTDPSKDGGS LEVATPID-- TTPKTYDQGY IHVQEQLNND QGAIIANGGV DLDSQNGLDN QGGQLNLGAI HIKGNSFNNN QGELTVKSAD IQTSSFSNQQ GLLQSLTSLD
nos-D12   ------VPTV PTDPSKDGGS LEVATPID-- TTPKTYDQGY IHVKEQLNND QGAIIANGGV DLDSQNGLDN QGGQLNLGAI HIKGNSFNNN QGELTVKSAD IQTSSFSNQQ GLLQSLTSLD
bau-D10   ------VPTV PTDPSKDGGS LGVVTPVD-- TTPKTYYTGF IYVSDVLNND QGAIVANGGV DLDSQNGLDN QSGQLNLGAI NIKGNSFNNN QGQLTVKSAN IQTSSFTNQQ GQLTSNSTLN
bau-D11   ------VPTV PTDPSKDGGS LGIVTPVD-- TTPKTYDTGF IHVSDVLNND QGAIVANGGV DLDSQNGLDN QGGQLNLGAI NIKGNSFNND QGQLTVKSAN IQTSSFTNQQ GQLASNSTLN
bau-D14   ------VPTV PTDPSKDGGS LGVVTPVD-- TTPKTYDTGF IHVSDVLNND QGAIVANGGV DLDSQNGLDN QGGQLNLGAI NIKGNSFNND QGQLTVKSAN IQTSSFTNQQ GQLASNSTLN
bau-D12   ------VPTV PTDPSKDGGS LGVVTPVD-- TTPKTYDTGF IHVSDVLNND QGAIVANGGV DLDSQNGLDN QGGQLNLGTI NIKGNSFNND QGQLTVKSAN IQTSSFTNQQ GQLASNSTLN
bau-D20   ------VPTV PTDPSKDGGS LGVVTPVD-- TTPKTYDTGF IHVSDVLNND QGAIVANGGV DLDSQNGLDN QGGQLNLGAI NIKGNSFNNN QGQLTVKSAN IQTSSFTNQQ GQLASNSTLN
bau-D15   ------VPTV PTDPSKDGGS LGVVTPVD-- TTPKTYDTGF IHVSDVLNND QGAIVANGGV DLDSQNGLDN QGGQLNLGAI NIKGNSFNNN QGQLTVKSAN IQTSSFTNQQ GQLASNSTLN
bau-D16   ------VPTV PTDPSKDGGS LGVVTPVD-- TTPKTYDTGF IHVSDVLNND QGAIVANGGV DLDSQNGLDN QGGQLNLGTI NIKGNSFNND QGQLTVKSAN IQTSSFTNQQ GQLASNSTLN
bau-D21   ------VPTV PTDPSKDGGS LGVVTPVD-- TTPKTYDTGF IHVSDVLNND QGAIVANGGV DLDSQNGLDN QGGQLNLGAI NIKGNSFNND QGQLTVKSAN IQTSSFTNQQ GQLASNSTLN
bau-D19   ------VPTV PTDPSKDGGS LGVVTPVD-- TTPKTYDTGF IHVSDVLNND QGAIVANGGV DLDSQNGLDN QGGQLNLGTI NIKGNSFNND QGQLTVKSAN IQTSSFTNQQ GQLASNSTLN
bau-D13   ------VPTV PTDPSKDGGS LGVVTPVD-- TTPKTYDTGF IHVSDVLNND QGAIVANGGV DLDSQNGLDN QGGQLNLGAI NIKGNSFNND QGQLTVKSAN IQTSTFTNQQ GQLASNSTLN
cal-D23   ------IPTV PTDPSKDGGS LGVVTPVD-- TTPKIYDTGF IHVSDVLNND QGAIVANGGV DLDSQNGLDN QGGQLNLGAI NIKGNSFNNN QGQLTVKSAN IQTSSFTNQQ GQLASNSTLN
pit-D20   ------VPTV PTDPSKDGGS LGVVTPVD-- TTPKTYDTGF IHVSDVLNND QGAIVANGGV DLDSQNGLDN QGGQLNLGAI NIKGNSFNNN QGQLTVKSAS IQTSSFTNQQ GQLASNSTLN
 pit-D6   ------VPTV PTDPSKDGGS LGVVTPVD-- TTPKTYDTGF IHVSDVLNND QGAIVANGGV DLDSQNGLDN QGGQLNLGAI NIKGNSFNNN QGQLTVKSAN IQTSSFTNQQ GQLASNSTLN
bau-D17   ------VPTV PTDPSKDGGS LGVVTPVD-- TTPKTYDTGF IHVSDVLNND QGAIVANGGV DLDSQNGLDN QGGQLNLGAI NIKGNSFNND QGQLTVKSAN IQTSTFTDQQ GQLVSNSTLN
nos-D18   TGSTVIVPRA PVDSAKDGGS LGVVSPID-- TTPKTYDIGF IHVSGVLGND QGTIIAHGGV DVESQNGLDN QGGQLNLGTV TIKGSNFNND LGQLNVKQAD IQTLDFTNQQ GQFASSSTLK
nos-D22   TGSTVIVPTV PVDPAQDGGS LGVVPPID-- TTPKTYDTGF IHVSGVLGND QGTIIANGGV DLESQNGLDN QGGQLNLGIV TIKGSNFNND LGQLNVKQAD IQTLGFTNQQ GQLASSDTLK
 bau-D2   V-----TDPE QQSSAQDSST VEVAPVD--- LTPKTFQTGN IQIAQGIDNV AGQIVNNADI NLKVQDSIKN NAGEIQLPEL QFSGQNFENQ QGKFTAKVVN INAQNIDNQK GVMSASQSFD
bay-D15   S-----QPVE PNNAAQDGGS LEVVNTDSTP TTPKTYETGY IHVRDQLNND QGAIVAQGGI DLESNNGLDN QGGQLNLGRV KIQGERFNND QGQLTVKQAE IQTNSLSNQS GQLISNQKLN

          601                                                                                                                      720
 bau-D1   VNSQSIDNQG GKINALNNIS MISSGNILNQ AGQIASSSEL YLQGLGLNNS GGDLEAE-QL LKLNLSGHLN NQKGKIVTNN NLDSSLFGLD NDQGEISAKN ITIQNKDQAL NNGSGTIYAD
 nos-D2   VNSQSIDNQG GKINALNNIS MISSGNILNQ AGQIASSSEL YLQGLGLNNS GGDLEAE-QL LKLNLSGHLN NQKGKIVTNN NLDSSLFGLD NDQGEISAKN ITIQNKDQAL NNGSGTIYAD
 bau-D3   VNSQSIDNQG GKINALNNIS MISSGNILNQ AGQIASSSEL YLQGLGLNNS GGDLEAE-QL LKLNLSGHLN NQKGKIVTNN NLDSSLFGLD NDQGEISAKN ITIQNKDQAL SNGSGTIYAD
 nos-D4   VNSQSIDNQG GKINALNNIS MISSGNILNQ AGQIASSSEL YLQGLGLNNS GGDLEAE-QL LKLNLSGHLN NQKGKIVTNN NLDSSLFGLD NDQGEISAKN ITIQNKDQAL NNGSGTIYAD
 bau-D7   VNSQSIDNQG GKINALNNIS MISSGNILNQ AGQIASSSEL YLQGLGLNNS GGDLEAE-QL LKLNLSGHLN NQKGKIVTNN NLDSSLFGLD NDQGEISAKN ITIQNKDQAL SNGSGTIYAD
 bau-D5   VNSQSIDNQG GKINALNNIS MISSGNILNQ AGQIASSSEL YLQGLGLNNS GGDLEAE-QL LKLNLSGHLN NQKGKIVTNN NLDSSLFGLD NDQGEISAKN ITIQNKDQAL SNGSGTIYAD
 nos-D8   VNSQSIDNQG GKINALNNIS MISSGNILNQ AGQIASSSEL YLQGLGLNNS GGDLEAE-QL LKLNLSGHLN NQKGKIVTNN NLDSSLFGLD NDQGEISAKN ITIQNKDQAL NNGSGTIYAD
 bau-D9   VNSQSIDNQG GKINALNNIS MISSGNILNQ AGQIASSSEL YLQGLGLNNS GGDLEAE-QL LKLNLSGHLN NQKGKIVTNN NLDSSLFGLD NDQGEISAKN ITIQNNDQAL SNGSGTIYAD
 bau-D6   VNSQSIDNQG GKINALNNIS MISSGNILNQ AGQIASSSEL YLQGLGLNNS GGDLEAE-QL LKLNLSGHLN NQKGKIVTNN NLDSSLFGLD NDQGEISAKN ITIQNNDQAL SNGSGTIYAD
nos-D12   VNSQSIDNQG GKINALNNIS MISSGNILNQ AGQIASSSEL YLQGLGLNNS GGDLEAE-QL LKLNLSGHLN NQKGKIVTNN NLDSSLFGLD NDQGEISAKN ITIQNKDQAV NNGSGTIYAD
bau-D10   IQSQSANNKS GKIQSIGQLD LAVTGELNNV DGQIASGATV NVTASDLKNQ SGVVYSENQA VNIKVNKAID NTEGLIQAKT NLNLDSQSLN NTSGQIVADQ I--NQKHVTV NNNQGSIAAQ
bau-D11   IQTQSANNKD GKIQSIGQLD LAVTGELNNV DGQIASGANI NVTASDLKNQ SGVVYSENQA VNIKANKAID NTEGLIQAKT NLNLDSQSLN NTAGQIVADQ I--NQKHVTV NNNQGSIAAQ
bau-D14   IQTQSANNKD GKIQSIGQLD LAVTGELNNV DGQIASGANI NVTASDLKNQ SGVVYSENQA VNIKANKAID NTEGLIQAKT NLNLDSQSLN NTAGQIVADQ I--NQKHVTV NNNQGSIAAQ
bau-D12   IQTQSANNKD GKIQSIGQLD LAVTGELNNV DGQIASGANI NVTASDLKNQ SGVVYSENQA VNIKANKAID NTEGLIQAKT NLNLDSQSLN NTAGQIVADQ I--NQKHVTV NNNQGSIAAQ
bau-D20   IQTQSANNKS GKIQSIGQLD LAVTGELNNV DGQIASGATV NVTASDLKNQ SGVVYSENQA VDIKANKAID NTKGLIQAKT NLNLNSQSLN NTAGQIVADQ I--NQKHVTV NNNQGSIAAQ
bau-D15   IQTQSANNKG GKIQSIGQLD LAVTGELNNV DGQIASGATV NVNASDVKNQ SGVVYSENQA VNIKANKAID NTEGLIQAKT NLNLDSQSLN NTSGQIVADQ I--NQKHVTV NNNQGSIAAQ
bau-D16   IQTQSANNKD GKIQSIGQLD LAVTGELNNV DGQIASGANI NVTASDLKNQ SGVVYSENQA VNIKANKAID NTEGLIQAKT NLNLNSQSLN NTSGQIVADQ I--KQKHVTV NNNQGSIAAQ
bau-D21   IQTQSANNKD GKIQSIGQLD LAVTGELNNV DGQIASGANI NVTASDLKNQ SGVVYSENQA VNIKANKAID NTEGLIQAKT NLNLDSQSLN NTAGQIVADQ I--NQKHVTV NNNQGSIAAQ
bau-D19   IQTQSANNKD GKIQSIGQLD LAVTGELNNV DGQIASGANI NVTASDLKNQ SGVVYSENQA VNIKANKAID NTEGLIQAKT NLNLNSQSLN NTSGQIVADQ I--KQKHVTV NNNQGSIAAQ
bau-D13   IQTQSANNKG GKIQSIGQLD LAVSGELNNV DGQIASGATV NVNASDLKNQ SGVVYSENQA VNIKTNKAID NTEGLIQAKT NLNLNSQSLN NTSGQIVADQ I--KQKHVTV NNNQGSIAAQ
cal-D23   IQSQSANNKG GKIQSIGQLD LAATGELNNV DGQIASGATV NVTASDLKNQ SGVVYSENQA VDIKANKAID NTEGLIQAKT NLNLNSQSLN NTAGQIVANQ I--NQKHVTV NNNQGSIAAQ
pit-D20   IQTQSANNKS GKIQSIGQLD LAVSGELNNV DGQIASGATV NVNASDLKNQ SGVVYSENQA VDIKANKAID NTEGLIQAKT NLNLDSQSLN NTSGQIVADQ I--NQKHVTV NNNQGSIAAQ
 pit-D6   IQTQSANNKG GKIQSIGQLD LAVIGELNNV DGQIASGATV NVNASDLKNQ SGVVYSENQA VNIKANKAVD NTEGLIQAKT NLNLDSQSLN NTSGQIVADQ I--NQKHVTV NNNQGSIAAQ
bau-D17   IQTQSANNKG GKIQSIGQLD LAVSGELNNV DGQIASGATV NVNASDLKNQ SGVVYSENQA VNIKANKAID NTEGLIQAKT NLNLNSQSLN NTSGQIVADQ I--KQKHVTV NNNQGSIAAQ
nos-D18   IQSQNAYNQS GKIQSIGQLD LAIGSELNNN KGYIASSDNV QVSAGDLSNQ FGVIYSENQS VNIKANQAID NTEGFIQAKT DLNLNSQALN NTSGHIIGNQ I--QQQHGTV NNSQGAIIAK
nos-D22   IQSQNANNRS GKIQSIGQLD LAIGGELNNS KGYVASGDNV LVSAGDLNNQ FGVVYSENQS VNIKANQAID NTEGVIQAKT DLNLNSQTLN NTSGQIIGNQ I--QQQHGTV NNSQGAIIAK
 bau-D2   LTAQQLNNNQ GRLQSTKALN IT-ATEIDNR QGQILAADAL TLNSSNTNNT QGVIASVNAD AQLNIK-SLD NSKGEISAQN -VHLTGQSLK NQQGTIQAKT A--DLNINVD QINNGS----
bay-D15   IQTQTANNQK GKIQSVDQLD LNVANGLNNI EGQIASGQQL NIQADQVNNQ SGLIYSEQQG IELKANHAIN NTSGIIQAKT QLNLASTQLD NTSGHIQAEN I--QQQHTTV NNTQGSIVAG

          721                                                                                                                      840
 bau-D1   QSLKIQTGSL NNAVNGTLSS HENLQIDSQQ LTNQGYIRAD QQLKINNTSV MTQQGGVLSA YGNIDLTSQR LVSDEKSVIA VGINAQGEQD QNAQADLNIK TEQALEHHGK LLASHNIDLD
 nos-D2   QSLKIQTGSL NNAVNGTLSS HENLQIDSQQ LTNHGYIRAD QQLKINNTGV MTQQGGVLSA YGNIDLTSQR LVSDEKSVIA VGINAQGEQD QNAQADLNIK TEQALEHHGK LLASHNIDLD
 bau-D3   QSLKIQTGSL NNAVNGTLSS HENLQIDSQQ LVNQGYIRAD QQLKINNTGV MTQQGGVLSA YGNIDLVSQR LVSDEKSIIA VGINAQGEQD QNAQADLNIK TEQALEHHGK LLASRNIDLD
 nos-D4   QSLKIQTGSL NNAVNGTLSS HENLQIDSQQ LTNQGYIRAD QQLKINNTGV MTQQGGILSA YGNIDLTSQR LVSDEKSVIA VGINAQGEQD QNAQADLNIK TEQALEHHGK LLASHNIDLD
 bau-D7   QSLKIQTGSL NNAVNGTLSS HENLQIDSQQ LVNQGYIRAD QQLKINNTGV MTQQGGVLSA YGNIDLVSQR LVSDEKSVIA VGINAQGEQD QNAQADLNIK TEQALEHHGK LLASRNIDLD
 bau-D5   QSLKIQTGSL NNAVNGTLSS HENLQIDSQQ LVNQGYIRAD QQLKINNTGV MTQQGGVLSA YGNIDLVSQR LVSDEKSVIA VGINAQG*QD QNAQADLNIK TEQALEHHGK LLASRNIDLD
 nos-D8   KSLKIQTGSL NNAVNGTLSS HENLQIDSQQ LTNQGYIRAD QQLKINNTGV MTQQGGVLSA YGNIDLTSQR LVSDEKSVIA VGINAQGEQD QNAQADLNIK TEQALEHHGK LLASHNIDLD
 bau-D9   QSLKIQTGSL NNAVNGTLSS HENLQIDSQQ LVNQGYIRAD QQLKINNTGV MTQQGGVLSA YGNIDLVSQR LVSDEKSVIA VGINAQGEQD QNAQADLNIK TEQALEHHGK LLASRNIDLD
 bau-D6   QSLKIQTGSL NNAVNGTLSS HENLQIDSQQ LVNQGYIRAD QQLKINNTGV MTQQGGVLSA YGNIDLVSQR LVSDEKSVIA VGINAQGEQD QNAQADLNIK TEQALEHHGK LLASRNIDLD
nos-D12   QSLKIQTGSL NNAVNGTLSS HENLQIDSQQ LTNQGYIRAD QQLKINNTGV MTQQGGVLSA YGNIDLTSQR LVSDEKSVIA VGINAQGEQD QNAQADLNIK TEQALEHHGK LLASHNIDLD
bau-D10   QNLNSTAQQF DNT-QGQIHA -KSIKLQHDQ LKNTGSVYAD QDLTV--TGQ NIQNAGTLGA GKNVQITSST LEHSVGGLIA AGLDREGKLG -DA-GDVSIR SDQV-SLHGQ TLAGGNLKVQ
bau-D11   QNLNSTAQQF DNT-QGQIQA -QSIKLQHDQ LKNTGNVYAD QELTI--SGK TIQNTGSLAA GENVQITGSV LEHAQDGLIA AGLNREGKLD -DT-GDLTIS VDQA-GLHGQ TFAGHNIQVD
bau-D14   QNLNSTAQQF DNT-QGQIQA -QSIKLQHDQ LKNTGNVYAD QELTI--SGK TIQNTGSLAA GENVQITGSV LEHAQDGLIA AGLNREGKLD -DT-GDLTIS VDQA-GLHGQ TFAGHNIQVD
bau-D12   QNLNSTAQQF DNT-QGQIQA -QSIKLQHDQ LKNTGNVYAD QELTI--SGK TIQNTGSLAA GENVQITGSV LEHAQDGLIA AGLNREGKLD -DT-GDLTIS VDQA-GLHGQ TFVGHNIQVD
bau-D20   QNLNSTVQQF DNT-QGQIQA -KSIKLQHDQ LKNTGSVYAD QDLTI--TGQ NTQNAGTLAA GKNVQITSSI LEHSVGGLIA AGLDREGKLV -NA-GDVSIS SDQV-GLHGQ TLAGGNLKVQ
bau-D15   QNLNSIAQQF DNT-QGQIQA -KSIKLQHDQ LKNTGSVYAD QDLTI--TGQ NTQNAGTLAA GKNVQITSST LEHSVGGLIA AGLDREGKLA -NA-GDVSIS SDQV-GLHGQ TLAGGNLKVQ
bau-D16   QNLNSTAQQF DNT-QGQIHA -KSIKLQHDQ LKNTGSVYAD QDLTV--TGQ NIQNAGTLGA GKNVQISSST LEHSVGGLIA AGLDREGKLG -DA-GDVTIR SDQV-GLHGQ TLAGGNLKVQ
bau-D21   QNLNSTAQQF DNT-QGQIQA -QSIKLQHDQ LKNTGNVYAD QELTI--SGK TIQNTGSLAA GENVQITGSV LEHAQDGLIA AGLNREGKLD -DT-GDLTIS VDQA-GLHGQ TFAGHNIQVD
bau-D19   QNLNSTAQQF DNT-QGQIHA -KSIKLQHDQ LKNTGSVYAD QDLTV--TGQ NIQNAGTLGA GKNVQISSST LEHSVGGLIA AGLDREGKLG -DA-GDVTIR SDQV-GLHGQ TLAGGNLKVQ
bau-D13   QNLNSTAQQF DNT-QGQIHA -KSIKLQHDQ LKNTGSVYAD QDLTV--TGQ NIQNAGTLGA GKNVQITSST LEHSVGGLIA AGLDREGKLG -DA-GDVTIR SDQV-GLHGQ TLAGGNLKVQ
cal-D23   QNLNSTAGQF DNT-QGQIQA -KSIKLQHDQ LKNTGSIYAD QDLTI--TGQ NTQNAGTLAA GKNVQIISST LEHNVGGLIA AGLDREGKLA -NV-GDMSIR ADQV-GLHGQ TLAGGNLKVQ
pit-D20   QNLNSIAQQF DNT-QGQIQA -KSIKLQHDQ LKNTGSVYAD QDLTI--TGQ NTQNAGTLAA GKNVQITSST LEHSVGGLIA AGLDREGKLA -NA-GDVSIS SDQV-GLHGQ TLAGGNLKVQ
 pit-D6   QNLNSIAQQF DNT-QGQIQA -KSIKLQHDQ LKNTGSVYAE QDLTI--TGQ NTQNAGTLAA GKNVQITSST LEHSVGGLIA AGLDREGKLA -NA-GDVSIS SDQV-GLHGQ TLAGGNLKVQ
bau-D17   QNLNSTAQQF DNT-QGQIHA -KSIKLQHDQ LKNTGSVYAD QDLTV--TGQ NIQNAGTLGA GKNVQITSST LEHSVGGLIA AGLDREGKLG -DA-GDVTIR SDQV-GLHGQ TLAGGNLKVQ
nos-D18   QNLNSTAQQF DND-QGQLQA -TTVQLQHDQ LKNTGGIYAD KDLTV--TGK DIQNSGSFAA GENVKITSSA LEHAQNGLIA AGLDREGKLA -DT-GMLTIN VDRA-DLHGQ TFAGGNIQVD
nos-D22   QNLNSTAQQF DND-QGQLQA -TTVQLQHDQ LKNTGGIYAD KDLTV--TGK DIQNSGSLAA GENVKITSLA LEHAQNGLIA AGLDREGKLA -DT-GMLTIN VDRA-GLHGQ TFAGQHIQIN
 bau-D2   -----TKDLA GN----LIAA -QNLKLNAQQ LQSTGQIYAG DTADL--TVE QLKQHGQLAA LNAVRVQSKD IESDQNAIWV AGLDQEGKLS -NTTSELNID AQNA-QIAGK ILSGDQINIK
bay-D15   QSLISTVDQF DNH-QGLIDA -KNIQLTHQV LNNSGSIYAD QNIKI--TGQ HILNTGSIAA GQNIQLNTAK LQHEQAALIA AGLDRQGKLT -ST-GDLDIT AEQT-TLHGQ SFAGQQLKVA

          841                                                                                                                      960
 bau-D1   G-ANVDLSQG TAAAQNINIT ARDGDINNQS GMLQADTIQL NAVQNQQSLI NQGGQILAKK LNLNIGKDIN NQQGLIQHTA SDDLNLTIQG VINNQQGRIL TNANQLNIQA QGLNSDSGVI
 nos-D2   G-ANVDLSQG TAAAQNINIT ARDGDINNQS GMLQADTIQL NAVQNQQSLI NQSGQILAKK LNLNIGKDIN NQQGLIQHTA SDDLNLTVQG VINNQQGRIL TNANQLNIQA QGLNSDSGVI
 bau-D3   G-ANVDLSQG TAAAQNINIT ARDGDINNQS GVLQADSIQL NAVQNQQSLI NQSGQILAKK LNLNIGKDIN NQQGLIQHTA TDDLNLTVQG IINNQQGRIL TNANQLNIKA QGLNSDSGVI
 nos-D4   G-ANVDLSQG TAAAQNINIT ARDGDINNQS GMLQADTIQL NAIQSQQSLI NQSGQILAKK LNLNIGKDIN NQQGLIQHTA SDDLNLTVQG VINNQQGRIL TNANQLNIQA QGLNSDSGVI
 bau-D7   G-ANVDLSQG TAAAQNINIT ARDGDINNQS GVLQADSIQL NAVRNQQSLI NQSGQILAKK LNLNIGKDIN NQQGLIQHTA TDDLNLTVQG IINNQQGRIL TNANQLNIKA QGLNSDSGVI
 bau-D5   G-ANVDLSQG TAAAQNINIT ARDGDINNQS GVLQADSIQL NAVQNQQSLI NQSGQILAKK LNLNIGKDIN NQQGLIQHTA TDDLNLTVQG IINNQQGRIL TNANQLNIKA QGLNSDSGVI
 nos-D8   G-ANVDLTQG TAAAQNINIT ARDGDINNQS GMLQADTIQL NAIQSQQSLI NQNGQILAKK LNLNIGKDIN NQQGLIQHTA SDDLNLTVQG VINNQQGRIL TNANQLNIQA QGLNSDSGVI
 bau-D9   G-ANVDLSQG TAAAQNINIT ARDGDINNQS GVLQADSIQL NAVQNQQSLI NQSGQILAKK LNLNIGKDIN NQQGLIQHTA TDDLNLTVQG IINNQQGRIL TNANQLNIKA QGLNSDSGVI
 bau-D6   G-ANVDLSQG TAAAQNINIT ARDGDINNQS GVLQADSIQL NAVQNQQSLI NQSGQILAKK LNLNIGKDIN NQQGLIQHTA TDDLNLTVQG IINNQQGRIL TNAIQLNIKA QGLNSDSGVI
nos-D12   G-ANVDLSQG TAAAQNINIT ARDGDINNQS GMLQADTIQL NAIQSQQSLI NQGGQILAKK LNLNIGKDIN NQQGLIQHTA SDDLNLTIQG VINNQQGRIL TNANQLNIQA QGLNSDSGVI
bau-D10   AQSNIDAAKG QLQAQNIELD SVTGNISTQA GSVVAQN-QL K-LTSQNLIN NQQGLLSSQD LILT-AKQLD NSQGKIQHTG NNEFTLDFVN GLNNKAGEIS SNASSINLNT SALNNETGKI
bau-D11   ATGKIDAAQG QLQSMTIALN AGT-DISTQT ATVVAQN-QL K-LTSQNLIN NQQGLLSSQD LILT-AKQLD NIQGKIQHTG NNEFALNFVN GLNNKAGEIS SNASAINLNT SALNNEAGKI
bau-D14   ATGKIDAAQG QLQSMTIALN AGT-DISTQT ATVVAQN-QL K-LTSQNLIN NQQGLLSSQD LILT-AKQLD NIQGKIQHTG NNEFALNFVN GLNNKAGEIS SNASAINLNT SALNNEAGKI
bau-D12   ATGKIDAAQG QLQSMTIALN AGT-DISTQT ATVVAQN-QL K-LTSQNLIN NQQGLLSSQD LILT-AKQLD NIQGKIQHTG NNEFALNFVN GLNNKAGEIS SNASAINLNT SALNNEAGKI
```

```
bau-D20  AQSNIDAAKG QLQAQNIELG SVTGNISTQA GSVVAKD-QL K-LVSQNLIN NQQGQLSAQN IVLN-AKQLD NSQGKIQHTG TNDLNIQFAN GLNNKAGEIS SNASSINLNT SALNNEAGKI
bau-D15  AQSNIDAAKG QLQAQNIELG SVTGNISTQA GSVVAKD-QL N-LVSQNLIN NQQGQLSAQN IVLN-AKQLD NSQGKIQHTG INEFALNFVN GLNNKAGEIS SNASSINLNT SALNNEAGKI
bau-D16  AQSNIDAAKG QLQAQNIELD SVTGNISTQA GSVVAQN-QL K-LTSQNLIN NQQGLLSSQD LILT-AKQLD NSQGKIQHTG NNEFTLDFVN GLNNKAGEIS SNASSINLNT SALNNETGKI
bau-D21  ATGKIDAAQG QLQSKKIALN AGT-DISTQT ATVVAQN-QL K-LTSRNLIN NQQGLLSSQD LILT-AKQLD NSQGKIQHTG NNEFALNFVN GLNNKAGEIS SNASVINLNT SALNNEAGKI
bau-D19  AQSNIDAAKG QLQAQNIELD SVTGNISTQA GSVVAQN-QL K-LTSQNLIN NQQGLLSSQD LILT-AKQLD NSQGKIQHTG NNEFTLDFVN GLNNKAGEIS SNASSINLNT SALNNETGKI
bau-D13  AQSNIDAAKG QLQAQNIELD SVTGNISTQA GSVVAQN-QL K-LTSQNLIN NQQGLLSSQD LILT-AKQLD NSQGKIQHTG NNEFTLDFVN GLNNKAGEIS SNASSINLNT SALNNETGKI
cal-D23  AQSNIDAAKG QLQAQNIELA SAAGNISTQA GNVVAQD-QL K-LVGQNLIN NQQGQLSAKN VLLT-AKQLD NSQGKIQHTG NNEFTLNFVN GLNNKAGEIS SNASSISLNT SILNNEDGKL
pit-D20  AQSNIDAAKG QLQAQNIELG SVTGNISTQA GSVVAKD-QL N-LVSQNLIN NQQGQLSAQN IVLN-AKQLD NSQGKIQHIG NNEFTLDFVN GLNNKSGEIS SNASSINLNT SALNNEAGKI
 pit-D6  AQSNIDAAKG QLQAQNIELG SVTGNISTQA GSVVAKD-QL N-LVSQNLIN NQQGQLSAQN IVLN-AKQLD NSQGKIQHIG NNEFTLDFVN GLNNKSGEIS SNASSINLNT SALNNEAGKI
bau-D17  AQSNIDAAKG QLQAQDIELD SVAGNISTQA GSVVAQN-QL K-LTSQNLIN NQQGLLSSQD LILT-AKQLD NSQGKIQHTG NNEFTLDFVN GLNNKAGEIS SNASSINLNT SALNNEAGKI
nos-D18  ATRTINAAQG QLQSKTIALN AGT-DISTQA ATVVAQD-QL K-LTSQNLIN NQQGQLNSQD IILS-AQQLD NSQGKIQHTG NNEFTLNFVN GLNNKSGEIS SNAVLMQLNT SILNNEAGQI
nos-D22  ATGTIDAAQG QLQSKTIALN AGT-DISTQA ATVVAQD-QL K-LASQNLIN NQQGQLNSQD IILS-AQQLD NSQGKIQHTG NNEFTLNFVN GLNNKLGEIS SNAALMQLNN SILNNEAGQI
 bau-D2  AAQTTDLSQS ESQAKNVQIE TTQFDTSS-A KIIADR--QL D-IRALQSIN NEKGQYSAEQ VNLN-TAQLN NNQGLIQHTG KNDFILNVAD RIANHAGKII SNAKTTEIKT NTLSSVAGEI
bay-D15  SKQQIDATNG QLQAKNIDLA SDAGDISTRA ATVIAE--QL S-LKSHDLID NQQGTLSAQQ LNLT-ANRLD NTQGHILHTG QDQLDLNFSQ SLNNQLGEIR SNAAELNIKT ALLNNTEGQV

         961                                                                                                                1080
 bau-D1  LHSGQAGLNM DIQNIHAQKA ELRTNGALKL TTDQALLDQS KVSATQANIQ ANQFTDRNGE MLFSAVNGQS SIRVNGDYQH QGSVLQSNHS LDIQTGSLSN QNGNILVTQN GSSSQPV---
 nos-D2  LHSGQAGLNM DIQNIHAQKA ELRTNGALKL TTDQALLDQS KVSATQANIQ ANQFTDRNGE MLFSAVNGQS SIRVNGDYQH QGSVLQSNHS LDIQTGSLSN QSGNILVTQN EATSQPV---
 bau-D3  LHSGQAGLNM DIQNIYAQKA ELRTNGALKL TTDQALLDQS KVSATQANIQ ANQLTDRNGE MLFSAANGQS SIRVNGDYQH QGSVLQSNHS LDIQTDSLSN QSGNILVTQN EATSQPV---
 nos-D4  LHSGQAGLNM DIQNIHAQKA ELRTNGALKL TTDQALLDQS KVSATQANIQ ANQFTDRNGE MLFSAVNGQS SIRVNGDYQH QGSVLQSNHS LDIQTGSLSN QSGNILVTQN EATSQPV---
 bau-D7  LHSGQAGLNM DIQNIHAQKA ELRTNGALKL TTDQALLDQS KVSATQANIQ ANQLTDRNGE MLFSAANGQS SIRVNGDYQH QGSVLQSNHS LDIQTGSLSN QSGNILVTQN EATSQPV---
 bau-D5  LHSGQAGLNM DIQNIHAQKA ELRTNGALKL TTDQALLDQS KVSATQANIQ ANQLTDRNGE MLFSAANGQS SIRVNGDYQH QGSVLQSNHS LDIQTGSLSN QSGNILVTQN EATSQPV---
 nos-D8  LHSGQAGLNM DIQNIHAQKA ELRTNGALKL TTDQALLDQS KVSATQANIQ ANQLTDRNGE MLFSAVNGQN SIRVNGDYQH QGSVLQSNHS LDIQTGSLSN QSGNILVTQN GSSSQPV---
 bau-D9  LHSGQAGLNM DIQNIHAQKA ELRTNGALKL TTDQALLDQS KVSATQANIQ ANQLTDRNGE MLFSAANGQS SIRVNGDYQH QGSVLQSNHS LDIQTDSLSN QSGNILVTQN EATSQPV---
 bau-D6  LHSGQAGLNM DIQNIHAQKA ELRTNGALKL TTDQALLDQS KVSATQANIQ ANQLTDRNGE MLFSAANGQS SIRVNGDYQH QGSVLQSNHS LDIQTDSLSN QSGNILVTQN EATSQPV---
nos-D12  LHSGQAGLNM DIQNIHAQKA ELRTNGALKL TTDQALLDQS KVSATQANIQ ANQFTDRNGE MLFSAVNGQS SIRVNGDYQH QGSVLQSNHS LDIQTGSLSN QNGNILVTQN GSSSQPV---
bau-D10  IHAGNQQLNI TADQLQGAQG QILSNGQLLL KGGQVVLDGA TTSANQINIS ADSLSHQKGQ MVQSGTLNPL TLAIKDQMNN QLGFIQSQSG LQLKTTTLHN QGGQLSSALN YDVQLDVAGL
bau-D11  IHAGNQQLNI TADQLQGAQG QILSNGQLLL KGGQVVLDGA TTSANQINIS ADSLSHQKGQ MVQSGTLNPL TLAIKDQMNN QLGFIQSQSG LQLKTTTFNN QGGQLSSALN YDVQIDVAGL
bau-D14  IHAGNQQLNI TADQLQGAQG QILSNGQLLL KGGQVVLDGA TTSANQINIS ADSLSHQKGQ MVQSGTLNPL TLAIKDQMNN QLGFIQSQSG LQLKTTTFNN QGGQLSSALN YDVQIDVAGL
bau-D12  IHAGNQQLNI TADQLQGAQG QILSNGQLLL KGGQVVLDGA TTSANQINIS ADSLSHQKGQ MVQSGTLNPL TLAIKDQMNN QLGFIQSQSG LQLKTTTFNN QGGQLSSALN YDVQIDVAGL
bau-D20  IHAGNQQLNI TADQLQGAQG QILSNGQLLL KGGQVVLDGA TTSANQINIS ADSLSHQKGQ MVQSGTLNPL TLAIKDQMNN QLGFIQSQSG LQLKTTTLNN QGGQLSSALN YDVQLDVAAL
bau-D15  IHAGSQQLNI TADQLQGAQG QILSNGQLLL KGGQVVLDGA TTSANQINLT ANSLSHQKGQ MVQRGTLKPL TLAVKEQINN QSGLIQSPTT LYITSGSLNN QGGQLSSAQD QNLNLQITGL
bau-D16  IHAGNQQLNI TADQLQGAQG QILSNGQLLL KGGQVVLDGA TTSANQINIS ADSLSHQKGQ MVQRGTLKPL TLAIKDQLNN QLGFIQSRSG LQLKTTTLNN QGGQLSSALN YDVQLDVAAL
bau-D21  IHVGNQQLNI TADQLQGAQG QILSNGQLLL KGGQVVLDGA TTSANQINIS ADSLSHQKGQ MVQNGTLNPL TLAIKDQMNN QLGFIQSQSG LQLKTTTFNN QGGQLSSALN YDVQIGVAGL
bau-D19  IHAGNQQLNI TADQLQGAQG QILSNGQLLL KGGQVVLDGA TTSANQINIS ADSLSHQKGQ MVQRGTLKPL TLAIKDQLNN QLGFIQSRSG LQLKTTTLNN QGGQLSSALN YDVQLDVAAL
bau-D13  IHAGNQQLNI TADQLQGAQG QILSNGQLLL KGGQVVLDGA TTSANQINIS ADSLSHQKGQ MVQSGTLNPL TLAIKDQMNN QLGFIQSQSG LQLKTTTLHN QGGQLSSALN YNVQLDVAGL
cal-D23  IHAGTQQLNI KANQLQGSQG QILTNGQLFV DAGQIVLDGA TTSANQINIS ANSLSHQKGQ MVQRGTLKPL TLAIKDQINN QLGFIQSQSG LQLKTTTLNN QSGQLSSALN YDVQLDVAGL
pit-D20  IHAGSQQLNI TADQLQGAQG QILSNGQLLL KGGQVVLDGA TTSVNQINIS ADSLSHQKGQ MVQRGTLKPL TLAIKDQLNN QLGFIQSQSG LQLKTTTLNN QGGQLSSALN YDVQLDVAAL
 pit-D6  IHVGSQQLNI TADQLQGAQG QILSNGQLLL KGGQVVLDGA TTSANQINIS ADSLSHQKGQ MVQRGTLKPL TLAIKDQLNN QLGFIQSQSG LQLKTTTLNN QGGQLSSALN YDVQLDVAAL
bau-D17  IHAGSQQLNI TADQLQGAQG QILSNGQLLL KGGQVVLDGA TTSANQINIS ADSLSHQKGQ MVQSGTLNPL TLAIKDQMNN QLGFIQSQSG LQLKTTTLHN QGGQLSSALN YNVQLDVAGL
nos-D18  IHSGNQQLNI NSNQLQGVQG KILSNGQLVL ATEQAILDGA ITSADQINIT AYNLSHQKGQ MIQRGTLKPL TLAITDQINN QLGFIQSPMA LYITAGSLNN QGGQLSSAAD HDLQLDVAGL
nos-D22  IHSGNQQLNI NSNQLQGAQG KILSNGQLVL ATEQAILDGA ITSADQINIT AYNLSHQKGQ MIQRGTLKPL TLAIRDQINN QLGFIQSPTA LYITAGSLNN QGGQLSSAAD HDLQLDVAGL
 bau-D2  LHAGDQHLKI TAQNLQGQQG KIQSNSDLQL NLGTANLDKA LTAAQSINLT ATELSHQQGQ LIQNDANGHL QVNVVQTLNN MSGVISAAGN ADIKTADLNN QSGVIQTLAN KDLSIESQKL
bay-D15  VHAGTQQLNI QTAQLNGHLG HILSNGTLQL KAGTLDVSGG VTSADQITIQ ANVLNNAQGQ LIQRGTSNPL NLTIQQQLNN QAGLIQSATA TDIKAGSLNN QRGTLSSAQG QNLDVD----

         1081                                                                                                               1200
 bau-D1  ---------- --QLNIVSQN DIQNDL-GNI VSEGQLSLKT GKGISNQQGN IISSNTLELT TDQL------ ---------- ------INNQ SGQIQA-KDI KLSASNLDNS SG--------
 nos-D2  ---------- --QLNIVSQN DIQNDL-GNI VSEGQLTLKA GKGLSNQQGS IISSNTLELT TDQL------ ---------- ------INNQ SGQIQA-KDI KLSASNLDNS SG--------
 bau-D3  ---------- --QLNIVSQN DIQNDL-GNI VSEGQLSLKT GKGISNQQGN IISSNTLELK TDQL------ ---------- ------INNQ SGQIQA-KDI KLSASNLDNS SG--------
 nos-D4  ---------- --QLNIVSQN DIQNDL-GNI VSEGQLTLKA GKGLSNQQGS IISSNTLELT TDQL------ ---------- ------INNQ SGQIQA-KDI KLSASNLDNS SG--------
 bau-D7  ---------- --QLNIVSQN DIQNDL-GNI VSEGQLSLKT GKGISNQQGN IISSNALELK TDQL------ ---------- ------INNQ SGQIQA-KDI KLSASNLDNS SG--------
 bau-D5  ---------- --QLNIVSQN DIQNDL-GNI VSEGQLSLKT GKGISNQQGN IISSNALELK TDQL------ ---------- ------INNQ SGQIQA-KDI KLSASNLDNS SG--------
 nos-D8  ---------- --QLNIVSQN DIQNDL-GNI VSEGQLSLKT GKGISNQQGN IISSNTLELT TDQL------ ---------- ------INNQ SGQIQA-KDI KLSASNLDNS SG--------
 bau-D9  ---------- --QLNIVSQN DIQNDL-GNI VSEGQLSLKT GKGISNQQGN IISSNALELK TDQL------ ---------- ------INNQ SGQIQA-KDI KLSASNLDNS SG--------
 bau-D6  ---------- --QLNIISQN DIQNDL-GNI VSEGQLSLKT GKGISNQQGN IISSNALELK TDQL------ ---------- ------INNQ SGQIQA-KDI KLSASNLDNS SG--------
nos-D12  ---------- --QLNIVSQN DIQNDL-GNI VSEGQLSLKT GKGISNQQGN IISSNTLELT TDQL------ ---------- ------INNQ SGQIQA-KDI KLSASNLDNS SG--------
bau-D10  LDNSQSGKIY AGQNGVIQAG SINNSLNGLI SAQNALTLTS LGIINNQSGK IVANQDVTLT SNGLDNSSGQ IGSSQGIVAI NAGSGVVNNT SGTLQAEKNL SVSADQINNQ SGLMNSQSSL
bau-D11  LDNSQSGKIY AGQSSVIQAG SINNSLNGLI SAQNALTLTS LGIINNQSGK IVANQDVTLT SNGLDNSSGQ IGSSQGMVAI NAGSGVVNNT SGTLQAEKDL SVSADQINNQ SGLINSQNSL
bau-D14  LDNSQSGKIY AGQSSVIQAG SINNSLNGLI SAQNALTLTS LGIINNQSGK IVANQDVTLT SNGLDNSSGQ IGSSQGMVAI NAGSGVVNNT SGTLQAEKDL SVSADQINNQ SGLINSQNSL
bau-D12  LDNSQSGKIY AGQSSVIQAG SINNSLNGLI SAQNALTLTS LGIINNQSGK IVANQDVTLT SNGLDNSSGQ IGSSQGMVAI NAGSGVVNNT SGTLQAEKDL SVSADQINNQ SGLINSQNSL
bau-D20  LDNSQSGKIY AGQNGVIQAG SIDNSLSGLI SAQNALTLTS LGIINNQSGK IVANQDVTLT SNGLDNSSGQ IGSSQGIVAI NAGSGVVNNT SGTLQAEKDL TVTADQINNQ SGLMNSQSSL
bau-D15  LDNSQAGKIY AGQDSIIQAG DVDNRLDGLI SAQNALTVTS LGVINNQSGK IIANSDVGIQ SEGLDNTGGQ IGSVQGGLTL DAGTGQLINQ SGSLQSAQDI QIKADQLKNE TGLISVQGSI
bau-D16  LDNSQSGKIY AGQNGVIQAG SIDNSLSGLI SAQNALTLTS LGIINNQSGK IVANQDVTLT SNGLDNSSGQ IGSSQGIVAI SAGSGVVNNT SGTLQAEKDL TVTADQINNQ SGLMNSQSSL
bau-D21  LDNSQSGKIY AGQNGIIQAG SINNSLNGLI SAQKALTLTS LGIINNQSGK IVANQDVTLT SNGLNNSNGQ IGTSQGMVAI NTGSGVFNNT SGTLQAEKDL TVTADQINNQ SGLMNSQSSL
bau-D19  LDNSQSGKIY AGQNGVIQAG SIDNSLSGLI SAQNALTLTS LGIINNQSGK IVANQDVTLT SNGLDNSSGQ IGSSQGIVAI SAGSGVVNNT SGTLQAEKDL TVTADQINNQ SGLMNSQSSL
bau-D13  LDNSQSGKIY AGRNGVIQAG SINNSLNGLI SAQNALTLTS LGIINNQSGK IVANQDVTLT SNGVDNSSGQ IGSSQGIVAI NAGSGVVNNT SGTLQAEKDL SVSADQINNQ SGLMNSQSSL
cal-D23  LDNSQSGKIY AGQNGVIQAG SIDNSLSGLI SAQNALTLTS LGIINNQSGK IVANQDVSIT SNGLDNSSGQ IGSSQGIVAI NAGSGVVNNT SGTLQAEKDL TVSADQINNQ SGLMNSQSSL
pit-D20  LDNSQSGKIY AGQNGVIQAG SIDNSLSGLI SAQNALTLTS LGIINNQSGK IVANQDVTLT SNGLDNSSGQ IGSSQGIVAI NAGSGVVNNT SGTLQAEKDL TVTADQINNQ SGLMNSQSSL
 pit-D6  LDNSQSGKIY AGQNGVIQAG SIDNSLSGLI SAQNALTLTS LGIINNQSGK IVANQDVTLT SNGLDNSSGQ IGSSQGIVAI NAGSGVVNNT SGTLQAEKDL TVTADQINNQ SGLMNSQSSL
bau-D17  LDNSQSGKIY AGQNGVIQAG SINNSLNGLI SAQNALTLTS LGIINNQSGK IVANQDVTLT SNGLDNSSGQ IGSSQGIVAI SAGSGVVNNT SGTLQAEKDL TVTADQINNQ SGLMNSQSSL
nos-D18  LDNSQSGQIY AGMNNNIHAG SINNSMAGQI SAQNALTLTS LGLINNQSGK IVANADVGIR SEGLDNTSGQ IGSVQAGLTL DVGKGKLTNQ SGSLQADKNI EIKADQLKTQ AGLISAQGII
nos-D22  LDNSQSGQIY AGLNNNIHAG SINNSMAGQI SAQNALTLTS LGLINNQSGK IVANADVGIR SEGLDNTNGQ IGSVQAGLTL DVGTGKLTNQ SGSLQADKNI EIKADQLKTQ AGLISAQGII
 bau-D2  --ENQSGKIV AGRDAAIKTT QLNND-TGTV YAAGKLGLHA TQDVSNQQGL IAAKQSLNIE AQNLNNSKGQ IQTESGDANL TIAQ-ILNNQ AGHIQAANAL NIVATQVENQ AG--------
bay-D15  ---------V AGKLD----- --NSQ-SGQI YAGNQAHLQV GSIDNSHQGQ INAQQNLQLT SQQ------- ---------- ---------- ---------- -----QINNQ SG--------

         1201                                                                                                               1320
 bau-D1  ---------- ----VIAAKT GDV-SLNINN Q--------- ---LINGRVE QGKSAGIIQA AQNIDVQTGL LDN-SGLIYA ---------- ---GQNQQLK VAQNLKNAGQ LAAQNNLNIQ
 nos-D2  ---------- ----VIAAKT GDV-SLNINN Q--------- ---LINGRVE QGKSAGIIQA AQNIDVQTGL LDN-SGLIYA ---------- ---GQDQQLK VAQNLKNAGQ LAAQNNLNIQ
 bau-D3  ---------- ----VIAAKT GDV-SLNINN Q--------- ---LINGRVE QAKSAGIIQA AQNIDVQTGL LDN-SGLIYA ---------- ---GQNQQLK VAQNLKNAGQ LAAQNNLNIQ
 nos-D4  ---------- ----VIAAKT GDV-SLNINN Q--------- ---LINGRVE QGKSAGIIQA AQNIDVQTGL LDN-SGLIYA ---------- ---GQDQQLK VAQNLKNAGQ LAAQNNLNIQ
 bau-D7  ---------- ----VIAAKT GDV-SLNINN Q--------- ---LINGRVE QAKSAGIIQA AQNIDVQTGL LDN-SGLIYA ---------- ---GQNQQLK VAQNLKNAGQ LAAQNNLNIQ
 bau-D5  ---------- ----MIAAKT GDV-SLNINN Q--------- ---LINGRVE QAKSAGIIQA AQNIDVQTGL LDN-SGLIYA ---------- ---GQNQQLK VAQNLKNAGQ LAAQNNLNIQ
 nos-D8  ---------- ----VIAAKT GDV-SLNINN Q--------- ---LINGRVE QGKSAGIIQA VQNIDVQTGL LDN-SGLIYA ---------- ---GQNQQLK VAQNLKNAGQ LAAQNNLNIQ
 bau-D9  ---------- ----VIAAKT GDV-SLNINN Q--------- ---LINGRVE QAKSAGIIQA AQNIDVQTGL LDN-SGLIYA ---------- ---GQNQQLK VAQNLKNAGQ LAAQNNLNIQ
 bau-D6  ---------- ----VIAAKT GDV-SLNINN Q--------- ---LINGRVE QAKSAGIIQA AQNIDVQTGL LDN-SGLIYA ---------- ---GQNQQLK VAQNLKNAGQ LAAQNNLNIQ
nos-D12  ---------- ----VIAAKT GDV-SLNINN Q--------- ---LINGRVE QGKSAGIIQA AQNIDVQTGL LDN-SGLIYA ---------- ---GQNQQLK VAQNLKNAGQ LAAQNNLNIQ
bau-D10  ALISRKDINN KSGQIIAKQD VTTQSQGLAN DLGQIGSVQG NVSLDAGLGD LSNQSGKILA AQDLTLSAQN LDNQSGLISA ---------- ---QKHLALK VQKVNNLKGQ IQSGDAIEIM
bau-D11  ALISRKDINN TSGQIIAKQD VTTYSQGLAN DLGQIGSVQG NVSLDAGLGD LSNQSGKILA AQDLTLSAQN LDNQSGLISA ---------- ---QKHLALK VQKVNNLKGQ IQSGDAIEIT
bau-D14  ALISRKDINN TSGQIIAKQD VTTYSQGLAN DLGQIGSVQG NVSLDAGLGD LSNQSGKILA AQDLTLSAQN LDNQSGLISA ---------- ---QKHLALK VQKVNNLKGQ IQSGDAIEIT
bau-D12  ALISRKDINN TSGQIIAKQD VTTYSQGLAN DLGQIGSVQG NVSLDAGLGD LSNQSGKILA AQDLTLSAQN LDNQSGLISA ---------- ---QKHLALK VQKVNNLKGQ IQSGDAIEIT
bau-D20  SLSSRKDVNN TSGQIIAKQD VTTHSQGLTN NSGQIGSVQG NVSLDAGLGD LNNQAGKILA AQDLTLSAQN LDNQSGLISA ---------- ---QKKLALK VQKLNNLKGQ IQSGDAIEIT
bau-D15  QAQITQDIDN SEGQIVANKA VLLSSQSLNN NAGLIGSVED IVNINAGSGV LSNQQGHIQS VKDLTLKAQG IDNQQGVIAT ---------- ---QAKLDMQ QQWLNNTKGQ IISGTALSFV
bau-D16  SLSSRKDVNN TSGQIIAKQD VTTHSQGLTN NSGQIGSVQG NVSLDAGLGD LNNQAGKILA AQDLTLSAQN LDNQSGLISA ---------- ---QKKLALK VQKLNNLKGQ IQSGDAIEIM
bau-D21  TLSSRKDVNN TSGQIIAKKD VTTHSQGLIN NSGQIGSVQG NVSLDTGLGD LSNQSGKILA AQDLTLSAQN LDNQSGLISA ---------- ---QKHLALK VQKVNNLKGQ IQSGDAIEIT
bau-D19  SLSSRKDVNN TSGQIIAKQD VTTHSQGLTN NSGQIGSVQG NVSLDAGLGD LNNQAGKILA AQDLTLSAQN LDNQSGLISA ---------- ---QKKLALK VQKLNNLKGQ IQSGDAIEIM
bau-D13  ALISRKDINS KSGQIIAKQD VTTQSQGLAN DLGQIGSVQG NVSLDAGLGD LSNQSGKILA AQDLTLSAQN LDNQSGLISA ---------- ---QKKLALK VQKLNNLKGQ IQSGDAIEIT
cal-D23  ALSSRKDVNN TSGQIIAKQD VTTQSQGLTN DLGQIGSVQG NVSLDAGLGD LSNQAGKILA AQDLTLSAQN LDNQSGFISA ---------- ---QKHLALK VQKLNNLKGQ IQSGDAIEIT
pit-D20  SLSSRKDVNN ASGQIIAKQD VTTHSQGLTN NSGQIGSVQG NVSLDVGLGD LNNQAGKILA AQDLTLSAQN LDNQSGLISA ---------- ---QKKLALK VQKLNNLKGQ IQSGDAIEIT
 pit-D6  SLSSRKDVNN TSGQIIAKQD VTTHSQGLTN NSGQIGSIQG NVSLDAGLGD LNNQAGKILA AQDLTLSAQN LDNQSGLISA ---------- ---QKKLALK VQKLNNLKGQ IQSGDAIEIT
bau-D17  SLSSRKDVNN TSGQIIAKQD VTTHSQGLTN NSGQIGSVQG NVSLDAGLGD LNNQAGKILA AQDLTLSAQN LDNQSGLISA ---------- ---QKKLALK VQKLNNLKGQ IQSGDAIEIT
nos-D18  QAQINQDIDN SSGQIVANKS VLLSSQGLNN NQGVIASIQD ELNINAGRLA LTNQAGLLQS GRNMNLITRG IDNSSGTIDS AGRLQMSGPI QTGQSTLSST TPWLMNTAGI IKMGTDFHLD
nos-D22  QAQINQDIDN SSGQIVANKS VLLSSQGLNN NQGVIASIQD ELNINAGRLA LTNQAGLLQS GRNMNLITRG IDNSSGTIDS AGRLQMSGPI QTGQSTLSST PPWLMNTAGI IKMGTDFHLD
 bau-D2  ---------- ---QLLSGTD TQLDVAHLNN QSGTIYSKKQ LDLNVSGSTD --NSAGTLAA DQNLNLNTQN LLNQAGQIRS EN-------- ---ADLKLNI AQDVQNNTGL ISAAKNLNLT
bay-D15  ---------- ---QIVANGD VQIQSQGLNN SHGQM----- ---------- -----GSLQG GLSLNTGSDT LINQSG---- ---------- ---------- ---------L IQAKKDIGIQ

         1321                                                                                                               1440
 bau-D1  TATLSQTSAG SIIAGLTSDG SLANQGNLNI DATGAI---- ---------- -NAQGQLIAG GDLSSQAANH VLNQSLVQAK NISLNSKSGQ LS-------- AQQSSIQAAQ QLNLTTPDTL
 nos-D2  TATLTQTSAG SIIAGLTSDG SLANQGNLNI DATGAI---- ---------- -SAQGQLIAG GDLSSQAASH VLNQSLVQAK NISLNSKSGQ LS-------- AQQSTIQAAQ QLNLTTPDTL
 bau-D3  TATLTQTSAG SIIAGLTSDG SLANQGNLNI DATGAI---- ---------- -NAQGQLIAG GDLNSQAASH VLNQSLVQAK NISLNSKSGQ LS-------- AQQSTIQAAQ QLNIVTPDTL
 nos-D4  TATLTQTSAG SIIAGLTSDG SLANQGNLNI DATGAI---- ---------- -SAQGQLIAG GDLSSQAASH VLNQSLVQAK NISLNSKSGQ LS-------- AQQSTIQAAQ QLNLTTPDTL
 bau-D7  TATLTQTSAG SIIAGLTSDG SLANQGNLNI DATGAI---- ---------- -NAQGQLIAG GDLNSQAASH VLNQSLVQAK NISLNSKSGQ LS-------- AQQSTIQAAQ QLNIVTPDTL
 bau-D5  TATLTQTSAG SIIAGLTSDG SLANQGNLNI DATGAI---- ---------- -NAQGQLIAG GDLNSQAASH VLNQSLVQAK NISLNSKSGQ LS-------- AQQSTIQAAQ QLNIVTPDTL
 nos-D8  TATLTQTSAG SIIAGLISDG SLANQGNLNI DATGAI---- ---------- -NAQGQLIAG GDLSSQAASH VLNQSLVQAK NISLNSKSGQ LS-------- AQQSTIQAAQ RLNIVTPDTL
 bau-D9  TATLTQTSAG SIIAGLTSDG SLANQGNLNI DATGAI---- ---------- -NAQGQLIAG GDLNSQAASH VLNQSLVQAK NISLNSKSGQ LS-------- AQQSTIQAAQ QLNIVTPDTL
 bau-D6  TATLTQTSAG SIIAGLTSDG SLANQGNLNI DATGAI---- ---------- -NAQGQLIAG GDLNSQAASH VLNQSLVQAK NISLNSKSGQ LS-------- AQQSTIQAAQ QLNIVTPDTL
```

```
nos-D12  TATLSQTSAG SIIAGLTSDG SLANQGNLNI DATGAI---- ---------- -SAQGQLIAG GDLNSQAASH VLNQSLVQAK NISLNSKSGQ LS-------- AQQSSIQAAQ QLNLTTPDTL
bau-D10  GQSLN-NQGG SIET--NADL TLNINGALDN SQSGKL-TGN TTKITAGSIN NSNKGQINAT DTLTVLSQQA INNQTGVMAA NQNVSIQSQG LDNTS----- GQIGSVQGSL TIDAQKEKLL
bau-D11  GQSLN-NQGG SIET--NADL TLNITDALDN SQSGKL-TGN TTKITAGSIN NSNKGQINAT DALTVLSQQE INNQTGVMAA NQNVSIQSQG LDNTS----- GQIGSVQGSL TIDAQKEKLL
bau-D14  GQSLN-NQGG SIET--NADL TLNITDALDN SQSGKL-TGN TTKITAGSIN NSNKGQINAT DALTVLSQQE INNQTGVMAA NQNVSIQSQG LDNTS----- GQIGSVQGSL TIDAQKEKLL
bau-D12  GQSLN-NQGG SIET--NADL TLNITDALDN SQSGKL-TGN TTKITAGSIN NSNKGQINAT DALTVLSQQE INNQTGVMAA NQNVSIQSQG LDNTS----- GQIGSVQGSL TIDAQKEKLL
bau-D20  GQSLN-NQGG SIET--NADL TLNIKGALDN SQSGKL-TGN TTEITAGSIN NINKGQINAT DTLTVLSQQG INNQTGIMAA NQNVSIQSQG LDNTS----- GQIGSVQGSL TIDAQKEKLL
bau-D15  GKDFN-NQGG LLQS--GADL NFKLSGLLDN SQSGQLASGG NTIIQAGSVK NSGQGKISAQ GVLNIDAVQA IDNQLGTMVA NQDITLTSQG LNNSQ----- GQIGSKNGSL MIDVGQGQLS
bau-D16  GQSLN-NQGG SIET--NADL TLNIKGALDN SQSGKL-TGN TTKITAGSIN NSNKGQINAI DTLTVLSQQA INNQTGVMAA NQNVSIQSQG LDNTS----- GQIGSVQGSL TIDAQKEKLL
bau-D21  GQSLN-NQGG SIET--NADL TLNITDALDN SQSGKL-TGN TTKITAGSIN NSNKGQINAT DALTVLSQQE INNQTGVMAA NQNVSIQSQG LDNTS----- GQIGSVQGSL TIDAQKEKLL
bau-D19  GQSLN-NQGG SIET--NADL TLNIKGALDN SQSGKL-TGN TTKITAGSIN NSNKGQINAI DTLTVLSQQA INNQTGVMAA NQNVSIQSQG LDNTS----- GQIGSVQGSL TIDAQKEKLL
bau-D13  GQSLN-NQGG SIET--NADL TLNIKGALDN SQSGKL-TGN TTKITAGSIN NSNKGQINAT DTLTVLSQQA INNQTGVMAA NQNVSIQSQG LDNTS----- GQIGSVQGSL TIDAQKEKLL
cal-D23  GQSLN-NQGG SIET--NADL TLNINAALDN SQSGKL-TGN TTKITAGSIN NSNKGQINAT DALTVLSQQA INNQAGVMTA NQNVSIQSQG LDNTG----- GQIGSVEGSL TLDAKKEKLL
pit-D20  GQSLN-NQGG SIET--NADL TLNIKGALDN SQSGKL-TGN TTEITAGSIN NSNKGQINAT DTLTVLSQQG INNQTGVMAA NQNVSIQSQG LDNTS----- GQIGSVQGSL TIDAQKEKLL
 pit-D6  GQSLN-NQGG SIET--NADL TLNIKGALDN SQSGKL-TGN TTEFTAGSIN NSNKGQINAT DTLTVLSQQG INNQTGVMAA NQNVSIQSQG LDNTS----- GQIGSVQGSL TIDAQKEKLL
bau-D17  GQSLN-NQGG SIET--NADL TLNIKGALDN SQSGKL-TGN TTKITAGSIN NSNKGQINAT DTLTVLSQQA INNQTGVMAA NQNVSIQSQG LDNTS----- GQIGSVQGSL TIDAQKEKLL
nos-D18  ASQFD-NRGG TLQTRDGYDL SLNITGQLDN SQGGQLQSGH DLMIASGSMN NEH-GHLSAQ GLASVKADHG LDNTAGSIIA NADVNVSNQG LNRTSTINDQ GTIGSVQGQL TVDAGQGLLS
nos-D22  ASQFD-NRGG TLQTRDGYDL SLNITGQLDN SQGGQLQSGH DLMIASGSMN NEH-GHLSAQ GLASVKADHG LDNTAGSIIA NADVNVSNQG LNRTSTINDQ GTIGSVQGQL TIDAGQGLLS
 bau-D2  AKNIT-SQQG KVQSGANANI QLNNF----- ---------- ---------- DNTEGVVYAE EQLQLSATGK LNNTQGIVAA EQSTDIRAGS VINDA----- GQIRSQQDQL KLNVQQD-IS
bay-D15  AQRID-NQQG QI-------- ---------- ---------- ---------- -TTQG----- -QAELISQAE IDNLEGAIAA DLDVNIRSQG LNNNK----- GQISSAQGNL TLNAG-----

         1441                                                                                                                   1560
 bau-D1  DTQQANLKAN KVQINAKDLN NQSGHIQQTG LDDTQILLTG DLSNQNGQID SLGQKLQINA NNLNNQNGLI QSGTADSQLI LAIA------ ---------- ---------- ----------
 nos-D2  DTQQANLKAN KVQINAKDLN NQSGHIQQTG LDHTQILLTG DLSNQNGQID SLGQKLQINA NNLNNQNGLI QSGTTDSQLI LAIA------ ---------- ---------- ----------
 bau-D3  DTQQANLKAN KVQINAKDLN NQSGHIQQTG LDDTQILLTG DLSNQNGQID SLGQKLQINA NNLNNQNGLI QSGTADSQLI LAIA------ ---------- ---------- ----------
 nos-D4  DTQQANLKAN KVQINAKDLN NQSGHIQQTG LDHTQILLTG DLSNQNGQID SLGQKLQINA NNLNNQNGLI QSGTTDSQLI LAIA------ ---------- ---------- ----------
 bau-D7  DTQQANLKAN KVQINAKDLN NQSGHIQQTG LDDTQILLTG DLSNQNGQID SLGQKLQINA NNLNNQNGLI QSGTADSQLI LAIA------ ---------- ---------- ----------
 bau-D5  DTQQANLKAN KVQINAKDLN NQSGHIQQTG LDDTQILLTG DLSNQNGQID SLGQKLQINA NNLNNQNGLI QSGTAGSQLI LAIA------ ---------- ---------- ----------
 nos-D8  DTQQANLKAN KVQINAKDLN NQSGHIQQTG LNDTQILLTG DLSNQNGQID SLGQKLQINA NNLNNQNGLI QSGTADSQLI LAIA------ ---------- ---------- ----------
 bau-D9  DTQQANLKAN KVQINAKDLN NQSGHIQQTG LDDTQILLTG DLSNQNGQID SLGQKLQINA NNLNNQNGLI QSGTAGSQLI LAIA------ ---------- ---------- ----------
 bau-D6  DTQQANLKAN KVQINAKDLN NQSGHIQQTG LDDTQILLTG DLSNQNGQID SLGQKLQINA NNLNNQNGLI QSGTADSQLI LAIA------ ---------- ---------- ----------
nos-D12  DTQQANLKAN KVQINAKDLN NQSGHIQQTG LDDTQILLTG DLSNQNGQID SLGQKLQINA NNLNNQNGLI QSGTADSQLI LAIA------ ---------- ---------- ----------
bau-D10  NTSGSLQAGT SLKITSGGLN NDSGTI--LA LTDNTITSTD DISNKAGKIA SNAHTTITTQ N-LNNQAGMI QSGSGSALDL VINGALDNSQ AGHLLSG--- ---------- -------AGL
bau-D11  NTSGSLQAGT SLKITSGGLN NDSGTI--LA LTDNTITSTD DISNKAGKIA SNAHTTITTQ N-LNNQAGMI QSGSGSALDL VINGALDNSQ AGHLLSG--- ---------- -------AGL
bau-D14  NTSGSLQAGT SLKITSGGLN NDSGTI--LA LTDNTITSTD DISNKAGKIA SNAHTTITTQ N-LNNQAGMI QSGSGSALDL VINGALDNSQ AGHLLSG--- ---------- -------AGL
bau-D12  NTSGSLQAGT SLKITSGGLN NDSGNI--LA LTDNTITSTD DISNKAGKIA SNAHTTITTQ N-LNNQAGMI QSGSGSALDL VINGALDNSQ AGHLLSG--- ---------- -------AGL
bau-D20  NTSGSLQAGT SLKITSGGLN NDSGTI--LA LTDNTITSTD DISNKAGKIA SNAHTTITSQ N-LTNQAGTI QSGSGSALDL VINGALDNSQ AGQLLSG--- ---------- -------AGL
bau-D15  NQAGSLQAGT ELHLQAAGIN NDSGIV--GS LGNSHILSSG DISNNAGQIA TNATLQVNGQ N-LTNQTGII QSGSGSALDL VLNGTLDNSQ AGQLLSG--- ---------- -------AGL
bau-D16  NTSGSLQAGT SLKITSGDLN NDSGTI--LA LTDNTITSTG DVSNKAGKIA SNAHTTVTTQ N-LTNQAGTI QSGSGSALDL VINGALDNSQ AGQLLSG--- ---------- -------ASL
bau-D21  NTSGSLQAGT SLKITSGGLN NDSGTI--LA LTDNTITSTD DISNKAGKIA SNAHTTITTQ N-LNNQAGMI QSGSGLALDL VINGALDNSQ AGHLLSG--- ---------- -------AGL
bau-D19  NTSGSLQAGT SLKITSGDLN NDSGTI--LA LTDNTITSTG DVSNKAGKIA SNAHTTVTTQ N-LTNQAGTI QSGSGSALDL VINGALDNSQ AGQLLSG--- ---------- -------ASL
bau-D13  NTSGSLQAGT SLKITSGDLN NDSGTI--LA LTDNTITSTG DVSNKAGKIA SNAHTTVTTQ N-LNNQAGMI QSGSGSALDL VINGALDNSQ AGHLLSG--- ---------- -------AGL
cal-D23  NTSGSIQAGT SLKITSGGLN NDSGTI--LA LSDNTITSTG DINNKAGKIA SNAHTTITSQ N-LTNQAGTI QSGSGSALDL VINGALDNSQ AGQLLSG--- ---------- -------AGL
pit-D20  NTSGSLQAGT SLKITSGGLN NDSGTI--LA LTDNTITSTG DISNKAGKIA SNAHTTITSQ N-LTNQAGTI QSGSGSALDL VINGALDNSQ AGQLLSG--- ---------- -------AGL
 pit-D6  NTSGSLQAGT SLKITSGGLN NDSGTI--LA LTDNTITSTG DISNKAGKIA SNAHTTITSQ N-LTNQAGTI QSGSGSALDL VINGALDNSQ AGQLLSG--- ---------- -------AGL
bau-D17  NTSGSLQAGT SLKITSGDLN NDSGTI--LA LTDNTVTSTG DISNKAGKIA SNAHTTVTTQ N-LTNQAGTI QSGSGSALDL VINGALDNSQ AGQLLSG--- ---------- -------ASL
nos-D18  NQHGTLQAAT RLDLNAADID STSGSI--QA QDDIQIISAT ALNNQSGHIV TTAGSIIATA NSLDNQAGLI LSNPQHDITL TVSDLLDNRQ QGQINSGHRI GISAGQLNNN SGQITAQSTA
nos-D22  NQHGTLQAAT RLDLNAADID STSGSI--QA QDDIQIISAT ALNNQSGHIV TTAGSIIATA NSLDNRAGLI LSNPQHDITL TVSDLLDNRQ QGQINSGHRI GISAGQLNNN SGQITAQSTA
 bau-D2  NQNGEISAAK SIELNAQKVS NQKGKV--IA ---------- ---------- ---------- ---------- ----GESLNV TAQ------- ---------- ---------- ----------
bay-D15  --NGTLT--- ---------- NQAATI--VS ---------- ---------- ---------- ---------- ----GKTLTL SAN------- ---------- ---------- ----------

         1561                                                                                                                   1680
 bau-D1  ---------- ---------- ---------- ---------- ---------- ---------- ---------N TLNNNQGNIK TAGSLQTNSS EL-NNDAGTL LSFNGFNINT QQLSNQAGQI
 nos-D2  ---------- ---------- ---------- ---------- ---------- ---------- ---------N TLNNNQGNIK TAGSLQTNSS EL-NNDAGTL LSFNGFNINT QQLSNQAGQI
 bau-D3  ---------- ---------- ---------- ---------- ---------- ---------- ---------N TLNNNQGNIK TAGSLQTNSS EL-NNDAGTL LSFNGFNINT QQLSNQAGQI
 nos-D4  ---------- ---------- ---------- ---------- ---------- ---------- ---------N TLNNNQGNIK TAGSLQTNSS EL-NNDAGTL LSFNGFNINT QQLSNQAGQI
 bau-D7  ---------- ---------- ---------- ---------- ---------- ---------- ---------N TLNNNQGNIK TAGSLQTNSS EL-NNDAGTL LSFNGFNINT QQLSNQAGQI
 bau-D5  ---------- ---------- ---------- ---------- ---------- ---------- ---------N TLNNNQGNIK TAGSLQTNSS EL-NNDAGTL LSFNGFNINT QQLSNQAGQI
 nos-D8  ---------- ---------- ---------- ---------- ---------- ---------- ---------N TLNNNQGNIK TAGSLQTNSS EL-NNDAGTL LSFNGFNINT QQLSNQAGQI
 bau-D9  ---------- ---------- ---------- ---------- ---------- ---------- ---------N TLNNNQGNIK TAGSLQTNSS EL-NNDAGTL LSFNGFNINT QQLSNQAGQI
 bau-D6  ---------- ---------- ---------- ---------- ---------- ---------- ---------N TLNNNQGNIK TAGSLQTNSS EL-NNDAGTL LSFNGFNINT QQLSNQAGQI
nos-D12  ---------- ---------- ---------- ---------- ---------- ---------- ---------N TLNNNQGNIK TAGSLQTNSS EL-NNDAGTL LSFNGFNINT QQLSNQAGQI
bau-D10  NLKVN----- ---------- ---------- ---------- ---------- ---------- ---------T LDNSQQGQIS AQDALNIISA GLINNQAGTL VANQNVTLSS KGLNNNQGQI
bau-D11  NLKVN----- ---------- ---------- ---------- ---------- ---------- ---------T LDNSQQGQIS AQDALNIISA GLINNQAGTL VANQNVTLSS KGLNNNQGQI
bau-D14  NLKVN----- ---------- ---------- ---------- ---------- ---------- ---------T LDNSQQGQIS AQDALNIISA GLINNQAGTL VANQNVTLSS KGLNNNQGQI
bau-D12  NLKVN----- ---------- ---------- ---------- ---------- ---------- ---------T LDNSQQGQIS AQDALNIISA GLINNQAGTL VANQNVTLSS KGLNNNQGQI
bau-D20  NLKVN----- ---------- ---------- ---------- ---------- ---------- ---------T LDNSQQGQIS AQDALNIINA GLINNQAGTL VANQNVTLSS KGLNNNQGQI
bau-D15  NLQVN----- ---------- ---------- ---------- ---------- ---------- ---------A LDNSQQGQIS AQDALNIMSA GLINNQTGTI VANQDVTLNS QGLNNNQGQI
bau-D16  NLQVN----- ---------- ---------- ---------- ---------- ---------- ---------S LDNSQQGQIS AQDALNIISA GLINNQAGTL VANQNVTLSS EGLNNNQGQI
bau-D21  NLKVN----- ---------- ---------- ---------- ---------- ---------- ---------T LDNSQQGQIS AQDALNIISA GLINNQAGTL VANQNVTLSS KGLNNNQGQI
bau-D19  NLQVN----- ---------- ---------- ---------- ---------- ---------- ---------S LDNSQQGQIS AQDALNIISA GLINNQAGTL VANQNVTLSS EGLNNNQGQI
bau-D13  NLKVN----- ---------- ---------- ---------- ---------- ---------- ---------T LDNSQQGQIS AQDALNIISA GLINNQAGTL VANQNVTLSS KGLNNNQGQI
cal-D23  NLQVN----- ---------- ---------- ---------- ---------- ---------- ---------S LDNNQQGQIS AQDALNIISA GLINNQAGTL VANQNVTLNS EGLNNNHGQI
pit-D20  NLKVN----- ---------- ---------- ---------- ---------- ---------- ---------T LDNSQQGQIS AQDALNIIST GLINNQAGTL VANQNVTLSS KGLNNNQGQI
 pit-D6  NLKVN----- ---------- ---------- ---------- ---------- ---------- ---------T LDNSQQGQIS AQDALNIIST GLINNQAGTL VANQNVTLNS NGLNNNQGQI
bau-D17  NLQVN----- ---------- ---------- ---------- ---------- ---------- ---------S LDNSQQGQIS AQDALNIISA GLINNQTGTL VANQNVTLSS EGLNNNQGQI
nos-D18  NLNINQDINN NNGSIVAYDD VTTTSVGVNN DAGKISSAKG LLSIDAGTGV LSSQVGTLQA GTNLTAAAAS INNTAQGLIS ALNAVTLTST GLINNDTGSI IANHDVNLTS QGLSNHAGEI
nos-D22  NLNINQDINN NNGSIVAYDD VTTTSVGVNN DAGKISSAKG LLSIDAGTGV LSSQVGTLQA GTNLTAAAAS INNTAQGLIS ALNAVTLTST GLINNDTGSI IANHDVNLTS QGLSNHAGEI
 bau-D2  ---------- ---------- ---------- ---------- ---------- ---------- ---------Q VD-SIEGTIY AKEQLHLTVA DQFNNQSGTI AANQLVQIQA KNLNNTAGKI
bay-D15  ---------- ---------- ---------- ---------- ---------- ---------- ---------Q LDNSNHGQIN SQNNADISIV KDINNTSGVI AAKQQLILSS QGLNNTTGQI

         1681                                                                                                                   1800
 bau-D1  --------VE AGTQ------ ---------- ---------- ---------- ---------- ---------- ---------- ---------- ---------- ---------- ----------
 nos-D2  --------VE AGTQ------ ---------- ---------- ---------- ---------- ---------- ---------- ---------- ---------- ---------- ----------
 bau-D3  --------VE AGTQ------ ---------- ---------- ---------- ---------- ---------- ---------- ---------- ---------- ---------- ----------
 nos-D4  --------VE AGTQ------ ---------- ---------- ---------- ---------- ---------- ---------- ---------- ---------- ---------- ----------
 bau-D7  --------VE AGTQ------ ---------- ---------- ---------- ---------- ---------- ---------- ---------- ---------- ---------- ----------
 bau-D5  --------VE AGTQ------ ---------- ---------- ---------- ---------- ---------- ---------- ---------- ---------- ---------- ----------
 nos-D8  --------VE AGTQ------ ---------- ---------- ---------- ---------- ---------- ---------- ---------- ---------- ---------- ----------
 bau-D9  --------VE AGTQ------ ---------- ---------- ---------- ---------- ---------- ---------- ---------- ---------- ---------- ----------
 bau-D6  --------VE AGTQ------ ---------- ---------- ---------- ---------- ---------- ---------- ---------- ---------- ---------- ----------
nos-D12  --------VE AGTQ------ ---------- ---------- ---------- ---------- ---------- ---------- ---------- ---------- ---------- ----------
bau-D10  GSIQGGLAID AGDQALTNQS GVLQAKTDLT AKALSIDSTE GQIISQAKID LQSLKEINNQ QGIISADQGI QVKSTGLNNN LGQISSAQGE IVLNAGQGLL SNQTGKIIAG QALQLTADQF
bau-D11  GSIQGGLAID AGDQALTNQS GVLQAKTDLT AKALSIDSTE GQIISQAKID LQSLKEINNQ QGIISADQGI QVKSTGLNNN LGQISSAQGE IVLNAGQGLL SNQTGKIIAG QALQLTADQF
bau-D14  GSIQGGLAID AGDQALTNQS GVLQAKTDLT AKALSIDSTE GQIISQAKID LQSLKEINNQ QGIISADQGI QVKSTGLNNN LGQISSAQGE IVLNAGQGLL SNQTGKIIAG QALQLTADQF
bau-D12  GSIQGGLAID AGDQALTNQS GVLQAKTDLT AKALSIDSTE GQIISQAKID LQSLKEINNQ QGIISADQGI QVKSTGLNNN LGQISSAQGE IVLNAGQGLL SNQTGKIIAG QALQLTADQF
bau-D20  GSIQGGLAID AGDQALTNQS GVLQAKTDLT AKALSMDSTE GQIISQAKID LQSLKEINNQ QGIISADQGI QVKSTGLNNN LGQISSAQGE IVLNAGQGLL SNQTGKIIAG QALQLTADQF
bau-D15  GSIQGGLTVD TGNQALSNKA GTLQAKTDLT AKALSIDSTE GQIISQAKID LQSQKEINNQ QGIISADQGI QVNSQGLNNN LGNISSAQGN IVLNAGQGVL SNQTGKVIAG QALQLSAGQV
bau-D16  GSIQGGLAID AGDQALTNQS GILQAKSDVT IKALSIDSTE GQIISQAKVD VQSQKEVNNQ QGLISADQGI QVNSQGLNNN LGNISSAQGN IVLNAGQGVL SNQTGKVIAG QALQLSAGQV
bau-D21  GSIQGGLAID AGDQALTNQS GVLQAKTDLT AKALSIDSTE GQIISQAKID LQSLKEINNQ QGIISADQGI QVKSTGLNNN LGQISSAQGN IVLNAGQGVL SNQTGKVIAG QALQLSAGQV
bau-D19  GSIQGGLAID AGDQALTNQS GILQAKSDVT IKALSIDSTE GQIISQAKVD VQSQKEVNNQ QGLISADQGI QVNSQGLNNN LGNISSAQGN IVLNAGQGVL SNQTGKVIAG QALQLSAGQV
bau-D13  GSIQGGLVID AGDQALTNQS GVLQAKTDLT AKALS----- ---------- ---------- ---------- ---------- ---------- ---------- ---------- ----------
cal-D23  GSIQGGLAID SGDLALTNQS GVLQAKTDLT AKALS----- ---------- ---------- ---------- ---------- ---------- ---------- ---------- ----------
pit-D20  GSIQGGLVID VGDQALTNQS GVLQAKTDLT AKALS----- ---------- ---------- ---------- ---------- ---------- ---------- ---------- ----------
 pit-D6  GSIQGGLAID AGDQALTNQS GVLQAKTDLT AKALS----- ---------- ---------- ---------- ---------- ---------- ---------- ---------- ----------
bau-D17  GSIQGGLAID AGDQALINQS GILQAKTDLT GKALS----- ---------- ---------- ---------- ---------- ---------- ---------- ---------- ----------
nos-D18  GSITGVLNAN AGNAALSNQS GRIQAATAIA LTAVGIDNTA GNISSQGTAT VDSRQIITNQ SGRLIAHDNL NVSSEGLNNN MGQIGSANGL LNLNTGNGAL SSRSGKLQAA TAATVIAHDI
nos-D22  GSITGVLNAN AGNAALSNQS GRIQAATAIA LTAVGIDNTA GNISSQGTAT VDSRQIITNQ SGRLIANNNL NVTSEGLNNN LGQIGSANGL LNLNTGNGAL SSRSGKLQAA TTATVIAHDI
 bau-D2  RS-------- ---------- ---------- ---------- ---------- ---------- ---------- ---------- ---------- ---------- ---------- ----------
bay-D15  ---------- ---------- ---------- ---------- ---------- ---------- ---------- ---------- ---------- ---------- ---------- ----------

         1801                                                                                                                   1920
 bau-D1  ---------- ---------- ---------- ---------- ---------- ---------- ---------- ---------- ---------- ---------- ---------- ----------
 nos-D2  ---------- ---------- ---------- ---------- ---------- ---------- ---------- ---------- ---------- ---------- ---------- ----------
 bau-D3  ---------- ---------- ---------- ---------- ---------- ---------- ---------- ---------- ---------- ---------- ---------- ----------
 nos-D4  ---------- ---------- ---------- ---------- ---------- ---------- ---------- ---------- ---------- ---------- ---------- ----------
```

```
 bau-D7  ---------- ---------- ---------- ---------- ---------- ---------- ---------- ---------- ---------- ---------- ---------- ----------
 bau-D5  ---------- ---------- ---------- ---------- ---------- ---------- ---------- ---------- ---------- ---------- ---------- ----------
 nos-D8  ---------- ---------- ---------- ---------- ---------- ---------- ---------- ---------- ---------- ---------- ---------- ----------
 bau-D9  ---------- ---------- ---------- ---------- ---------- ---------- ---------- ---------- ---------- ---------- ---------- ----------
 bau-D6  ---------- ---------- ---------- ---------- ---------- ---------- ---------- ---------- ---------- ---------- ---------- ----------
nos-D12  ---------- ---------- ---------- ---------- ---------- ---------- ---------- ---------- ---------- ---------- ---------- ----------
bau-D10  DNSQ------ ---------- ---------- ---------- ---------- ---------- ---------- ---------- ---------Q GQLNSQTTLD IQTKKDINNQ SGIIAANQKV
bau-D11  DNSQ------ ---------- ---------- ---------- ---------- ---------- ---------- ---------- ---------Q GQLNSQTTLD IQTKKDINNQ SGIIAANQKV
bau-D14  DNSQ------ ---------- ---------- ---------- ---------- ---------- ---------- ---------- ---------Q GQLNSQTTLD IQTKKDINNQ SGIIAANQKV
bau-D12  DNSQ------ ---------- ---------- ---------- ---------- ---------- ---------- ---------- ---------Q GQLNSQTTLD IQTKKDINNQ SGIVAANQKV
bau-D20  DNSQ------ ---------- ---------- ---------- ---------- ---------- ---------- ---------- ---------Q GQLNSQTTLD IQTKKDINNQ SGIIAANQKV
bau-D15  DNSQ------ ---------- ---------- ---------- ---------- ---------- ---------- ---------- ---------T GQLNSQTTLD IQATKDINNQ SGIIAANQQV
bau-D16  DNSQ------ ---------- ---------- ---------- ---------- ---------- ---------- ---------- ---------T GQLNSQTTLD IQATKDINNQ SGIIAANQQV
bau-D21  DNSQ------ ---------- ---------- ---------- ---------- ---------- ---------- ---------- ---------T GQLNSQTTLD IQATKDINNQ SGIIAANQQV
bau-D19  DNSQ------ ---------- ---------- ---------- ---------- ---------- ---------- ---------- ---------T GQLNSQTTLD IQATKDINNQ SGIIAANQQV
bau-D13  ---------- ---------- ---------- ---------- ---------- ---------- ---------- ---------- ---------- ---------- ---------- ----------
cal-D23  ---------- ---------- ---------- ---------- ---------- ---------- ---------- ---------- ---------- ---------- ---------- ----------
pit-D20  ---------- ---------- ---------- ---------- ---------- ---------- ---------- ---------- ---------- ---------- ---------- ----------
 pit-D6  ---------- ---------- ---------- ---------- ---------- ---------- ---------- ---------- ---------- ---------- ---------- ----------
bau-D17  ---------- ---------- ---------- ---------- ---------- ---------- ---------- ---------- ---------- ---------- ---------- ----------
nos-D18  DNTSGLIDAQ GNVLLTSQTG LVNQGGQIES ATGTINASAV TLNNQAGLIQ TNPDNDIHLN IVGMLDNGQK GQILSGANAL LQSGQLNNST GQISAQRTAT VKTAQDINNT AGNIVANGDV
nos-D22  DNTSGLIDAQ GNVLLTSQTG LVNQGGQIES ATGTINASAA TLNNQAGLIQ TNPDNDIHLT IIGMLDNGQK GQILSGANAL LQLGQLNNST GQISAQGTAT VKTAQDINNT AGNIVANGDV
 bau-D2  ---------- ---------- ---------- ---------- ---------- ---------- ---------- ---------- ---------- ---------- ---------- ----------
bay-D15  ---------- ---------- ---------- ---------- ---------- ---------- ---------- ---------- ---------- ---------- ---------- ----------

         1921                                                                                                                    2040
 bau-D1  ---------- ---------- ---------- ---------- ---------- ---------- ---------- ---------- ---------- ---------- ---------- ----------
 nos-D2  ---------- ---------- ---------- ---------- ---------- ---------- ---------- ---------- ---------- ---------- ---------- ----------
 bau-D3  ---------- ---------- ---------- ---------- ---------- ---------- ---------- ---------- ---------- ---------- ---------- ----------
 nos-D4  ---------- ---------- ---------- ---------- ---------- ---------- ---------- ---------- ---------- ---------- ---------- ----------
 bau-D7  ---------- ---------- ---------- ---------- ---------- ---------- ---------- ---------- ---------- ---------- ---------- ----------
 bau-D5  ---------- ---------- ---------- ---------- ---------- ---------- ---------- ---------- ---------- ---------- ---------- ----------
 nos-D8  ---------- ---------- ---------- ---------- ---------- ---------- ---------- ---------- ---------- ---------- ---------- ----------
 bau-D9  ---------- ---------- ---------- ---------- ---------- ---------- ---------- ---------- ---------- ---------- ---------- ----------
 bau-D6  ---------- ---------- ---------- ---------- ---------- ---------- ---------- ---------- ---------- ---------- ---------- ----------
nos-D12  ---------- ---------- ---------- ---------- ---------- ---------- ---------- ---------- ---------- ---------- ---------- ----------
bau-D10  NLNSQGLNNN QGQIVSLNDA LTVNSGSGVL DNQSGTLQAK GDIQLNAEQV ---------- ---------- ---------- ---------- ---------- ---------- ----------
bau-D11  NLNSQGLNNN KGQIVSLNDA LTVNSGTSVL DNQSGVLQAK GNIRIDAEQV ---------- ---------- ---------- ---------- ---------- ---------- ----------
bau-D14  NLNSQGLNNN KGQIVSLNDA LTVNSGTSVL DNQSGVLQAK GNIRIDAEQV ---------- ---------- ---------- ---------- ---------- ---------- ----------
bau-D12  NLNSQGLNNN KGQIVSLNDA LTVNSGTSVL DNQSGVLQAK GNIRIDAEQV ---------- ---------- ---------- ---------- ---------- ---------- ----------
bau-D20  NLNSQGLNNN KGQIVSLNDA LTVNSGTSVL DNQSGVLQAK GNIRIDAEQV ---------- ---------- ---------- ---------- ---------- ---------- ----------
bau-D15  NLNSQGLNNN KGQIVSLNDA LTVNSGTGAL DNQSGVLQSK SNIQIDAEQV ---------- ---------- ---------- ---------- ---------- ---------- ----------
bau-D16  NLNSQGLNNN QGQIVSLNDA LTVNSGSGVL DNQSGTLQAK GDIQLNAEQV ---------- ---------- ---------- ---------- ---------- ---------- ----------
bau-D21  NLNSQGLNNN QGQIVSLNDA LTVNSGSGVL DNQSGTLQAK GDIQLNAEQV ---------- ---------- ---------- ---------- ---------- ---------- ----------
bau-D19  NLNSQGLNNN QGQIVSLNDA LTVNSGSGVL DNQSGTLQAK GDIQLNAEQV ---------- ---------- ---------- ---------- ---------- ---------- ----------
bau-D13  ---------- ---------- ---------- ---------- ---------- ---------- ---------- ---------- ---------- ---------- ---------- ----------
cal-D23  ---------- ---------- ---------- ---------- ---------- ---------- ---------- ---------- ---------- ---------- ---------- ----------
pit-D20  ---------- ---------- ---------- ---------- ---------- ---------- ---------- ---------- ---------- ---------- ---------- ----------
 pit-D6  ---------- ---------- ---------- ---------- ---------- ---------- ---------- ---------- ---------- ---------- ---------- ----------
bau-D17  ---------- ---------- ---------- ---------- ---------- ---------- ---------- ---------- ---------- ---------- ---------- ----------
nos-D18  NLTSQGLNNN QGMIGSAQGL LVIEAGRDIL SSQGGHLQSA KTLNLSANSI DNTAGSIEAV GDISLSSQKA LINQTGQIAS TAGNIKVIAS DLNNQTGRIQ SNSGFDVGLS VSNLLDNSQT
nos-D22  NLTSQGLNNN QGMIGSAQGL LAIEAGRGIL SSQGGHLQSA KTLNLSANSI DNTAGSIEAV GDISLSSQKA LINQTGQIAS TAGNINVIAS DLNNQTGRIQ SNSGFDVGLS VSDLLDNSQT
 bau-D2  ---------- ---------- ---------- ---------- ---------- ---------- ---------- ---------- ---------- ---------- ---------- ----------
bay-D15  ---------- ---------- ---------- ---------- ---------- ---------- ---------- ---------- ---------- ---------- ---------- ----------

         2041                                                                                                                    2160
 bau-D1  ---------- ---------- ---------- ---------- ---------- ---------- ---------- ---------- ---------- ---------- ---------- ----------
 nos-D2  ---------- ---------- ---------- ---------- ---------- ---------- ---------- ---------- ---------- ---------- ---------- ----------
 bau-D3  ---------- ---------- ---------- ---------- ---------- ---------- ---------- ---------- ---------- ---------- ---------- ----------
 nos-D4  ---------- ---------- ---------- ---------- ---------- ---------- ---------- ---------- ---------- ---------- ---------- ----------
 bau-D7  ---------- ---------- ---------- ---------- ---------- ---------- ---------- ---------- ---------- ---------- ---------- ----------
 bau-D5  ---------- ---------- ---------- ---------- ---------- ---------- ---------- ---------- ---------- ---------- ---------- ----------
 nos-D8  ---------- ---------- ---------- ---------- ---------- ---------- ---------- ---------- ---------- ---------- ---------- ----------
 bau-D9  ---------- ---------- ---------- ---------- ---------- ---------- ---------- ---------- ---------- ---------- ---------- ----------
 bau-D6  ---------- ---------- ---------- ---------- ---------- ---------- ---------- ---------- ---------- ---------- ---------- ----------
nos-D12  ---------- ---------- ---------- ---------- ---------- ---------- ---------- ---------- ---------- ---------- ---------- ----------
bau-D10  ---------- ------NSQS GLISSEAGID LQVNQLVDNS SGQIIANNAV QIQSQGLKNN Q--------- GQIASVNDVL NINSGSGTLE NQAGLLQAQG DIQLTADRLL NQSGLINTQG
bau-D11  ---------- ------NSQS GLISSEGGIN LQAHQLVDNS SGQIIANNAV QIQSLGLKNN L--------- GQIASVNDVL NINSGSGTLE NQAGLLQAQG DIQLTADRLL NQSGLINTQG
bau-D14  ---------- ------NSQS GLISSEGGIN LQAHQLVDNS SGQIIANNAV QIQSLGLKNN L--------- GQIASVNDVL NINSGSGTLE NQAGLLQAQG DIQLTADRLL NQSGLINTQG
bau-D12  ---------- ------NSQS GLISSEGGIN LQAHQLVDNS SGQIIANNAV QIQSLGLKNN L--------- GQIASVNDVL NINSGSGTLE NQAGLLQAQG DIQLTADRLL NQSGLINTQG
bau-D20  ---------- ------NSQS GLISSEGRIN LQAHQLVDNS SGQIIANNAV QIQSQGLKNN Q--------- GQIASVNDVL NINSGSGTLE NQAGLLQAQG DIQLTSDRLL NQSGLINTQG
bau-D15  ---------- ------NSQS GLISSEDRID LQAHQLVDNS SGQIIANNAV QIQSLGLNNN Q--------- GQIASVNDVL NINSGSGELQ NQAGLLQAQG DIQLIADRLL NQSGLINTQG
bau-D16  ---------- ------NSQS GLISSEAGID LQVNQLVDNS SGQIIANNAV QIQSQGLKNN Q--------- GQIASVNDVL NINSGSGTLE NQAGLLQAQG DIQLTADRLL NQSGLINTQG
bau-D21  ---------- ------NSQS GLISSEAGID LQVNQLVDNS SGQIIANNAV QIQSQGLKNN Q--------- GQIASVNDVL NINSGSGTLE NQAGLLQAQG DIQLTADRLL NQSGLINTQG
bau-D19  ---------- ------NSQS GLISSEAGID LQVNQLVDNS SGQIIANNAV QIQSQGLKNN Q--------- GQIASVNDVL NINSGSGTLE NQAGLLQAQG DIQLTADRLL NQSGLINTQG
bau-D13  ---------- ---------- ---------- ---------- ---------- ---------- ---------- ---------- ---------- ---------- ---------- ----------
cal-D23  ---------- ---------- ---------- ---------- ---------- ---------- ---------- ---------- ---------- ---------- ---------- ----------
pit-D20  ---------- ---------- ---------- ---------- ---------- ---------- ---------- ---------- ---------- ---------- ---------- ----------
 pit-D6  ---------- ---------- ---------- ---------- ---------- ---------- ---------- ---------- ---------- ---------- ---------- ----------
bau-D17  ---------- ---------- ---------- ---------- ---------- ---------- ---------- ---------- ---------- ---------- ---------- ----------
nos-D18  GMIGSGNTLT INSGVINNQS GSITAQNSAS IYAAQSIDNR AGVIAANHDI TMNSQGLSND AVDRSGKMVA GQISSVQGLL DIDAGQGSLS NQGGQLLAGT DLTLRSGSLN NEQGRMSAQN
nos-D22  GMIGSGNTLT INSGVINNQS GSITAQNSAS IHAAQSIDNT AGAIAANHDI TMSSQGLSND AVDRSGKMVA GQISSVQGLL DIDAGQGSLS NQGGQLLAGT DLTLHSGSLN NEQGRMSAQN
 bau-D2  ---------- ---------- ---------- ---------- ---------- ---------- ---------- ---------- ---------- ---------- ---------- ----------
bay-D15  ---------- ---------- ---------- ---------- ---------- ---------- ---------- ---------- ---------- ---------- ---------- ----------

         2161                                                                                                                    2280
 bau-D1  ---------- ---------- ---------- ---------- ---------- ---------- ---------- ---------- ---------- ---------- ---------- ----------
 nos-D2  ---------- ---------- ---------- ---------- ---------- ---------- ---------- ---------- ---------- ---------- ---------- ----------
 bau-D3  ---------- ---------- ---------- ---------- ---------- ---------- ---------- ---------- ---------- ---------- ---------- ----------
 nos-D4  ---------- ---------- ---------- ---------- ---------- ---------- ---------- ---------- ---------- ---------- ---------- ----------
 bau-D7  ---------- ---------- ---------- ---------- ---------- ---------- ---------- ---------- ---------- ---------- ---------- ----------
 bau-D5  ---------- ---------- ---------- ---------- ---------- ---------- ---------- ---------- ---------- ---------- ---------- ----------
 nos-D8  ---------- ---------- ---------- ---------- ---------- ---------- ---------- ---------- ---------- ---------- ---------- ----------
 bau-D9  ---------- ---------- ---------- ---------- ---------- ---------- ---------- ---------- ---------- ---------- ---------- ----------
 bau-D6  ---------- ---------- ---------- ---------- ---------- ---------- ---------- ---------- ---------- ---------- ---------- ----------
nos-D12  ---------- ---------- ---------- ---------- ---------- ---------- ---------- ---------- ---------- ---------- ---------- ----------
bau-D10  KLSAQINQDI DNSAGQIVAN QAVQLNSQGL NNN-AGQVGS VAGHVILDAG SGDLSN-QSG KIISAQDLTL NA-------- ---------- ----QHVDNQ SGLISAQQQL VLNTQGLNNL
bau-D11  NLSAQINQDI DNNAGQIIAN QAVQLNSQGL NNN-AGQIGS VTGPVTLEAG SGDLIN-QSG KIISTQDLTL SA-------- ---------- ----QHVDNQ SGLISAQQ-L VLNTQGLSNL
bau-D14  NLSAQINQDI DNNAGQIIAN QAVQLNSQGL NNN-AGQIGS VTGPVTLEAG SGDLIN-QSG KIISTQDLTL SA-------- ---------- ----QHVDNQ SGLISAQQ-L VLNTQGLSNL
bau-D12  NLSVQINQDI DNNAGQIIAN QAVQLNSQGL NNN-AGQIGS VTGPVTLEAG SGDLIN-QSG KIISTQDLTL SA-------- ---------- ----QHVDNQ SGLISAQQ-L VLNTQGLSNL
bau-D20  KLSAQINQDI DNSAGQIVAN QAVQLNSQGL NNN-AGQVGS VAGHVILDAG SGDLSN-QSG KIISAQDLTL NA-------- ---------- ----QHVDNQ SGLISTQQQL VLNTQGLNNL
bau-D15  NLSAQINQDI DNNAGQIVAN HAVQLNSQGL NND-AGQIGS VTGPVMIEAG SGDLSN-QSG KIISTQDLTL SA-------- ---------- ----QHVDNQ SGLISAQQ-L VLNTQGLNNL
bau-D16  KLSAQINQDI DNSAGQIVAN QAVQLNSQGL NNN-AGQVGS VAGHVILDAG SGDLSN-QSG KIISAQDLTL NA-------- ---------- ----QHVDNQ SGLISAQQQL VLNTQGLNNL
bau-D21  KLSAQINQDI DNSAGQIVAN QAVQLNSQGL NNN-AGQVGS VAGHVILDAG SGDLSN-QSG KIISAQDLTL NA-------- ---------- ----QHVDNQ SGLISAQQQL VLNTQGLNNL
bau-D19  KLSAQINQDI DNSAGQIVAN QAVQLNSQGL NNN-AGQVGS VAGHVILDAG SGDLSN-QSG KIISAQDLTL NA-------- ---------- ----QHVDNQ SGLISAQQQL VLNTQGLNNL
bau-D13  ---------- ---------- ---------- ---------- ---------- ---------- ---------- ---------- ---------- ---------- ---------- ----------
cal-D23  ---------- ---------- ---------- ---------- ---------- ---------- ---------- ---------- ---------- ---------- ---------- ----------
pit-D20  ---------- ---------- ---------- ---------- ---------- ---------- ---------- ---------- ---------- ---------- ---------- ----------
 pit-D6  ---------- ---------- ---------- ---------- ---------- ---------- ---------- ---------- ---------- ---------- ---------- ----------
bau-D17  ---------- ---------- ---------- ---------- ---------- ---------- ---------- ---------- ---------- ---------- ---------- ----------
nos-D18  KLTITSIGLI NNHTGHMIAN HDVHLQSQGL NNSQSGQIAS LQGQVWLDAG TGLLNNNQSG LIQATMDSIL HAAGIDNSAG GQIKTQGALN IQSSQLVNNR AGQITANHDV QVSSQGLDNA
nos-D22  KLTITSIGLI NNRTGHMIAN HDVHLQSQGL NNSQSGQIAS LQGQVWLDAG TGLLNNSQSG LIQATMDSTL HAAGIDNSAD GQIKTQGALN IQSSQLVNNR AGQITANHDV QVSSQGLDNA
 bau-D2  ---------- ---------- ---------- ---------- ---------- ---------- ---------- ---------- ---------- ---------- ---------- ----------
bay-D15  ---------- ---------- ---------- ---------- ---------- ---------- ---------- ---------- ---------- ---------- ---------- ----------
```

```
         2281                                                                                                                    2400
 bau-D1  ---------- ---------- ---------- ---------- ---------- ---------- ---------- ---------- ---------- ---------- ---------- ----------
 nos-D2  ---------- ---------- ---------- ---------- ---------- ---------- ---------- ---------- ---------- ---------- ---------- ----------
 bau-D3  ---------- ---------- ---------- ---------- ---------- ---------- ---------- ---------- ---------- ---------- ---------- ----------
 nos-D4  ---------- ---------- ---------- ---------- ---------- ---------- ---------- ---------- ---------- ---------- ---------- ----------
 bau-D7  ---------- ---------- ---------- ---------- ---------- ---------- ---------- ---------- ---------- ---------- ---------- ----------
 bau-D5  ---------- ---------- ---------- ---------- ---------- ---------- ---------- ---------- ---------- ---------- ---------- ----------
 nos-D8  ---------- ---------- ---------- ---------- ---------- ---------- ---------- ---------- ---------- ---------- ---------- ----------
 bau-D9  ---------- ---------- ---------- ---------- ---------- ---------- ---------- ---------- ---------- ---------- ---------- ----------
 bau-D6  ---------- ---------- ---------- ---------- ---------- ---------- ---------- ---------- ---------- ---------- ---------- ----------
nos-D12  ---------- ---------- ---------- ---------- ---------- ---------- ---------- ---------- ---------- ---------- ---------- ----------
bau-D10  ---------- ---------- ---------- ---------- ---------- ---------- --------KG QIISGTN-LT FVGQDLNNQG GLLQT--NAD LDLNLSGVLD NSL----AGQ
bau-D11  ---------- ---------- ---------- ---------- ---------- ---------- --------KG QIISGTA-LT FVGQHLNNQG GLLQT--NSD LNLNLSGVLD NSL----GGQ
bau-D14  ---------- ---------- ---------- ---------- ---------- ---------- --------KG QIISGTA-LT FVGQHLNNQG GLLQT--NSD LNLNLSGVLD NSL----GGQ
bau-D12  ---------- ---------- ---------- ---------- ---------- ---------- --------KG QIISGTA-LT FVGQHLNNQG GLLQT--NSD LNLNLSGVLD NSL----GGQ
bau-D20  ---------- ---------- ---------- ---------- ---------- ---------- --------KG QIISGTN-LT FVGQDLNNQG GLLQT--NAD LDLNLSGVLD NSL----AGQ
bau-D15  ---------- ---------- ---------- ---------- ---------- ---------- --------KG QIISGTA-LT FVGKDLNNQG GLLQT--NSD LNLNLSGVLD NSL----GGQ
bau-D16  ---------- ---------- ---------- ---------- ---------- ---------- --------KG QIISGTN-LT FVGQDLNNQG GLLQT--NAD LDLNLSGVLD NSL----GGQ
bau-D21  ---------- ---------- ---------- ---------- ---------- ---------- --------KG QIISGTN-LT FVGQDLNNQG GLLQT--NAD LDLNLSGVLD NSL----AGQ
bau-D19  ---------- ---------- ---------- ---------- ---------- ---------- --------KG QIISGTN-LT FVGQDLNNQG GLLQT--NAD LDLNLSGVLD NSL----AGQ
bau-D13  ---------- ---------- ---------- ---------- ---------- ---------- ---------- ---------- ---------- ---------- ---------- ----------
cal-D23  ---------- ---------- ---------- ---------- ---------- ---------- ---------- ---------- ---------- ---------- ---------- ----------
pit-D20  ---------- ---------- ---------- ---------- ---------- ---------- ---------- ---------- ---------- ---------- ---------- ----------
 pit-D6  ---------- ---------- ---------- ---------- ---------- ---------- ---------- ---------- ---------- ---------- ---------- ----------
bau-D17  ---------- ---------- ---------- ---------- ---------- ---------- ---------- ---------- ---------- ---------- ---------- ----------
nos-D18  QQGLVGSVSG ALNIDAGQGL LNNQQGGQLL AATTADLKAA DIDNSNAGII NAHNDIQIQS LGEINNQNKG QIVSGAGDIS VIANQFGNQG GVIQTYPDHD IYLNVKGMLD NSAFNRTAGQ
nos-D22  QQGLVGSVSG ALNIDAGQGL LNNQQGGQLL AATTADLKAA DIDNSNAGII NAHNDIQIQS LGEINNQNKG QIVSGAGDIS VIANQFGNQG GVIQTYPDHD IHLNVKGILD NSAFNGTAGQ
 bau-D2  ---------- ---------- ---------- ---------- ---------- ---------- ---------- ---------- ---------- ---------- ---------- ----------
bay-D15  ---------- ---------- ---------- ---------- ---------- ---------- ---------- ---------- ---------- ---------- ---------- ----------

         2401                                                                                                                    2520
 bau-D1  ---------- ---------- ---------- ---------- ---------- ---------- ---------- ---------- ---------- ---------- ---------- ----------
 nos-D2  ---------- ---------- ---------- ---------- ---------- ---------- ---------- ---------- ---------- ---------- ---------- ----------
 bau-D3  ---------- ---------- ---------- ---------- ---------- ---------- ---------- ---------- ---------- ---------- ---------- ----------
 nos-D4  ---------- ---------- ---------- ---------- ---------- ---------- ---------- ---------- ---------- ---------- ---------- ----------
 bau-D7  ---------- ---------- ---------- ---------- ---------- ---------- ---------- ---------- ---------- ---------- ---------- ----------
 bau-D5  ---------- ---------- ---------- ---------- ---------- ---------- ---------- ---------- ---------- ---------- ---------- ----------
 nos-D8  ---------- ---------- ---------- ---------- ---------- ---------- ---------- ---------- ---------- ---------- ---------- ----------
 bau-D9  ---------- ---------- ---------- ---------- ---------- ---------- ---------- ---------- ---------- ---------- ---------- ----------
 bau-D6  ---------- ---------- ---------- ---------- ---------- ---------- ---------- ---------- ---------- ---------- ---------- ----------
nos-D12  ---------- ---------- ---------- ---------- ---------- ---------- ---------- ---------- ---------- ---------- ---------- ----------
bau-D10  LYSGGQTTIK AASVDNSAQG QINSQGDLNI TTAQDINNTE GVIVATKQMQ LKSQGLENTA ---------- GQIGTEQGDI LIQTGDLALN NGTGAIQSGK TLTLDVNSLT NS--------
bau-D11  LYSGGSTTVQ AASIDNSSQG QISAQGDLNL TTAQEINNAS GIIVASQQMQ LKSQGLNNDG ---------- GQIGTEQGAL LIETGDLALN NGTGAIQSGK SLSLDVNGLT NS--------
bau-D14  LYSGGSTTVQ AASIDNSSQG QISAQGDLNL TTAQEINNAS GIIVASQQMQ LKSQGLNNDG ---------- GQIGTEQGAL LIETGDLALN NGTGAIQSGK SLSLDVNGLT NS--------
bau-D12  LYSGGSTTVQ AASIDNSSQG QISAQGDLNL TTAQEINNAS GIIVASQQMQ LKSQGLNNDG ---------- GQIGTEQGDL LIETGDLALN NGTGAIQSGK SLSLDVNGLT NS--------
bau-D20  LYSGGQTTIK AASVDNSAQG QINSQGDLNI TTAQDINNTE GVIVATKQMQ LKSQGLENTA ---------- GQIGTEQGDI LIQTGDLALN NGTGAIQSGK TLTLDVNSLT NS--------
bau-D15  LYSGGSTKVQ AASVDNSSQG QISAQGDLNL TTAQGINNAG GIIVSSQQMQ LKSQGLNNDG ---------- GQIGTEQGDL LIETGDLALN NGTGAIQSGK SLSLDVKGLT NS--------
bau-D16  LYSGGQTTIK AASVDNSAQG QINSQGDLNI TTAQDINNTE GVIVATKQMQ LKSQGLENTA ---------- GQIGTEQGDI LIQTGDLALN NGTGAIQSGE SLSLDVNGLT NS--------
bau-D21  LYSGGQTTIK AASVDNSAQG QINSQGDLNI TTAQDINNTE GVIVATKQMQ LKSQGLENTA ---------- GQIGTEQGDI LIQTGDLALN NGTGAIQSGK TLTLDVNSLT NS--------
bau-D19  LYSGGQTTIK AASVDNSAQG QINSQGDLNI TTAQDINNTE GVIVATKQMQ LKSQGLENTA ---------- GQIGTEQGDL LIETGDLALN NGTGAIQSGK SLSLDVNGLT NS--------
bau-D13  ---------- ---------- ---------- ---------- ---------- ---------- ---------- ---------- ---------- ---------- ---------- ----------
cal-D23  ---------- ---------- ---------- ---------- ---------- ---------- ---------- ---------- ---------- ---------- ---------- ----------
pit-D20  ---------- ---------- ---------- ---------- ---------- ---------- ---------- ---------- ---------- ---------- ---------- ----------
 pit-D6  ---------- ---------- ---------- ---------- ---------- ---------- ---------- ---------- ---------- ---------- ---------- ----------
bau-D17  ---------- ---------- ---------- ---------- ---------- ---------- ---------- ---------- ---------- ---------- ---------- ----------
nos-D18  INSGHSLSIS SNSINNQF-G SMASQDTLSL TTAQRIDNTA GHIAAGNTVN IHSNGLINDA AVDTEGHLVA GQIGSVHDAL SIDAGQDSLS NRGGKLLAAT TVQLNALNID NSNAGKINAQ
nos-D22  INSGHSLSIS SSLINNQF-G SMASQDTLSV TTAQRIDNTA GHIAAGNTVN IHSNGLINDA AVDAEGHLVA GQIGSVHDAL SIDAGQDSLS NRGGKLLAAT TVQLNALNID NSNAGKINAQ
 bau-D2  ---------- ---------- ---------- ---------- ---------- ---------- ---------- ---------- ---------- ---------- ---------- ----------
bay-D15  ---------- ---------- ---------- ---------- ---------- ---------- ---------- ---------- ---------- ---------- ---------- ----------

         2521                                                                                                                    2640
 bau-D1  ---------- ---------- ---------- ---------- ---------- ---------- ---------- ---------- ---------- ---------- ---------- ----------
 nos-D2  ---------- ---------- ---------- ---------- ---------- ---------- ---------- ---------- ---------- ---------- ---------- ----------
 bau-D3  ---------- ---------- ---------- ---------- ---------- ---------- ---------- ---------- ---------- ---------- ---------- ----------
 nos-D4  ---------- ---------- ---------- ---------- ---------- ---------- ---------- ---------- ---------- ---------- ---------- ----------
 bau-D7  ---------- ---------- ---------- ---------- ---------- ---------- ---------- ---------- ---------- ---------- ---------- ----------
 bau-D5  ---------- ---------- ---------- ---------- ---------- ---------- ---------- ---------- ---------- ---------- ---------- ----------
 nos-D8  ---------- ---------- ---------- ---------- ---------- ---------- ---------- ---------- ---------- ---------- ---------- ----------
 bau-D9  ---------- ---------- ---------- ---------- ---------- ---------- ---------- ---------- ---------- ---------- ---------- ----------
 bau-D6  ---------- ---------- ---------- ---------- ---------- ---------- ---------- ---------- ---------- ---------- ---------- ----------
nos-D12  ---------- ---------- ---------- ---------- ---------- ---------- ---------- ---------- ---------- ---------- ---------- ----------
bau-D10  ---------- ---------- ---------- ---------- ---------- ---------- ---------- ---------- -GVISALDQL TLNSQGDVNN DNGQILSNQQ LLLTSQNLSN
bau-D11  ---------- ---------- ---------- ---------- ---------- ---------- ---------- ---------- -GVISALDQL TVNSQGDVNN DHGQILSNKQ LQLTGQNLSN
bau-D14  ---------- ---------- ---------- ---------- ---------- ---------- ---------- ---------- -GVISALDQL TVNSQGDVNN DHGQILSNKQ LQLTGQNLSN
bau-D12  ---------- ---------- ---------- ---------- ---------- ---------- ---------- ---------- -GVISALDQL TVNSQGDVNN DHGQILSNKQ LQLTGQNLSN
bau-D20  ---------- ---------- ---------- ---------- ---------- ---------- ---------- ---------- -GVISALDQL TLNSQGDVNN DNGQILSNQQ LLLTSQNLSN
bau-D15  ---------- ---------- ---------- ---------- ---------- ---------- ---------- ---------- -GVISALEQL TISSQGDVNN DHGQILSNKQ LQLTGQNLSN
bau-D16  ---------- ---------- ---------- ---------- ---------- ---------- ---------- ---------- -GVISALDQL TVNSQGDVNN DHGQILSNKQ LQLTGQNLSN
bau-D21  ---------- ---------- ---------- ---------- ---------- ---------- ---------- ---------- -GVISALDQL TLNSQGDVNN DNGQILSNQQ LLLTSQNLSN
bau-D19  ---------- ---------- ---------- ---------- ---------- ---------- ---------- ---------- -GVISALDQL TVNSQGDVNN DHGQILSNKQ LQLTGQNLSN
bau-D13  ---------- ---------- ---------- ---------- ---------- ---------- ---------- ---------- ---------- ---------- ---------- ----------
cal-D23  ---------- ---------- ---------- ---------- ---------- ---------- ---------- ---------- ---------- ---------- ---------- ----------
pit-D20  ---------- ---------- ---------- ---------- ---------- ---------- ---------- ---------- ---------- ---------- ---------- ----------
 pit-D6  ---------- ---------- ---------- ---------- ---------- ---------- ---------- ---------- ---------- ---------- ---------- ----------
bau-D17  ---------- ---------- ---------- ---------- ---------- ---------- ---------- ---------- ---------- ---------- ---------- ----------
nos-D18  NDITIQTQGT LGNQNHGQIV SATGNINATA NGLNNQQGLI ETYLNHDIDL TLKDTLDNSQ SGTISSGNRL NISSGSVDNH LGTLLAQEMA SIQTKQGINN TAGKITANQD IGINGTGLIN
nos-D22  NDITIQTQGT LGNQNHGQIV SATGNINATA NGLNNQQGLI ETYLNHDIDL TLKDTLDNSQ SGTISSGNRL NISSDSVDNH LGTLLAQEMA SIQTKQGINN TAGKITANQD IGINSTGLIN
 bau-D2  ---------- ---------- ---------- ---------- ---------- ---------- ---------- ---------- ---------- ---------- ---------- ----------
bay-D15  ---------- ---------- ---------- ---------- ---------- ---------- ---------- ---------- ---------- ---------- ---------- ----------

         2641                                                                                                                    2760
 bau-D1  ---------- ---------- ---------- ---------- ---------- ---------- ---------- ---------- ---------- ---------- ---------- ----------
 nos-D2  ---------- ---------- ---------- ---------- ---------- ---------- ---------- ---------- ---------- ---------- ---------- ----------
 bau-D3  ---------- ---------- ---------- ---------- ---------- ---------- ---------- ---------- ---------- ---------- ---------- ----------
 nos-D4  ---------- ---------- ---------- ---------- ---------- ---------- ---------- ---------- ---------- ---------- ---------- ----------
 bau-D7  ---------- ---------- ---------- ---------- ---------- ---------- ---------- ---------- ---------- ---------- ---------- ----------
 bau-D5  ---------- ---------- ---------- ---------- ---------- ---------- ---------- ---------- ---------- ---------- ---------- ----------
 nos-D8  ---------- ---------- ---------- ---------- ---------- ---------- ---------- ---------- ---------- ---------- ---------- ----------
 bau-D9  ---------- ---------- ---------- ---------- ---------- ---------- ---------- ---------- ---------- ---------- ---------- ----------
 bau-D6  ---------- ---------- ---------- ---------- ---------- ---------- ---------- ---------- ---------- ---------- ---------- ----------
nos-D12  ---------- ---------- ---------- ---------- ---------- ---------- ---------- ---------- ---------- ---------- ---------- ----------
bau-D10  QAGIIQSGEQ ANLDLTINGV LNNQTGQIHG GANTQIHANS INNSQQGQIS AQDALNIISA GLVDNQ-AGQ MIANQNVTLT SQGLNN-NQG QIGSIQGSLT VDAGNQALSN -QSGTLQAKS
bau-D11  QTGIIQSGEQ SNLILDINGI LNNQAGQIHG GANTQIDASS INNSQQGQIS AQDALNVISA GLVNNQ-AGT LVANKNVTLS SQGLNN-NQG QIGSVEGGLT IDTGNQALSN -QAGTLQAKT
bau-D14  QTGIIQSGEQ SNLILDINGI LNNQAGQIHG GANTQIDASS INNSQQGQIS AQDALNVISA GLVNNQ-AGT LVANKNVTLS SQGLNN-NQG QIGSVEGGLT IDTGNQALSN -QAGTLQAKT
bau-D12  QTGIIQSGEQ SNLILDINGI LNNQAGQIHG GANTQIDASS INNSQQGQIS AQDALNVISA GLVNNQ-AGT LVANKNVTLS SQGLNN-NQG QIGSVEGGLT IDTGNQALSN -QAGTLQAKT
bau-D20  QAGIIQSGEQ SNLNLDINGV LNNQAGQIHG GANTQIDANS INNSQQGQIS AQDALNIISA GLVNNQ-AGT LVANKNVTLS SQGLNN-NQG QIGSVEGGLT IDTGNQALSN -QAGTLQAKT
bau-D15  QTGIIQSGEQ ADLDLNINGA LNNQTGQIHS GASTQINASS INNSQQGQIS AQDALNIISA DLVDNQ-AGT LVANHEVTLN SQGLNN-NQG QIGSVEGGLA IDTGNQALSN -QAGTLQAKT
bau-D16  QTGIIQSGEQ SNLILDINGI LNNQAGQIHG GANTQIHANS INNSQQGQIS AQDALNIISA GLVDNQ-AGQ MIANQNVTLT SQGLNN-NQG QIGSIQGSLT VDAGNQALSN -QSGTLQAKS
bau-D21  QAGIIQSGEQ ANLDLTINGV LNNQTGQIHG GANTQIHANS INNSQQGQIS AQDALNIISA GLVDNQ-AGQ MIANQNVTLT SQGLNN-NQG QIGSIQGSLT VDAGNQALSN -QSGTLQAKS
bau-D19  QTGIIQSGEQ SNLILDINGI LNNQAGQIHG GANTQIHANS INNSQQGQIS AQDALNIISA GLVDNQ-AGQ MIANQNVTLT SQGLNN-NQG QIGSIQGSLT VDAGNQALSN -QSGTLQAKS
bau-D13  ---------- ---------- ---------- ---------- ---------- ---------- ---------- ---------- ---------- ---------- ---------- ----------
cal-D23  ---------- ---------- ---------- ---------- ---------- ---------- ---------- ---------- ---------- ---------- ---------- ----------
pit-D20  ---------- ---------- ---------- ---------- ---------- ---------- ---------- ---------- ---------- ---------- ---------- ----------
 pit-D6  ---------- ---------- ---------- ---------- ---------- ---------- ---------- ---------- ---------- ---------- ---------- ----------
bau-D17  ---------- ---------- ---------- ---------- ---------- ---------- ---------- ---------- ---------- ---------- ---------- ----------
```

```
 nos-D18  DSGLIGSASG QLQVDAGSGS LTNHAGQLLS AGNMDLHAAG IDNSGQGEIN TQAQLNINSD QVLSNSDAGQ IIAKQDLSIH SDGLDNYNAG RIGSQQGMLN LNIGTGLLNN SQQGLLQAAT
 nos-D22  DSGLIGSASG QLQVDAGLGS LTNHAGQLLS AGNMDLHAAG IDNSGQGEIN TQAQLNINSD QVLSNSDAGQ IIAKQDLSIH SDGLDNYNAG RIGSQQGMLN LNIGTGLLNN SQQGLLQAAT
  bau-D2  ---------- ---------- ---------- ---------- ---------- ---------- ---------- ---------- ---------- ---------- ---------- ----------
 bay-D15  ---------- ---------- ---------- ---------- ---------- ---------- ---------- ---------- ---------- ---------- ---------- ----------

          2761                                                                                                                    2880
  bau-D1  ---------- ---------- ----DNIVNQ LIVKNLLNNS NGKIQTNHNL EIQAGALKNN TGSI------ ---------- ---------- ---------- ---------- ----------
  nos-D2  ---------- ---------- ----DNIVNQ LIVKNLLNNS NGKIQTNHNL EIQAGALKNN TGSI------ ---------- ---------- ---------- ---------- ----------
  bau-D3  ---------- ---------- ----DNIVNQ LIVKNLLNNS NGKIQTNHNL EIQADALKNN AGSI------ ---------- ---------- ---------- ---------- ----------
  nos-D4  ---------- ---------- ----DNIVNQ LIVKNLLNNS NGKIQTNHNL EIQAGALKNN MGSI------ ---------- ---------- ---------- ---------- ----------
  bau-D7  ---------- ---------- ----DNIVNQ LIVKNLLNSS NGKIQTNHNL EIQADALKNN AGSI------ ---------- ---------- ---------- ---------- ----------
  bau-D5  ---------- ---------- ----DNIVNQ LIVKNLLNNS NGKIQTNHNL EIQADALKNN AGSI------ ---------- ---------- ---------- ---------- ----------
  nos-D8  ---------- ---------- ----DNIVNQ LIVKNLLNNS NGKIQTNHNL EIQAGALKNN TGSI------ ---------- ---------- ---------- ---------- ----------
  bau-D9  ---------- ---------- ----DNIVNQ LIVKNLLNNS NGKIQTNHNL EIQADALKNN AGSI------ ---------- ---------- ---------- ---------- ----------
  bau-D6  ---------- ---------- ----DNIVNQ LIVKNLLNNS NGKIQTNHNL EIQADALKNN AGSI------ ---------- ---------- ---------- ---------- ----------
 nos-D12  ---------- ---------- ----DNIVNQ LIVKNLLNNS NGKIQTNHNL EIQAGVLKNN TGSI------ ---------- ---------- ---------- ---------- ----------
 bau-D10  DVSVKALSID NT-------V GQINTQGNIN LLSQQSINNT QGVIVGDQSI NINSQGLNNN QGQI------ ---------- ---------- ---------- ---------- ----------
 bau-D11  DVAVKALSVD NT-------A GQINTPGGID LVSQQNINNT QGSIVGDQRL NLKAQGLNNN QGQI------ ---------- ---------- ---------- ---------- ----------
 bau-D14  DVAVKALSVD NT-------A GQINTPGGID LVSQQNINNT QGSIVGDQRL NLKAQGLNNN QGQI------ ---------- ---------- ---------- ---------- ----------
 bau-D12  DVAVKALSVD NT-------A GQINTPGGID LVSQQNINNT QGSIVGDQRL NLKAQGLNNN QGQI------ ---------- ---------- ---------- ---------- ----------
 bau-D20  DVAVKALSVD NT-------A GQINTPGGID LVSQQNINNT QGSIVGDQRL NLKAQGLNNN QGQI------ ---------- ---------- ---------- ---------- ----------
 bau-D15  DIAVKALSVD NS-------A GQINTQGGID LVSQQNINNT QGSIVGDQRL NINSQGLNNN QGQI------ ---------- ---------- ---------- ---------- ----------
 bau-D16  DVSVKALSID NT-------A GQINTQGNIN LLSQQSINNT QGVIVGDQSI NINSQGLNNN QGQI------ ---------- ---------- ---------- ---------- ----------
 bau-D21  DVSVKALSID NT-------A GQINTQGNIN LLSQQSINNT QGVIVGDQSI NINSQGLNNN QGQI------ ---------- ---------- ---------- ---------- ----------
 bau-D19  DVSVKALSID NT-------A GQINTQGNIN LLSQQSINNT QGVIVGDQSI NINSQGLNNN QGQI------ ---------- ---------- ---------- ---------- ----------
 bau-D13  --------VD NT-------A GHFGAQGKID LTSQQTINNT QGSIVADQNL NIHSQGLINN QGKL------ ---------- ---------- ---------- ---------- ----------
 cal-D23  --------VD NT-------A GHFGAQGKID LNSQQTINNT QGSIVADQNL NMHSQGLINN QGKL------ ---------- ---------- ---------- ---------- ----------
 pit-D20  --------VD NT-------A GHFSAQGKID LTSQKNINNT QGSIVADQNL NIHSQGLINN QGKL------ ---------- ---------- ---------- ---------- ----------
  pit-D6  --------VE NT-------A GHISAQGKID LNSQKNINNT QGSIVADQNL NIHSQGLINN QGKL------ ---------- ---------- ---------- ---------- ----------
 bau-D17  --------VD NT-------A GHFSAQGKID LTSQQTINNT QGSIIADQNL NIHSQGLINN QGKL------ ---------- ---------- ---------- ---------- ----------
 nos-D18  DLNIESGSID NSHTLSAAQS NGIKAGGKLQ LISQQSLNNQ QGAIVAGKTL SIHSTEVNNN QGLMNSLDDL GIVTSGALTN QHGQLQSATN LSIESESLNN DHGDLSAQNT LAVSINQEIN
 nos-D22  DLNIESGSID NSHTLSAAQS NGIKAGGKLQ LISQQSLNNQ QGAIVAGKTL SIHSTEVNND QGLMNSLDDL GIVTSGALTN QHGQLQSATN LSIESDSLNN DHGDLSAQNT LAVSINQEIN
  bau-D2  ---------- ---------- ----EQNQLD LNVEQNLDNQ TGQIYAGSQA NIHTTELNNQ QGTI------ ---------- ---------- ---------- ---------- ----------
 bay-D15  ---------- ---------- ---------- ---------- ---------- ---------- -GTV------ ---------- ---------- ---------- ---------- ----------

          2881                                                                                                                    3000
  bau-D1  ---------- ---------- ---------- -------VTA GTAGLNLNTA SRLDNQSGNI HSSGDLNIKA QDILND-QGQ ILAAKNAQFN SQNTLSNQ-- ---------- ----------
  nos-D2  ---------- ---------- ---------- -------VTA GTAGLNLNTA SRLDNQSGNI HSSGDLNIKA QDILND-QGQ ILAAKNAQFN SQNTLSNQ-- ---------- ----------
  bau-D3  ---------- ---------- ---------- -------VTA GAAGLNLNTT SRLDNQSGNI RSSGDLNVNA QDILND-QGQ ILAAKNAQLN SQNTLSNQ-- ---------- ----------
  nos-D4  ---------- ---------- ---------- -------VTA GTAGLNLNTA SRLDNQSGNI HSSGDLNIKA QDILND-QGQ ILAAKNAQFN SQDTLSNQ-- ---------- ----------
  bau-D7  ---------- ---------- ---------- -------VTA GTAGLNLNTT SRLDNQSGNI RSSGDLNVNA QDILND-QGQ ILAAKNAQLN SQNTLSNQ-- ---------- ----------
  bau-D5  ---------- ---------- ---------- -------VTA GAAGLNLNTT SRLDNQSGNI RSSGDLNVNA QDILND-QGQ ILAAKNAQLN SQNTLSNQ-- ---------- ----------
  nos-D8  ---------- ---------- ---------- -------VTA GTAGLNLNTA SRLDNQSGNI HSSGDLNIKA QDILND-QGQ ILAAKNAQFN SQNTLSNQ-- ---------- ----------
  bau-D9  ---------- ---------- ---------- -------VTA GAAGLNLNTT SRLDNQSGNI RSSGDLNVNA QDILND-QGQ ILAAKNAQLN SQNTLSNQ-- ---------- ----------
  bau-D6  ---------- ---------- ---------- -------VTA GAAGLNLNTT SRLDNQSGNI RSSGDLNVNA QDILND-QGQ ILAAKNAQLN SQNTLSNQ-- ---------- ----------
 nos-D12  ---------- ---------- ---------- -------VTA GTAGLNLNTA SRLDNQSGNI RSSGDLNVNA QDILND-QGQ ILAAKNAQLN SQNTLSNQ-- ---------- ----------
 bau-D10  ---------- ---------- ---------- -------GTQ GDLTVQAGTQ V-LQNQTGLL QAGQNLNISA AAIDNLLSGQ INAQGSAILQ STGLVNNE-- ---------- ----------
 bau-D11  ---------- ---------- ---------- -------GTQ GDLNLSVGSG S-LQNQTGLL QAGQNIIVDA AAIDNSSTGK INAQGNVTLQ STGLINNE-- ---------- ----------
 bau-D14  ---------- ---------- ---------- -------GTQ GDLNLSVGSG S-LQNQTGLL QAGQNIIVDA AAIDNSSTGK INAQGNVTLQ STGLINNE-- ---------- ----------
 bau-D12  ---------- ---------- ---------- -------GTQ GDLNLSVGSG S-LQNQTGLL QAGQNIIVDA AAIDNSSTGK INAQGNVTLQ STGLINNE-- ---------- ----------
 bau-D20  ---------- ---------- ---------- -------GTQ GDLNLSVGSG S-LQNQTGLL QAGQNIIVDA AAIDNSSTGK INTQGNVTLQ STGLINNE-- ---------- ----------
 bau-D15  ---------- ---------- ---------- -------GTQ GDLTVQAGTQ V-LQNQTGLL QAGQNLNISA AAIDNSLSGK INAQGSVALQ STGLINNE-- ---------- ----------
 bau-D16  ---------- ---------- ---------- -------GTQ GDLTVQAGTQ V-LQNQTGLL QAGQNLNISA AAIDNLLSGQ INAQGSAILQ STGLVNNE-- ---------- ----------
 bau-D21  ---------- ---------- ---------- -------GTQ GDLTVQAGTQ V-LQNQTGLL QAGQNLNISA AAIDNLLSGQ INAQGSAILQ STGLVNNE-- ---------- ----------
 bau-D19  ---------- ---------- ---------- -------GTQ GDLTVQAGTQ V-LQNQTGLL QAGQNLNISA AAIDNLLSGQ INAQGSAILQ STGLVNNE-- ---------- ----------
 bau-D13  ---------- ---------- ---------- -------ATQ GNLTVQAGTQ A-LKNQAGII QSGQDMSVIA ASIDNSLSGQ INAQGSANLQ STGLLNNE-- ---------- ----------
 cal-D23  ---------- ---------- ---------- -------ATQ GNLTLQAGTQ A-LQNQAGII QSGQDMSMIA GSIDNSLSGQ INAQSRATLQ STGLLNNE-- ---------- ----------
 pit-D20  ---------- ---------- ---------- -------ATQ GNLTVQAGTQ A-LQNQAGII QSGQDMSVIA ASIDNSLSGQ INAQGSATLQ STGLLNNE-- ---------- ----------
  pit-D6  ---------- ---------- ---------- -------ATQ GNLTVQAGTQ A-LKNQAGII QSGQDMSVIA ASIDNSLSGQ INAQGSATLQ STGLLNNE-- ---------- ----------
 bau-D17  ---------- ---------- ---------- -------ATQ GNLTVQAGTQ A-LQNQAGMI QSGQDLSVMA ASIDNSLSGQ INAQGSANLQ STGLLNNE-- ---------- ----------
 nos-D18  NVGGHIAAFN DVNLHSNGLN NNAVSNAAGS MDVSQIGSVY SNLNIDAGRG A-LTNQGSSL QAGRSIQLSA DGIDSSNGGY IHAAQNIELN SQHRLNNDYG QIVADSGSIY SQSSDLSNLG
 nos-D22  NVGGHIAAFN DVNLHSNGLN NNAVSNAAGS MDVSQIGSVY GNLNIDAGRG A-LTNQGSSL QAGRSIQLSA DGIDSSNGGY IHAVQNIELN SQHRLNNDYG QIVADSGSIY SQSSDLSNLG
  bau-D2  ---------- ---------- ---------- ---------Y SQNQLDLNAS Q-LNNQQGQI YSG------- ---------- ----GQEQLK VQGDIQNQ-- ---------- ----------
 bay-D15  ---------- ---------- ---------- ---------Q GNLHIDAGSQ L-LNNQQGRI QSG------- ---------- ---------- ---------- ---------- ----------

          3001                                                                                                                    3120
  bau-D1  ---------- ---------- ---------- ---------- ---------- ---------- ---------A GLIAAQQQLM IQSAALNNQA GQIGSVDAGV NIQTTQQALN NQSGKIQANQ
  nos-D2  ---------- ---------- ---------- ---------- ---------- ---------- ---------A GLIAAQQQLM IQSAALNNQA GQIGSVDAGV NIQTTQQALN NQSGKIQANQ
  bau-D3  ---------- ---------- ---------- ---------- ---------- ---------- ---------A GLIAAQQQLM IQSAALNNQA GQIGSVDAGV NIQTTQQALN NQSGKIQANQ
  nos-D4  ---------- ---------- ---------- ---------- ---------- ---------- ---------A GLIAAQQQLM IQSAALNNQA GQIGSVDAGV NIQTTQQALN NQSGKIQANQ
  bau-D7  ---------- ---------- ---------- ---------- ---------- ---------- ---------A GLIAAQQQLM IQSAALNNQA GQIGSVDAGV NIQTTQQALN NQSGKIQANQ
  bau-D5  ---------- ---------- ---------- ---------- ---------- ---------- ---------A GLIAAQQQIM IQSAALNNQA GQIGSVDAGV NIQTTQQALN NQSGKIQANQ
  nos-D8  ---------- ---------- ---------- ---------- ---------- ---------- ---------A GLIAAQQQLM IQSAALNNQA GQIGSVDAGV NIQTTQQALN NQSGKIQANQ
  bau-D9  ---------- ---------- ---------- ---------- ---------- ---------- ---------A GLIAAQQQLM IQSAALNNQA GQIGSVDAGV NIQTTQQALN NQSGKIQANQ
  bau-D6  ---------- ---------- ---------- ---------- ---------- ---------- ---------A GLIAAQQQLM IQSAALNNQA GQIGSVDAGV NIQTTQQALN NQSGKIQANQ
 nos-D12  ---------- ---------- ---------- ---------- ---------- ---------- ---------A GLIAAQQQLM IQSAALNNQA GQIGSVDAGV NIQTTQQALN NQSGKIQANQ
 bau-D10  ---------- ---------- ---------- ---------- ---------- ---------- ---------T GTITANQDIT LTSQGLNNTQ GQIGSVVGSL NLDSGQQILN NQSGSLLSGH
 bau-D11  ---------- ---------- ---------- ---------- ---------- ---------- ---------T GVIAANQDVT LTGQGLNNTQ GQIGSVAGSL NLDSGDQILN NQSGSLLSNN
 bau-D14  ---------- ---------- ---------- ---------- ---------- ---------- ---------T GVIAANQDVT LTGQGLNNTQ GQIGSVAGSL NLDSGDQILN NQSGSLLSNN
 bau-D12  ---------- ---------- ---------- ---------- ---------- ---------- ---------T GVIAANQDVT LTGQGLNNTQ GQIGSVAGSL NLDSGDQILN NQSGSLLSNN
 bau-D20  ---------- ---------- ---------- ---------- ---------- ---------- ---------T GVIAANQDVT LTGQGLNNTQ GQIGSVAGSL NLDSGDQILN NQSGSLLSNN
 bau-D15  ---------- ---------- ---------- ---------- ---------- ---------- ---------T GVIAANQDVM LTSQGLNNTQ GQIGSVTGSL NLDSGDQILN NQSGSLLSNN
 bau-D16  ---------- ---------- ---------- ---------- ---------- ---------- ---------T GTITANQDIT LTSQGLNNTQ GQIGSVVGSL NLDSGQQILN NQSGSLLSGH
 bau-D21  ---------- ---------- ---------- ---------- ---------- ---------- ---------T GTITANQDIT LTSQGLNNTQ GQIGSVVGSL NLDSGQQILN NQSGSLLSGH
 bau-D19  ---------- ---------- ---------- ---------- ---------- ---------- ---------T GTITANQDIT LTSQGLNNTQ GQIGSVVGSL NLDSGQQILN NQSGSLLSGH
 bau-D13  ---------- ---------- ---------- ---------- ---------- ---------- ---------T GVLAAGQDLT LNGNGLNNAN GKIGSVNGAL NINVGSSLLN NQSGSLQSSG
 cal-D23  ---------- ---------- ---------- ---------- ---------- ---------- ---------T GVLAAGQDLT LNSNGLNNTK GKIGSVNGAL NINAGSSLLN NQSGSLQSAG
 pit-D20  ---------- ---------- ---------- ---------- ---------- ---------- ---------T GVLAAGQDLT LNGNGLNNAN GKIGSVNGAL NINVGSSLLN NQSGSLQSSG
  pit-D6  ---------- ---------- ---------- ---------- ---------- ---------- ---------T GVLAAGQDLT LNGNGLNNAN GKIGSVNGAL NINAGSSLLN NQSGLLQSSG
 bau-D17  ---------- ---------- ---------- ---------- ---------- ---------- ---------T GVLAAGQDLT LNSNGLNNTK GKIGSVSGAL NINAGSSLLN NQSGSLQSSG
 nos-D18  GIILTQGLNN SVSPDIVLKQ SGILDNRQNG QISSSNQLNI SSGSIQNDHG DIHSKNTATL NTVQKIDNTA GKIAAINNVT VNSNGLINDT GHIGSVNGDL NVDAGSGVLS NQRGDLQSGQ
 nos-D22  GIILTQGLNN SVSPDIVLKQ SGILDNRQNG QISSSNQLNI SSGSIQNDQG DIHSKNTATL NTVQKIDNTA GKIAAINNVT VNSNGLINDT GHIGSVNGDL NVDAGSGVLS NQRGDLQSGH
  bau-D2  ---------- ---------- ---------- ---------- ---------- ---------- ---------K GVLAAGQNLN INSAGLDNTA GTIRSENADI TLNAQGQLIN AQ-GDIYAGH
 bay-D15  ---------- ---------- ---------- ---------- ---------- ---------- ---------- ------QDLN LNATGIDNT- ---------- ---------- ----------

          3121                                                                                                                    3240
  bau-D1  AINLDVQGLD NSLQGLISST KGDQSKIQID THQQSLNNQN GQINSGNTLQ ISTNSLNNQQ -GLITAQGDL GINAVQLIDN RQTYLNATLP ELAQGIQSLG QVLLQT-SEL NNEQGQVIAG
  nos-D2  AINLDVQGLD NSLQGLISST KGDQSKIQID THQQSLNNQN GQINSGNTLQ ISTNSLNNQQ -GLITAQGDL GINAVQLIDN RQTYLNATLP ELAQGIQSLG QVLLQT-SEL NNEQGRVIAG
  bau-D3  AINLDVQGLD NSLQGLISST KGDQSKIQID THQQNLNNQN GQINSGNTLQ ISTNGLNNQQ -GLITAQGDL GINAVQLIDN RQTYLNATLP ELAQGIQSLG QVVLQT-SEL NNEQGQVIAG
  nos-D4  AINLDVQGLD NSLQGLISST KGDQSKIQID THQQSLNNQN GQINSGNTLQ ISTNSLNNQQ -GLITAQGDL GINAVQLIDN RQTYLNATLP ELAQGIQSLG QVLLQT-SEL NNEQGQVIAG
  bau-D7  AINLDVQGLD NSLQGLISST KGDQSKIQID THQQNLNNQN GQINSGNTLQ ISTNGLNNQQ -GLITAQGDL GINAVQLIDN RQTYLNATLP ELAQGIQSLG QVVLQT-SEL NNEQGQVIAG
  bau-D5  AINLDVQGLD NSLQGLISST KGDQSKIQID THQQSLNNQN GQINSGNTLQ ISTNGLNNQQ -GLITAQGDL GINAVQLIDN RQTYLNATLP ELAQGIQSLG QVVLQT-SEL NNEQGQVIAG
  nos-D8  AINLDVQGLD NSLQGLISST KGDQSKIQID THQQSLNNQN GQINSGNTLQ ISTNSLNNQQ -GLITAQGDL GINAVQLIDN RQTYLNATLP ELAQGIQSLG QVLLQT-SEL NNEQGQVIAG
  bau-D9  AINLDVQGLD NSLQGLISST KGDQSKIQID THQQNLNNQN GQINSGNTLQ ISTNGLNNQQ -GLITAQGDL GINAVQLIDN RQTYLNATLP ELAQGIQSLG QVVLQT-SEL NNEQGQVIAG
  bau-D6  AINLDVQGLD NSLQGLISST KGDQSKIQID THQQNLNNQN GQINSGNTLQ ISTNGLNNQQ -GLITAQGDL GINAVQLIDN RQTYLNATLP ELAQGIQSLG QVVLQT-SEL NNEQGQVIAG
 nos-D12  AINLDVQGLD NSLQGLISST KGDQSKIQID THQQNLNNQN GQINSGNTLQ ISTNGLNNQQ -GLITAQGDL GINAVQLIDN RQTYLNATLP ELAQGIQSLG QVVLQT-SEL NNEQGQVIAG
 bau-D10  QINIHAAGLN NTQGQIVA-- ---QQVLDID AELQALNNQQ GLISS-DTVN ITSGLLNNDQ -GLIQSNSAM ------MIDT QGQSLINTNS GTQGGLLSQG NLSLKNVGLL DNKLGYLASG
 bau-D11  QISIHATGLN NSQGQIVA-- ---QQLLDID AELQALNNQN GLMSS-DTVN ITSGLLNNDQ -GLIQSNSAM ------TIDT QGHNLINTNS GTQGGLLSQG NLSLKNVGLL DNKLGYLASG
 bau-D14  QISIHATGLN NSQGQIVA-- ---QQLLDID AELQALNNQN GLMSS-DTVN ITSGLLNNDQ -GLIQSNSAM ------TIDT QGHNLINTNS GTQGGLLSQG NLSLKNVGLL DNKLGYLASG
 bau-D12  QISIHATGLN NSQGQIVA-- ---QQLLDID AELQALNNQN GLMSS-DTVN ITSGLLNNDQ -GLIQSNSAM ------TIDT QGQNLINTNS GTQGGLLSQG NLSLKNVGLL DNKLGYLASG
 bau-D20  QISIHATGLN NSQGQIVA-- ---QQLLDID AELQALNNQN GLMSS-DTVN IMSGLLNNDQ -GLIQSNSAM ------TIDT QGQNLINTNS GTQGGLLSQG NLSLKNVGLL DNKLGYLASG
 bau-D15  QISIHATGLN NSQGQIVA-- ---QQLLDID AELQAINNQN GLMSS-DTVN VTSGAFNNDQ -GLVQAKTSL ------VLDT NGQNLVNSNS GTQGGLLSQG NLTLKNLGTL DNTKGYVASG
 bau-D16  QINIHAAGLN NTQGQIVA-- ---QQVLDID AELQALNNQQ GLISS-DTVN ITSGLLNNDQ -GLIQSNSAM ------MIDT QGQSLINTNS GTQGGLLSQG NLSLKNVGLL DNKLGYLASG
 bau-D21  QINIHAAGLN NTQGQIVA-- ---QQVLDID AELQALNNQQ GLISS-DTVN ITSGLLNNDQ -GLIQSNSAM ------TIDT QGQNLINTNS GTQGGLLSQG NLSLKNVGLL DNKLGYLASG
 bau-D19  QINIHAAGLN NTQGQIVA-- ---QQVLDID AELQALNNQQ GLISS-DTVN ITSGLLNNDQ -GLIQSNSAM ------MIDT QGQSLINTNS GTQGGLLSQG NLSLKNVGLL DNKLGYLASG
```

```
bau-D13  VIDLKASGVN NQLGKITS-- ---LKAINID SQQQAFTNQQ GTISS-DLVS ISSGLFNNDQ -GLVQAKTSL ------VLDT NGQNLVNTNS GTQGGLLSQG NLTLKNLIAL DNTQGYIASG
cal-D23  LIDLKAGGVN NQLGKITS-- ---LKAINID SQQQAFTNQQ GTISS-DLVS ISSGLFNNDQ -GLVQAKTSL ------VLDT NGQNLVNTNS GTQGGLLSQG NLTLKNLSAL DNTQGYIASG
pit-D20  VIDLKAGGVN NKLGKITS-- ---LTAINID SQKQAFTNQQ GTISS-DQIS ILSGPFNNDQ -GLVQAKTSL ------VLDT NGQNLVNTNS GTQGGLLSQG NLTLKNLSAL DNTQGYIASG
 pit-D6  VIDLKAGGVN NKLGKITS-- ---LTAINID SQQQAFTNQQ GTISS-DQIS ILSGPFNNDQ -GLVQAKTSL ------VLDT NGQNLVNTNS GTQGGLLSQG NLTLKNLSAL DNTQGYIASG
bau-D17  VIDVQAGGVN NQLGKITS-- ---LKAINID SQRQAFNNQQ GTISS-DLVS ISSGLFNNDQ -GLVQAKTSL ------VLDT NGQNLVNTNS GIQGGLLSQG NLTLKNLSAL DNTQGYIASG
nos-D18  NASIAATGID NQTGHIFA-- ---NQALSID SRGQALQNQQ GQING-TGVQ LSSGALDNGQ SGLIQSGALL ------SINT HGQALNNQNS GDTGGILSRG DLTLNQISQL DNTSGYIASG
nos-D22  NASIAATGID NQTGHIFA-- ---NQALSID SRGQALQNQQ GQISG-TGVQ LSSGALDNGQ SGLIQSGALL ------SINT HGQALNNQNS GDTGGILSRG DLTLNQISQL DNTSGYIASG
 bau-D2  NASLNSVGLD NSSGQIAA-- ---QNQLTID TQKQQLSNQN AKIIA-RAVD LKTGKLDNQT -GLIQAEQSV ------KIDT QQNALLNNNS GNNAGILSQG GLDIANVSQL ENSNGYIAAI
bay-D15  ---------- --QGQISS-- ---KNQLQLD SREQLLNNRQ GTLSA-ENLT IDSGELNNDQ -GLIQAQQSI ------QIDT HQHNLSNTNS GAQGGILSQG NIQLNNIQTL DNQQGYLASA

         3241                                                                                                                  3360
 bau-D1  NGLTIQAPKV NNSNAGLLAS GQDLLIDSVG QA--GTINNQ KGKISANQNI SLNTGLMS-- GSQLDNSQQG FISAAKQVKI ISQNIDNSNN DQN------- QGIQAGQIEI AASTLNNSAG
 nos-D2  NGLTIQAPKV NNSNAGLLAS GQDLLIDSVG QA--GTINNQ KGKISANQNI SLNTGLIS-- GSQLDNSQQG LISAAKQVKI VSQNIDNSNN DQN------- QGIQAGQIEI AASTLNNSSG
 bau-D3  NGLTIQAPKV NNSNAGLLAS GRDLLIDSVG QA--GTINNQ KGKISANQNI SLNTGLMS-- GSQLDNSQQG LISAAKQVKI ISQNIDNSNN DQN------- QGIQAGQIEI AASTLNNSAG
 nos-D4  NGLTIQAPKV NNSNAGLLAS GQDLLIDSVG QA--GTINNQ KGKISANRNI SLNTGLMT-- GSQLDNSQQG LISAAKQVKI VSQNIDNSNN DQN------- QGIQAGQIEI AASTLNNSAG
 bau-D7  NGLTIQAPKV NNSNAGLLAS GRDLLIDSVG QA--GTINNQ KGKISANQNI SLNTGLMS-- GSQLDNSQQG LISAAKQVKI ISQNIDNSNN DQN------- QGIQAGQIEI AASTLNNSAG
 bau-D5  NGLTIQAPKV NNSNAGLLAS GRDLLIDSVG QA--GTINNQ KGKISANQNI SLNTGLMS-- GSQLDNSQQG LISAAKQVKI ISQNIDNSNN DQN------- QGIQAGQIEI AASTLNNSAG
 nos-D8  NGLTIQAPKV NNSNAGLLAS GQDLLIDSVG QA--GTINNQ KGKISANQNI SLNTGLIS-- GSQLDNSQQG LISAAKQVKI VSQNIDNSNN DQN------- QGIQAGQIEI AASTLNNSAG
 bau-D9  NGLTIQAPKV NNSNAGLLAS GRDLLIDSVG QA--GTINNQ KGKISANQNI SLNTGLMS-- GSQLDNSQQG FISAAKQVKI ISQNIDNSNN DQN------- QGIQAGQIEI AASTLNNSAG
 bau-D6  NGLTIQAPKV NNSNAGLLAS GRDLLIDSVG QA--GTINNQ KGKISANQNI SLNTGLMS-- GSQLDNSQQG LISAAKQVKI ISQNIDNSNN DQN------- QGIQAGQIEI AASTLNNSAG
nos-D12  NGLTIQAPKV NNSNAGLLAS GRDLLIDSVG QA--GTINNQ KGKISANQNI SLNTGLMS-- GSQLDNSQQG LISAAKQVKI ISQNIDNSNN DQN------- QGIQAGQIEI AASTLNNSAG
bau-D10  QNLDISANQV QNSGGTLLAA -QNLKLQGIG QN--QLLNNQ SGQILSMGDM QLSVEHINNQ GKLASTDADS HIMATGQLDI QTQQLDNQNT -LVADT---V QGIDAGNLNL NAAIVNNQSG
bau-D11  QNLGLSANQV QNSGGTLLAA -QNLKLQGIG QN--QLLNNQ SGQILSMGDM QLSVEHINNQ GKLASTDADS HIMATGQLDV QTQQLDNQNT -LVADT---V QGIDAGNLNL NAAIVNNQSG
bau-D14  QNLGLSANQV QNSGGTLLAA -QNLKLQGIG QN--QLLNNQ SGQILSMGDM QLSVEHINNQ GKLASTDADS HIMATGQLDI QTQQLDNQNT -LVADT---V QGIDAGNLNL NAAIVNNQSG
bau-D12  QNLGLSANQV QNGGGTLLAA -QNLKLQGIG QN--QLLNNQ SGQILSMGDM QLSVEHINNQ GKLASTDADS HIMATGQLDI QTQQLDNQNT -LVADT---V QGIDAGNLNL NAAIVNNQSG
bau-D20  QNLGLSANQV QNGGGTLLAA -QNLKLQGIG QN--QLLNNQ SGQILSMGDM QLSVEHINNQ GKLASTDADS HIMATGQLDI QTQQLDNQNT -LVADT---V QGIDAGNLNL NAAIVNNQSG
bau-D15  QNLDISLNQV QNSGGTLLAV -QNLKLQGTG QN--QLLNNQ SGQILSMGDM QLSIEHINNQ GKLTSTDADS HIMAAGQLDI QTQQLDNQNT KALTDTDTSV QGIDAGNLNL DTSIVNNQSA
bau-D16  QNLGLSANQV QNSGGTLLAA -QNLKLQGIG QN--QLLNNQ SGQILSMGDM QLSVEHINNQ GKLASTDADS HIMATGQLDI QTQQLDNQNT -LVADT---V QGIDAGNLNL NAAIVNNQSG
bau-D21  QNLGLSANQV QNGGGTLLAA -QNLKLQGIG QN--QLLNNQ SGQILSMGDM QLSVEHINNQ GKLASTDADS HIMTTGQLDI QTQQLDNQNT -LVADT---V QGIDAGNLNL NAAIVNNQSG
bau-D19  QNLGLSANQV QNSGGTLLAA -QNLKLQGIG QN--QLLNNQ SGQILSMGDM QLSVEHINNQ GKLASTDADS HIMATGQLDI QTQQLDNQNT -LVADT---V QGIDAGNLNL NAAIVNNQSG
bau-D13  QNLDLSANQV INNKGTLLAT -QNLKLQGTG QN--QLLNNQ SGQILSMGDM QLTIEHVNNQ GKLVATDPDS HIMATGQLDL VTQQLDNQNT LTTDST--SV QGIDAGNLNL SAQTLNNKSG
cal-D23  QNLDLSANQV INNKGTLLAT -QNLKLQGTG QK--QLLNNQ SGQILSMGDM QLTIEQVNNQ GKLVATDLDS HIMAAGQLDL VAQHLDNQNT LTTDST--SV QGIDAGNLNL STQTLNNKSG
pit-D20  QNLDLSANQV INNKGTLLAT -QNLKLQGTG QK--QLLNNQ SGQILSMGDM QLTIEQINNQ GKLVATDPDS HIMATGQLDL VTQQLDNQNT FT-DST--SV QGIDAGNLNL SAQTLNNKSG
 pit-D6  QNLDLSANQV INNKGTLLAT -QNLKLQGTG QK--QLLNNQ SGQILSMGDM QLTIEQVNNQ GKLVATDPDS HIMATGQLDL VTQQLDNQNT FT-DST--SV QGIDAGNLNL SAQTLNNKSG
bau-D17  QNLDLSANQV INNKGTLLAT -QNLKLQGTG QK--QLLNNQ SGQILSMGDM QLTIEQINNQ GKLVATDPDS HIMATGQLDL VTQQLDNQNT FT-DST--SV QGIDAGNLNL SAQTLNNKSG
nos-D18  NDLNGNVDQV LNSHGQLIAA -HDLNLQSNG ASGSMLLNDQ SGQVNAGHDI SVHFDSINNQ G--------G HLVAANQLNL TSNVLDNQNT NNNLGT---- QGIDATTVNL NVNSLNNQSG
nos-D22  NDLNGNVDQV LNSHGQLIAA -HDLNLQSNG ASGSMLLNDQ SGQVNAGHDI SVHLDSINNQ G--------G HLVAANQLNL TSNVLDNQNT NNNLGT---- QGIDATTFNL NVNSLNNQSG
 bau-D2  GSANLTAQNI NNNSGQINSQ -ADLTIQQQL AG--GRIDNV AGQIQAQKNI SLNADTINNA GTV------S HIIAGEKLTA NASKVMNEQT KDSN----LL GGLHAKNIEI NAGELDNQSG
bay-D15  QQLDLQANQV FNQNGTLLAA -KQLNLQGTG QN--QLLNNQ SGQMLSMGGM NLNIDQINNK GKANTADADS HIMAAGELNI TTQQLDNQNT LNTDPANTSI QGIDANTLNV TSKVLNNQSG

         3361                                                                                                                  3480
 bau-D1  RISAEQQLNL NILDNLNNTK GLISSLDQLT IQGQQDNNRL IVNNQQGTII AGEEGSSTAG LNILAKGLTG DGKVLSQGQL NLQLNDDYVQ DAQ--GQLQA QGNLNLSSKG KVTNHGAIKS
 nos-D2  RISAEQQLNL NISDNLNNTK GLISSLDQLT IQGQQDNNRL IVNNQQGTII AGEEGSSTAS LNILAKGLTG DGKVLSQGQL NLQLNDDYVQ NAQ--GQLQA QGNLNLSSKG KVTNHGAIKS
 bau-D3  RISAEQQLNL NILDNLNNTK GLISSLDQLT IQGQQDNNRL IVNNQQGTII AGEEGSSTAG LNILAKGLTG DGKVLSQGQL NLQLNDDYVQ DAQ--GQLQA QGNLNLSSKG KVTNHGAIKS
 nos-D4  RISAEQQLNL NILDNLNNTK GLISSLDQLT IQGQQDNNRL IVNNQQGTII AGEEGSSTAG LNILAKGLTG DGKVLSQGQL NLQLNDDYVQ DAQ--GQLQA QGNLNLSSKG KVTNHGAIKS
 bau-D7  RISAEQQLNL NILDNLNNTK GLISSLDQLT IQGQQDNNRL IVNNQQGTII AGEEGSSTAG LNILAKGLTG DGKVLSQGQL NLQLNDDYVQ DAQ--GQLQA QGNLNLSSKG KVTNHGAIKS
 bau-D5  RISAEQQLNL NILDNLNNTK GLISSLDQLT IQGQQDNNRL IVNNQQGTII AGEEGSSTAG LNILAKGLTG DGKVLSQGQL NLQLNDDYVQ DAQ--GQLQA QGNLNLSSKG KVTNHGAIKS
 nos-D8  RISAEQQLNL NISDNLNNTK GLISSLDQLT IQGQQDNNRL IVNNQQGTII AGEEGSSTAS LNILAKGLTG DGKVLSQGQL NLQLNDDYVQ DAQ--GQLQA QGNLNLSSKG KVTNHGAIKS
 bau-D9  RISAEQQLNL NILDNLNNTK GLISSLDQLT IQGQQDNNRL IVNNQQGTII AGEEGSSTAG LNILAKGLTG DGKVLSQGQL NLQLNDDYVQ DAQ--GQLQA QGNLNLSSKG KVTNHGAIKS
 bau-D6  RISAEQQLNL NILDNLNNTK GLISSLDQLT IQGQQDNNRL IVNNQQGTII AGEEGSSTAG LNILAKGLTG DGKVLSQGQL NLQLNDDYVQ DAQ--GQLQA QGNLNLSSKG KVTNHGAIKS
nos-D12  RISAEQQLNL NILDNLNNTK GLISSLDQLT IQGQQDNNRL IVNNQQGTII AGEEGSSTAG LNILAKGLTG DGKVLSQGQL NLQLNDDYVQ DAQ--GQLQA QGNLNLSSKG KVTNHGAIKS
bau-D10  AIRSSQNSQL NISQQLNNQS SEISAVKQLG IQGDQ----L VMNNLNGQLL AGEN------ LNINAKSLTG DGKVLSLGNA DIQLKDSYQH NAT--AQLQA NQNLSLTSAG DINNDGTINA
bau-D11  AIRSSQNSQL NISQQLNNQS GEISAVKQLG IQGDQ----L AINNLNGQLL AGEN------ LNINAKSLTG DGRVLSLGNA DIQLKDSYQH NAT--AQLQA NQNLSLTSAG DINNDGVINA
bau-D14  AIRSSQNSQL NISQQLNNQS GEISAVKQLG IQGDQ----L AINNLNGQLL AGEN------ LNINAKSLTG DGRVLSLGNA DIQLKDSYQH NAT--AQLQA NQNLSLTSAG DINNDGVINA
bau-D12  AIRSSQNSQL NISQQLNNQS GEISAVKQLG IQGDQ----L AINNLNGQLL TGEN------ LNINAKSLTG DGRVLSLGNA DIQLKDSYQH NAT--AQLQA NQNLSLTSAG DINNDGVINA
bau-D20  AIRSSQNSQL NISQQLNNQS GEISAVKQLG IQGDQ----L AINNLNGQLL TGEN------ LNINAKSLTG DGRVLSLRNA DIQLKDSYQH NAT--AQLQA NQNLSLTSAG DINNDGVINA
bau-D15  TIRSSENSQL NISQQLNNQA GEISAVKQLG IQGDQ----L VVNNLNGQLL AGEN------ LNINAKSLTG DGKVLSLGNA DIQLKDSYQH NAT--AQLQA NQNLSLTSAG DINNDGTINA
bau-D16  AIRSSQNSQL NISQQLNNQS GEISAVKQLG IQGDQ----L AMNNLNGQLL AGEN------ LNINAKSLTG DGKVLSLGNA DIQLKDSYQH NAT--AQLQA NQNLSLTSAG DINNDGTINA
bau-D21  AIRSSQNSQL NISQQLNNQS GEISAVKQLG IQGDQ----L AMNNLNGQLL AGEN------ LNINAKSLTG DGKVLSLGNA DIQLKDSYQH NAT--AQLQA NQNLSLTSAG DINNDGTINA
bau-D19  AIRSSQNSQL NISQQLNNQS GEISAVKQLG IQGDQ----L AMNNLNGQLL AGEN------ LNINAKSLTG DGKVLSLGNA DIQLKDSYQH NAT--AQLQA NQNLSLTSAG DINNDGTINA
bau-D13  AIRSNQNAVL NVSGQLNNQS GEISAVKQLN LQGDQ----L VINNQKGKLL SGEN------ LNITAQSLTG DGQIASLGDA NITLKESFKQ TQE--GQLQA NKNLSLSTTS DITNDGKINA
cal-D23  AIRSNQNAVL NVSGQLNNQS GEISAVKQLG LQGDQ----L VINNQKGKLL SGGN------ LNITAQSLSG DGQIASLGDA NITLKENFKQ TQD--GQLQA NKNLSLSTAS DITNDGKINA
pit-D20  AIRSNQNAVL NISGQLSNQS GEISAVKQLN LQGNQ----L VINNQKGKLL SGEN------ LNITAQSLTG DGQIASLGDA NITLKESFKQ TQE--GQLQA NNNLSLSTAA DITNDGKINA
 pit-D6  AIRSNQNAVL NVSGQLSNQS GEISAVKQLN LQGNQ----L VINNQKGKLL SGEN------ LNITAQSLTG DGQIASLGDA NITLKESFKQ TQE--GQLQA NNNLSLSTAA DITNDGKINA
bau-D17  AIRSNQNAVL KVNGQLSNQS GEISAVKQLN LQGDQ----L VINNQKGKLL SGEN------ LNITAQSLTG DGQIASLGNA NITLKESFKQ TQE--GQLQA NNNLSLSTTS DITNDGKINA
nos-D18  AIRANDRAVL NIVQQLNNNQ GSISGQNLLS VVGSTTSGGL VVTNSQGLLS AGKQ------ LDLSSQSLSS DGRILSLGDT TLNLGSSYTQ TADNSSQLQA NGHLTLNTTG DINNDGVIQA
nos-D22  AIRANDRAVL NIVQQLNNNQ GSISGQNLLS VVGSTTSGGL VVTNSQGLLS AGKQ------ LDLSSQSLSS EGRILSLGDA TLNLGSSYTQ TADNSSQLQA NGHLTLNTTG DINNNGVIQA
 bau-D2  VIRASENATL NINNQLSNQL GSITSLNRLN IGTANKI--L NLNNTDGELL AKNQ------ LNLKANELVN KGKIISEGNV DIDLMQSYSH TQA--DQIAA NGTLKLSTEQ DLINQSELSA
bay-D15  VIRSSADQNL NVQQALNNQS GQISSEKQLT L--VGDQ--L EINNLNGQIL AGTA------ LDLIAQSLSG DGKLLSLGNA KLSLQNDYIH SSG--AQLQA NQDLSLSTKG NIINNGTINA

         3481                                                                                                                  3600
 bau-D1  NGQLSISANT IEN---AVDG SLESQQTQLE SVGVLNNYGL INGSNTYVKA GQLNNI-AGR IYGDHIAIQA NTLNNQ-SLK GLAPVIASRG DLDLGVQVLN NLENNLNHQ- ------SGSQ
 nos-D2  NGQLSISANT IEN---AIDG SLESQQTQLE SVGVLNNYGL INGSNTYVKA GQLNNI-AGR IYGDHIAIQA NTLNNQ-SLN GLAPVIASRG DLDLGVQVLN NLENNLNHQ- ------SGSQ
 bau-D3  NGQLSISANT IEN---AVDG SLESQQTQLE SVGVLNNYGL INGSNTYVKA GQLNNI-AGR IYGDHIAIQA NTLNNQ-SLK GLAPVIASRG DLDLGVQVLN NLENNLNHQ- ------SGSQ
 nos-D4  NGQLSILANT IEN---AVDG SLESQQTQLE SVGVLNNYGL INGSNTYVKA GQLNNI-AGR IYGDHIAIQA NTLNNQ-SLN GLAPVIASRG DLDLGVQVLN NLENNLNHQ- ------SGSQ
 bau-D7  NGQLSISANT IEN---AVDG SLESQQTQLE SVGVLNNYGL INGSNTYVKA GQLNNI-AGR IYGDHIAIQA NTLNNQ-SLK GLAPVIASRG DLDLGVQVLN NLENNLNHQ- ------SGSQ
 bau-D5  NGQLSISANT IEN---AVDG SLESQQTQLE SVGVLNNYGL INGSNTYVKA GQLNNI-AGR IYGDHIAIQA NTLNNQ-SLK GLAPVIASRG DLDLGVQVLN NLENNLNHQ- ------SGSQ
 nos-D8  NGQLSISANT IEN---AVDG SLESQQTQLE SVGVLNNYGL INGSNTYVKA GQLNNI-AGR IYGDHIAIQA NTLNNQ-SLN GLAPVIASRG DLDLGVQVLN NLENNLNHQ- ------SGSQ
 bau-D9  NGQLSISANT IEN---AVDG SLESQQTQLE SVGVLNNYGL INGSNTYVKA GQLNNI-AGR IYGDHIAIQA NTLNNQ-SLK GLAPVIASRG DLDLGVQVLN NLENNLNHQ- ------SGSQ
 bau-D6  NGQLSISANT IEN---AVDG SLESQQTQLE SVGVLNNYGL INGSNTYVKA GQLNNI-AGR IYGDHIAIQA NTLNNQ-SLK GLAPVIASRG DLDLGVQVLN NLENNLNHQ- ------SGSQ
nos-D12  NGQLSISANT IEN---AVDG SLESQQTQLE SVGVLNNYGL INGSNTYVKA GQLNNI-AGR IYGDHIAIQA NTLNNQ-SLK GLAPVIASRG DLDLGVQVLN NLENNLNHQ- ------SGSQ
bau-D10  GNQLQLSAVN ISN---SNNA KIESHDTQLI AQQQINNTGL INGDLTTLTA DTVNNQGTGR IYGTDLAIST NTLNNLPDAN GTAPVIASRG DMNLGVNVLN NLANTQDYD- ------SQAL
bau-D11  GNQLQLSAVN ISN---SSNA KIESHDTQLT AQQQLNNTGL INGDLTTLTA DTVNNQGTGR IFGTDLVISA NTLNNLPDAN GTAPVIASRG DMNLGVNVLN NLANTQDYD- ------SQAL
bau-D14  GNQLQLSAVN ISN---SSNA KIESHDTQLT AQQQLNNTGL INGDLTTLTA DTVNNQGTGR IFGTDLVISA NTLNNLPDAN GTAPVIASRG DMNLGVNVLN NLANTQDYD- ------SQAL
bau-D12  GNQLQLSAVN ISN---SSNA KIESHDTQLT AQQQLNNTGL INGDLTTLTA DTVNNQGTGR IYGTDLVISA NTLNNLPDAN GTAPVIASRG DMNLGVNVLN NLANTQDYD- ------SQAL
bau-D20  GNQLQLSAVN ISN---SSNA KIESHDTQLT AQQQLNNTGL INGDLTTLTA DTVNNQGTGR IYGTDLVISA NTLNNLPDAN GTAPVIASRG DMNLGVNVLN NLANTQDYD- ------SQAL
bau-D15  GNQLQLSAVN ISN---SNNA KIESHDTQLI AQQQINNTGL INGDLTTLTA DTVNNQGTGR IYGTDLAIST NTLNNLPDAN GTAPVIASRG DMNLGVNVLN NLANTQDYD- ------SQAL
bau-D16  GNQLQLSAVN ISN---SNNA KIESHDTQLI AQQQINNTGL INGDLTTLTA DTVNNQGTGR IYGTDLAIST NTLNNLPDAN GTAPVIASRG DMNLGVNVLN NLANTQDYD- ------SQAL
bau-D21  GNQLQLSAVN ISN---SSNA KIESHDTQLI AQQQINNTGL INGDLTTLTA DTVNNQGTGR IYGTDLAISA NTLNNLPDAN GTAPVIASRG DMNLGVNVLN NLANTQDYD- ------SQAL
bau-D19  GNQLQLSAVN ISN---SNNA KIESHDTQLI AQQQINNTGL INGDLTTLTA DTVNNQGTGR IYGTDLAIST NTLNNLPDAN GTAPVIASRG DMNLGVNVLN NLANTQDYD- ------SQAL
bau-D13  GNILKLNAAN ITN---SSNA QIESHDTQIE AQDQINNTGL INGDDTTLKA NTVNNQGTGR IYGTDLAISA NTLNNLPDIQ GRAPVIASRG DMNLGVGVLN NLANTQDYV- ------SQAL
cal-D23  GNILKLNAAN ITN---NSNA QIESHDTQIE AQDQINNTGL INGDYTTLKA NTVNNRGTGR IYGTDLAISA NTLNNLPDAQ GKAPVIASRG DMNLGVGVLN NLANTQDYA- ------SQAL
pit-D20  GNILKLNAAN ITN---NSNA QIESHDTQIE AQDQINNTGL INGDYTTLKA NTVNNQGTGR IYGTDLAISA NTLNNLPDVQ GKAPVIASRG DMNLGVGVLN NLANTQDYA- ------SQAL
 pit-D6  GNILKLNAAN ITN---NSNA QIESHDTQIE AQDQINNTGL INGDYTTLKA NTVNNQGTGR IYGTDLAISA NTLNNLPDTQ GKAPVIASRG DMNLGVGVLN NLANTQDYA- ------SQAL
bau-D17  GNILKLNAAN ITN---NSNA QIESHDTQIE AQDQINNTGL INGDDTTLKA NTVNNQGTGR IYGTDLAISA NTLNNLPDIQ GKAPVIASRG DMNLGVGVLN NLANTQDYA- ------SQAL
nos-D18  GQTLSLNAAN INNGLNNSTA RLESYDTQLS AQQQINNQGL INGELTTLRA NTVNNTGTGR IYGTSLAIGA NTLNNAPNSS GVAPVIASRG DMLLGVQTLN NMENPTDQNK GMQGTNNGAQ
nos-D22  GQTLSLNAAN INNGLNNSTA RLESYDTQLS AQQQINNQGL INGELTTLRA NTVNNTGTGR IYGTSLAIGA NTLNNAPNSS GVAPVIASRG DMLLGVQTLN NMENPTDQNK GMQGTNNGAQ
 bau-D2  GQKVELNAKN IQN---QTGA SISSNQTHLI AQDTIHNQGL INGELTHIQA NRVWNDGA-R IYGTQVAIQS NTLDNKSNAN GTGAVIASRG DMDLGIQILN NQSGGVVKEN G----RDNAW
bay-D15  GNQLALNAAN ITN---QQAG KIESHDTRLT AQNTLNNTGL INGDFTRLQA ETLNNQGTGR IYGTTLAIQA NTLNNQPDST GTAPVIASRG DLHLGVQTLN NLSNPT---D Y----TSQAL

         3601                                                                                                                  3720
 bau-D1  IIAMGDLRIG GSLDSQWHAQ GTAQQVNNRS SVINANGNID LNADIVNNQN VFFTTKQQQT TEQLDYWNMY ISDSRAGWYN PGPQSLITQE IYSQNPDLFT KLKPAQQLVT SY--------
 nos-D2  IIAMGDLRIG GSLDSQWHAQ GTAQQVNNRS SVINANGNID LNADTVNNQN VFFTTKQQQT TEQLDYWNIY ISDSRAGWYN PGPQSLITQE IYSQNPDLFT KLKPAQQLVT SY--------
 bau-D3  IIAMGDLRIG GSLDSQWHAQ GTAQQVNNRS SVINANGNID LNADIVNNQN VFFTTKQQQT TEQLDYWNMY ISDSRAGWYN PGPQSLITQE IYSQNPDLFT KLKPAQQLVT SY--------
 nos-D4  IIAMGDLRIG GSLDSQWHAQ GTAQQVNNRS SVINANGNID LNADTVNNQN VFFTTKQQQT TEQLDYWNMY ISDSRAGWYN PGPQSLITQE IYSQNPDLFT KLKPAQQLVT SY--------
 bau-D7  IIAMGDLRIG GSLDSQWHAQ GTAQQVNNRS SVINANGNID LNADIVNNQN VFFTTKQQQT TEQLDYWNMY ISDSRAGWYN PGPQSLITQE IYSQNPDLFT KLKPAQQLVT SY--------
 bau-D5  IIAMGDLRIG GSLDSQWHAQ GTAQQVNNRS SVINANGNID LNADIVNNQN VFFTTKQQQT TEQLDYWNMY ISDSRAGWYN PGPQSLITQE IYSQNPDLFT KLKPAQQLVT SY--------
 nos-D8  IIAMGDLRIG GSLDSQWHAQ GTAQQVNNRS SVINANGNID LNADTVNNQN VFFTTKQQQT TEQLDYWNMY ISDSRAGWYN PGPQSLITQE IYSQNPDLFT KLKPAQQLVT SY--------
 bau-D9  IIAMGDLRIG GSLDSQWHAQ GTAQQVNNRS SVINANGNID LNADIVNNQN VFFTTKQQQT TEQLDYWNMY ISDSRAGWYN PGPQSLITQE IYSQNPDLFT KLKPAQQLVT SY--------
 bau-D6  IIAMGDLRIG GSLDSQWHAQ GTAQQVNNRS SVINANGNID LNADIVNNQN VFFTTKQQQT TEQLDYWNMY ISDSRAGWYN PGPQSLITQE IYSQNPDLFT KLKPAQQLVT SY--------
nos-D12  IIAMGDLRIG GSLDSQWHAQ GTAQQVNNRS SVINANGNID LNADIVNNQN VFFTTKQQQT TEQLDYWNMY ISDSRAGWYN PGPQSLITQE IYSQNPDLFT KLKPAQQLVT SY--------
bau-D10  IFSAGNLYLG GALDENRKAT GQATVINNES ATIESLGDMR LSTQTINNIN KNFSTHE--- ---------- ---------- ---------- ---------- -VEVSRQEGL ----------
bau-D11  IFSAGNLYLG GALDENRKAT GQATVINNES ATIESLGDMR LSAQTINNIN KNFSTHE--- ---------- ---------- ---------- ---------- -VEVSRQEGL ----------
bau-D14  IFSAGNLYLG GALDENRKAT GQATVINNES ATIESLGDMR LSAQTINNIN KNFSTHE--- ---------- ---------- ---------- ---------- -VEVSRQEGL ----------
bau-D12  IFSAGNLYLG GALDENRKAT GQATVINNES ATIESLGDMR LSAKQINNIN KHFITDDN-- ---------- ---------- ---------- ---------- -VEVSRVDGL ----------
```

```
bau-D20  IFSAGNLYLG GALDENRKAT GQATVINNES ATIESLGDMR LSAKQINNIN KHFITDDN-- ---------- ---------- ---------- ---------- -VEVSRVDGL ----------
bau-D15  IFSAGNLYLG GALDENRKAT GQATVINNES ATIESLGDMR LSTQTINNIN KNFSTHE--- ---------- ---------- ---------- ---------- -VEVSRQEGL ----------
bau-D16  IFSAGNLYLG GALDENRKAT GQATVINNES ATIESLGDMR LSTQTINNIN KNFSTHE--- ---------- ---------- ---------- ---------- -VEVSRQEGL ----------
bau-D21  IFSAGNLYLG GALDENRKAT GQAVVINNES ATIESLGDMH LSAQTINNIN KNFSTHE--- ---------- ---------- ---------- ---------- -VETAREDGL ----------
bau-D19  IFSAGNLYLG GALDENRKAT GQATVINNES ATIESLGDMR LSTQTINNIN KNFSTHE--- ---------- ---------- ---------- ---------- -VEVSRQEGL ----------
bau-D13  IFSAANLYLG GALDENRKAT GQASVVNNES ATIESLGNMR LSAAQINNVN KHFITDDN-- ---------- ---------- ---------- ---------- -VEVSRIDGL ----------
cal-D23  IFSAGNLYLG GALDENRKAT GQASAVNNES ATIESLGNMR LSAAQINNIN KHFITDDN-- ---------- ---------- ---------- ---------- -VEVSRIDSL ----------
pit-D20  IFSAANLYLG GALDENSKAT GQASVVNNES ATIESLGNMR LSAAQINNMN KHLVTG-L-- ---------- ---------- ---------- ---------- -VETSRQDGL ----------
 pit-D6  IFSAGNLYLG GALDENRKAT GQASAINNES ATIESLGNMR LSAAQINNIN KHFITDDN-- ---------- ---------- ---------- ---------- -VEVSRIDGL ----------
bau-D17  IFSAANLYLG GALDENRKAT GQASVVNNES ATIESLGNMR LSAAQINNMN KHLVTG-L-- ---------- ---------- ---------- ---------- -VETSHQDGL ----------
nos-D18  IISMGDMHIG GALDSNRQVT GQASTLNNRS ATINVDGNLR LDVDQVNNQN LYVNTEIRSS APEQIDYWDM KANNPSWMWL NGRYNPGADS LITDVMYAQM AAQLSHLSGT TTAVSSNLES
nos-D22  IISLGDMHIG GALDSNRQVT GQASTLNNRS ATINVDGNLR LDVDQVNNQN LYVNTEIRSS APEQIDYWDM KANNPGWMWL NGRYNPGADS LITDVMYAQM AAQLSHLSGT TTAVSSNLES
 bau-D2  IFSAGQLNVG GSLNEELQAQ GNADKIYNGS AVIESLGDMY LGANNIQNTN ENLVIAL--- ---------- ---------- ---------- ---------- -IEKYRKQVH E---------
bay-D15  IFSAGNLYLG AALDANDYAT GQADVIHNES ATIESLGDMN LSAKQINNIN KYFKTEQ--- ---------- ---------- ---------- ---------- -VEVSRKDGL E---------

         3721                                                                                                                        3840
 bau-D1  ---------- --DNATMQDY LSRPDGYFFI VNNYDDSSAG NMFKGYVVYN GQLYSTDHYE NVKSTQTTTT TVADESAPAV ISAGGNLSFT --GTINNDKS QIMVGQKLIG DANAIHN---
 nos-D2  ---------- --DNATMQDY LSRPDGYFFI VNNYDDSSAG NMFKGYVVYN GQLYSTDHYE NVKSTQTTTT TVADESAPAV ISAGGNLSFT --GTINNDKS QIMVGQKLIG DAKAIHN---
 bau-D3  ---------- --DNATMQDY LSRPDGYFFI VNNYDDSSAG NMFKGYVVYN GQLYSTDHYE NVKSTQTTTT TVADESAPAV ISAGGNLSFT --GTINNDKS QIMVGQKLIG DANAIHN---
 nos-D4  ---------- --DNATMQDY LSRPDGYFFI VNNYDDSSAG NMFKGYVVYN GQLYSTDHYE NVKSTQTTTT TVADESAPAV ISAGGNLSFT --GTINNDKS QIMVGQKLIG DAKAIHN---
 bau-D7  ---------- --DNATMQDY LSRPDGYFFI VNNYDDSSAG NMFKGYVVYN GQLYSTDHYE NVKSTQTTTT TVADESAPAV ISAGGNLSFT --GTINNDKS QIMVGQKLIG DANAIHN---
 bau-D5  ---------- --DNATMQDY LSRPDGYFFI VNNYDDSSAG NMFKGYVVYN GQLYSTDHYE NVKSTQTTTT TVADESAPAV ISAGGNLSFT --GTINNDKS QIMVGQKLIG DANAIHN---
 nos-D8  ---------- --DNATMQDY LSRPDGYFFI VNNYDDSSAG NMFKGYVVYN GQLYSTDHYE NVKSTQTTTT TVADESAPAV ISAGGNLSFT --GTINNDKS QIMVGQKLIG DAKAIHN---
 bau-D9  ---------- --DNATMQDY LSRPDGYFFI VNNYDDSSAG NMFKGYVVYN GQLYSTDHYE NVKSTQTTTT TVADESAPAV ISAGGNLSFT --GTINNDKS QIMVGQKLIG DANAIHN---
 bau-D6  ---------- --DNATMQDY LSRPDGYFFI VNNYDDSSAG NMFKGYVVYN GQLYSTDHYE NVKSTQTTTT TVADESAPAV ISAGGNLSFT --GTINNDKS QIMVGQKLIG DANAIHN---
nos-D12  ---------- --DNATMQDY LSRPDGYFFI VNNYDDSSAG NMFKGYVVYN GQLYSTDHYE NVKSTQTTTT TVADESAPAV ISAGGNLSFT --GTINNDKS QIMVGQKLIG DANAIHN---
bau-D10  ---------- ---NKYFDGV WANFDGTVFR YMHPGE---- ---------- ------DSSS YDY-DEVITE TQVLTSAPAK IIAGGNMDLS -GSQVINDKS QIFAGGNLTN TGGTITNT--
bau-D11  ---------- ---NKYFDGV WANFDGTVFR YMHPGE---- ---------- ------DSSS YDY-DEVITE TQVLTSAPAK IIAGGNMDLS -GSQVINDKS QIFAGGNLTN TGGTITNT--
bau-D14  ---------- ---NKYFDGV WANFDGTVFR YMHPGE---- ---------- ------DFSS YDY-DEVITE TQVLTSAPAK IIAGGNMDLS -GSQVINDKS QIFAGGNLTN TGGTITNT--
bau-D12  ---------- ---EK-FCNA WCTFDGQ-YD GGNWFE---- ---------- ------DWMR YTY-SEITYE TRTVESAPGQ IKAGGNLDLS -GAQVLNDKS QILAGGELKN VGGTIDSG--
bau-D20  ---------- ---EK-FCNA WCTFDGQ-YD GGNWFE---- ---------- ------DWMR YTY-SEITYE TRTVESAPGQ IKAGGNLDLS -GAQVLNDKS QILAGGELKN VGGTIDSG--
bau-D15  ---------- ---NKYFDGV WANFDGTVFR YMHPGE---- ---------- ------DSSS YDY-DEVITE TQVLTSAPAK IIAGGNMDLS -GSQVINDKS QIFAGGNLTN TGGTITNT--
bau-D16  ---------- ---NKYFDGV WANFDGTVFR YMHPGE---- ---------- ------DSSS YDY-DEVITE TQVLTSAPAK IIAGGNMDLS -GSQVINDKS QIFAGGNLTN TGGTITNT--
bau-D21  ---------- ---VLHYQGV WANFDGTVFR NMNPRE---- ---------- ------DWSE YRY-SEVTYE TQALESAPAK IIAGGNMDLL -GSQVINDKS QILAGGELKN DGGSISSI--
bau-D19  ---------- ---NKYFDGV WANFDGTVFR YMHPGE---- ---------- ------DSSS YDY-DEVITE TQVLTSAPAK IIAGGNMDLS -GSQVINDKS QIFAGGNLTN TGGTITNT--
bau-D13  ---------- ---EK-FCNA WCTFDGQ-YD GGNWFE---- ---------- ------DWMR YTY-SEITYE TKAIESAPGQ IKAGGNLDLS -GAQVFNDKS QILAGGELIN LGGAIVSD--
cal-D23  ---------- ---EK-FCNA WCTFDGQ-YD GGNWFE---- ---------- ------DWMR YTY-SEITYE TKALESAPGQ IKVGGNLDLS -GAQVFNDKS QILAGGELIN LGGTIVSD--
pit-D20  ---------- ---EK-FCNA WCNFDGT-YY DGNWYE---- ---------- ------DWTK YHY-SEITYE TKAIESAPSQ IKAGGDLDLT -NAKVVNDSS QILAGGELKN DGGSITSK--
 pit-D6  ---------- ---EK-FCNA WCTFDGQ-YD GGNWFE---- ---------- ------DWMR YTY-SEITYE TKAIESAPGQ IKAGGNLDLS -GAQVFNDKS QILAGGELIN LGGAIISD--
bau-D17  ---------- ---EK-FCNA WCNFDGT-YY DGNWYE---- ---------- ------DWTK YHY-SEITYE TKAIESAPSQ IKAGGDLDLT -NAEVVNDSS QILAGGELKN DGGSITSK--
nos-D18  TSSSLPQFSS RNGNGSLEDY LAQSEGKFFI VSNYADSSRG QMTKGYLVLN GKLYTTDTYD HTYGTQTVTA PYVTNSAAGV ISVGGNLSFS -GA-INNDKS QILVGGTLLG DQGQSIAN--
nos-D22  TSSSLPQFSS RNGNGSLEDY LAQSEGKFFI VSNYADSSRG QMTKGYLVLN GKLYTTDTYD HTYGTQTVTA PYVTNSAAGV ISVGGNLSFS -GA-INNDKS QILVGGTLLG DQGQSIAN--
 bau-D2  ---------- ---YGKDGEV WDSSVIRLGS SSRGLSNAIL YVPQEEGGAP TREIGEDWSY YEY-TQIHSE DEVQSTSPAQ IIAGGNLSFT PDADFVNKDS QVLVGLQITN GIGSISNQST
bay-D15  ---------- -----KYCDA WCTF------ ---------- -----DGNYY DGNPYEDWMR YYY-SEITYE TRTTESAPGQ IKAGGHLDLN -GAQVVNDKS QILAGGELKN EGGTIDSG--

         3841                                                                                                                        3960
 bau-D1  -------VDA KGPKITHETG TVTPSYGSYG GKGHDRVWAS AEQAYNPADV TKGVDLFIYE NPTSATPVKV QDQTQQVNKD NLTEQALSQN HDSNIIGQQN TQVNQTETV- ------QQTT
 nos-D2  -------VDA KGPKITHEEG TVTPSYGSYG GKGHDRVWAS AEQAYNPADV TKGVDLFIYE NPTSATPVKV QDQTQQVNKD NLAEQALSQN HESTIIGQQN TQVNQTETV- ------QQTA
 bau-D3  -------VDA KGPKITHETG TVTPSYGSYG GKGHDRVWAS AEQAYNPADV TKGVDLFIYE NPTSATPVKV QDQTQQVNKD NLTEQALSQN HDSNIIGQQN TQVNQTETV- ------QQTT
 nos-D4  -------VDA KGPKITHEEG TVTPSYGSYG GKGHDRVWAS AEQAYNPADV TKGVDLFIYE NPTSATPVKV QDQTQQVNKD NLAEQALSQN HESTIIGQQN TQVNQTETV- ------QQTA
 bau-D7  -------VDA KGPKITHEEG TVTPSYGSYG GKGHDRVWAS AEQAYNPADV TKGVDLFIYE NPTSATPVKV QDQTQQVNKD NLTEQALSQN HDSNIVGQQN TQVNQTETV- ------QQTT
 bau-D5  -------VDA KGPKITHETG TVTPSYGSYG GKGHDRVWAS AEQAYNPADV TKGVDLFIYE NPTSATPVKV QDQTQQVNKD NLTEQALSQN HDSNIIGQQN TQVNQTETV- ------QQTT
 nos-D8  -------VDA KGPKITHEEG TVTPSYGSYG GKGHDRVWAS AEQAYNPADV TKGVDLFIYE NPTSATPVKV QDQTQQVNKD NLTEQALSQN HGSTIIGQQN TQVNQTETV- ------QQTA
 bau-D9  -------VDA KGPKITHEEG TVTPSYGSYG GKGHDRVWAS AEQAYNPADV TKGVDLFIYE NPTSATPVKV QDQTQQVNKD NLTEQALSQN HDSNIVGQQN TQVNQTETV- ------QQTT
 bau-D6  -------VDA KGPKITHEEG TVTPSYGSYG GKGHDRVWAS AEQAYNPADV TKGVDLFIYE NPTSATPVKV QDQTQQVNKD NLTEQALSQN HDSNIVGQQN TQVNQTETV- ------QQKT
nos-D12  -------VDA KGPKITHEEG TVTPSYGSYG GKGHDRVWAS AEQAYNPADV TKGVDLFIYE NPTSATPVKV QDQTQQVNKD KLTEQALSQN HDSNIVGQQN TQVNQTETV- ------QQTA
bau-D10  -------EVL -GNRETNVSG WQH-LYEKRS GK--DFFGDY QFSFSSVE-- --TIKLPV-- -SQTIEHYPL NQTNQDITRV SNNTSSNNIT SAQTNTTNVN GLKGTDQTTD V----GQAGT
bau-D11  -------EVL -GNRETNVSG WQH-LYEKRS GK--DFFGDY QFSFSSVE-- --TIKLPV-- -SQTIEHYPL NQTNQDITRV SNNTSSNNIT SAQTNTTNVN GLKVTDQTTD V----GQAGT
bau-D14  -------EVL -GNRETNVSG WQH-LYEKRS GK--DFFGDY QFSFSSVE-- --TIKLPV-- -SQTIEHYPL NQTNQDITRV SNNTSSNNIT SAQTNTTNVN GLKGTDQTID V----GQAGT
bau-D12  -------NSL DGIRKVVDQG IRT-KYWKYE GK-----WNN PFEVSTES-- --TFDLQI-- -SQALGNQNF SQNPISITAV ANNQSGEQVQ AGQTQVGHLN DLKDSDTSTQ V----GQVQN
bau-D20  -------NSL DGIRKVVDQG IRT-KYWKYE GK-----WNN PFEVSTES-- --TFDLQI-- -SQALGNQNF SQNPISITAV ANNQSGEQVQ AGQTQVGHLN DLKDSDTSTQ V----GQVQN
bau-D15  -------EVL -GNRETNVSG WQH-LYEKRS GK--DFFGDY QFSFSSVE-- --TIKLPV-- -SQTIEHYPL NQTNQDITRV SNNTSSNNIT SAQTNTTNVN GLKGTDQTTD V----GQAGT
bau-D16  -------EVL -GNRETNVSG WQH-LYEKRS GK--DFFGDY QFSFSSVE-- --TIKLPV-- -SQTIEHYPL NQTNQDITRV SNNTSSNNIT SAQTNTTNVN GLKGTDQTTD V----GQAGT
bau-D21  -------EAQ -GIRKIVDTG EQV-LYWKYE GE-----WHN PFEVSSET-- --TIDVPV-- -SQVADHYQL NQNSQSIATV SNNTSSNNIT SAQTNTTNIN GLKGTDQSTD V----GQAGT
bau-D19  -------EVL -GNRETNVSG WQH-LYEKRS GK--DFFGDY QFSFSSVE-- --TIKLPV-- -SQTIEHYPL NQTNQDITRV SNNTSSNNIT SAQTNTTNVN GLKGTDQTTD V----GQAGT
bau-D13  -------NSI KGTRKVVDRG TVY-KHWEYE GD-----WEG PFEVSSET-- --TIDVPI-- -GQVADHYEL NQTNQDITAV SKSAGDP-IT NAQIKTTHVN DLTATSNTTF V----EQAGV
cal-D23  -------NGI KGTRKVVDQG TVY-KHWEYE GD-----WEG PFEVSSET-- --TIDVPI-- -GQVADHYEL NQTNQDITAV SKNTNGP-IN SAQTKTTQVN GLTATSNTTF V----EQAGV
pit-D20  -------S-F EGIKRVVDTG TKE-IFWKYE GQ-----WTE PFEVSSET-- --TTNVSI-- -GQVADHYEL NQTNQDIAAV SKSNSDP-IT NAQTKTTHVN DLTATSNTTF V----EQAGV
 pit-D6  -------NGI KGTRKVVDQG TVY-KHWEYE GD-----WEG PFEVSSET-- --TIDVPI-- -GQVADHYEL NQTNQDITAV SKSAGGP-IT NAQIKTTHVN DLTATSNTTF V----EQAGV
bau-D17  -------S-F EGVKRIVDTG TKE-IFWKYE GQ-----WTE PFEVSSET-- --TTNVSI-- -GQVADHYEL NQTNQDISAV SKSNSAP-IT NAQTKTTHVN NLTATSNTTF V----EQAGV
nos-D18  -------LGM SVDQVTRISG QVD-HYVGYY GG--SGHDHI GIQDSTTNIP TKTTPLELFS SAPITGHQQV IRLNPSIDAR NTDKIGTGIA TATGTDGRSK DQQSLNNSTT VHGTTAQGGE
nos-D22  -------LGM SVDQVTRISG QVD-HYVGYY GG--SGHDHI GIQDSTTNIP TKTTPLELFS SAPITGHQQV IRLNPSIDAR NTDKIGTGIA TATGTDGRSK DQQSLNNSTT VHGTTAQGGE
 bau-D2  ALRSVDQTAP GGFSQWHSVG WNT-KGTEHR HRWGSKVNYQ PADIINTM-- --PISLGIVK EYTSQQSSNP VENLNSTNVQ TEIAQAQSVD LTALSTQKLN NQQLDLNAG- ------QSIA
bay-D15  -------TQI AGIRKIVNTG TQV-KYWKYE GQWDN----- PFENSSET-- --TFDLSISQ ALGNQ---NL NQNQLSTVAV SNNQSAEHIQ NSQTTQQEIN GLQTSNNNT- ------QLNQ

         3961                                                                                                                        4080
 bau-D1  A--------- --QTDRIQSN E--------- ---------- ---------- ---------- ---------- ---------- ---------- ---------- -------IKT GQAQLKGVV-
 nos-D2  A--------- --QSDRIQSN E--------- ---------- ---------- ---------- ---------- ---------- ---------- ---------- -------VKI GQAQLEGVV-
 bau-D3  A--------- --QTDRIQSN E--------- ---------- ---------- ---------- ---------- ---------- ---------- ---------- -------IKT GQAQLKGVV-
 nos-D4  A--------- --QSDRIQSN E--------- ---------- ---------- ---------- ---------- ---------- ---------- ---------- -------IKT GQAQLEGVV-
 bau-D7  A--------- --QTDRIQSN E--------- ---------- ---------- ---------- ---------- ---------- ---------- ---------- -------IKT GQAQLERVV-
 bau-D5  A--------- --QTDRIQSN E--------- ---------- ---------- ---------- ---------- ---------- ---------- ---------- -------IKT GQAQLKGVV-
 nos-D8  A--------- --QSDRIQSN E--------- ---------- ---------- ---------- ---------- ---------- ---------- ---------- -------IKT GQAQLEGVV-
 bau-D9  A--------- --QTDRIQSN E--------- ---------- ---------- ---------- ---------- ---------- ---------- ---------- -------IKT GQAQLERVV-
 bau-D6  A--------- --QTDRIQSN E--------- ---------- ---------- ---------- ---------- ---------- ---------- ---------- -------IKT GQAQLERVV-
nos-D12  A--------- --QSDRIQSN E--------- ---------- ---------- ---------- ---------- ---------- ---------- ---------- -------IKT GQAQLEGVV-
bau-D10  S--------- --QGQNLSVS DS-GQTAQGT DAQTTSQNPD ISQTTQNNDV TVSTSQVDVA DAQKAQGQDL VSAQPNAVQS DIQVSD---- ---------- -------LNQ DTANTQAKN-
bau-D11  S--------- --QGQNLSVS DS-GQTAQGT DAQTTSQNPD ISQTTQNNDV TVSTSQVDVA DAQKAQGQDL TSAQPDIVQS DIQVVG---- ---------- -------LNQ GTANTQAKD-
bau-D14  S--------- --QGQNLSVS DS-GQTAQGT DAQTTSQNPD ISQTTQNNDV TVSTSQVDVA DAQKAQGQDL VSAQPNAVQS DIQVSD---- ---------- -------LNQ DTANTQAKN-
bau-D12  A--------- --QGQQLNVA DT-GATSHQI SIESGQQNLS TQEMKELN-V DVDGSGLDVS QAGQASGQNL ASIQPDTIQS EVQVGD---- ---------- -------VNQ NTNIISGQD-
bau-D20  A--------- --QGQQLNVA DT-GATSHQI SIESGQQNLS TQEMKELN-V DVDGSGLDVS QAGQASGQNL ASIQPDTIQS DVQVGD---- ---------- -------VNQ NTNIISGKD-
bau-D15  S--------- --QGQNLSVS DS-GQTAQGT DAQTTSQNPD ISQTTQNNDV TVSTSQVDVA DAQKAQGQDL VSAQPNAVQS DIQVSN---- ---------- -------LNQ DTANTQAKN-
bau-D16  S--------- --QGQNLSVS DS-GQTAQGT DAQTTSQNPD ISQTTQNNDV TVSTSQVDVA DAQKAQGQDL VSAQPNAVQS DIQVSN---- ---------- -------LNQ DTANTQAKN-
bau-D21  S--------- --QGQNLSVS DS-GQTAQGT DAQTTSQSPD ISQTIQNNDV TVSTSQVDVA DAQKAQGQDL VSAQPNAVQS DIQVSN---- ---------- -------LNQ DTANTQAKN-
bau-D19  S--------- --QGQNLSVS DS-GQTAQGT DAQTTSQNPD ISQTTQNNDV TVSTSQVDVA DAQKAQGQDL VSAQPNAVQS DIQVSN---- ---------- -------LNQ DTANTQAKN-
bau-D13  A--------- --QNKNLQTA ---GQTS--- ---------- ---------- ---------- ---------- -------VKT DVQTSG---- ---------- -------LNG NSSNVQQKD-
cal-D23  A--------- --QNKDLQAA ---GQTS--- ---------- ---------- ---------- ---------- -------VKT DVKTLG---- ---------- -------LNG NSSNVQLKD-
pit-D20  A--------- --QNKNLQAA ---GQTS--- ---------- ---------- ---------- ---------- -------VKT DVQTSG---- ---------- -------LNG NSSNLQQKD-
 pit-D6  A--------- --QNKNLQAA ---GQTS--- ---------- ---------- ---------- ---------- -------VKT DVQTSG---- ---------- -------LNG NSSNIQQKD-
bau-D17  A--------- --QNKDLQAA ---GQTS--- ---------- ---------- ---------- ---------- -------VKT DVQTSG---- ---------- -------LNQ NSANVQQKD-
nos-D18  ATQVEAIKNS SQQGLNIHAA EGVGNRTGAI DQATVGAGNT AIDTIRNSNT ALKVDTATTV AKQQVSIGQT GQVSGSTAVS GVQVTDSGIT IVSLAGPEHG SAGPLDTLKV QTSNIGGSGL
nos-D22  ATQVEAIKNS SQQGLNIHAA EGVGNRTGAI DQATVGAGNT AIDTTRNSNT ALNVDTATTV AKQQVSIGQT GQVSGSTAVS GVQVTDSGIT IVSLAGPEHG SAGPLDTLKV QTGNIGGSGL
 bau-D2  E--------- --IGKNIQQP D--------- ---------- ---------- ---------- ---------- ---------- ---------- ---------- -------LPD QIGV------
bay-D15  T--------- --KDVDLDQL N--------- ---------- ---------- ---------- ---------- ---------- ---------- ---------- -------LKD SQSQ------

         4081                                                                                                                        4200
 bau-D1  ---GANTTDH KAMSNRAVDQ IQNTDTKAAS VQGQTTQIDG SG--FEIRT- NNANVKVPNN ALYSKNPDSR ANYLVETDPA FSNYRNWLSS DYMLNALGLD PALQQKRLGD GYYEQRMVQD
 nos-D2  ---GANTTNH KALSSSAVDQ IQNTDTKASA VQGQTTQIDG SG--FEIRT- NNANVKVPNN ALYSKNPDSR ANYLVETDPA FSNYRNWLSS DYMLNALGLD PALQQKRLGD GYYEQRMVQD
 bau-D3  ---GANTTDH KALSSSAVDQ IQNTDTKAAS VQGQTTQIDG SG--FEIRT- NNANVKVPNN ALYSKNPDSR ANYLVETDPA FSNYRNWLSS DYMLNALGLD PALQQKRLGD GYYEQRMVQD
 nos-D4  ---GANTTDH KAMSNRAVDQ IQNTDTKAAS VQGQTTQIDG SG--FEIRT- NNANVKVPNN ALYSKNPDSR ANYLVETDPA FSNYRNWLSS DYMLNALGLD PALQQKRLGD GYYEQRMVQD
 bau-D7  ---GANTTDH KAMSSSAVDQ IQNTDTKATS VQGQTTQIDG SG--FEIRT- NNANVKVPNN ALYSKNPDSR ANYLVETDPA FSNYRNWLSS DYMLNALGLD PALQQKRLGD AYYEQRMVQD
 bau-D5  ---GANTTDH KAMSNRAVDQ IQNTDTKAAS VQGQTTQIDG SG--FEIRT- NNANVKVPNN ALYSKNPDSR ANYLVETDPA FSNYRNWLSS DYMLNALGLD PALQQKRLGD GYYEQRMVQD
 nos-D8  ---GANTTDH KAMSSSAVDQ IQNTDTKASA VQGQTTQIDG SG--FEIRT- NNANVKVPNN ALYSKNPDSR ANYLVETDPA FSNYRNWLSS DYMLNALGLD PALQQKRLGD GYYEQRMVQD
 bau-D9  ---GANTTDH KAMSSSAVDQ IQNTDTKAAS VQGQTTQIDG SG--FEIRT- NNANVKVPNN ALYSKNPDSR ANYLVETDPA FSNYRNWLSS DYMLNALGLD PALQQKRLGD GYYEQRMVQD
 bau-D6  ---GANTTDH KAMSSSAVDQ IQNTDTKATS VQGQTTQIDG SG--FEIRT- NNANVKVPNN ALYSKNPNSR ANYLVETDPA FSNYRNWLSS DYMLNALGLD PALQQKRLGD GYYEQRMVQD
```

```
nos-D12  ---GANTTDH KAMSSSAVDQ IQNTDTKAAS VQGQTTQIDG SG--FEIRT- NNANVKVPNN ALYSKNPDSR ANYLVETDPA FSNYRNWLSS DYMLNGLGLD PALQQKRLGD GYYEQRMVQD
bau-D10  -----NTTQV SVLEQLQGQT AEQVSEASTT DQANAPDQAG TGEQLEIRTI NNSQFKVANN ALYRVGADSK AGYLIETDPS FTNYNQWLSS DYMLDALGLD PALQQKRLGD GFYEQRMVQD
bau-D11  -----NTTQV SVLDQLQGQT AEQVSEASTT DQANAPDQAG TGEQLEIRTI NNSQFKVANN ALYRVGADNK AGYLIETDPS FTNYNQWLSS DYMLNALGLD PALQQKRLGD GFYEQRMVQD
bau-D14  -----NTTQV SVLEQLQGQT AEQVSEASTT DQANAPDQAG TGEQLEIRTI NNSQFKVANN ALYRVGADSK AGYLIETDPS FTNYNQWLSS DYMLDALGLD PALQQKRLGD GFYEQRMVQD
bau-D12  -----NTAQV SVLDQLQGQT ASQVSGVTTT DQTTAPDQAG AGEQFEIRTI NTNNVKVANN ALYRTEADSK AGYLVETDPS FTNYNQWLSS DYMLNALGLD PALQQKRLGD GFYEQRMVQD
bau-D20  -----NTAQV SVLDQLQGQT ASQVSDVTTT DQTTAPDQAG AGEQFEIRTI NTNNVKVANN ALYRTESDSK AGYLVETDPS FTNYNQWLSS DYMLNALGLD PALQQKRLGD GFYEQRMVQD
bau-D15  -----NTTQV SVLEQLQGQT AEQVSEASTT DQANAPDQAG TGEQLEIRTI NDSQFKVANN ALYRVGADSK AGYLIETDSS FTNYNQWLSS DYMLDALGLD PALQQKRLGD GFYEQRMVQD
bau-D16  -----NTTQV SVLEQLQGQT AEQVSEASTT DQANAPDQAG TGEQLEIRTI NNSQFKVANN ALYRVGADSK AGYLIETDSS FTNYNQWLSS DYMLDALGLD PALQQKRLGD GFYEQRMVQD
bau-D21  -----NTTQV SVLEQLQGQT AEQVSEASTT DQANAPDQAG TGEQLEIRTI NNSQFKVANN ALYRVGADSK AGYLIETDPS FTNYNQWLSS DYMLDALGLD PALQQKRLGD GFYEQRMVQD
bau-D19  -----NTTQV SVLEQLQGQT AEQVSEASTT DQANAPDQAG TGEQLEIRTI NNSQFKVANN ALYRVGADSK AGYLIETDSS FTNYNQWLSS DYMLDALGLD PALQQKRLGD GFYEQRMVQD
bau-D13  -----NTTQI SVLDQLQGQK ASQVSGVSTS DQTTAPDQAG TGEQLEIRTI NNNNIKVANN ALYRTGADSK AGYLIETDPN FTNYNQWLSS DYMLNALGLD PALQQKRLGD GFYEQRMVQD
cal-D23  -----NTTQI SVLDQLQGQK ASQVSGVLTS DQATAPDQAG TGEQLEIRTI NNNNIKVANN ALYRTGADSK VGYIIETDPD FTNYNQWLSS DYMLDALGLD PTLQQKRLGD GFYEQRMVQD
pit-D20  -----NTTQI SVLDQLQGQK ASQVSGASTS GQATAPDQAG TGEQLEIRTI NNNNIKVANN ALYRTGADSK AGYLIETDPN FTNYNQWLSS NYMLSALGLD PALQQKRLGD GFYEQRMVQD
 pit-D6  -----NTTQI SVLDQLQGQK ASQVSGVSTS DQATAPDQAG TGEQLEIRTI NNNNIKVANN ALYRTGADSK AGYLIETDPN FTNYNKWLSS DYMLNALGLD PALQQKRLGD GFYEQRMVQD
bau-D17  -----NTTQI SVLDQLQGQK ASQVSGVSTS DQATAPDQAG TGEQLEIRTI NNNNIKVANN ALYRTGADSK AGYLIETDPN FTNYNQWLSS DYMLNALGLD PALQQKRLGD GFYEQRMVQD
nos-D18  DGEVKNSTTV NQASMLTGEQ AQQAQANATN SSVATGTKAV TAPSYEIRTI NTDGQKISNS ALYRINPDSR SGYIIETDPD FSQYRQWLSS DYMLNALGLD PAFQQKRLGD GYYEQRQVQD
nos-D22  DGEVKNSTTV NQASMLTGEQ AQQAQANATN SSVATGTKAV TAPSYEIRTI NTDGQKISNS ALYRINPDSR SGYIIETDPD FSQYRQWLSS DYMLNALGLD PAFQQKRLGD GYYEQRQVQD
 bau-D2  -----EANPT TDIQAEQ-NN IDRVEGEELN QVNNTASAVD SATDITVRTV SQDFLNLPSN ALFITTKDSQ AQYLVETDPA YANYRNWLSS DYMIKALGLD PAMQQKRIGD GYYEQRLVQD
bay-D15  -----NAPQA SAVETVQ-QQ ---------- SATSAPSSTP QGTSEEIRSI DFSNIKVPNS ALYKVNADSK AGYLIETDPA FADYKKWLSS DYMLDALGLD PALQQKRLGD GFYEQRLVQD

         4201                                                                                                                  4320
 bau-D1  QVAQLTGYRF LQGYGSDEEQ YKALMNNGLS YAKQYNLRPG IALTPEQMAQ LSSDIVWLVE KETTLPDGTK TKALVPQVYV KARVGDLKGD GTLISADSIQ LNLSKDLTNA GTIAGRQVVQ
 nos-D2  QVAQLTGYRF LQGYGSDEEQ YKALMNNGLS YAKQYNLRPG IALTPEQMAQ LSSDIVWLVE KETTLPDGTK TKALVPQVYV KARVGDLKGD GTLISADSIQ LNLSKDLTNA GTIAGRQVVQ
 bau-D3  QVAQLTGYRF LQGYGSDEEQ YKALMNNGLS YAKQYNLRPG IALTPEQMAQ LSSDIVWLVE KETTLLDGTK TKALVPQVYV KARVGDLKGD GTLISADSIQ LNLSKDLTNA GTIAGRQVVQ
 nos-D4  QVAQLTGYRF LQGYGSDEEQ YKALMNNGLS YAKQYNLRPG IALTPEQMAQ LSSDIVWLVE KETTLPDGTK TKALVPQVYV KARVGDLKGD GTLISADSIQ LNLSKDLTNA GTIAGRQVVQ
 bau-D7  QVAQLTGYRF LQGYGSDEEQ YKALMNNGLS YAKQYNLRPG IALTPEQMAQ LSSDIVWLVE KETTLPDGTK TKALVPQVYV KARVGDLKGD GTLISADSIQ LNLSKDLTNA GTIAGRQVVQ
 bau-D5  QVAQLTGYRF LQGYGSDEEQ YKALMNNGLS YAKQYNLRPG IALTPEQMAQ LSSDIVWLVE KETTLPDGTK TKALVPQVYV KARVGDLKGD GTLISADSIQ LNLSKDLTNA GTIAGRQVVQ
 nos-D8  QVAQLTGYRF LQGYGSDEEQ YKALMNNGLS YAKQYNLRPG IALTPEQMAQ LSSDIVWLVE KETTLPDGTK TKALVPQVYV KARVGDLKGD GTLISADSIQ LNLSKDLTNA GTIAGRQVVQ
 bau-D9  QVAQLTGYRF LQGYGSDEEQ YKALMNNGLS YAKQYNLRPG IALTPEQMAQ LSSDIVWLVE KETTLLDGTK TKALVPQVYV KARVGDLKGD GTLISADSIQ LNLSKDLTNA GTIAGRQVVQ
 bau-D6  QVAQLTGYRF LQGYGSDEEQ YKALMNNGLS YAKQYNLRPG IALTPEQMAQ LSSDIVWLVE KETTLPDGTK TKALVPQVYV KARVGDLKGD GTLISADSIQ LNLSKDLTNA GTIAGRQVVQ
nos-D12  QVAQLTGYRF LQGYGSDEEQ YKALMNNGLS YAKQYNLRPG IALTPEQMAQ LSSDIVWLVE KETTLPDGTK TKALVPQVYV KARVGDLKGD GTLISADSIQ LNLSKDLTNA GTIAGRQVVQ
bau-D10  QIANLTGYRF LEGYGSDEEQ YKALMNNGVT YAKQYGLRPG IALTPAQIAQ LTSDIVWLVE KDVTLPDGTQ TKALAPQVYV KARVGDLKGD GTLLSGNSIK FNLDGDLLNT ATIAGREAVQ
bau-D11  QIANLTGYRF LEGYGSDEEQ YKALMNNGIT YAKQYGLRPG IALTPAQIAQ LTSDIVWLVE KDVTLPDGTQ TKALAPQVYV KARAGDLKGD GTLLSGNSIK FNLDGDLLNT ATIAGREAVQ
bau-D14  QIANLTGYRF LEGYGSDEEQ YKALMNNGVT YAKQYGLRPG IALTPAQIAQ LTSDIVWLVE KDVTLPDGTQ TKALAPQVYV KARAGDLKGD GTLLSGNSIK FNLDGDLLNT ATIAGREAVQ
bau-D12  QIANLTGYRF LEGYASDEEQ YKALMNNGVT FAKQYGLRPG IALSAAQIAQ LTSDIVWLVE KDVTLQDGTK TKALIPQVYV KARVGDLKGD GTLLTGNSVN FKLNGDLLNG STIAGREAVQ
bau-D20  QIANLTGYRF LEGYASDEEQ YKALMNNGVT FAKQYGLRPG IALSAAQIAQ LTSDIVWLVE KDVTLQDGTK TKALVPQVYV KARVGDLKGD GTLLTGNSVN FKLNGDLLNG STIAGREAVQ
bau-D15  QIANLTGYRF LEGYGSDEEQ YKALMNNGVT YAKQYGLRPG IALTPAQIAQ LTSDIVWLVE KDVTLPDGTQ TKALAPQVYV KARAGDLKGD GTLLSGNSIK FNLDGDLLNT ATIAGREAVQ
bau-D16  QIANLTGYRF LEGYGSDEEQ YKALMNNGVT YAKQYGLRPG IALTPAQIAQ LTSDIVWLVE KDVTLPDGTQ TKALAPQVYV KARAGDLKGD GTLLSGNSIK FNLDGDLLNT ATIAGREAVQ
bau-D21  QIANLTGYRF LEGYGSDEEQ YKALMNNGVT YAKQYGLRPG IALTPAQIAQ LTSDIVWLVE KDVTLPDGTQ TKALAPQVYV KARAGDLKGD RTLLSGNSIK FNLDGDLLNT ATIAGREAVQ
bau-D19  QIANLTGYRF LEGYGSDEEQ YKALMNNGVT YAKQYGLRPG IALTPAQIAQ LTSDIVWLVE KDVTLPDGTQ TKALAPQVYV KARAGDLKGD GTLLSGNSIK FNLDGDLLNT ATIAGREAVQ
bau-D13  QIANLTGYRF LEGYASDEEQ YKALMNNGVT FAKQYGLRPG IALSAAQIAQ LTSDIVWLVE KDVTLQDGTK TKALVPQVYV KARVGDLKGD GTLLTGNSVN FKLNGDLLNG STIAGREAVQ
cal-D23  QIANLTGYRF LEGYASDEEQ YKALMNNGVT FAKQYGLRPG IALSAAQIAQ LTSDIVWLVE KDVTLQDGTK TKALVPQVYV KARVGDLKGD GSLLTGNSVN FKLNGDLLNG STIAGREAVQ
pit-D20  QIANLTGYRF LEGYASDEEQ YKALMNNGVT FAKQYGLRPG IALSAAQIAQ LTSDIVWLVE KDVTLQDGTK TKALVPQVYV KARVGDLKGD GSLLTGNSVN FKLNGDLLNG STIAGREAVQ
 pit-D6  QIANLTGYRF LEGYASDEEQ YKALMNNGVT FAKQYGLRPG IALSAAQIAQ LTSDIVWLVE KDVTLQDGTK TKALVPQVYV KARVGDLKGD GSLLTGNSVN FKLNGDLLNG STIAGREAVQ
bau-D17  QIANLTGYRF LEGYASDEEQ YKALMNNGVT FAKQYGLRPG IALSAAQIAQ LTSDIVWLVE KDVTLQDGTK TRALVPQVYV KARVGDLKGD GSLLTSNSVN FKLNGDLLNG STIAGREAVQ
nos-D18  QIAKLTGSRY LVGYHSDEEQ YKALLDAGVT FAQQYSLRPG VALTAAQMAE LTSDIVWLVE KEVTLTNGTT TRALVPQVYV RAKAGDLLGD GSLIAANDIQ LKLKGNVLNE ATIAGRHALQ
nos-D22  QIAKLTGSRY LVGYHSDEEQ YKALLDAGVT FAQQYSLRPG VALTAAQMAE LTSDIVWLVE KEVTLTNGTT TRALVPQVYV RAKAGDLLGD GSLIAANDIQ LKLKGNVLNE ATIAGRHALQ
 bau-D2  QVAKLTGYRF LEGYGSDEEQ YKALMNNGLS FAKQYKLRPG IALTETQIAQ LTSDIVWLEE KTVKLADGTT TQALVPQIYV KARVGDLKGD GTLISADSIQ LDVQGDVFNS GTIAGRQAVV
bay-D15  QIANLTGYRF LEGFANDDEQ YKALMNNGIT FAKLYGLRPG IALTAAQTAQ LTSDIVWLVE QSVTLPDGTI TKALVPQVYV KVRVGDLKGD GTLLSGNSVN LKLDGDLING ATIAGREALQ

         4321                                                                                                                  4440
 bau-D1  INSQNLNNLA GNIRGGVVSI STEKDLNNIG GMIQADQAMN LQVKGNLNIQ STTQSSQNA- ---------- LGQSNYSWTG IDRIAGLYIG S----AQQQA LSDQPTLVMN VGGDTRILAA
 nos-D2  INSQNLNNLA GNIRGGVVSL GTEKDLNNIG GMIQADQAMN LQVKGNLNIQ STTQSSQNA- ---------- LGQSNYSWTG IDRIAGLYVG S----AQQQA LSDQPTLVMN VGGDTRILAA
 bau-D3  INSQNLNNLA GNIRGGVVSI STEKDLNNIG GMIQADQAMN LQVKSNLNIQ STTQSSQNA- ---------- LGQSNYSWTG IDRIAGLYIG S----AQQQA LSDQPTLVMN VGGDTRILAA
 nos-D4  INSQNLNNLA GNIRGGVVSL GTEKDLNNIG GMIQADQAMN LQVKGNLNIQ STTQSRQNA- ---------- LGQSNYSWTG IDRIAGLYVG S----AQQQA LSDQPTLVMN VGGDTRILAA
 bau-D7  INSQNLNNLA GNIRGGVVSL STEKDLNNIG GMIQADQAMN LQVKGNLNIQ STTQSSQNA- ---------- LGQSNYSWTG IDRIAGLYVG S----AQQQA LSDQPTLVMN VGGDTRILAA
 bau-D5  INSQNLNNLA GNIRGGVVSI STEKDLNNIG GMIQADQAMN LQVKGNLNIQ STTQSSQNA- ---------- LGQSNYSWTG IDRIAGLYVG S----AQQQA LSDQPTLVMN VGGDTRILAA
 nos-D8  INSQNLNNLA GNIRGGVVSL STEKDLNNIG GMIQADQAMN LQVKGNLNIQ STTQSSQNA- ---------- LGQSNYSWTG IDRIAGLYVG S----AQQQA LSDQPTLVMN VGGDTRILAA
 bau-D9  INSQNLNNLA GNIRGGVVSI STEKDLNNIG GMIQADQAMN LQVKGNLNIQ STTQSSQNA- ---------- LGQSNYSWTG IDRIAGLYIG S----AQQQA LSDQPTLVMN VGGDTRILAA
 bau-D6  INSQNLNNLA GNIRGGVVSL GTEKDLNNIG GMIQADQAMN LQVKGNLNIQ STTQSSQNA- ---------- LGQSNYSWTG IDRIAGLYVG S----AQQQA LSDQPTLVMN VGGDTRILAA
nos-D12  INSQNLNNLA GNIRGGVVNL GTEKDLNNIG GMIQADQAMN LQVKGNLNIQ STTQSSQNA- ---------- LGQSNYSWTG IDRIAGLYVG S----AQQQA LSDQPTLVMN VGGDTRILAA
bau-D10  ITADNVNNLM GRIQGNTVDI TSKKDLNNIG GSIQAKDAMA MNVGGNLNVT STTRTTDTQ- ---------- VGGFSASTTG LDRVAGLYVG D---IQAGAV DLNKATFVLN VAGDSVLKGA
bau-D11  ITADNVNNLM GRIQGNTVDI TSKKDLNNIG GSIQAKDAMA MNVGGNLNVT STTRTTDTQ- ---------- VGGFSASTTG LDRVAGLYVG D---IQAGAV DLNKATFVLN VAGNSVLKGA
bau-D14  ITADNVNNLM GRIQGNTVDI TSKKDLNNIG GSIQAKDAMA MNVGGNLNVT STTRTTDTQ- ---------- VGGFSASTTG LDRVAGLYVG D---IQAGAV DLNKATFVLN VAGNSVLKGA
bau-D12  ITADNINNLK GRIQGNIVDI TTQKDLNNIG GQISAKDAMA VKVGGNLKIE TTTRTSTSQ- ---------- VGDFSASTTG VDRVAGLYVG S-----DNKI QPNKATLMVD VAGNTVLKAA
bau-D20  ITADNINNLK GRIQGNTVDI TTQKDLNNIG GQISAKDAMA VKVGGNLKIE TTTRTSTSQ- ---------- VGDFSASTTG VDRVAGLYVG S-----GNKI QPNKATLMVD VAGNTVLKAA
bau-D15  ITADNVNNLM GRIQGNAVDI TSKKDLNNIG GSIQAKDTMA MNVGGNLNVT STTRTTDTQ- ---------- VGGFSASTTG LDRVAGLYVG D---IQAGAV DLNKATFVLN VAGNSVLKGA
bau-D16  ITADNVNNLM GRIQGNAVDI TSKKDLNNIG GSIQAKDTMA MNVGGNLNVT STTRTTDTQ- ---------- VGGFSASTTG LDRVAGLYVG D---IQAGAV DLNKATFVLN VAGNSVLKGA
bau-D21  ITADNVNNLM GRIQGNTVDI TSKKDLNNIG GSIQAKDAMA MNVGGNLNVT STTRTTDTQ- ---------- VGGFSASTTG LDRVAGLYVG D---IQAGAV DLNKATFVLN VAGDSVLKGA
bau-D19  ITADNVNNLM GRIQGNAVDI TSKKDLNNIG GSIQAKDTMA MNVGGNLNVT STTRTTDTQ- ---------- VGGFSASTTG LDRVAGLYVG D---IQAGAV DLNKATFVLN VAGNSVLKGA
bau-D13  ITADNINNLK GRIQGNTVDI TTQKDLNNIG GQISAKDAMA VKVGGNLKIE TTTRTSTSQ- ---------- VGDFSASTTG VDRVAGLYVG S-----GNKI QPNKATLMVD VAGNTVLKAA
cal-D23  ITADNINNLM GRIQGNTVDI TTKKDLNNIG GQISAKDAMA VKVGGNLKIE TTTRTSTTQ- ---------- VGGFGSSTTG VDRVAGLYVG S-----GSKI QPNKATLVVD VAGNTVLKAA
pit-D20  ITANNINNLM GRIQGNTVDI TTKKDLNNIG GQISAQDAMA VKVGGNLKIE TTTKTSESQ- ---------- IGGLGSSTTG VDRVAGLYVG S-----GNKI QPNKATLVVD VAGNTVLKAA
 pit-D6  ITANNINNLM GRIQGNTVDI TTKKDLNNIG GQISAKDAMA VKVGGNLKIE TTTRTSTTQ- ---------- VGGFGSSTTG VDRVAGLYVG S-----GNKI QPNKATLVVD VAGNTVLKAA
bau-D17  ITANNINNLM GRIQGNTVDI TTKKDLNNIG GQISAKEAMA VKVGGNLKIE TTIRTSTTQ- ---------- VGDFGSSTTG VDRVAGLYVG S-----GNKI QPNKATLMVD VAGNTVLKAA
nos-D18  ISAEQIDNIG GRIEGNQVAL TTQKDLNNIG GTLKAGSAMA LNVGGNFNHV STSSTSISGN PVFGQGGMNG ISGNYTSQTS IDRIAGLYVG D----GTGPI DPNNVSLVMN VGGKSTLTAA
nos-D22  ISAEQIDNIG GRIEGNQVAL TTQKDLNNIG GTLKAGSAMA LNVGGNFNHV STSSTSISGN PVFGQGGMNG ISGNYTSQTS IDRIAGLYVG D----GTGPI DPNNVSLVMN VGGKSTLTAA
 bau-D2  LNAENVEILN GRIQANQVGL NTSKDLNNIIG GQIQAEQAVD LNVGRNFNLE SSTQHSENK- ---------- IGESTFTYTG LDRVAGVYTK APTAIKSSDT ENLKTSISIR VGGDSDLKAA
bay-D15  ITADNVQNLN GRIQGNQVAI ETQKDLNNIG GQIIAKDAMA LKVGGNLNIE STTRTSESK- ---------- VGNFSSSITN VDRVAGLYVG NGS---TKQL DPNQATLMLN VAGNSSLKGV

         4441                                                                                                                  4560
 bau-D1  NIQNNSGGRS IIRTGGDLKV GSLNTEVTNN VVGSSKAYHL DTRQVEVGSQ IKSQGDLSLQ GNNIQIHGSQ ISSSEGLTAL QAKERLQIEE GRNKLDQQDQ SSFSKKGKLS SSTTSDFSHR
 nos-D2  NIQNNSGGQS IIRTGGDLKV GSLNTEVTNN VVGSSKAYHL DTRQVEVGSQ IKSQGDLSLQ GNNIQIHGSQ ISSSEGLTAL QAKEQLQIEE GRNKLDQQDQ SSFSKKGKLS SSTTSDFSHR
 bau-D3  NIQNNSGGRS IIRTGGDLKV GSLNTEVTNN VVGSSKAYHL DTRQVEVGSQ IKSQGDLSLQ GNNIQIHGSQ ISSSEGLTAL QAKEQLQIEE GRNKLDQQDQ SSFSKKGKLS SSTTSDFSHR
 nos-D4  NIQNNSGGQT IIRTGGDLKV GSLNTEVTNN VVGSSKAYHL DTRQVEVGSQ IKSQGDLSLQ GNNIQIHGSQ ISSSEGLTAL QAKEQLQIEE GRNKLDQQDQ SSFSKKGKLS SSTTSDFSHR
 bau-D7  NIQNNSGGQS IIRTGGDLKV GSLNTEVTNN VVGSSKAYHL DTRQVEVGSQ IKSQGDLSLQ GNNIQIHGSQ ISSSEGLTAL QAKEQLQIEE GRNKLDQQDQ SSFSKKGKLS SSTTSDFSHR
 bau-D5  NIQNNSGGQS IIRTGGDLKV GSLNTEVTNN VVGSSKAYHL DTRQVEVGSQ IKSQGDLSLQ GNNIQIHGSQ ISSSEGLTAL QAKEQLQIEE GRNKLDQQDQ SSFSKKGKLS SSTTSDFSHR
 nos-D8  NIQNNSGGQS IIRTGGDLKV GSLNTEVTNN VVGSSKAYHL DTRQVEVGSQ IKSQGDLSLQ GNNIQIHGSQ ISSSEGLTAL QAKEQLQIEE GRNKLDQQDQ SSFSKKGKLS SSTTSDFSHR
 bau-D9  NIQNNSGGRS IIRTGGDLKV GSLNTEVTNN VVGSSKAYHL DTRQVEVGSQ IKSQGDLSLQ GNNIQIHGSQ ISSSEGLTAL QAKEQLQIEE GRNKLDQQDQ SSFSKKGKLS SSTTSDFSHR
 bau-D6  NIQNNSGGQS IIRTGGGLKV GSLNTEVTNN VVGSSKAYHL DTRQVEVSSQ IKSQGDLSLQ GNNIQIHGSQ ISSSEGLTAL QAKEQLQIEE GRNKLDQQDQ SSFSKKGKLS SSTTSDFSHR
nos-D12  NIQNNSGGRS IIRTGGDLKV GSLNTEVTNN VVGSSKAYHL DTRQVEVGSQ IKSQGDLSLQ GNNIQIHGSQ ISSSEGLTAL QAKEQLQIEE GRNKLDQQDQ SSFSKRGKLS SSTTSDFSHR
bau-D10  EI-NNSNGAT LINTQGNVDI GTVQTGYKLG TVLDARNNSV SQKTQDVGSQ INSTGSLLIN GQNINIKGSG LNS-QATTQL AAVKQLNIEE GRQTSQLDSQ WYSKRKGTLS SKTDTGSIHD
bau-D11  EI-NNSNGAT LINTQGNVDI GTVQTGYKLG TVLDARNNSV SQKTQDVGSQ INSTGSLLIN GQNINIKGSG LNS-QATTQL AAVKQLNIEE GRQTSQLDSQ WYSKRKGTLS SKTDTGSIHD
bau-D14  EI-NNSNGAT LINTQGNVDI GTVQTGYKLG TVLDARNNSV SQKTQDVGSQ INSTGSLLIN GQNINIKGSG LNS-QATTQL AAVKQLNIEE GRQTSQLDSQ WYSKRKGTLS SKTDTGSIHD
bau-D12  EL-NNSSGAS VINTVGNVDI GTVQTGYKLS TYRDIQNQTT SQKAQDVGSQ LNSNGSLLLN GQNIKIQGSG LNSVQGTAQI SAVNQLDIEE GRKTSQFDRQ WYEKHKGTLG SKTDTGFTHD
bau-D20  EL-NNSNGAS VINTVGNVDI GTLQTGYKLS TYRDIQNQTT SQKAQDVGSQ LNSNGSLLLN GQNIKIQDSG LNSVQGTAQI SAVNQLDIEE GRKTSQFDRQ WYEKHKGTLG SKTDTGFTHD
bau-D15  EI-NNSNGAT LINTQGNVDI GAVQIGKQEQ LNLDAKNNYK IAQQQDIGSQ ISSAGTLAIQ GKNITGKAVQ FSSQIGNVEL LAENDIQLEN GLTQSTASSQ FAYKSSFG-G KKSSTYNAHE
bau-D16  EI-NNSNGAT LINTQGNVDI GAVQIGKQEQ LNLDAKNNYK IAQQQDIGSQ ISSAGTLAIQ GKNITGKAVQ FSSQIGNVEL LAENDIQLEN GLTQSTASSQ FAYKSSFG-G KKSSTYNAHE
bau-D21  EI-NNSNGAT LINTQGNVDI GAVQIGKQEQ LNLDAKNNYK IAQQQDIGSQ ISSAGTLAIQ GKNITGKAVQ LSSQIGNVEL LAENDIQLEN GLTQSTASSQ FAYKSSFG-G KKSSTYNAHE
bau-D19  EI-NNSNGAT LINTQGNVDI GAVQIGKQEQ LNLDAKNNYK IAQQQDIGSQ ISSAGTLAIQ GKNITGKAVQ FSSQIGNVEL LAENDIQLEN GLTQSTASSQ FAYKSSFG-G KKSSTYNAHE
bau-D13  EL-NNSNGAS VINTVGNVDI GTVQTGYKLS TYRDIQNQTT SQKAHDVGSQ LNSNGSLLLN GQNIKIQGSG LNSVQGTAQI SAVNQLDIEE GRKTSQFDRQ WYEKHKGTLG SKTDTGFTHD
cal-D23  EL-NNSNGAA VINTVGNVDI GTVQTGYKLT TYRDAQNHAT SQKTQDVGSQ LNSNGSLLLN GQNIKVQGSG LNSEQGTTQL SAVNQLDIEE GRKTSQFDRQ WYEKHKGTLG SKTDTGFTND
pit-D20  EL-NNNNGAA VINTVGNVDI GTVQTGYKLS TYRDAQNHST SQKTQDVGSQ VNSNGSLLLN GQNIKVQGSG LNSEQGTTQL SAVNQLDIVE GRKTSELDSQ WYNKHKGTLS SKTTSGFIHD
 pit-D6  EL-NNSNGAA VINTVGNVDI GTVQKGYKLT TYRDAQNHAT SQKTQDVGSQ LNSNGSLLLN GQNIKVQGSG LNSVQGTTQL SAVNQLDIEE GRKTSQFDRQ WYEKHKGTLG SKTDTGFTHD
bau-D17  EL-NNSNGAS VINTIGNVDI GTVQTGYKLS TYRDAQNHAT SQKVQDVGSQ IASAGSLIVQ GQNISGKAIQ LSSQAGNIEL LAQKDIQLDN GLKQNSVDSK --ASWKGSFG GKKSSTYKAH
nos-D18  KI-ENRNGAS LIQIQGNVEL GTIKVGQQHD NGVSGSGRYS SGSSQEIGSS ITSVGNTVIT GNNIHGTAVK LGSQQGDVAL LAEHNLTLGD GRSLNE---- ---EHYTTGN KKNGSSHDAN
nos-D22  KI-ENRNGAS LIQIQGNVEL GTIKVGQQYD NGVSGSGRYS SGSSQEIGSS ITSVGNTVIT GNNIHGTAVK LGSQQGDVAL LAEHNLTLDD GRSLNE---- ---EHYTTGN KKNGSSHDAN
 bau-D2  QI-QNANGST QILTQGNLNI GAIQTGISNK GYANEKNYNY SITQIDQGSS IQSQGTANLS ANKIQGKAVD IASQQGDIVL NAQQGISLEN GKNLHSTDSA MSAQSSGMLG RNTERNEVWK
bay-D15  QI-NNSNSAT VLNTTGNVDL GTVSVGKQET LKIDDKNGYQ LKQQQDVGSQ IKSAGSLLIN GQNITSKAVD LNSQQGDIQL SAQKDIVLDN GLNQSTVSS- -QSQSKGSFG GKKSSTYDAS

         4561                                                                                                                  4680
 bau-D1  SSDIAVGSIV EGKKVLLDAG NINVRGSNIV SDELTQIQAK DNANIEGAQN QYNSQTNSVV KNSGLMSTGG IGFSLGKKQE TTLKTEQQLT NSA---SQVG SLNGNTNIVA GKTYQQTGST
 nos-D2  SSDVAVGSIV EGKKVLLDAG NINVRGSNIV SDELTQIQAK DNANIEGAQN QYNSQTNSVV KNSGLMSTGG IGFSLGKKQE TTLKTEQQLT NSA---SQVG SLNGNTNIVA GKAYQQTGST
 bau-D3  SSDVAVGSIV EGKKVLLDAG NINVRGSNVV SDELTQIQAK DNANIEGAQN QYNSQTNSVV KNSGLMSTGG IGFSLGRKQE TTLKTEQQLT NSA---SQVG SLNGNTNIVA GKTYQQTGST
 nos-D4  SSDVAVGSIV EGKKVLLDAG NINVRGSNIV SDELTQIQAK DNANIEGAQN QYNSQTNSVV KNSGLMSTGG IGFSLGKKQE TTLKTEQQLT NSV---SQVG SLNGNTNIVA GKTYQQTGST
```

```
 bau-D7   SSDIAVGSIV EGKKVLLDAG NINVRGSNVV SDELTQIQAK DNANIEGAQN QYNSQTNSVV KNSGVMSTGG IGFSLGKKQE TTLKTEQQLT NSA---SQVG SLNGNTNIVA GKTYQQTGST
 bau-D5   SSDIAVGSIV EGKKVLLDAG NINVRGSNVV SDELTQIQAK DNANIEGAQN QYNSQTNSVV KNSGVMSTGG IGFSLGKKQE TTLKTEQQLT NSA---SQVG SLNGNTNIVA GKTYQQTGST
 nos-D8   SSDIAVGSIV EGKKVLLDAG NINVRGSNIV SDELTQIQAK DNANIEGAQN QYNSQTNSVV KNSGLMSTGG IGFSLGKKQE TTLKTEQQLT NSA---SQVG SLNGNTNIVA GKTYQQTGST
 bau-D9   SSDIAVGSIV EGKKVLLDAG NINVRGSNIV SDELTQIQAK DNANIEGAQN QYNSQTNSVV KNSGVMSTGG IGFSLGKKQE TTLKTEQQLT NSA---SQVG SLNGNTNIVA GKAYQQTGST
 bau-D6   SSDIAVGSIV EGKKVLLDAG NINVRGSNIV SDELTQIQAK DNANIEGAQN QYNSQTNSVV KNSGVMSTGG IGFSLGKKQE TTLKTEQQLT NSA---SQVG SLNGNTNIVA GKAYQQTGST
nos-D12   SSDVAVGSIV EGKKVLLDAG NINVRGSNVV SDELTQIQAK DNANIEGAQN QYNSQTNSVV KNSGVMSTGG IGFSLGKKQE TTLKTEQQLT NSA---SQVG SLNGNTNIVA GKTYQQTGST
bau-D10   QANEAIASTI GGKKVILDAN NINIRGSQVV SDDLTQIQAK ENISITTAEN QYSNQFEQTV KKSGFKASLS DGVASVGYGK SSLNTKEDGK STTLTQSVIR SKTGDTTII- AGKDLTTEAA
bau-D11   QANEAIASTI GGKKVILDAN NINIRGSQVV SDDLTQIQAK ENISITTAEN QYSNQFEQTV KKSGFSASLS DGVASVGYGK SSLNTKEDGK STTLTQSVIR SKTGDTTII- AGKDLTTEAA
bau-D14   QANEAIASTI GGKKVILDAN NINMRGSQVV SDDLTQIQAK ENISITTAEN QYSNQFEQTV KKSGFSASLS DGVASVGYGK SSLNTKEDGK STTLTQSVIR SKTGDTTII- AGKDLTTEAA
bau-D12   QASEAIASTI SGKKVLLDAN NVNIRGSQVV ADELTQIQAK ENINIVAAEN HYSNQQEQTV KKSGFKASLS DGVASVGYGK SSLNTKEDGK STTLTQSMIR SKAGDTTII- AGKDLTTEAA
bau-D20   QASEAIASTI SGKKVLLDAN NVNIRGSQVV ADELTQIQAK ENINIVAAEN HYSNQQEQTV KKSGFKASLS DGVASVGYGK SSLNTKEDGK STTLTQSMIR SKAGDTTII- AGKDLTTEAA
bau-D15   TVSQANQIN- AGQNVILNSQ QGNITAIDLK AT------AG NNIQIQAEQG NV--QLLSAL NEKSESSTSS KKNAATYNNR QSGYIDQEVA QTTL------ -KAGNTVDVN AAKNIELQAN
bau-D16   TVSQANQIN- AGQNVILNSQ QGNITAIDLK AT------AG NNIQIQAEQG NV--QLLSAL NEKSESSTSS KKNAATYNNR QSGYIDQEVA QTTL------ -KAGNTVDVN AAKNIELQAN
bau-D21   TVSQANQIN- AGQNVILNSQ QGNITAIDLK AT------AG NNIQIQAEQG NV--QLLSAL NEKSESSTSS KKNAATYNNR QSGYIDQEVA QTTL------ -KAGNTVDVN AAKNIELQAN
bau-D19   TVSQANQIN- AGQNVILNSQ QGNITAIDLK AT------AG NNIQIQAEQG NV--QLLSAL NEKSESSTSS KKNAATYNNR QSGYIDQEVA QTTL------ -KAGNTVDVN AAKNIELQAN
bau-D13   QASEAIASTI SGKKVLLDAN NVNIRGSQVV ADELTQIQAK ENINIVAAEN HYSNQQEQTV KKSGFKASLS DGVASVGYGK SSLNTKEDGK STTLTQSMIR SKAGDTTII- AGKDLTTEAA
cal-D23   QASEAIASTI SGKKVLLDAN NINIRGSQVV SNELTQIQAK ENVNITAAQN QYSNQQEQTV KKSGFKASLS DGVASVGYGK SSLNTKEDGK STTLTQSVVR SQKGDTTII- AGKDLTTEAA
pit-D20   QRDESIASTV EGNKVILDAK NVNIRGSQVV SNELTQIQAK ENVNITAAQN QYSNQQEQTV KKSGFKASLS DGVASVGYGK SSLNTKEDGK STTLTQSVVR SQKGDTTII- AGKDLTTEAA
 pit-D6   QSSEAIASTI SGKKILLDAN NINIRGSQVV SNELTQIQAK ENVNITAAQN QYSNQQEQTV KKSGFKASLS DGVASVGYGK SSLNTKEDGK STTLTQSVVR SQKGDTTII- AGKDLTTEAA
bau-D17   ETVSQANQLN AGQNIIINSQ QGNISATHVL AN------AG NNIQIHAEQG DV-NLLSAID EKSSSATSSK KNAATYNNRQ SGYIDQEVAQ TTLKAGNTV- ------DVN- AAKNIELQAN
nos-D18   TSSSQASQIN AGQNLILHSA HGDINTTNLQ G------QAE NIIQIHADEG NV--HLTSAI NEQSGQDSSH KTSWIKSSSS QSGYVHQQVA QSQL------ -RASNNIDIN AKKDISLQAI
nos-D22   TSSSQASQIN AGQNLILHSA HGDINTTNLQ G------QAE NIIQIHADEG NV--HLTSAI NEQSGQDSSH KTSWIKSSSS QSGYVHQQVA QSQL------ -RAGNNIDIN AKNDISLQAI
 bau-D2   SDQSIANNLK AGGNIVLNTS QGDITATHLK AN------AG ETIQVQAKNG HV--TLNSAL NETSQSTTSS STNFATYNNR QSGYIDQEVA QTQL------ -VAGKNVDIN AAKNIELQAN
bay-D15   SSTSIANQLN AGQNIIINSA QGNISATHLQ AE------AG NNIQIHAQQG NV--TLLSAI DDKRESSTSS KKGVATYNNR QSGYIDQEVA QTTL------ -KAGDTVDIN AAKNIELQAN

          4681                                                                                                                     4800
 bau-D1   VSSQKGDVNI LAQQVNIEAA KEQSTSDYKH EMQQKGLTLA VNVPVVSAVQ SVAESAKQVG QSKNDRVN-A MAAANAGFDA YKAGQALSSL QGALSNAGSL N--GGVEVGV SLTYGEQKNT
 nos-D2   VSSQKGDVNI LAEQVNIEAA KEQSTSDYKH EMQQKGLTLA VNVPVVSAVQ SVVESAKQVG QSKNDRVN-A MAAANAGFDA YKAGQSLGKL QGALSDASKF K--D-MEVGV SLTYGEQKNT
 bau-D3   VSSQKGDVNI LAQQVNIEAA KEQSTSDYKH EMQQKGLTLA VNVPVVSAVQ SVAESAKQVG QSKNDRVN-A MAAANAGFDA YKAGQALSSL QGALSNAGSL N--GGVEVGV SLTYSEQKNT
 nos-D4   VSSQKGDVNI LAEQVNIEAA KEQSTSDYKH EMQQKGLTLA VNVPVVSAVQ SVAESAKQVG QSKNDRVN-A MAAANAGFDA YKAGQALSSL QGALSNAGSL N--GGVEVGV SLTYGEQKNT
 bau-D7   VSSQKGDVNI LAQQVNIEAA KEQSTSDYKH EMQQKGLTLA VNVPVVSAVQ SVAESAKQVG QSKNDRVN-A MAAANAGFDA YKAGQALSSL QGALSNAGSL N--GGVEVGV SLTYGEQKNT
 bau-D5   VSSQKGDVNI LAQQVNIEAA KEQSTSDYKH EMQQKGLTLA VNVPVVSAVQ SVAESAKQVG QSKNDRVN-A MAAANAGFDA YKAGQALSSL QGALSNAGSL N--GGVEVGV SLTYGEQKNT
 nos-D8   ISSQKGDVNI LAEQVNIEAA KEQSTSDYKH EMQQKGLTLA VNVPVVSNVQ SVVESAKQVG QSKNDRVN-A MAAANASFDA YKAGQSLGKL QGALSDASKF K--D-MEVGV SLTYGEQKNT
 bau-D9   VSSQKGDVNI LAQQVNIEAA KEQSTSDYKH EMQQKGLTLA VNVPVVSAVQ SVVESAKQVG QSKNDRVN-A MAAANAGFDA YKAGQSLSQL KDVASAAQMA Q--S-ANVSV SITYGEQKNT
 bau-D6   VSSQKGDVNI LAQQVNIEAA KEQSTSDYKH EMQQKGLTLA VNVPVVSAVQ SVVESAKQVG QSKNDRVN-A MAAANAGFDA YKAGQSLSQL KDVASAAQMA Q--S-ANVSV SITYGEQKNT
nos-D12   VSSQKGDVNI LAEQVNIEAA KEQSTSDYKH EMQQKGLTLA VNVPVVSAVQ SVAESAKQVG QSKNDRVN-A MAAANAGFDA YKAGQALSSL QGALSNAGSL N--GGVEVGV SLTYGEQKNT
bau-D10   ILDAGKDLNL KGANVNLNAG YTTDEQHSEV HSKQSGISVG VTYSPAMAAA AAYKNSMNNG QFSDSAVGQV MAQGEAARKA SMAAMTLVTI QAGSKKTNEV SNTSSTQAVI TQATAKGNLN
bau-D11   ILDAGKDLNL KGANVNLNAG YTTDEQHSEV HSKQSGISVG VTYSPAMAAA AAYKNSMNNG QFSDSAVGQV MAQGEAARKA SMAAMTLVTI QAGSKKTNEV SNTSSTQAVI TQATAKGNLN
bau-D14   ILDAGKDLNL KGANVNLNAG YTTDEQHSEV HSKQSGISVG VTYSPAMAAA AAYKNSMNNG QFSDSAVGQV MAQGEAARKA SMAAMTPVTI QAGSKKTNEV SNTSSTQAVI TQATAKGNLN
bau-D12   ILDAGKDLNL KGANVNLNAG YTTDEQHSEV HSKQSGISVG VTYSPAMAAA AAYKNSMNNG QFSDSAVGQV MAQGEAARKA SMAAMTPVTI QAGSKKTNEV SNTSSTQAVI TQATAKGNLN
bau-D20   ILDAGKDLNL KGANVNLNAG YTTDEQHSEV HSKQSGISVG VTYSPAMAAA AAYKNSMNNG QFSDSAVGQV MAQGEAARKA SMAAMTPVTI QAGSKKTNEV SNTSSTQAVI TQATAKGNLN
bau-D15   DVQAGQSIYV GNTQMQRQAD GTLKAADGSV MPENVTLSTL ETHDQQWDEQ QKGYRGIAKE LVKGLAVG-- MSGLEALAPG LKLDKKITVG ESNNQRTEQI RQTGTSLNAA NIAVGSSGQT
bau-D16   DVQAGQSIYV GNTQMQRQAD GTLKAADGSV MPENVTLSTL ETHDQQWDEQ QKGYRGIAKE LVKGLAVG-- MSGLEALAPG LKLDKKITVG ESNNQRTEQI RQTGTSLNAA NIAVGSSGQT
bau-D21   DVQAGQSIYV GNTQMQRQAD GTLKAADGSV MPENVTLSTL ETHDQQWDEQ QKGYRGIAKE LVKGLAVG-- MSGLEALAPG LKLDKKITVG ESNSQRTEQI RQTGTSLNAA NIAVGSSGQT
bau-D19   DVQAGQSIYV GNTQMQRQAD GTLKAADGSV MPENVTLSTL ETHDQQWDEQ QKGYRGIAKE LVKGLAVG-- MSGLEALAPG LKLNKKITVG ESNNQRTEQI RQTGTSLNAA NIAVGSSGQT
bau-D13   ILDAGKDLNL KGANVNLNAG YTTEEQHSEV HSKQSGISVG VTYSPAMAAA AAYKNSMNNG QFSDSAVGQV MAQGEAARKA SMAAMTPVTI QAGSKKTNEV SNTSSTQAVI TQATAKGNLN
cal-D23   ILEAGKDLNL KGANVNLNAA YATGEQHSEV HSKQSGVSVG VTYSPAMAAA SAYKNSMNNG QFSDSAVGQV MAQGEAVRKA SMAAMTPVTI QAGSNKTNQV SNTSSTQAVV TQATAKGNLN
pit-D20   ILDAGKDLNL KGANVNLNSA YATDEQHSEV HSKQSGISVG VTYSPAMAAA SAYKNSMNNG QFSDSAVGQV MAQGEAVRKA SMAAMTPVTI QAGSNKTNQV SNTSSTQAVV TQATAKGNLN
 pit-D6   ILDAGKDLNL KGANVNLNSA YATDEQHSEV HSKQSGISVG VTYSPAMAAA SAYKNSMNNG QFSDSAVGQV MAQGEAVRKA SMAAMTPVTI QAGSNKINQV SNTSSTQAVV TQATAKGNLN
bau-D17   DVQAGQSIYV GNTQMQRQAD GTLKAADGSV MPENVTLSTL ETHDQQWDEQ QKGYRGIAKE LVKGLAVG-- LSGLEALAPG LKLDKKITVG ESNSQRTEQI RQTGTSLNAA SIAVGSSGQT
nos-D18   DADAGKSIYV GNTLMQRQAD GTLKSADGSL MPKNVTLSTL ETHDQQWEEQ RKGYRGIAKE LVKGLAIG-- LPGLQAAAPG LDLQKKLTVG ESNSHRTEET HQAGTSLSAE NIAVGSSGQT
nos-D22   DAEAGKSIYV GNTLMQRQAD GTLKAADGSV MPKNVLLSTL ETHNQQWEEQ QKGYRGIAKE LVKGLAIG-- LPGLQAAAPG LDLQKKLTVG ESNSHRTEET HQSGTSLSAE NIAVGSSGQT
 bau-D2   DVQAGNSIYV GNTLMQRQVD GTLKAADGSV MPENVKLSTL ETHDQQWDEQ QKGYRGVVKE LMKVTEVG-- LAGVQSLFPG VKVDTKLTIG ESNSKREEQI KQTGTNLTAN DVYVGSSGQT
bay-D15   DVQAGNSIYV GNTLMQRQAD GSLKAADGSL MPENVTLSTL ATKDEQWDEQ QKGYRGIVKD IVKATAVG-- LAGIEALAPG LKAP-KITVS ESTGKRTEQI SQTGSSLKAD NVYVGSSGQT

          4801                                                                                                                     4920
 bau-D1   ETSHSQSTTA SQSQVNAGGT TTIVATGAGD QSNINIVGSD VLGQQGTRLA ADNNVNIKAA ----EQNHIE ESKNESAGWN AGVTVS---N KTGFGVTAGG NLGKGKGNGT -DTSYVNSHV
 nos-D2   ETSHSQSTTA SQSQVNAGGR TSIVATGAGN QSNINIVGSD VLGQQGTRLA ADNNVNIKAA ----EQNHIE ESKNESAGWN AGVTVS---N KTGFGVTAGG NLGKGKGNGT -DTSYVNSHV
 bau-D3   ETSQSQSTTA SQSQVNAGGT TTIVATGAGD QSNINIVGSD VLGQQGTRLA ADNNVNIKAA ----EQNHIE ESKNESAGWN AGVTVS---N KTGFGVTAGG NLGKGKGNGT -DTSYVNSHV
 nos-D4   ETSHSQSTTA SQSQVNAGGR TSIVATGAGD QSNINIVGSD VLGQQGTRLA ADNNVNIKAA ----EQNHIE ESKNESAGWN AGVTVS---N QTGFGVTAGG NLGKGKGNGT -DTSYVNSHV
 bau-D7   ETSHSQSTTA SQSQVNAGGR TSIVATGAGD QSNINIVGSD VLGQQGTRLA ADNNVNIKAA ----EQNHIE ESKNESAGWN AGVTVN---N KTGFGVTAGG NLGKGKGNGT -DTSYVNSHV
 bau-D5   ETSHSQSTTA SQSQVNAGGR TSIVATGAGD QSNINIVGSD VLGQQGTRLA ADNNVNIKAA ----EQNHVE ESKNESAGWN AGVTVS---N KTGFGVTAGG NLGKGKGNGT -DTSYVNSHV
 nos-D8   ETSHSQSTTA SQSQVNAGGT TTIVATGAGD QSNINIVGSD ILGQQGTRLA ADNNVNIKAA ----EQNHIE ESKNESAGWN AGVTVS---N KTGFGVTAGG NLGKGKGNGT -DTSYVNSHV
 bau-D9   ETSHSQSTTA SQSQVNAGGR TSIVATGAGD QSNINIIGSD VLGQQGTRLA ADNNVNIKAA ----EQNHLE ESKNESAGWN AGVAVSYGSN GLAFGVTAGG NVGKGKGDGS -ETSYLTSHV
 bau-D6   ETSHSQSTTA SQSQVNAGGR TSIVATGAGD QSNINIIGSD VLGQQGTRLA ADNNVNIKAA ----EQNHLE ESKNESAGWN AGVAVSYGSN GLAFGVTAGG NVGKGKGDGS -ETSYLTSHV
nos-D12   ETSHSQSTTA SQSQVNAGGR TSIVATGAGD QSNINIVGSD VLGQQGTRLA ADNNVNIKAA ----EQNHIE ESKNESAGWN AGVTVS---N QTGFGVTAGG NLGKGKSNGT -DTSYVNSHV
bau-D10   IIATEGSINS QGAQLSAEGD ALLHAKENIN LNVAQSHSEQ TVDRKQSGFS IDNRDWAAPA GTFKNKNQGD GRNTQTTVTQ LSVGGKTTLQ TGQGDINIVG SSVASKGDVN L-HAARDINI
bau-D11   IIATEGSINS QGAQLSAEGD ALLHAKENIN LNVAQSHSEQ TVDRKQSGFS IDNRDWAAPA GTFKNKNQGD GRNTQTTGTQ LSVGGKTTLQ TEQGDINVVG SSVASKGDVN L-HAARDINI
bau-D14   IIATEGSINS QGAQLSAEGD ALLHAKENIN LNVAQSHSEQ TVDRKQSGFS IDNRDWAAPA GTFKNKNQGD GRNTQTTGTQ LSVGGKTTLQ TGQGDINIVG SSVASKGDVN L-YAARDINI
bau-D12   IIATEGSINS QGAQLSAEGD ALLHAKENIN LNVAQSHSEQ TVDRKQSGFS IDNRDWAAPA GTFKNKNQGD GRSTQTTGTQ LSVGGKTTLQ TRQGDINVVG SSVASKGDVN L-HAARDINI
bau-D20   IIATEGSINS QGAQLSAEG- --LHAKENIN LNVAQSHSEQ TVDRKQSGFS IDNRDWAAPA GTFKNKNQGD GRSTQTTGTQ LSVGGKTTLQ TGQGDINVVG SSVASKGDVN L-HAARDINI
bau-D15   TL-TSADLTA KNVALSGQK- VTLNAAEEQN IE-SSSHSTE TIEGL--GVK L-NKDSIRLG GFVSEKDKNT LTVTETTHKA GSINTDNLSI NGGKGVDILG QNIKATGDTT IDHGRGELNI
bau-D16   TL-TSADLTA KNVALSGQK- VTLNAAEEQN IE-SSSHSTE TIEGL--GVK L-NKDSIRLG GFVSEKDKNT LTVTETTHKA GSINTDNLSI NGGKGVDILG QNIKATGDTT IDHGRGELNI
bau-D21   TL-TSADLTA KNVALSGQK- VTLNAAEEQN IG-SSSHSTE TIEGL--GVK L-NKDSIRLG GFVSEKDKNT LMVTETTRKA GSINTDNLSI NGAKGVDILG QNIKATGDTT IDHGRGELNI
bau-D19   TL-TSADLTA KNVALSGQK- VTLNAAEEQN IE-SSSHSTE TIEGL--GVK L-NKDSIRLG GFVSEKDKNT LTVTETTHKA GSINTDNLSI NGGKGVDILG QNIKATGDTT IDHGRGELNI
bau-D13   IIATEGSINS QGAQLSAEGD ALLHAKENIN LNVAQSHSEQ TVDRKQSGFS IDNRDWAAPA GTFKNKNQGD GRSTQTTGTQ LSVGGKTTLQ TGQGDINVVG SSVASKGDVN LHAAR-DINI
cal-D23   IIATEGSINS QGAELSAEGD ALLHAKENIN LGVAESHSEQ TADRKQSGFS IDNRDWAAPA GTFKNKNQGD GRSTQTTGTQ LSVGGKTTLQ SGQGDINIIG SSVASKGDVN LNAAR-DINI
pit-D20   IIATEGSINS QGAELSAEGN ALLHAKENIN LGIAESHSEQ TADRKQSGFS IDNRDWAAPA GTFKNKNQGD GRSTQTTGTQ LSVGGKTTLQ TGQGDINIIG SSVASKGYVN LNAAR-DINI
 pit-D6   IIATEGSINS QGAELSAEGD ALLHAKENIN LGIAESHSEQ TADRKQSGFS IDNRDWAAPA GTFKNKNQGD GRSTQTTGTQ LSVGGKTTLQ TGEGDINIIG SSVASKGDVN LNAAR-DINI
bau-D17   TL-TSADLTA KNIALSGQ-K VTLNAAEEQN I---ESSSHS TETIEGLGVK L-NKDSIRLG GFVSEKDKNT LTVTETTRKA GSINTDNLSI NGAKGVDILG QNIKATGDTT IDHGRGELNI
nos-D18   TL-TSADLTA KNVALSGQK- VTLNAAEEQS IE-SSSHTIE TIEGL--GVK L-NKDSIRLG GFVSEKDKNT LTTTETTHKA GTINTDNLSI KGTEGVDVLG QNIKATGDTT IDHGRGELNI
nos-D22   TL-TSADLTA KNVALSGQK- VILNAAEEQS IE-SSSHTIE TIEGL--GVK L-NKDSIRLG GFVSEKDKNT LTTTETTHKA GTINTDNLSI KGTEGVDVLG QNIKATGDTT IDHGRGELNI
 bau-D2   TL-TSSDITA KNTILSGQK- VTLNAAEEQN IS-ATSHSKE TIEGL--GVK L-NKDSVRLG GFVSEDTTQS TKTTETTHKA GSIQTENLKI QGAEGVDILG QNIKATGDTV LDHGRGDLNI
bay-D15   TL-TSTDITA KNTILLGQK- VTLNAAEEQK IT-VESTSKE TVQGL--GAK L-NKDSIRLG GFVLEDASQS SKTTETTHKL GSINTENLKI QGVEGVDILG QNITATGDTL IDHGRGDLNV

          4921                                                                                                                     5040
 bau-D1   GSKDSLTTIT SGKTTN---- ---------- ---------- ---------- ---IIGGQVQ GKGVQIEADN LNVESLQDKA TYKSKQQNMS AQVSVGFSGG NVSGSFSKSN VDANYASVNE
 nos-D2   GSKDSLTTIT SGKTTN---- ---------- ---------- ---------- ---IIGGQVQ GKGVQIEADN LNVESLQDKA TYKSKQQNMS AQVSVGFSGG NVSGSFSKSN VDANYASVNE
 bau-D3   GSKDSLTTIT SGKTTN---- ---------- ---------- ---------- ---IIGGQVQ GKGVQIEADN LNVESLQDKA TYKSKQQNMS AQVSVGFSGG NVSGSFSKSN VDANYASVNE
 nos-D4   GSKDSLTTIT SGKTTN---- ---------- ---------- ---------- ---IIGGQVQ GKGVQIEADN LNVESLQDKA TYKSKQQNMS AQVSVGFSGG NVSGSFSKSN VDANYASVNE
 bau-D7   GSKDSLTTIT SGKTTN---- ---------- ---------- ---------- ---IIGGQVQ GKGVQIEADN LNVESLQDKA TYKSKQQNMS AQVSVGFSGG NVSGSFSKSN VDANYASVNE
 bau-D5   GSKDSLTTIT SGKTTN---- ---------- ---------- ---------- ---IIGGQVQ GKGVQIEADN LNVESLQDKA TYKSKQQNMS AQVSVGFSGG NVSGSFSKSN VDANYASVNE
 nos-D8   GSKDSLTTIT SGKTTN---- ---------- ---------- ---------- ---IIGGQVQ GKGVQIEADN LNVESLQDKA TYKSKQQNMS AQVSVGVSGG NVSGSFSKSN VDANYASVNE
 bau-D9   GSKDSLTTIS SGNATN---- ---------- ---------- ---------- ---IIGGQVQ GKGVQIEADN LNVESLQNKA DYKSKQQNVS GQATVGY-GA SVSGSFSKSD VKAHYASVQD
 bau-D6   GSKDSLTTIS SGNATN---- ---------- ---------- ---------- ---IIGGQVQ GKGVQIEADN LNVESLQNKA DYKSKQQNVS GQATVGY-GA SVSGSFSKSD VKAYYASVQD
nos-D12   GSKDSLTTIT SGKTTN---- ---------- ---------- ---------- ---IIGGQVQ GKGVQIEADN LNVESLQDKV TYKSKQQSMS AQVSVGFSGG SVSGSFSNSN VDANYASVNE
bau-D10   KSSQNSQSQS EQSSNKGIGS AQISDTEQFY GYMSGKSQST SNSVEQQRSQ VGSLEGNVNI QAGRQYTQQV ADVIAAKDLN IKAQDIQVLE GHNTGSSSEN SKDLKVGQFS RVSSPLIDLV
bau-D11   KSSQNSQSQS EQSSNKGIGS AQISDTEQFY GYMSGKSQST SNSVEQQRSQ VGSLEGNVNI QAGRQYTQQV ADVIAAKDLN IKAQDIQVLE GHNTGSSSEN SKDLKVGQFS RVSSPLIDLV
bau-D14   KSSQNSQSQS EQSSNKGIGS AQISDTEQFY GYMSGKSQST SNSVEQQRSQ VGSLEGNVNI QAGRQYTQQV ADVIAAKDLN IKAQDIQVLE GHNTGSSSEN SKDLKVGQFS RVSSPLIDLV
bau-D12   KSSQNSQSQS EQSSNKGIGS AQISDTEQFY GYMSGKSQST SNSVEQQRSQ VGSLEGNVNI QAGRQYTQQV ADVIAAKDLN IKAQDIQVLE GHNTGSSSEN SKDLKVGQFS RVSSPLIDLV
bau-D20   KSSQNSQSQS EQSSNKGIGS AQISDTEQFY GYMSGKSQST SNSVEQQRSQ VGSLEGNVNI QAGRQYTQQV ADVIAAKDLN IKAQDIQVLE GHNTGSSSEN SKDLKVGQFS RVSSPLIDLV
bau-D15   GGYENKTTTE EKTKNE---- ---------- ---------- ---------- ----KTSVEV GVRNAYLDAA LAVGAVKDAA SALKDAK--- ----DAYSQA QRDYAAGKLT KEA--LDDSK
bau-D16   GGYENKTTTE EKTKNE---- ---------- ---------- ---------- ----KTSVEV GVRNAYLDAA LAVGAVKDAA SALKDAK--- ----DAYSQA QRDYAAGKLT KEA--LDDSK
bau-D21   GGYENKTTTE EKTKNE---- ---------- ---------- ---------- ----KTSVEV GVRNAYLDAA LAVGAVKDAA SALKDAK--- ----DAYSQA QRDYAAGKLT KEA--LDDSK
bau-D19   GGYENKTTTE EKTKNE---- ---------- ---------- ---------- ----KTSVEV GVRNAYLDAA LAVGAVKDAA SALKDAK--- ----DAYSQA QRDYAAGKLT KEA--LDDSK
bau-D13   KSSQNSQSQS EQSSNKGIGS AQISDTEQFY GYMSGKSQST SNSVEQQRSQ VGSLEGNVNI QAGRQYTQQV ADVIAAKDLN IKAQDIQVLE GHNTGSSSEN SKDLKVGQFS RVSSPLIDLV
cal-D23   KSSQNSQSQS EQSSNKGIGS AQISDTEQFY GYMNGQSQSN SHGIEQQRSQ VGSLEGNVNI QAGRQYTQQV ADVVAAKDLN IKAQDIQVLE GHDTGSSSEN SKELKIGQFS RVSSPLIDLL
pit-D20   KSSQNSQSQS EQSSNKGIGS AQISDTEQFY GYMNGQSQSN SKGVEQQRSQ VGSLEGNVNI QAGRQYTQQV ADVVAAKDLN INAQDIQVLE GHDTGSSSEN SKDLKIGQFS RVSSPLIDLL
 pit-D6   KSSQNSQSQS EQSSNKGIGS AQISDTEQFY GYMNGQSQSN SKGVEQHRSQ VGSLEGNVNI QAGRQYTQQV ADVVAAKDLN IKAQDIQVLE GHDTGSSSEN SKDLKIGQFS RVSSPLIDLL
bau-D17   GGYENKTTTE EKTKNE---- ---------- ---------- ---------- ----KTSVEV GVRNAYLDAA LAVGAVKDAA SALKDAK--- ----DAYSQA QRDYAAGKLT KEA--LDDSK
nos-D18   GGYENKTTTE EKTKNE---- ---------- ---------- ---------- ----KISTEV GVRNAYLDAA LAVVAVKDAA SALKDAK--- ----NAYSQA QRDYAAGKLT KEA--LDDSK
nos-D22   GGYENKTTTE EKTKNE---- ---------- ---------- ---------- ----KISTEV GVRNAYLDAA LAVVVVKDAA SALKDAK--- ----NAYSQA QRDYAAGKLT KEA--LDDSK
 bau-D2   GGYENKTTVE DKTHKE---- ---------- ---------- ---------- ----TISTEV GVRNAYLDAA LAVGAVKDAA EVVKQAK--- ----DQYSQA QRDYASGKIT KEA--LDDTK
bay-D15   GGYENKTTVE DKTHVE---- ---------- ---------- ---------- ----TISTEV GVRNAYLDAA LALGAVKDAA EAVKQAK--- ----DQYSQA QRDYASGKIT KEA--LDDSK
```

```
         5041                                                                                                            5160
 bau-D1  QSGVFAGDDG YQIKVNKNTD LKGAIITSTQ TAENLKKNSL DTGTLTSSNI QNVTEYDAKG ISVGGGFNAG KSGTAGNKEP GTVLSTPNKI DQHASTTVGV SKSVGFGLDS DKDSSVTKSG
 nos-D2  QSGVFAGDDG YQIKVNKNTD LKGAIITSTQ TAENLKKNSL DTGTLTSSNI QNVTEYDAKG VSVGGGFNAG KSGTAGNKEP GTVLSTPNKI DQHASTTVGV SKSVGFGLDS DKDSSMTKSG
 bau-D3  QSGVFAGDDG YQIKVNKNTD LKGAIITSTQ TAENLKKNSL DTGTLTSSNI QNVTEYDAKG ISVGGGFNAG KSGTAGNKEP GTVLSTPNKI DQHASTTVGV SKSVGFGLDS DKDSSVTKSG
 nos-D4  QSGVFTGEDG YQIKVNKNTD LKGAIITSTQ TAENLKKNSL DTGTLTSSNI QNVTEYDAKG ISVGGGFNAG KSGTAGNKEP GTVLSTPNKI DQHASTTVGV SKSVGFGLDS DKDSSVTKSG
 bau-D7  QSGVFAGDDG YQIKVNKNTD LKGAIITSTQ TAENLKKNSL DTGTLTSSNI QNVTEYDAKG ISVGGGFNAG KSGTAGNKEP GTVLSTPNKI DQHASTTVGV SKSVGFGLDS DKDSSVTKSG
 bau-D5  QSGVFAGDDG YQIKVNKNTD LKGAIITSTQ TAENLKKNSL DTGTLTSSNI QNVTEYDAKG ISVGGGFNAG KSGTAGNKEP GTVLSTPNKI DQHASTTVGV SKSVGFGLDG DKDSSVTKSG
 nos-D8  QSGVFAGDDG YQIKVNKNTD LKGAIITSTQ TAENLKKNSL DTGTLTSSNI QNVTEYDAKG ISVGGGFNAG KSGTAGNKEP GTVLSTPNKI DQHASTTVGV SKSVGFGLDS DKDSSVTKSG
 bau-D9  QAGIYAGDDG YQIKVNKNTD LKGAIITSTQ TAENLKKNSL DTGTLTSSNI QNVTEYDAKG ISVDGGFNAG KSGTAGNKEP GTVLSTPNKI DQHASTTVGV SKSVGFGLDG DKGSSVTKSG
 bau-D6  QAGIYAGDDG YQIKVNKNTD LKGAIITSTQ TAENLKKNSL DTGTLTSSNI QNVTEYDAKG ISVDGGFNAG KSGTAGNKEP GTVLSTPNKI DQHASTTVGV SKSVGFGLDG DKGSSVTKSG
nos-D12  QTGVFAGDDG YQIKVNKNTD LKGAIITSTQ TAENLKKNSL DTGTLTSSNI QNVTEYDAKG ISVGGGFNAG KSGTAGNKEP DTVLSTPNKI DQHASTTVGV SKSVGFGLDG DKDSSVTKSG
bau-D10  NAADKATKSQ ADDRTQALQA VAAGAQGYQT YSDIKGGALF KAESGIGFST SKNQQNSSYA TSQQNNLKAG GNVNLTSTHG DIHLQN-TQV KAEDKINLDS AKNIVLESGQ SQNKADGKNS
bau-D11  NAADKATKSQ ADDRTQALQA VAAGAQGYQT YSDIKGGALF KAESGIGFST SKNQQNSSYA TSQQNNLKAG GNVNLTSTHG DIHLQN-TQV KAEDKINLDS AKNIVLESGQ SQNKADGKNS
bau-D14  NAADKATKSQ ADDRTQALQA IAAGAQGYQT YSDIKGGALF KAESGIGFST SKNQQNSSYA TSQQNNLKAG GNVNLTSTHG DIHLQN-TQV KAEDKINLDS AKNIVLESGQ SQNKADGKNS
bau-D12  NAADKATKSQ ADDRTQALQA VAAGAQGYQT YSDIKGGALF KAESGIGFST SKNQQNSSYA TSQQNNLKAG GNVNLTSTHG DIHLQN-TQV KAEDKINLDS AKNIVLESGQ SQNKADGKNS
bau-D20  NAADKATKSQ ADDRTQALQA VAAGAQGYQT YSDIKGGALF KAESGIGFST SKNQQNSSYA TSQQNNLKAG GNVNLTSTHG DIHLQN-TQV KAEDKINLDS AKNIVLESGQ SQNKADGKNS
bau-D15  ANVAMATANL ASAQIAVGAS AAAAAASSAT YGFTIGANGE RIET----TT NNNQTQGHW- --QGSNLELN -NLTLKSEGQ NTNIQG-SRL TATGTTTFNG TKDLNVTAGT EHSTQESSSK
bau-D16  ANVAMATANL ASAQIAVGAS AAAAAASSAT YGFTIGANGE RIET----TT NNNQTQGHW- --QGSNLELN -NLTLKSEGQ NTNIQG-SRL TATGTTTFNG TKDLNVTAGT EHSTQESSSK
bau-D21  ANVAMATANL ASAQIAVGAS AAAAAASSAT YGFTIGANGE RIET----TT NNNQTQGHW- --QGSNLELN -NLTLKSEGQ NANIQG-SRL TATGTTTFNG TKDLNVTAGT EHSTQESSSK
bau-D19  ANVAMATANL ASAQIAVGAS AAAAAASSAT YGFTIGANGE RIET----TT NSNQTQGHW- --QGSNLELN -NLTLKSEGQ NANIQG-SRL TATGTTTFNG TKDLNVTAGT EHSTQESSSK
bau-D13  NAADKATKSQ ADDRTQALQA IVAGAQGYQT YSDIKGGALF KAESGIGFST SKNQQNSSYA TSQQNNLKAG GNVNLTSTHG DIHLQN-TQV KAEDKINLDS AKNIILESGQ SQNKADGKNS
cal-D23  NAADKAKNSK ADDRTQALQA VAAGAQGYQT YSDIKGGALF KAESGIGFST SKNQQNSSYA TSQQNNLKAG GNVNLTSTHG DIHLQN-TQV KAEDKINLDS SKNIILESGQ SQNKADGKNS
pit-D20  NAADKAKNSK ADDRTQALQA VAAGAQGYQT YSDIKGGALF KAESGIGFST NKNQQNSSYA TSQQNNLKAG GNVNLTSTHG DIHLQN-TQV KAEDKINLDS SKNIILESGQ SQNKADGKNS
 pit-D6  NAADKAKNSK ADDRTQALQA VAAGAQGYQT YSDIKGGALF KAESGIGFST NKNQQNSNYA ISQQNNLKAG GNVNLTSTHG DIHLQN-TQV KAEDKINLDS SKNIILESGQ SQNKADGKNS
bau-D17  ANVAMATANL ASAQIAVGAS AAAAAASSAT YGFTIGANGE RIET----TT NSNQTQGHW- --QGSNLELN -NLTLKSEGQ NANIQG-SRL TATGTTTFNG TKDLNVTAGT EHSTQESSSK
nos-D18  ANVAMATANL ASAQIAVGAS AAAAAASSAT YGFTIGANGE HIET----TT TTTTTQGQW- --QGSNLDLN -NLTLKSEDQ NANIQG-SRL SATGTTIFNG TKDLNITAGI EHSSRESSSK
nos-D22  ANVAMATANL ASAQIAVAAS VAAAAANSGT YGFTIGANGE HIET----TA TTTTTQGQW- --QGSHLDLN -NLTLKSEDQ NANIQG-SRL SVAGTTTFNG TKDLNITAGI EHGSKESSSK
 bau-D2  ANVAMATANL ANAQIAVGSA AATAAASSAT YGFTIGANGE RIET----TT TTNTTQGKW- --QGSQLDLN -NLTLKSENQ DVNLQG-SRL TATGTTTFDG TKDFNISAGK EHGQQETSSK
bay-D15  ANVAMATANL ANAQIAVGSA AATAAASSAT YGFTIGANGE RIET----TN TTNTTQGKW- --QGSTLELN -NLTLKSENQ DVNVQG-SRL TATGTTSFDG TKDLNVTAGT EHSQQETSSK

         5161                                                                                                            5280
 bau-D1  INTQNITIRD EQGQQALTGK TAGQIKSEIL TSVTTDTARE NSGALQNNFD KDKVQSEINL QMDVTKNFDA NRQEAGITVD NIVKALEVTG QAI-EGAKQT LAIAKQAQQE IQKLSPEDQ-
 nos-D2  VNTQNITIRE EQGQQALTGK TAGQIKSEIL TSVTTDTARE NSGALQNNFD KDRVLKKEINT QVSVTQKFGE NAPKAVADY- ---------- -----AQKQA LILRAQGNEA ------EAKK
 bau-D3  INTQNITIRD EQGQQALTGK TAGQIKSEIL TSVTTDTARE NSGALQNNFD KDKVLKEINT QVSVTQKFGE NAPKAVADY- ---------- -----AQKQA LILRAQGNEA ------EAKK
 nos-D4  VNTQNITIRD EQGQQALIGK TAGKIKSEIL TSVTTDTARE NSGALQNNFD KDRVQSEINL QMDVTKNFDA NRQEAKAEIN KKIDDAKKEN QSIIDKQKQG LGLSKQEQDQ LNAYNDKVEN
 bau-D7  INTQNITIRD EQGQQALTGK TAGQIKSEIL TSVTTDTARE NSGALQSNFD KDRVQSEINL QMDVTKNFDA NRQEAKAEIN KKIDDAKKEN QSIIDKQKQG LDLSKQEQDQ LKAYNNKVEN
 bau-D5  INTQNITIRD EQGQQALTGK TAGQIKSEIL TSVTTDTARE NSGALQNNFD KDKVQSEINL QMDVTKNFDA NRQEAKAEIN KKIDDAKKEN QSIIDKQKQG LDLSKQEQDQ LKAYNNKVEN
 nos-D8  VNTQNITVRD EQGQQALTGK TAGQIKSEIL TSVTTDTARE NSGALQNNFD KDRVQSEINL QMDVTKNFDA NRQEAKAEIN KKIDDAKKEN QSIIDKQKQG LGLSKQEQDQ LNAYNDKVEN
 bau-D9  INTQNITIRD EQGQQALTGK TAGEIKSEIL TSVTTDTARE NSGALQNNFD KDKVQSEINL QMDVTKNFDA NRQEAKAEIN KKIDDAKKEN QSIIDKQKQG LGLSKQEQDQ LNAYNDKVEN
 bau-D6  INTQNITIRD EQGQQALTGK TAGEIKSEIL TSVTTDTARE NSGALQNNFD KDKVQSEINL QMDVTKNFDA NRQEAKAEIN KKIDDAKKEN QSIIDKQKQG LGLSKQEQDQ LNAYNDKVEN
nos-D12  INTQNITIRD EQGQQALTGK TAGQIKSEIL TSVTTDTARE NSGALQNNFD KDRVQSEINL QMDVTKNFDA NRQEAKAEIN KKIDDAKKEN QSIIDKQKQG LGLSKQEQDQ LNAYNDKVEN
bau-D10  NAGLSVGVGA SVGAQTGVYI YGEAGFGKGS NHLDSNTHNQ TTLDAKNISL TSKGDTTLRG AQAKADRIDA N-VGGQLKVE SLQDTVEQNT KQTGAGGRLQ ASFGTAWQAS GNFSSSKASG
bau-D11  NAGLSVGVGA SVGAQTGVYI YGEAGFGKGS NHLDSNTHNQ TTLDAKNISL TSKGDTTLRG AQAKADRIDA N-VGGQLKVE SLQDTVEQNT KQTGAGGRLQ ASLGSAWQAS GNFSSSKASG
bau-D14  NAGLSVGVGA SVGAQTGVYI YGEAGFGKGS NHLDSNTHNQ TTLDAKNISL TSKGDTTLRG AQAKADRIDA N-VGGQLKVE SLQDTVEQNT KQTGAGGRLQ ASLGSAWQAS GNFSSSKASG
bau-D12  NAGLSVGVGA SVGAQTGVYI YGEAGFGKGS NHLDSNTHNQ TTLDAKNISL TSKGDTTLRG AQAKADRIDA N-VGGQLKVE SLQDTVEQNT KQTGAGGRLQ ASLGSAWQAS GNFSSSKASG
bau-D20  NAGLSVGVGA SVGAQTGVYI YGEAGFGKGS NHLDSNTHNQ TTLDAKNISL TSKGDTTLRG AQAKADRIDA N-VGGQLKVE SLQDTVEQNT KQTGAGGRLQ ASLGSAWQAS GNFSSSKASG
bau-D15  TNSQSISYSS GGGGSASIGK QTSQSQSESL THVNSEVALN RTEGQLNKLN IQGGEVSI-- ----ADRGNL Q-VN-QIHVE SLQDTAKSSN SSKGGSIGAG FGSSGISNVS ASYNQSKGSS
bau-D16  TNSQSISYSS GGGGSASIGK QTSQSQSESL THVNSEVALN RTEGQLNKLN IQGGEVSI-- ----ADRGNL Q-VN-QIHVE SLQDTAKSSN SSKGGSIGAG FGSSGISNVS ASYNQSKGSS
bau-D21  TNSQSISYSS GGGGSASIGK QTSQSQSESL THVNSEVALN RTEGQLNKLN IQGGEVSI-- ----ADRGNL Q-VN-QIHVE SLQDTAKSSN SSKGGSIGAG FGSSGISNVS ASYNQSKGSS
bau-D19  TNSQSISYSS GGGGSASIGK QTSQSQSESL THVNSEVALN RTEGQLNKLN IQGGEVSI-- ----ADRGNL Q-VN-QIHVE SLQDTAKSSN SSKGGSIGAG FGSSGISNVS ASYNQSKGSS
bau-D13  NAGLSVGVGA SVGAQTGVYI YGEAGFGKGS NHLDSNTHSQ TTLDAKDINL VSQGDTTLRG AQAKADRIDA N-VGGQLKVE SLQDTVEQNT KQTGAGGRLQ ASLGSAWQAS GNFSSSKASG
cal-D23  NAGLSVGVGV SVGAQTGAYI YGEAGFGKGS NHLDSNTHSQ TTLDAKNISL TSKGDTTLRG AQAKADRIDA N-VGGQLKVE SLQDTVEQNT KQTGAGGRLQ ASFGTAWQAS GNFSSSKASG
pit-D20  NAGLSVGVGA SVGAQTGVYI YGEAGFGKGS NHLDSNTHSQ TTLDAKNISL TSKGDTTLRG AQAKADRIDA N-VGGQLKVE SLQDTVEQNT KQTGAGGRLQ ASFGTAWQAS GNFNSSKASG
 pit-D6  NAGLSVGVGV SVGAQTGAYI YGEAGFGKGS NHLDSNTHSQ TTLDAKNISL TSKGDTTLRG AQAKADRIDA N-VGGQLKVE SLQDTVEQNT KQTGAGGRLQ ASFGTAWQAS GNFSSSKASG
bau-D17  TNSQSISYSS GGGGSASIGK QTSQSQSESL THVNSEVALN RTEGQLNKLN IQGGEVSI-- ----ADRGNL Q-VN-QIHVE SLQDTAKSSN SSKGGSIGAG FGSSGISNVS ASYNQSKGSS
nos-D18  TNNQSISYSS GGSGSASIGK QTSQSQSESL THVNSEVALN RTEGQLNKLN IQGGEVSI-- ----ADRGNL Q-FN-QILVE SLQDTAKSSN SSKGGSIGAG FGSSGISNVS ASYNQSKGSS
nos-D22  TTSQSVSYTY GGGGGASIGK QTSQSQSESL THVNSEVALN RTEGQLNKLN IQGGEVSI-- ----ADRGNL Q-VN-QILVE SLQDTAKSSN SSKGGSIGAG FGSSGISNVS ASYNQSKGSS
 bau-D2  TNNQSVSYTY GGGGSASVGK QTSKSHAESL THVNSEVALN RTEGQLNKLN IQGGEVSI-- ----ADRGNL Q-VN-QIHVE SLQDTASGNS SSKGGSVGVG IGSGGLSNVT ASYNQAKGQN
bay-D15  TNSQSVSYTY GGGGSASIGK QTSTSESKSL TYVNSEVTLN KTEGRLNSLN IKGGEVSI-- ----QDRGNL Q-VN-NIHVE SLQDTASSSN SSRGGSIGGG YGSGS-KNIT ASYNQSKGSS

         5281                                                                                                            5400
 bau-D1  -----VIFEN FLTELAQSQR TDRTDTY--- ---YPLVLAA GEGLAMAAAA CARSPACASA TVNVLGAVGV AILNEQSDDK NKKTTPKPLP STSGNSATGM PPNKDDNERD FKYDNKQIGK
 nos-D2  WDEGGIYRVA MHTALGALGT GTLEGAV--- ---TTGTVAA SAPKINDFQN SVAQ-ALTQT GISEKTAEAV ASGVTSLTLA AAGVVAGLNT GSTATAVNVD ANNRQLHVDE AKVLKKLTEG
 bau-D3  WDEGGIYRVA MHTALGALGT GTLEGAI--- ---TTGTVAA SAPKINDFQN SVAQ-ALTHT GMSEKAAEAV ASGVTSLTLA GVGVASGLNT GSTATAVNVD ANNRQLHVDE AKVLKKLTEG
 nos-D4  YQRLGVLLDS ISTGLSATTS SGLGIAT--- ---ATLSPAA SYQIGQYFKE QASNNPNGQL TSGQEAAHIL AHTVLGAAVA AAGGNDAMTA GLSAGGAEAA APILSSFL-Y GKDAKDLTAD
 bau-D7  YQRLGVLLDS ISTGLSAPTS SGLGIAT--- ---ATLSPAA SYKIGQYFKE QANNNANGQL TSGQEAAHIL AHTVLGAAVA AAGGNDALTA GLSAGGAEAA APILSSFL-Y GKDAKDLTAD
 bau-D5  YQRLGVLLDS ISTGLSAPTS SGLGIAT--- ---ATLSPAA SYKIGQYFKE QASNNANGQL TSGQEAAHIL AHTVLGAAVA AAGGNNALTA GLSAGGAEAA APILSSFL-Y GRDAKDLTVD
 nos-D8  YQKLGVLLDS ISTGLSAPTS SGLGIAT--- ---ATLSPAA SYKIGQYFKE QANNNANGQL TSGQEAAHIL AHTVLGAAVA AAGGNDALTA GLSAGGAEAA APILSSFL-Y GKAAKDLTAD
 bau-D9  YQRLGVLLDS ISTGLSAPTS SSLGIAT--- ---ATLSPAV SYKIGQYFKE QASNNANGQL TSGQEAAHIL AHTVLGAAVA AAGGNNALTA GLSAGGAEAA APILSSFL-Y GKDAKELTAD
 bau-D6  YQRLGVLLDS ISTGLSAPTS SSLGIAT--- ---ATLSPAV SYKIGQYFKE QASNNANGQL TSGQEAAHIL AHTVLGAAVA AAGGNNALTA GLSAGGAEAA APILSSFL-Y GKDAKELTAD
nos-D12  YQRLGVLLDS ISTGLSAPTS SSLGIAT--- ---ATLSPAV SYKIGQYFKE QASNNANGQL TSGQEAAHIL AHTILGAAVA AAGGNNALTA GLSAGGAEAA APILSSFL-Y GKAAKELTAD
bau-D10  DSNSVNQQSG LFAGNGGYHV KADSVDLKG- ---GAIVSTA TKDKNDLTTN RLT-FSNIEN QSQYDATTVS LSGGTSLGKG KT--GDSKAS TPTNNDNWRN ATSFSPSLPQ HESDKDSSTT
bau-D11  NSNSVNQQSG LFAGDGGYHV KADGVDLKG- ---GAIVSTA TKDNNVLTAN RLS-FSDVQN NSAYDATSVS LSGGTSLGGG KSSPGDSKAP KPTNNDNWRN ATSFSPSLPQ HDSDKDSSTT
bau-D14  NSNSVNQQSG LFAGDGGYHV KADSVDLKG- ---GAIVSTA TKDKNDLTTN RLT-FSNIEN QSEYDATSVS LSGGTSLGGG KSSPGDSKAP KPTNNDNWRN ATSFSPSLPQ HESDKDSSTT
bau-D12  DSNSVNQQSG LFAGDGGYHV KADSVDLKG- ---GAIVSTA TKDKNDLTTN RLT-FSNIEN QSEYDATSVS LSGGTSLGGG KSSPGDSKAP KPTNNDNWRN ATSFSPSLPQ HESDKDSSTT
bau-D20  NSNSVNQQSG LFAGDGGYHV KADSVDLKG- ---GAIVSTA SKDNNDLTAN RLS-FSDVQN NSAYDATSVS LSGGTSLGGG KTSTGDSKAP KPTNNDNWRN ATSFSPSLPQ HESDKDSSTT
bau-D15  DSAWVNNTSK LLIGDKDHDA NLDAMGVKQ- ---VTNIGGV IANATKNADG TLTDYKGLNY SGALELKDIQ DHNYNSSRGF NVSTTIGKTT QEKDGQKSKY PNGSTTLGLQ SNGQETEQLT
bau-D16  DSAWVNNTSK LLIGDKDHDA NLDAMGVKQ- ---VTNIGGV IANATKNADG TLTDYKGLNY SGALELKDIQ DHNYNSSRGF NVSTTIGKTT QEKDGQKSKY PNGSTTLGLQ SNGQETEQLT
bau-D21  DSAWVNNTSK LLIGDKDHDA NLDAMGVKQ- ---VTNIGGV IANATKNADG TLTDHEGLNY SGALELKDIQ DHNYNSSRGL NVSTTIGKTT QEKDGEKGKY PNGSTTLGLQ SSGQETEQLT
bau-D19  DSAWVNNTSK LLIGDKDHDA NLDAMGVKQ- ---VTNIGGV IANATKNADG TLTDHKGLNY SGALELKDIQ DHNYNSSRGL NVSTTIGKTT QEKDGQKSKY PNGSTTLGLQ SNGQETEQLT
bau-D13  NSNSVNQQSG LFAGDGGYHV KADSVDLKG- ---GAIVATA TKDKNDLTTN RLT-FSNIEN QSEYDATSVS LSGGTSLGGG KSSTGDSKAP KPTNNDNWRN ATSFSPSLPQ HDSDKDSSTT
cal-D23  DSNSVKQQSG LFAGDGGYHV KADSVDLKG- ---GAIVSAA TKDKNDLTTN RLT-FSNIEN QSQYDATTVS LSGGTSLGKG K--TGDSKAS TPTNNDNWRN ATSFSPSLPQ HDSDKDSSTT
pit-D20  DSNSVNQQSG LFAGDGGYHV KADSVDLKG- ---GAIVSTA TKDKNDLTTN RLT-FSNIEN QSQYDATTVS LSGGTSLGKG KS-TGDSKAS TPTNNDNWRN ATSFSPSLPQ HDSDKDSSTT
 pit-D6  DSNSVKQQSG LFAGNGGYHV KADSVDLKG- ---GAIVSTA TKDKNDLTTN RLT-FSNIEN QSQYDATTVS LSGGTSLGKG K--TGDSKAS TPTNNDNWRN ATSFSPSLPQ HESDKDSSTT
bau-D17  DSAWVNNTSK LLIGDKDHDA NLDAMGVKQV TNIGGVIANA TKNADGTLTD HKG-LNYSGA LELKDIQDHN YNSSRGLNVS ---TTIGKTT QEKDGQKSKY PNGSTTLGLQ SNGQETEQLT
nos-D18  DSAWVNNTSK LIIGDKDHDA NLDAMGVKQ- ---VTNIGGV IANATKNADG TLTDHKDLNY SGALELKDLQ DHNYNSSRGF NVSTTIGKTT QEKDGEKGKY PNGSTTLGLQ SNGQETEQLT
nos-D22  DSAWVNNTSK LLIGDKDHDA NLDAMGVKQ- ---VTNIGGV IANATKNADG TLTDHKDLNY SGALELKDLQ DHNYNSSRGF NVSTTIGKTT QEKDGEKGKY PNGSTTLGLQ SNGQETEQLT
 bau-D2  DKAWVNETSK LLIGNAQNDA DLDAMGVQK- ---VSNIGGV IANSTKNSDA SLTDHGKLNY TGALELKDLQ DHSSESNRGF NVSTSVGTSI KGESKESSFH PSGSTTVGLQ STGNEQEQLT
bay-D15  DSAWVNTTSK LLIGNAQNDA NLDAMGVKN- ---ITNIGGV IANANKNADG SLTDHKNLNY TGNLELKDLQ DHNNNSSGF NVSTNIGIP- QGGSKAASTA PKGSTTIGLN SSGQETEQLT

         5401                                                                                                            5520
 bau-D1  KLGKHVSDFG RNPANATDRE YVLAQIENIA RHPDKVVRGT FAGQGPMNTR GAVQFRIKGQ DVVVTKPDGT FVT------- -ILRNGIN-- --NPSVKQAL NGAK
 nos-D2  KTEAEQTRLR AAACALVECS KGVPESDANY KSLKAFEALG KTYTKEQNAL KATGLF---- TYSNWDKAND IRT------- SNDKYVSK-- --GKNVGQVV IGVAGTVGGG LATGGLALAC
 bau-D3  KTEVEQTRLR AAACALVECS EGVPESDVNY KTLKAFEALG KTYIKEQNAL KATGLF---- TYSNWDKAND IRT------- NHDEAVTR-- --VGGAKGVI TGGVTAIGGS AAGAGLCT--
 nos-D4  QKSTISSIVG LAGSAVGATT GDISSTVQSG QVAQNAVENN IFGVLTSNKQ I--------- ---DKQKNNE YFQ------- --REFACK-- --GNAQCLKT VLTENALDAM KYAQKELILN
 bau-D7  QKSTISSIVG LAGSAVGATT GDIVSTVQSG QVAQNAVEDN YLTVKQIQAY K--------- ---TQMQLCK VKQ------- --NCDQVI-- --DKFRKL-A IKQDEQLVAV CSTSPTNCKQ
 bau-D5  QKSTISSIVG LAGSAVGATT GDIGSIVQNG QVAQNAVENN FLSVKEAARK NALIYK---- SKHEELSSNE KKE----LAE INRDKAR--- --DEFIKNVC QLGNVSSSAC QSALQAAWST
 nos-D8  QKSTISSIVG LAGSAVGLTT GDIGSTVQSG QVAQNAVENN FLSVKEAARK NALIYK---- SKHEELSSNE KKE----LAE INRDKAR--- --DEFIKNVC QLGNVSSSAC QSALQAAWST
 bau-D9  QKSTISSIVG LAGSAVGATT GDIGSIVQSG QVAQNAVENN FLSVKEAARK NALIYK---- SKHEELSSNE KKE----LAE INRDKAR--- --DEFIKNVC QLGNVSSSAC QRALQAAWST
 bau-D6  QKSTISSIVG LAGSAVGATT GDIGSIVQSG QVAQNAVENN YLTYKEALTK EQAKTKLANC NRTNKCSPDE IRQAELTVKL LDSKDQIT-- --DVKILETC RT-NPQSSAC QQQKKDLQAT
nos-D12  QKSTISSIVG LAGSAVGATT GDIGSTVQNG QVAQNAVENN EFSIVTDASN KQAGKSIA-- KMRAECQANG INP------N SAACGQYQ-- --DAKLRKEA SGTLGNLSDG ATIVGLYPTP
bau-D10  YATLSEGNIK VGGKSTTVEQ LGIHSDASTA NRGVETLPNL QTILDKQKTV ADATSTIAAA TRTYSQNQQQ QAEKAKNAEK QNVLNQLGLD SDALKYYESL DSAKQEEYLR QYS----PDY
bau-D11  YATLSAGKIT VGGQSTTVEQ LGIHSDASTA NRAVETLPNL QTILDKQKTV ADATSTIVAA SRTYAQDQIK NAAAEKEAIG KQITSQLS-- SEEQTKLKEM TAAEKDAYLA QYGVY-DAAL
bau-D14  YATLSAGNIT VGGQSTTVEQ LGIHSDASTA NRAVETLPNL QTILDKQKTV ADATSTIAAA SRTYAQDQIK NAAAEKEEIG KQITSQLS-- SEEQTKLKEM TAAEKDAYLA QYGVY-DAAL
bau-D12  YATLSEGNIK VGGKSITVEQ LGIHSDASTA NRAVETLPNL QTILDKQKTV ADATSTIAAA SRTYAQDQIK NATAEKEAIG KQIASQLS-- PEEQIKLKGM SAADKDAYLA QYGVY-DAAL
bau-D20  YATLSAGNIN IGGKATTVEQ LGIHSDAATA SRTLETLPNL QTILDKQKTV ADATSTIAAA SRTYAQDQIK NAAAEKEAIG KQITSQLS-- SEEQTKLKEM TAAEKDAYLA QYGVY-DAAL
bau-D15  KATMGQGTVK ---NTTELTN RDINTTQEIT RDQTTGMLNG SFTVDHRLLS ESGRAEIVQQ QKDLPENFRQ ---------- SAENVIKAL- PESEYKNQVL ----QTLNNV QAKLY-KMPT
bau-D16  KATMGQGTVK ---NTTELTN RDINTTQEIT RDQTTGMLNG SFTVDHRLLS ESGRAEIVQQ QKDLPENFRQ ---------- SAENLVQAL- PDNAYKQRAL ----KSLNNI QAGLA-NNPE
bau-D21  KATMGLGSVT ---GAPTTAN RDVNNSQEIT RDQTTGMLNG SVTVDHRLLS ESGRAEIVQQ QKDLPENFRQ ---------- SAENLVQAL- PDNAYKQRAL ----KSLNNI QAGLA-NNPE
bau-D19  KAMMGQGTVK ---NTTELTN HDINTTQEIT RDQTTGMLNG SFTVDHRLLS ESGRAEIVQQ QKDLPENAEI ----VGKMTA AGVTSLGVAT AALATKDQNL ----KQAYDT VMNPA-RTFD
bau-D13  YATLSAGKIT VGGQSTTVEQ LGIHSDVSTA NRAVETLPNL QTILDKQKTV ADATSTIAAA TRTYSQNQAQ AAKKAEKDQLK AQAIKDLQ-- TEGGADWETY QSLSTDAAKQ EFLIKKDMGF
cal-D23  YATLSEGNIK VGGKSTTVEQ LGIHSDASTA NRAVETLPNL QTILDKQKTV ADATSTIAAA TRTYSQNQAQ AATKERDQLK AQAIKDLQ-- AEGGLDWETY QSLSTEAAKQ DFLVKKDMGY
pit-D20  YATLSAGNIT VGGKSTTVEQ LGIHSDAATA NRAVETLPNL QTILDKQKTV ADATSTIAAA SRTYAQDQIK NAAAEKTEIG KQIASQLS-- PEEQIKLKEM SASDKDAYLA QYGVY-DAAL
 pit-D6  YATLSAGNIK VGGQSTTVEQ LGIHSDTATA NRAVETLPNL QTILDKQKTV ADATSTIAAA SRTYALDQIK NATAEKEAIG KQIASQLS-- PEEQIKLKGM SAADKDAYLA QYGVY-DAAL
bau-D17  KATMGQGTVK ---NTTELTN RDINTTQEIT RDQTTGMLNG SVTVDHRLLS ESGRAEIVQQ QKDLPENFRQ SAENLAKALP DGVYKDKA-- LQTLNNIQAK LYNLPSEYKE VGVV------
```

```
nos-D18  KATMGLGTVK ---NTTELTN RDINTTQEIT RDQTTGMLNG SVTVDHRLLS ESGRAEIVQQ QKDLPENLRQ SAENVIKALP ESEYKSQV-- LQTLNNVQAK LYKMPTQYKD SGTL------
nos-D22  KATMGLGTVK ---NTTELTN RDINTTQEIT RDQTTGMLNG SVTVDHRLLS ESGRAEIVQQ QKDLPENFRQ SAENLAKALP DGAYKDKA-- LQTLNNVQAK LYNLPSEYKE AGVV------
 bau-D2  KATMGQGTLK ---NSTALSN RDINNTQEIT RDQATGLLNG SVTVDNRLLT ESGRQQIIQE QKDLPQNAEI IGKMTAAGVT SLGVATAS-- ------LASG DQNLKQAYDT VMNPARTFDF
bay-D15  KATLGQGSVT ---NATADTN RDINNTQEIT RDQTTGMLNG SVTVDHRLLS ESGRAEIVKE QEQLIENFRQ SAENLAKALP DGVYKDKA-- ------LQTL NDVQAKLYNL PSEYKEVG--

         5521                                                                                                                 5640
 nos-D2  PETGVSC-VG ATVAGTGTAL SAKQTVDGVK GLLTPYESTQ GQKVIQSYSP NRKDELSALT QDSIDLAV-- --AAGEAAL- GIVGGKVVTQ V---GGKAVE ITKDINRVLK NDPAKLPTVT
 bau-D3  --TGLGCAVG APLAGVSVVG GVSTAIEGKN QVFGEYTSQE GKKVIDSYNP NRKQELSQLS QDGLNGAV-- --AVAEAVLI GKVGAKLSGS E---AKVSSS VTSKLNPTFT IDPKIENQMS
 nos-D4  GKNYKPGDII SNPDPKSDGS GLKYIVVNKN GVLKAEILPA ADQVYYYYVTH KQELSKSIED MSFFSPGA-- --NSVLG-IY GAATGQTL-- ------LSGT QLSTGQRVLS AVDGVSSVLP
 bau-D7  YYQFILNDRL AVKEAIGDAG --KFIPAFDE SVLWSQQNTA EGIVSNTEFA KQLQQRYGLD EQTAKLTA-- --SAVMSAIT GVKAKQIS-- ------GNAS KNEITQNGYS FKKGIDLDLR
 bau-D5  QADYNADIAN NLKNRDVYSQ DAKHLDQLLK GLSQDQVLGL QAIERIAKTS GRPVEEVAKE YDRAMAL--- --HGVVSTLA GFYGGKAISV EKVKNGGGSF NALTEAEKQR ALDNIANSKI
 nos-D8  QADYNADIAN NLKNRDVYSQ DAKHLDQLLK GLSQDQVLGL QAIERIAKTS GRPVEEVAKE YDRAMAL--- --HGVVSTLA GFYGGKAISV EKVKNGGGSF NALTEAEKQR ALDNIANSKI
 bau-D9  QADYNADIAN NLKNRDVYSQ DAKHLDQLLK GLSQDQVLGL QAIERIAKTS GRPVEEVAKE YDRAMAL--- --HGVVSTLA GFYGGKAISV EKVKNGGGSF NALTEAEKQR VLDNIANSKA
 bau-D6  IASYAFKDSP AQYQAALNAE RNQDLG-LLK NLPQSSIVPV DYIK-----G NEFIYELAPT FLTGLQLT-- --SGGSISSL GKYSGEIIGA I----GGAAA GYVSGGDAQS VGIGAAVGVV
nos-D12  ASPYLLGAGT LLGTASTLLD DEKSIYQKGA EIGLSTIAGS AASKVVKGVK TPEGDRLYSE --TVEKIF-- --SETSGKVA EKVPGGVISC EKNPKQIGCG K
bau-D10  AKASDMNQAW GMGGDKSRAL NAVTMA-VTG ALGGQTDVQV VANTLAPYAA NMIGEKFGHG EDKNKAA--- --QLASHAIL GATLAYLNGG NPAAGGSAAV ASEAAADYLA NQYKDDPAYK
bau-D11  AKEKAVTQQW GMGGDKSRAL NAVTTA-ITG ALGGQTDLQV AANTLAPYAA NMIGEKFGHG EDKNKAA--- --QLASHAIL GATLAYLNGG NPAAGGSAAV ASEAAADYFA NQYNDGKTAI
bau-D14  ANEKAVTQQW GMGGDKSRAL NAVTTA-ITG ALGGQTDLQV AANTLAPYAA NMIGEKFGHG EDKNKAA--- --QLVSHAIL GATLAYLNGG NPAAGGSAAV ASEAAADYFA NQYNDGKTAI
bau-D12  AKEKAVTQQW GMGGDKSRAL NAVTTA-ITG ALGGQTDLQV AANTLAPYAA NMIGEKFGHG EDKNKAA--- --QLASHAIL GATLAYLNGG NPAAGGSAAV ASEAAADYFA NQYNDGKTAI
bau-D20  AKEKAVTQQW GMGGDKSRAL NAVTTA-ITG ALGGQTDLQV AANTLAPYAA NVIGEKFGHG EDKNKAA--- --QLASHAIL GATLAYLNGG NPAAGGSAAV ASEAAADYFA NQYNDGKTAI
bau-D15  QYKDSGTL-- GSEVVLGLIK QGVEPQ-TIN KLFDDKAAGM -----TTVLE NLNDLNNKID HLKQ------ ----QGLSIN DILGTDRNQS PTANDEITFK DYKVEIQNQS TIGMKLLQEI
bau-D16  MLAKGGDE-- VYEGYKEFIR QGGEPE-QYE ALLNKEVLPL AKELNNTAKQ TLDEIQKQIA EEYQ-VS--- --DDTARSIV REVLTAVSQQ DGANTNTTDE TGTLNPNQPY KGTFELPTIV
bau-D21  MLAKGGDE-- VYEGYKEFIR QGGEPE-QYE ALLNKEVLPL AKELNNTAKQ TLDEIQKQIA EEYQ-VS--- --DDTARSIV REVLTAVSQQ DGANTNTTDE TGTLNPNQPY KGTFVLPTIV
bau-D19  FVQKHPEAAT VLEQFKNGNY DGLLQT-KGS IQLLAQALGQ DVDVLTTSMT SFLGIKGAYD HQTNTVV--- --LDVNNENR SSILSTTGHE IAHGQGISNE TSADLVGKSV DWAFSSGVKN
bau-D13  AEAFNNAESW GMGGDKSRAL NAVTTA-ITG ALGGQTDLQV VANTLAPYAS KLIGEKFGHG EDKNKAA--- --QMASHAIL GAVLAYVNNG NPAAGGSAAV ASETAADYLT KQYKDNPAYQ
cal-D23  AEAFNDAQSW GMGGDKSRAL NAVTTA-ITG ALGGQTDLQV VANTLAPYAS KLIGENLGHG EDKNKAA--- --QMASHAIL GAVLTYVNNG NPAAGGSAAV ASETAADYLT KQYKDNPAYQ
pit-D20  ANEKAVTQQW GMGGNKGRAL NAVTTV-ITG ALGGQTDIQV AANALAPYAA QQIGEKFGHG EDKNKAA--- --QLASHAIL GATLAYLNGG NPAAGGSVAV ASEAAADYFA NQYNDGKTAI
 pit-D6  ANEKAVTQQW GLGGNKGRAL NAVTTV-ITG ALGGQTDIQV AANTLAPYAA NIIGEKFGHG EDKNKAA--- --QLASHAIL GATLAYLNGG NPAAGGSAAV ASEAAADYFA NQYNDGKTAI
bau-D17  --GNSVASEL LKRGMESQDV EALFSK-LDF FYAAK-EFSE IQNQLTDLEK S--GVNLETI FKEPKAG--- --GNEQETIY AQGTDFETKV SSQTTLGMTI LSNVADLGNS IKDISESTGV
nos-D18  --GSEVVLGL IKQGVEPQTI NKLFDDKAAG MTTVLENLND LNNKIDHLKQ QGLSINDILG TDRNQ-S--- --PTANDEIT FN--DYKVEI QNQSTIGMKL LQEINFIGNS IEEISKDTGV
nos-D22  --GNNVASEL LKRGMESKDV EALFSK--LD FFYAAKEFSE IKNQLTDLEK SGFDLETIFR QPKSGVN--- --EEKTIYAQ GT--DFETKV SSQTTLGMTI LSNVADLGNS IKDISESTGV
 bau-D2  VQKHPEAATV LEQFKNGNYD RLLATK--GS IQLLAQALGQ DVNVLTTSIT SFLNIKGAYD HQTDTVVLDV NNENRSSILN TTGHEIAHGQ GIKNETSADL VGKSVDWAFN SGVKNNQVTI
bay-D15  VVGNSVASEL LKRGMESQDV EALFSK--LD FFYAAKEFSE IQNQLTDLEK SGVNLETIFK QPKAGV---- ---NEQETIY AQGTDFETKV RSQTTLGMTI LSNIADLGNS IKDISESTGV

         5641                                                                                                                 5760
 nos-D2  ASASGNISAK QISENGKIID PPKEVLSKQK QLLNITDNNT SGILREEIAD SYFTNSGYTK LESKCG---- ---------- -NNCFDGVYM KNGELYVVEV KPLKERGSVK LSDNKNSPNN
 bau-D3  TRGWSNKDIA DVMKKG---- PVGYTIDKRR -----ASKTP DNLPRNDTAT VYGSKNGYVV VNDRTK---- ---------- -EVV--QISD KTDKNWIPDS RIQWK
 nos-D4  GVVF-LKDSK VLSSDSVGMT WGKGINEQGK PWENYIQSTS PATTIDLNSI KSNFKAFDHF DPATGL---- ---------- -ATSDKTLNT AAKTYQDPKK ITSQINKYID QMDNFTGDNK
 bau-D7  GKNYSTKDAL NIAFEKTGVP KNEFTVTKWA VDQN--GKSF PAEYRVLSG- -SNRGAEVSV DLGH------ ---------- --TSKGTPGA PHVGWQTPGK KNTVGHIFLD NVNVNRSKSK
 bau-D5  GRESSNFDTH VINEKVAATI PPNMQQFLME HNTQIGSRNS QGSIGGSHNA DSFNQALERS KSAIIG---- ---------- -DPVIDPKYP GLKTYNYQAP AVDRAGKPTG NYKDKQQKTV
 nos-D8  GRESSNFND- -FNQKAK-EI EVYEN-LKTQ IDSKIAQSQR KHIKDDAQHT HGSYFNNTQD AQKVLD---- ---------- -AFHNNEVKI VGYNKQGQPI IKYDGVKGIY ATKNKEGES-
 bau-D9  GRESSNFND- -FNQKAKSEA KTYKKETETT VNANISEDKN ALTPIGREGS PLSVPRGTNS SSTING---- ---------- -NTYSGHALD EMQADGIMSS VVDNTLKTIT PVKGKVPGTV

 bau-D6  GGR------- -VNEKVVGKV ESTLK--KGG YDPLAIGMAK GATTIGMGGA SGA--GGTVI NNYVNN---- ---------- -DPLKKDVPK SAVVGALVPA ISGEAAVTAA GVGGKIQKVV
bau-D10  NGK-GEFEPN LLPENVKSSI RDLTAAIGAV VGGTV--GDS ASNVQLAGVI GQNAVENNID SPAER----- KASQQIMKKE LNASCSAAGL APGSAACGQY LRNETLTILK KAGSLTADFL
bau-D11  NPETGEFDAN LLPENVKSGI RDLTAAIGAV VGGTV--GDS ASNAQLAGVI GQNAVENNTV SDMVNAHLKG KTLAQEAEDQ VKEQDEAYRK NNCSGLSASA CSAQMYKERR EVLWIGTSFI
bau-D14  NPETGKFDAN LLPENIKSGI RDLTAAIGAV VGGTV--GDS SSNAQLAGVI GQNAVENNEF SIITKG--VE KKLAEN---- -KKEKEAQAH LICP--KGQS CIIPIPEKSL GDKALNVIND
bau-D12  NPNTGEFDAN LLPENVKSGI RDLTAAIGAV VGGTV--GDS ASNAQLAGVI GQNAVENNEF SIVTEA--SN KQAGKT---- -IAKMAECQ AKGI--NPNS GACGQYHDTK LRKDISDTSG
bau-D20  NPETGKFDAN LLPENVKSGI RDLTAAIGAV VGGTV--GDS ASNAQLAGVI GQNAVENNEF SIITKG--VE KKLAEN---- -KKEKEAQAH LSCP--KGQS CMIPIPEKSL GDKALNVINN
bau-D15  NFIGNSIEEI SKDTGVDLET VQLAVGLMIS GPVRL--VVG SAQSLGMDAL AGPY-KKKGI D------VLA TAFTASAHST STDVVEKMIN PQYAKEVPEA ERD------- ---NYESLSN
bau-D16  VTAKGDAVLI QGSQSDEASI AGENVVKVID GSNLA--IKL FDQSAAVSYK VNDAIEKTGI DPATAGLALS LAFGGPIGLA RDLITDTLVG EPLAQGSDVL KNEVTAV--V RDTSYDTVSG
bau-D21  VTAKGDAVLI QGSQSDEASI AGENVVKVID GSNLA--IKL FDQSAAVSYK VNDAIEKTGI DPATAGLALS LAFGGPVGLA RDLITDTLVG EPLAQGSDVL KNEVTAV--V RDTSYDTVSG
bau-D19  NEATIDQYKD QLGDGKDAST QAQNVAVLEK DNTKA--LD- -----AISDH ADQIDEKTSY WQDIKHLGCW SKECTVAYQK MDAAQEKAFR LGQAKAVTKF MNDIKNLPNV PKEVYDALTN
bau-D13  N-DKGEFDAN LLPEDVKSSI RDLTSAIGVA VGGTV--GDS AFSAQIAGVV GQNAVENNLF GVLTA----- ---------- -DKKIDKQKN NSYFQALQAC KNAQCEKSVI TQNALAAMQN
cal-D23  N-DRGEFDAN LLPEDVKSSI RDLTSAIGVA VGGTV--GDS AFSAQIAGVV GQNAVENNLF GVLTA----- ---------- -DKKIDKQKN NSYFQALQAC KNAQCEKSVI TQNALAAMQN
pit-D20  NPETGKFDAN LLPENVKSGI RDLTAAIGAV VGGTV--GDS ASNAQLAGVI GQNAVENNEF S---I----- ---------- -ITKGVEKKL ADNKKEKEAQ AHLSCPKGQS CMIPIPEKS-
 pit-D6  NPDTGEFDAN LLPENVKSGI RDLTAAIGAV VGGTV--GDS KSNAQLAGVI GQNAVENNYL SYKEV----- ---------- -LTKEQAKTK LENCNRTNKC SPNEIRQAEL TIKLLESKDQ
bau-D17  DIEKVQLAVG LLISEPVRLV --IESGKSMA VDSVV--GEK VAQGVDILAN SMTAAAHDT- SVNTI----- ---------- -SNWTNPDKI SELETSTGEN KEASLKFANE VQKDKQGAEY
nos-D18  DLETVQLAVG LMISGPVRLV --VGSAQSLG MDALA--GPY KKKGIDVLAT AFTASAHSTS TDVV------ ---------- -EKMINPQYA KEVPEAERDN YES---LSNE LSSSKQGAEY
nos-D22  DIEKVQLAVG LLISGPVRLV --VESGKSMA VDSLV--GDK VAQGIDILAN SMTAAAHDTS VNTI------ ---------- -NNWTNPDKI SELENSTSEN KEASLEFANE VQKDKQGAEY
 bau-D2  DQYKGQLGDG KDASTQAQNI AELVKDNTTI VDAISDHGDK IDEKTSVWQD IKYLGCWNDE C--V------ ---------- -AAYKQMDAA QEKAYRLGQA K-ATTKFVND IKNLPNVPKE
bay-D15  DIEKVQLAVG LLISGPVRLV IESGK--SMA VDSVV--GEK VAQGVDILAN SMTAAAHDTS VNTI------ ---------- -NNWTNPDKI SELENSTGEN KDASLKFANE VQKDKQGAEY

         5761                                                                                                                 5880
 nos-D2  IGVQMTNEWI ISRANELANS KNSPTAKATG NKILQAVEGG KPINKIVVGV NDSRAVTINL GNKVSSK
 nos-D4  GRFELTNDKI KSKEMQLAIP VNTSKSQLDA IQKSIDYANT KGITIKVTKV K
 bau-D7  D
 bau-D5  YDPKVISDQK MVSMQMQAVK KGAELLLSMP KDKREVNVNI EGYIFNVTRD RNTGKISNAF PIIPNAPSPV IPPKYKSKNI K
 nos-D8  --FESITNYF LI----KGTK KVSIVPASPK GPK
 bau-D9  AYLDPVNNIT VI----RSTT TGKIITVSRG QIRQ
 bau-D6  SINSAVVGTA AEKIQKVAPK KASQNNKEGG K
bau-D10  PVIGDIKGFV EAETVGDYVF ASVGLVPLVG DAAKDFYKAE KAYE-AASKV GDVAKMKAAM QDAVHACSGS TCFTAGTLIE TDQGLKAVEE FVGGELVWAR NDLTLEYGYR PVIATKVTAD
bau-D11  PVIGDIQTIA QAQSAADVIW GIAGFVPGLG EARNAYKVAE KAGDLAEMKK ALEKGVAIAT EKGHINKTKI TASQTTLTVT NKSDEHLVNQ IAKGQDQGGE LTEQLFDSLA KQNGFTVVKG
bau-D14  MTLRQLAAVA GAKYDPLTGE EITPNERQLA KASMLGLGLT KSVS-GVTRL TDEALVGIEK QYGKSIAEKI NKSNAVNLNA QDALNRKLSG LEKAQKMA-E VTKTLPDGRV RYYNKEVT-A
bau-D12  -TLSDISTAA GLYPTPAS-- -----PYLLG AGTLLGTAST LLDD-EKSIY QKGAEIGLSS VAGAAASKVV S-GVKTLDGD RLYSETVEKI FSEASGKVAE KV-PGGVISC EKNPKQIGCG
bau-D20  MTLRQLAAVA GAKYDPLTGE EITPNERQLA KASMLGLGLT RTVS-GVTRL TDDALIGIEK QYGKSIAEKI S-NNFYRDDD LISRTKVRDE GLKNAEKIST ATNPKNIAKG QVNQPRNVQE
bau-D15  ELSSSKQGAE YLINAAAGIV VGGIGKGAVK NNNSDIDISK SGFEIGNINP ITGHKILGTH KDESNNTVGN YIVEGTKPST KDKVIKTDIQ YYENPGHHDP KGGNQIGYNK TKSVLPDNHK
bau-D16  YTSNSTKQQL SGLTDPASQT ALQVSEQLQR TKDGVGFLAS VVLGAGGIAK TAKIDGNEVE VSTAGIGSRG ELTKPYDPVQ TRNDLEVVHG SENVKSTTVP PYNAKNVKLA GQRHPVTGVV
bau-D21  YTSNSTKQQL SGLTDPASQT ALQVSEQLKR TKDGVGFLAS VVLGAGGIAK TAKIDGNEVE VSQSGL-EIG WVDQYGVQKK VTGDGTVARD HQPSKAALIR RAEQLKGEPL TQSEK-NRIV
bau-D19  DPMGAMAAIW DGVKNIPGE- -LWQTGKTIT NVNLVGNSPA EFEKLGNAEM TTVLNAVSAG TITVAKKGGK IVVEAAKKVK LKADFDPSLN VQTNGFSLPP KTTLKVINIK ADQAIKGTKE
bau-D13  VPKVLNVSSS KYKEGEIITN PNDVTGLRYL VVKDGGVMKA KLLPADYQVV FSVNQLNSS- RNLVQGGALA SVIANTLK-S GHDVFADRTL FTNQEITALD RAFAGLEVVG NTLLIGGSVL
cal-D23  VPKVLNVSSS KYKEGEIITN PNDVTGLRYL VVKDGGVMKA KLLPADYQVV FSVNQLNSS- RNLVQGGALA SVIANTLK-S GHDVFADRTL FTNQEITALD RAFAGLEVVG NTLLIGGSVL
pit-D20  LGDKALNVIN NMT----LRQ LAAVAGAKYD PLT-GEEITP NERQLAKASM LGLG-LTRT- VSGVTRLTDD ALIGIEKQ-Y GKSIAEKISN NFYRDDDLIS RTKVRDEGLK NAEKISTATN
 pit-D6  ITDTKILETC RAN----PQS PACQQQKKEL QAT-IGSYAF KDSPTQYQTA LNAE-RNQD- LGLLKNLQQS SIVPADYK-N GNGFIYELAP TFLTGLQLTS GGSISSLGKY SGEIIGAIGG
bau-D17  LINAAAGIVV GGIGGTAKTT KSDGKEAGVN SVGVGGATWD TSKPVKTLDK VDNYYINKTD ENIIKQIDID HIAQGHINNS GKAVGFHHEP SGGQAARVTE YTSPPNAQGV YKGQVEVKSN
nos-D18  LINAAAGIIV GGIGKDIAKK GENGNISSTN NIN-EEISAL NRIKENDKGT DLSNKSSDSI INQQASKRVD EVTAPIDFDG HILNVEISKR GKLTGGHSIA NGKVNI-VSK SAPNAQGVYE
nos-D22  LINAAAGIVV GGIGGTAKTT KIDGKDVEVS TAGIGSNSKK DPVKELEVGS YQELKSRAQV GDGLEHDHIP SFAALKKAKE N----ELGKE LTDNEAKALY NDATTIEISR ETHKAGRTYG
 bau-D2  VYEALTKDPM GTMSAIWEGV KNIPGELWDT GKTITKGNLV GKSPAEFEKM GNAEMTTALN GLSAAISAGT VTVAKKGGGI VIEAAKSAKR ILQNDPAKLP TITTSAGGYI KASLITENGK
bay-D15  LINAASGIIV GGIAK--GTV KNNNSDIDIS KSGFEIGNI- --NPITGHKI LGTHKDESNN TVGNYIVEGT KPSTKD---- --KVIKTDIQ YYENPGHHDP KGGNQIGYNK TKSVLPDNHK

         5881                                                                                                                 6000

bau-D10  QPIFHVTVQN EQGQIDVLET TAEHPFWIKD LGWLKASLLQ SGMTLLDRDN QEITIVSQAL ISNKLETVYN IEVEGFHTYH VGELGVWVHN ANCCDIKSTN AFVPIKPKLN YTVDIENKAH
bau-D11  GKYGANNGFD HV--WVAKDG SVTILSDSKQ IKNGTVSLNP NGAGGNTQLS SDWIAAVMRK LPDSDPTKIA VEQARI---- AGKLKTAVTG VDRATGKAVI IPIQVPSKPT ISKKVTK
bau-D14  AKDGPTRGAS FVTEYNPETG AVRQWMESYD HSGNVIRVHP KSINGQPVIG QHYPPTGKEI QLGAK
bau-D12  K
bau-D20  QIVWNDVVEN PKSGVELRSL NKDQRFPQDA GFVKMSRTVK TKEGKTVEVH YQYNDVTKKV YDMKIVTKPF HEQ
bau-D15  TLWDNSIVAS DGNRWAVEVI NGKKVYHRFQ NDGNGNFHWN GSTNGKTASG VDRSDVGKIP PEILRK
bau-D16  FDNKGYPVFD DIARYDTNLI NKGYSNLSYT KQMELASKDL WNAIQRGEVN SSQFSSSQLR QIQSGSS--- KIDDYTWHHH QDNGRMQLVP EWEHSKTGHI GGDAMQGGK
bau-D21  NEAISVTVPN SVHVAGPTNA GKNTKELIEK DASDLQAAQK RDSDAMVE-N AKVLTPEHVQ KLEEACT--- TLGCKTNVDY DKFLKNIIQE EAKKNDK
bau-D19  YDILNNPPAN SHVKLDNGTE FKTNSYGRVD EVEFQPSLTK NPRDSRQTAV GKQGNKGDVG GHIQGCSLGG TCDNFNLFPQ NSNFNNSAYK KWENEIRGAL KNGEQVGKVK VQFDRSNPTN
bau-D13  PKKVSIDAVA FKENNIYRDG DLDNVLNGNP NAPADFAIGK KKATDISSGL DNPHYDSKHG AYTTLEQQYD RAMNG--TNP QTGGKGRPAD ASKFFNSTDM QTAIGKAEAL YKQNPSNVKE
cal-D23  PKKVSIDAVV VKENNFYRDN DS---ITGSF NYGKTISIDP TRLSFSQATV SHQKVGKPYN YAGMVESMKK NGWNGDPVDI VIMPNGSATS MDNTRILAAR EAGIDVKAKV RDFNTPLTSL
pit-D20  PK-NIAKGQV NQPRNVQEQI -VWNDVVENP KSGVELR-SL NKDQRFPQDA GFVKMSRTVK ----TKEGKT VEVHYQYNDV TKKVYDMKIV TKPFHEQ
 pit-D6  AAAGYVSGGD AQSVGIGAAV GVVGGRLNES VVGKIDR-KL TTAGYDPLAV GIAKGTTTIG MGGLSGAGGT VINNYFNNDP LKKDVPKSAV VGALVPTISG EAAVTAAGAG GKIQKVVSIN
bau-D17  TSNKWITKVD KQGQPNGSTF FPQGWSEARL KYELSEAFKN KPANLRPQEG FTTTTPSGVK VKFVPPSGNI SQWRGWPLQ
nos-D18  AKISVSDPKN PNKFLTKNST MFPDSWSADR VKVEVDAAYK NKSIVTNSRG ERMWEGITPS GVKVTGYLEP NTTVYPLMK
nos-D22  GKNTSEQIVK DASNLCEAQR CDIDVLRQNL LERGHSQKAV DEAIIKLKER NKD-KGIY
 bau-D2  IIDPPKDVLN KQKQLLGNKD KNVTGVLREE IADSYFKNSG YTKLESKCGS NCFDGVYMKN GELYVVEVKP LSRNAINLSS NKNSPNDIGV QMSDKWIDSR ITALKESNNV DSMKTATILQ
bay-D15  NLWDNSIVAS -------DGN RWAVEVINGK KVYHRFQNDG NGNFHWNGST NGKTASGVDR SDVSKIPPEI LRK

         6001                                                                                                                 6120
bau-D10  GYQNIDVSTK QTFDSNGIPK DGKFTKSYLY SSFNENSGEL YIQKMAALQP GNATGKEMLS QVIEKIGYSK IKTAKAELAQ TNKEAFDNAY KKSGNLIDAV NNTPLGKSMR DLGFKVKSVE
bau-D19  IRPD---SLK IDYSINGVKL RKTFTNESSK
bau-D13  DRG----QIS VNIEFNRPIG EGFTGNTKTN VRNNIPVGEY RWSNTATINI DPKTGKAFTA YPNLKLGQSK PNPLKRR
```

```
cal-D23  EKD----RFK SGNVVPKTWG EAI--KLRVE KQGQFEKGWE IKFPYGSI-Y DPKVTK
 pit-D6  SAV----VGT AAEKIQKVEP KKATQNNKEG NK
 bau-D2  KAR------L DKKPINKIVV GVNDSRAITL NLGQVGSK

         6121       6133
bau-D10  NTSGMPKVIF ERK
```

Supplementary file 9

a) type-II CdiA repeats. The first ten 20-21 aa repeats found in type-II CdiA are shown. The first four are common to all proteins. From repeat 5 on, class 1 (blu residues) and class 2 and 3 proteins (red residues) repeats diverge.
Regularly repeated residues are highlighted.

```
bau-D9   LNNDQGAIIANGGVDLDSQNG LDNQGGQLNLGAIHIKGNS FNNNQGELTVKSADIQTSS FSNQQGLLQSLTSLDVNSQS IDNQGGKINALNNISMISSGN ILNQAGQIASSSELYLQGLG LNNSGGDLEAEQLLKLNLSGH LNNQKGKIVTNNNLDSSLFG LDNDQGEISAKNITIQNNDQA LSNGSGTIYADQSLKIQTGS class 1 CdiA
bau-D7   LNNDQGAIIANGGVDLDSQNG LDNQGGQLNLGAIHIKGNS FNNNQGELTVKSADIQTSS FSNQQGLLQSLTSLDVNSQS IDNQGGKINALNNISMISSGN ILNQAGQIASSSELYLQGLG LNNSGGDLEAEQLLKLNLSGH LNNQKGKIVTNNNLDSSLFG LDNDQGEISAKNITIQNKDQA LSNGSGTIYADQSLKIQTGS
bau-D5   LNNDQGAIIANGGVDLDSQNG LDNQGGQLNLGAIHIKGNS FNNNQGELTVKSADIQTSS FSNQQGLLQSLTSLDVNSQS IDNQGGKINALNNISMISSGN ILNQAGQIASSSELYLQGLG LNNSGGDLEAEQLLKLNLSGH LNNQKGKIVTNNNLDSSLFG LDNDQGEISAKNITIQNKDQA LSNGSGTIYADQSLKIQTGS
bau-D6   LNNDQGAIIANGGVDLDSQNG LDNQGGQLNLGAIHIKGNS FNNNQGELTVKSADIQTSS FSNQQGLLQSLTSLDVNSQS IDNQGGKINALNNISMISSGN ILNQAGQIASSSELYLQGLG LNNSGGDLEAEQLLKLNLSGH LNNQKGKIVTNNNLDSSLFG LDNDQGEISAKNITIQNNDQA LSNGSGTIYADQSLKIQTGS
bau-D3   LNNDQGAIIANGGVDLDSQNG LDNQGGQLNLGAIHIKGNS FNNNQGELTVKSADIQTSS FSNQQGLLQSLTSLDVNSQS IDNQGGKINALNNISMISSGN ILNQAGQIASSSELYLQGLG LNNSGGDLEAEQLLKLNLSGH LNNQKGKIVTNNNLDSSLFG LDNDQGEISAKNITIQNKDQA LSNGSGTIYADQSLKIQTGS
bau-D1   LNNDQGAIIANGGVDLDSQNG LDNQGGQLNLGAIHIKGNS FNNNQGELTVKSADIQTSS FSNQQGLLQSLTSLDVNSQS IDNQGGKINALNNISMISSGN ILNQAGQIASSSELYLQGLG LNNSGGDLEAEQLLKLNLSGH LNNQKGKIVTNNNLDSSLFG LDNDQGEISAKNITIQNKDQA LNNGSGTIYADQSLKIQTGS
nos-D2   LNNDQGAIIANGGVDLDSQNG LDNQGGQLNLGAIHIKGNS FNNNQGELTVKSADIQTSS FSNQQGLLQSLTSLDVNSQS IDNQGGKINALNNISMISSGN ILNQAGQIASSSELYLQGLG LNNSGGDLEAEQLLKLNLSGH LNNQKGKIVTNNNLDSSLFG LDNDQGEISAKNITIQNKDQA LNNGSGTIYADQSLKIQTGS
nos-D4   LNNDQGAIIANGGVDLDSQNG LDNQGGQLNLGAIHIKGNS FNNNQGELTVKSADIQTSS FSNQQGLLQSLTSLDVNSQS IDNQGGKINALNNISMISSGN ILNQAGQIASSSELYLQGLG LNNSGGDLEAEQLLKLNLSGH LNNQKGKIVTNNNLDSSLFG LDNDQGEISAKNITIQNKDQA LNNGSGTIYADQSLKIQTGS
nos-D8   LNNDQGAIIANGGVDLDSQNG LDNQGGQLNLGAIHIKGNS FNNNQGELTVKSADIQTSS FSNQQGLLQSLTSLDVNSQS IDNQGGKINALNNISMISSGN ILNQAGQIASSSELYLQGLG LNNSGGDLEAEQLLKLNLSGH LNNQKGKIVTNNNLDSSLFG LDNDQGEISAKNITIQNKDQA LNNGSGTIYADKSLKIQTGS

bau-D12  LNNDQGAIVANGGVDLDSQNG LDNQGGQLNLGTINIKGNS FNNDQGQLTVKSANIQTSS FTNQQGQLASNSTLNIQTQS ANNKDGKIQSIGQLDLAVTGE LNNVDGQIASGANINVTASD LKNQSGVVYSENQAVNIKANKA IDNTEGLIQAKTNLNLDSQS LNNTAGQIVADQINQKHVT VNNNQGSIAAQQNLNSTAQQF class 2 CdiA
bau-D10  LNNDQGAIVANGGVDLDSQNG LDNQSGQLNLGAINIKGNS FNNNQGQLTVKSANIQTSS FTNQQGQLTSNSTLNIQSQS ANNKSGKIQSIGQLDLAVTGE LNNVDGQIASGATVNVTASD LKNQSGVVYSENQAVNIKVNKA IDNTEGLIQAKTNLNLDSQS LNNTSGQIVADQINQKHVT VNNNQGSIAAQQNLNSTAQQF
bau-D11  LNNDQGAIVANGGVDLDSQNG LDNQGGQLNLGAINIKGNS FNNDQGQLTVKSANIQTSS FTNQQGQLASNSTLNIQTQS ANNKDGKIQSIGQLDLAVTGE LNNVDGQIASGANINVTASD LKNQSGVVYSENQAVNIKANKA IDNTEGLIQAKTNLNLDSQS LNNTAGQIVADQINQKHVT VNNNQGSIAAQ-NLNSTAQQF
bau-D13  LNNDQGAIVANGGVDLDSQNG LDNQGGQLNLGAINIKGNS FNNDQGQLTVKSANIQTST FTNQQGQLASNSTLNIQTQS ANNKGGKIQSIGQLDLAVSGE LNNVDGQIASGATVNVNASD LKNQSGVVYSENQAVNIKTNKA IDNTEGLIQAKTNLNLNSQS LNNTSGQIVADQIKQKHVT VNNNQGSIAAQQNLNSTAQQF
bau-D14  LNNDQGAIVANGGVDLDSQNG LDNQGGQLNLGAINIKGNS FNNDQGQLTVKSANIQTSS FTNQQGQLASNSTLNIQTQS ANNKDGKIQSIGQLDLAVTGE LNNVDGQIASGANINVTASD LKNQSGVVYSENQAVNIKANKA IDNTEGLIQAKTNLNLDSQS LNNTAGQIVADQINQKHVT VNNNQGSIAAQQNLNSTAQQF
bau-D20  LNNDQGAIVANGGVDLDSQNG LDNQGGQLNLGAINIKGNS FNNNQGQLTVKSASIQTSS FTNQQGQLASNSTLNIQTQS ANNKSGKIQSIGQLDLAVSGE LNNVDGQIASGATVNVNASD LKNQSGVVYSENQAVDIKANKA IDNTEGLIQAKTNLNLDSQS LNNTSGQIVADQINQKHVT VNNNQGSIAAQQNLNSIAQQF
cal-D23  LNNDQGAIVANGGVDLDSQNG LDNQGGQLNLGAINIKGNS FNNNQGQLTVKSANIQTSS FTNQQGQLASNSTLNIQSQS ANNKGGKIQSIGQLDLAATGE LNNVDGQIASGATVNVTASD LKNQSGVVYSENQAVDIKANKA IDNTEGLIQAKTNLNLNSQS LNNTAGQIVANQINQKHVT VNNNQGSIAAQQNLNSTAGQF
pit-D20  LNNDQGAIVANGGVDLDSQNG LDNQGGQLNLGAINIKGNS FNNNQGQLTVKSASIQTSS FTNQQGQLASNSTLNIQTQS ANNKSGKIQSIGQLDLAVSGE LNNVDGQIASGATVNVNASD LKNQSGVVYSENQAVDIKANKA IDNTEGLIQAKTNLNLDSQS LNNTSGQIVADQINQKHVT VNNNQGSIAAQQNLNSIAQQF
pit-D6   LNNDQGAIVANGGVDLDSQNG LDNQGGQLNLGAINIKGNS FNNNQGQLTVKSANIQTSS FTNQQGQLASNSTLNIQTQS ANNKGGKIQSIGQLDLAVIGE LNNVDGQIASGATVNVNASD LKNQSGVVYSENQAVNIKANKA VDNTEGLIQAKTNLNLDSQS LNNTSGQIVADQINQKHVT VNNNQGSIAAQQNLNSIAQQF

bau-D15  LNNDQGAIVANGGVDLDSQNG LDNQGGQLNLGAINIKGNS FNNNQGQLTVKSANIQTSS FTNQQGQLASNSTLNIQTQS ANNKGGKIQSIGQLDLAVTGE LNNVDGQIASGATVNVNASD VKNQSGVVYSENQAVNIKANKA IDNTEGLIQAKTNLNLDSQS LNNTSGQIVADQINQKHVT VNNNQGSIAAQQNLNSIAQQF class 3 CdiA
bau-D16  LNNDQGAIVANGGVDLDSQNG LDNQGGQLNLGTINIKGNS FNNDQGQLTVKSANIQTSS FTNQQGQLASNSTLNIQTQS ANNKDGKIQSIGQLDLAVTGE LNNVDGQIASGANINVTASD LKNQSGVVYSENQAVNIKANKA IDNTEGLIQAKTNLNLNSQS LNNTSGQIVADQIKQKHVT VNNNQGSIAAQQNLNSTAQQF
bau-D17  LNNDQGAIVANGGVDLDSQNG LDNQGGQLNLGAINIKGNS FNNDQGQLTVKSANIQTST FTDQQGQLVSNSTLNIQTQS ANNKGGKIQSIGQLDLAVSGE LNNVDGQIASGATVNVNASD LKNQSGVVYSENQAVNIKANKA IDNTEGLIQAKTNLNLNSQS LNNTSGQIVADQIKQKHVT VNNNQGSIAAQQNLNSTAQQF
nos-D18  LGNDQGTIIAHGGVDVESQNG LDNQGGQLNLGTVTIKGSN FNNDLGQLNVKQADIQTLD FTNQQGQFASSSTLKIQSQN AYNQSGKIQSIGQLDLAIGSE LNNNKGYIASSDNVQVSAGD LSNQFGVIYSENQSVNIKANQA IDNTEGFIQAKTDLNLNSQA LNNTSGHIIGNQIQQQHGT VNNSQGAIIAKQNLNSTAQQF
bau-D19  LNNDQGAIVANGGVDLDSQNG LDNQGGQLNLGTINIKGNS FNNDQGQLTVKSANIQTSS FTNQQGQLASNSTLNIQTQS ANNKDGKIQSIGQLDLAVTGE LNNVDGQIASGANINVTASD LKNQSGVVYSENQAVNIKANKA IDNTEGLIQAKTNLNLNSQS LNNTSGQIVADQIKQKHVT VNNNQGSIAAQQNLNSTAQQF
bau-D21  LNNDQGAIVANGGVDLDSQNG LDNQGGQLNLGAINIKGNS FNNDQGQLTVKSANIQTSS FTNQQGQLASNSTLNIQTQS ANNKDGKIQSIGQLDLAVTGE LNNVDGQIASGANINVTASD LKNQSGVVYSENQAVNIKANKA IDNTEGLIQAKTNLNLDSQS LNNTAGQIVADQINQKHVT VNNNQGSIAAQQNLNSTAQQF
nos-D22  LGNDQGTIIANGGVDLESQNG LDNQGGQLNLGIVTIKGSN FNNDLGQLNVKQADIQTLG FTNQQGQLASSDTLKIQSQN ANNRSGKIQSIGQLDLAIGGE LNNSKGYVASGDNVLVSAGD LNNQFGVVYSENQSVNIKANQA IDNTEGVIQAKTDLNLNSQT LNNTSGQIIGNQIQQQHGT VNNSQGAIIAKQNLNSTAQQF
bay-D15  LNNDQGAIVAQGGIDLESNNG LDNQGGQLNLGRVKIQGER FNNDQGQLTVKQAEIQTNS LSNQSGQLISNQKLNIQTQT ANNQKGKIQSVDQLDLNVANG LNNIEGQIASGQQLNIQADQ VNNQSGLIYSEQQGIELKANHA INNTSGIIQAKTQLNLASTQ LDNTSGHIQAENIQQQHTT VNNTQGSIVAGQSLISTVDQF

         |----repeat 01--------| |----repeat 02-----| |----repeat 03----| |----repeat 04------| |----repeat 05------| |----repeat 06------| |----repeat 07-------| |----repeat 08-----| |----repeat 09----|   |----repeat 10------|
```

Supplementary file 9

b) PT-VENN and PT-LPEN modules in type-I and type-II proteins The size of the pretoxin PT-VENN module is defined (see PF04829. The upstream boundary of the PT-LPEN module is unknown. For sake of simplicity, the two pre-toxin modules have the same size in the alignment shown

```
bau-A1     KDATVEDISKYQEKVINTSKLISGSLASLAGYDAGYAASSASLA VSNN PT-VENN domain (type-I CdiA)
bau-B1     PANGIEYTDAEKLKIRNIGKLVSGTVAAYTGYDVNTAANSADTA IQNN
pit-B5     PANGIEYTDAEKLKIRNIGKLVSGTVAAYTGYDVNTAANSADTA IQNN
bau-C1     VAEYMKQYSSDQKLITNISNAMAVTIAAATGYDTAIAQQASETA VTNN
bau-C2     GRNPTFVSAEEKAKIEAYSKLIAGTISAYAGYDVNTAANSATIA IQNN
bau-C5     PANGIEYTEAEKSKILNITKLTTGVVAAYVGYDVTAAANAADTA FSNN
bau-C8     PINGIEYNDEEKVKIKNVGKVVAGVVSAYAGYDVNTAANSADIA IQNN

nos-4      AKDLTADQKSTISSIVGLAGSAVGATTGDISSTVQS-GQVAQNA VENN PT-VENN domain (type-II CdiA, class 1 proteins)
bau-5      AKDLTVDQKSTISSIVGLAGSAVGATTGDIGSIVQN-GQVAQNA VENN
bau-6      AKELTADQKSTISSIVGLAGSAVGATTGDIGSIVQS-GQVAQNA VENN
bau-7      AKDLTADQKSTISSIVGLAGSAVGATTGDIVSTVQS-GQVAQNA VEDN
nos-8      AKDLTADQKSTISSIVGLAGSAVGLTTGDIGSTVQS-GQVAQNA VENN
bau-9      AKELTADQKSTISSIVGLAGSAVGATTGDIGSIVQS-GQVAQNA VENN

bau-D10    PNLLPENVKSSIRDLTAAIGAVVGGTVGDSASNVQLAGVIGQNA VENN PT-VENN domain (type-II CdiA, class 2 proteins)
bau-D11    ANLLPENVKSGIRDLTAAIGAVVGGTVGDSASNAQLAGVIGQNA VENN
bau-D12    ANLLPENVKSGIRDLTAAIGAVVGGTVGDSASNAQLAGVIGQNA VENN
bau-D14    ANLLPENIKSGIRDLTAAIGAVVGGTVGDSSSNAQLAGVIGQNA VENN
bau-D13    ANLLPEDVKSSIRDLTSAIGVAVGGTVGDSAFSAQIAGVVGQNA VENN
bau-D20    ANLLPENVKSGIRDLTAAIGAVVGGTVGDSASNAQLAGVIGQNA VENN


nos-D22    NRDINTTQEITRDQTTGMLNGSVTVDHRLLSESGRAEIVQQQKD LPEN PT-LPEN domain (type-II CdiA, class 3 proteins)
bau-D17    NRDINTTQEITRDQTTGMLNGSVTVDHRLLSESGRAEIVQQQKD LPEN
bay-D15    NRDINNTQEITRDQTTGMLNGSVTVDHRLLSESGRAEIVKEQEQ LIEN
bau-D15    NRDINTTQEITRDQTTGMLNGSFTVDHRLLSESGRAEIVQQQKD LPEN
nos-D18    NRDINTTQEITRDQTTGMLNGSVTVDHRLLSESGRAEIVQQQKD LPEN
bau-D16    NRDINTTQEITRDQTTGMLNGSFTVDHRLLSESGRAEIVQQQKD LPEN
bau-D21    NRDVNNTQEITRDQTTGMLNGSVTVDHRLLSESGRAEIVQQQKD LPRN
bau-D19    NHDINTTQEITRDQTTGMLNGSFTVDHRLLSESGRAEIVQQQKD LPRN
bau-D2     NRDINNTQEITRDQATGMLNGSVTVDNRLLTESGRQQIIQEQKD LPQN
```

Supplementary file 9
c) potential N-domains in the CT region of type-I CdiA. Conserved residues are highlighted.

```
bau-B1  SLVRLATTTGKVLVKTLDKYNALRKTGK-QVTKDDIVASLKEQSADELIGIADDLLTVFGRSTSPFDRAMAAIDLVVGTDLKPGK
pit-B5  SLVRLATTTGKLLVKAAEIYGDIKKANKGIVDSKVFVQKLKDQGVSELVGIADDLVTVFGPASTPFDRALAAFDLIIGTDLKPGK
pit-A4  SLPKLATSGAKIAKKVLDQIKKWPSNKP--LEPNDVTKMFKDAGVQEIVDVANNIKTLVSPTSTWFEKISAAVDLAVGIDIKAGK
pit-B6  TLGKLATTAGKAAYRVAKRITDMPASVRANLKPDDVIKIFKEEGVQGVIDIGDNIKTLISPTSTPGDRAFAAIDLLIGIDLKAGK
pit-A5  -WGKVLSSTGKAAYKIFKKATELQKAGKNLNDTNVWKDILKDTATGEYQDMLNSFKTAFSAGSTPLERTWALIDLATGIDKKDVK
```

Supplementary file 9
d) upstream N-domains in the CT region of type-II CdiA (class-1 and class-2 proteins)
proteins with similar domains are marked

nos-D2
GENAPKAVADYAQKQALILRAQGNEAEAKKWDEGGIYRVAMHTALGALGTGTLEGAVTTGTVAASAPKINDFQNSVAQALTQTGISEKTAEAVASGVTSLTLAAAG
VVAGLNTGSTATAVNVDANNRQLHVDEAKVLKKLTEGKTEAEQTRLRAAACALVECSKGVPESDANYKSLKAFEALGKTYTKEQNALKATGLFTYSNWDKANDIRTSND
bau-D3
GENAPKAVADYAQKQALILRAQGNEAEAKKWDEGGIYRVAMHTALGALGTGTLEGAITTGTVAASAPKINDFQNSVAQALTHTGMSEKAAEAVASGVTSLTLAGVG
VASGLNTGSTATAVNVDANNRQLHVDEAKVLKKLTEGKTEVEQTRLRAAACALVHCSEGVPESDVNYKTLKAFEALGKTYIKEQNALKATGLFTYSNWDKANDIRTNHD

bau-D5
FLSVKEAARKNALIYKSKHEELSSNEKKELAEINRKDKARDEFIKNVCQLGNVSSSACQSALQAAWSTQADYNADIANNLKNRDVYSQDAKHLDQLLKGLSQDQVLG
LQAIERIAKTSGRPVEEVAKEYDRAMALHGVVSTLAGFYGGKAISVEKVKNGGGSFNALTEAEKQRALDNIANSKIGRESSNF
nos-D8
FLSVKEAARKNALIYKSKHEELSSNEKKELAEINRKDKARDEFIKNVCQLGNVSSSACQSALQAAWSTQADYNADIANNLKNRDVYSQDAKHLDQLLKGLSQDQVLG
LQAIERIAKTSGRPVEEVAKEYDRAMALHGVVSTLAGFYGGKAISVEKVKNGGGSFNALTEAEKQRALDNIANSKIGRESSNF
bau-D9
FLSVKEAARKNALIYKSKHEELSSNEKKELAEINRKDKARDEFIKNVCQLGNVSSSACQRALQAAWSTQADYNADIANNLKNRDVYSQDAKHLDQLLKGLSQDQVLG
LQAIERIAKTSGRPVEEVAKEYDRAMALHGVVSTLAGFYGGKAISVEKVKNGGGSFNALTEAEKQRVLDNIANSKAGRESSNF

bau-D14
EFSIITKGVEKKLAENKKEKEAQAHLICPKGQSCIIPIPEKSLGDKALNVINDMTLRQLAAVAGAKYDPLTGEEITPNERQLAKASMLGLGFTKSVSGVTRLTDEA
LVGIEKQYGKSIAEKI
bau-D20
EFSIITKGVEKKLADNKKEKEAQAHLSCPKGQSCMIPIPEKSLGDKALNVINNMTLRQLAAVAGAKYDPLTGEEITPNERQLAKASMLGLGLTRTVSGVTRLTDDA
LIGIEKQYGKSIAEKI

bau-D13
LFGVLTADKKIDKQKNNSYFQALQACKNAQCEKSVITQNALAAMQNVPKVLNVSSSKYKEGEIITNPNDVTGLRYLVVKDGGVMKAKLLPADYQVVFSVNQLNSSR
NLVQGGALASVIANTLKSGHDVFADRTLFTNQEITALDRAFAGLEVVGNTLLIGGSVLPKKVSIDAVAFKENNIYRD
cal-D23
LFGVLTADKKIDKQKNNSYFQALQACKNAQCEKSVITQNALAAMQNVPKVLNVSSSKYKEGEIITNPNDVTGLRYLVVKDGGVMKAKLLPADYQVVFSVNQLNSSR
NLVQGGALASVIANTLKSGHDVFADRTLFTNQEITALDRAFAGLEVVGNTLLIGGSVLPKKVSIDAVVVKENNFYRD

Supplementary file 9

e) upstream N-domains in the CT region of type-II CdiA (class-3 CdiA). Similar regions are highlighted. SWRs (switch regions) located immediately upstream of the C-domain of the CT region are shown. Only

```
nos-D22     NRDINTTQEITRDQTTGMLNGSFTVDHRLLSESGRAEIVQQQKDLPEN  FRQSAENLAKALPDGAYKDKALQTLNNVQA  KLYNLPSEYKEAGVVGNNVASELLKRGMESKDVEALFSKLDFFYAAKEFSEIKNQLTDLEKSGFDLETIFRQP
bau-D17     NRDINTTQEITRDQTTGMLNGSVTVDHRLLSESGRAEIVQQQKDLPEN  FRQSAENLAKALPDGVYKDKALQTLNNIQA  KLYNLPSEYKEVGVVGNSVASELLKRGMESQDVEALFSKLDFFYAAKEFSEIQNQLTDLEKSGVNLETIFKEP
bay-D15     NRDINNTQEITRDQTTGMLNGSVTVDHRLLSESGRAEIVKEQEQLIEN  FRQSAENLAKALPDGVYKDKALQTLNDVQA  KLYNLPSEYKEVGVVGNSVASELLKRGMESQDVEALFSKLDFFYAAKEFSEIQNQLTDLEKSGVNLETIFKQP
bau-21L     NRDINTTQEITRDQTTGMLNGSVTVDHRLLSESGRAEIVQQQKDLPEN  FRQSAENLAKALPDGAYKDKALQTLNNIQA  KLYNLPSEYKEVGVVGNTVASELLKRGMESQDVEALFSKLDFFYAAKEFSEIQNQLTDLEKSGFNLETIFRQP

bau-D15     NRDINTTQEITRDQTTGMLNGSFTVDHRLLSESGRAEIVQQQKDLPEN  FRQSAENVIKALPESEYKNQVLQTLNNVQA  KLYKMPTQYKDSGTLGSEVVLGLIKQGVEPQTINKLFDDKAAGMTTVLENLNDLNNKIDHLKQQGLSINDILG
nos-D18     NRDINTTQEITRDQTTGMLNGSVTVDHRLLSESGRAEIVQQQKDLPEN  LRQSAENVIKALPESEYKSQVLQTLNNVQA  KLYKMPTQYKDSGTLGSEVVLGLIKQGVEPQTINKLFDDKAAGMTTVLENLNDLNNKIDHLKQQGLSINDILG
bau-leaf    NRDVNSTQEITRDQTTGMLNGSVTVDHRLLSESGRAEIVQQQKDLPEN  FRQSAENVIKALPESEYKKQVLQTLNNVQA  KLYTMPTQYKDSGTLGSEVVLGLIKQGVEPQTINKLFDEKSAGMITVLENLNDLNSKIDNLKQQGLTINDILG

bau-D16     NRDINTTQEITRDQTTGMLNGSFTVDHRLLSESGRAEIVQQQKDLPEN  FRQSAENLVQALPDNAYKQRALKSLNNIQA  GLANNPEMLAKGGDEVYEGYKEFIRQGGEPEQYEALLNKEVLPLAKELNNTAKQTLDEIQKQIAEEYQVSDDT
bau-D21J    NRDVNNSQEITRDQTTGMLNGSVTVDHRLLSESGRAEIVQQQKDLPEN  FRQSAENLVQALPDNAYKQRALKSLNNIQA  GLANNPEMLAKGGDEVYEGYKEFIRQGGEPEQYEALLNKEVLPLAKELNNTAKQTLDEIQKQIAEEYQVSDDT

bau-D19     NHDINTTQEITRDQTTGMLNGSFTVDHRLLSESGRAEIVQQQKDLPRN  AEIVGKMTAAGVTSLGVATAALATKDQNLK  QAYDTVMNPARTFDFVQKHPEAATVLEQFKNGNYDGLLQTKGSIQLLAQALGQDVDVLTTSMTSFLGIKGAYD
bau-D2      NRDINNTQEITRDQATGLLNGSVTVDNRLLTESGRQQIIQEQKDLPQN  AEIIGKMTAAGVTSLGVATASLASGDQNLK  QAYDTVMNPARTFDFVQKHPEAATVLEQFKNGNYDRLLATKGSIQLLAQALGQDVNVLTTSITSFLNIKGAYD

            |_______________PT-LPEN _______________||_______________________________________ upstream N-domain ___________________________

nos-D22     KSGVNEEKTIYAQGTDFETKVS-SQTTLGMTILSNVADLGNSIKDISESTGVDIEKVQLAVGLLISGPVRLVVESGKSMAVDSLVGDKVAQ
bau-D17     KAGGNEQETIYAQGTDFETKVS-SQTTLGMTILSNVADLGNSIKDISESTGVDIEKVQLAVGLLISEPVRLVIESGKSMAVDSVVGEKVAQ
bay-D15     KAGVNEQETIYAQGTDFETKVR-SQTTLGMTILSNIADLGNSIKDISESTGVDIEKVQLAVGLLISGPVRLVIESGKSMAVDSVVGEKVAQ
bau-21L     KSGGNEQETIYAQGTDLETKVS-SQTTLGMTILSNVADLGNSIKDISESTGVDIEKVQLAVGLLISGPVRLVIESGKSMAVDSIVGEKVAQ

bau-D15     TDRNQSPTANDEITFKDYKVEIQNQSTIGMKLLQEINFIGNSIEEISKDTGVDLETVQLAVGLMISGPVRLVVGSAQSLGMDALAGPYKKK
nos-D18     TDRNQSPTANDEITFNDYKVEIQNQSTIGMKLLQEINFIGNSIEEISKDTGVDLETVQLAVGLMISGPVRLVVGSAQSLGMDALAGPYKKK
bau-leaf    ADKNQSSTVSDEITFKDYKVGIENKSTIGASLLQEINFIGNSIKEISKDTGVDLETVQLAVGLMVSGPVRLVVDSAKSLGVDTLVGPYKEK

bau-D16     ARSIVREVLTAVSQQDGANTNTTDETGTLNPNQPYKGTFELPTIVVTAKGDAVLIQGSQSDEASIAGENVVKVIDGSNLAIKLFDQSAAVSYKVNDAIEKTGIDPATAGLALSLAFGGPIGLARDLITDTLVGEPLAQ
bau-D21J    ARSIVREVLTAVSQQDGANTNTTDETGTLNPNQPYKGTFVLPTIVVTAKGDAVLIQGSQSDEASIAGENVVKVIDGSNLAIKLFDQSAAVSYKVNDAIEKTGIDPATAGLALSLAFGGPVGLARDLITDTLVGEPLAQ

bau-D19     HQTNTVVLDVNNENRSSILSTTGHEIAHGQGISNETSADLVGKSVDWAFSSGVKNNEATIDQYKDQLGDGKDASTQAQNVAVLEKDNTKALDAISDHADQIDEKTSYWQDIKH-DGCWSKECTVAYQKMDAAQEKAFRLGQAKAVTKFMNDIKNL
bau-D2      HQTDTVVLDVNNENRSSILNTTGHEIAHGQGIKNETSADLVGKSVDWAFNSGVKNNQVTIDQYKGQLGDGKDASTQAQNIAELVKDNTTIVDAISDHGDKIDEKTSVWQDIKY-LGCWNDECVAAYKQMDAAQEKAYRLGQAKATTKFVNDIKNL

A.seifertii                                                                           //*asnaqlagvigQNAVENN KTSIKQDIKHALTCWGLECHQQYKHLDVVQEAAYQKGKDRAVAKFVSDLKNL
A.junii                                                                  // aaigavvggtvgdsafeaqisgvvaqNAVENN KTSVKQDIKHALTCWGAECRQQYKDLKVAQKAAYQKGKDQAITKFVNDLNNL

            ____________________________________________________   upstream N-domain   ______________________________________________

nos-D22                                 GIDILANSMTAAAHDTSVNTINNWTNPDKISELENSTSENKEASLEFANEVQKD KQGAEYLINAAAGIVVGGIGGTAKTTKIDGKDVEVSTAGIG  SNSKKDPVKELEVGSYQELKSR //
bau-D17                                 GVDILANSMTAAAHDTSVNTISNWTNPDKISELETSTGENKEASLKFANEVQKD KQGAEYLINAAAGIVVGGIGGTAKTTKSDGKEAGVNSVGVG  GATWDTSKPVKTLDKVDNYYIN //
bay-D15                                 GVDILANSMTAAAHDTSVNTINNWTNPDKISELENSTGENKDASLKFANEVQKD KQGAEYLINAASGIIVGGIAKGTVKNNNSDIDISKSGFEIG  NINPITGHKILGTHKDESNNTV....
bau-D21L                                GVDILANSLTAAAHDTSVNTINNWTNPGKISELENSTGENKDASLKFANEVQKD KQGAEYLINAATGIVVGGIGGTAKTTKIDGKDVEVSQSGLE  IGWVDQYGVQKKVTGDGTVARD

nos-D18                                 GIDVLATAFTASAHSTS---TDVVEKMINPQYAKEVPEAERDNYESLSNELSSS KQGAEYLINAAAGIIVGGIGKDIAKKGENGNISSTNNINEE  ISALNRIKENDKGTDLSNKSSD //
bau-D15                                 GIDVLATAFTASAHSTS---TDVVEKMINPQYAKEVPEAERDNYESLSNELSSS KQGAEYLINAAAGIVVGGIGKGAVKNNNSDIDISKSGFEIG  NINPITGHKILGTHKDESNNTV....
bau-leaf                                GIDVLANAFTASAHLTS---TDVVEKLTNPQYESEVPEAERDTYESLSNELSNS KQGAEYLINAAAGIVVGGIGGTAKTTKIDGKDVEVSTAGIG  SRGELTKPYDPVQTRKDLEAVH....

bau-D16                                 GSDVLKNEVTAVVRDTSYDTVSGYTSNSTKQQLSGLTDPASQTALQVSEQLQRT KDGVGFLASVVLGA--GGIAKTA---KIDGNEVEVSTAGIG  SRGELTKPYDPVQTRNDLEVVH....
bau-D21J                                GSDVLKNEVTAVVRDTSYDTVSGYTSNSTKQQLSGLTDPASQTALQVSEQLKRT KDGVGFLASVVLGA--GGIAKTA---KIDGNEVEVSQSGLE  IGWVDQYGVQKKVTGDGTVARD....

bau-D19     VPNPKEVYDALTNDPMGAMAAIWDGVKNIPGELWQTGKTITNVNLVGNSPAEFEKLGNAEMTTVLN----AVSAGTITVAKKGGKI VVEAAKKVKLKADFDPSLNVQ--------------------  TNGFSLPPKTTLKVINIKADQA....
bau-D2      VPNPKEVYEALTKDPMGTMSAIWEGVKNIPGELWDTGKTITKGNLVGKSPAEFEKMGNAEMTTALNGLSAAISAGTVTVAKKGGGI VIEAAKSAKRILQNDPAKLPT--------------------  ITTSAGGYIKASLITENGKIID....

A.seifertii VPNPKEVYEAIKNDPAGTAKAIYEGLKQVPRDIIDTGKTIANVNTVGDTPAEFEKLGEADMALALNTLSGLVSAGTVTVAKKGGKI VIDASKTIKLKADFDPSLNVQ--------------------  TNGFSLPPKTAPKVINIKADQA....
A.junii     VPNPKELYDALKNDPQGTAKAIYEGVKQIPGDLIDTGKTIATVNTVGDTPAEFEKLGEADMALSLNALTGLISAGSVTVAKKGGKV VIEAAKKVKLKADFDQNLNVQ--------------------  VNGISGNRAVSDSNTSLIDEEK....
            ______________________________________________________________________ ||____________ SWR  _______________||_____C-domain_____
```

Supplementary file 10. Unrooted neighbor-joining analyses of the ST population of *A. pittii* and *A. nosocomialis.* CDI-positive ST are circled.

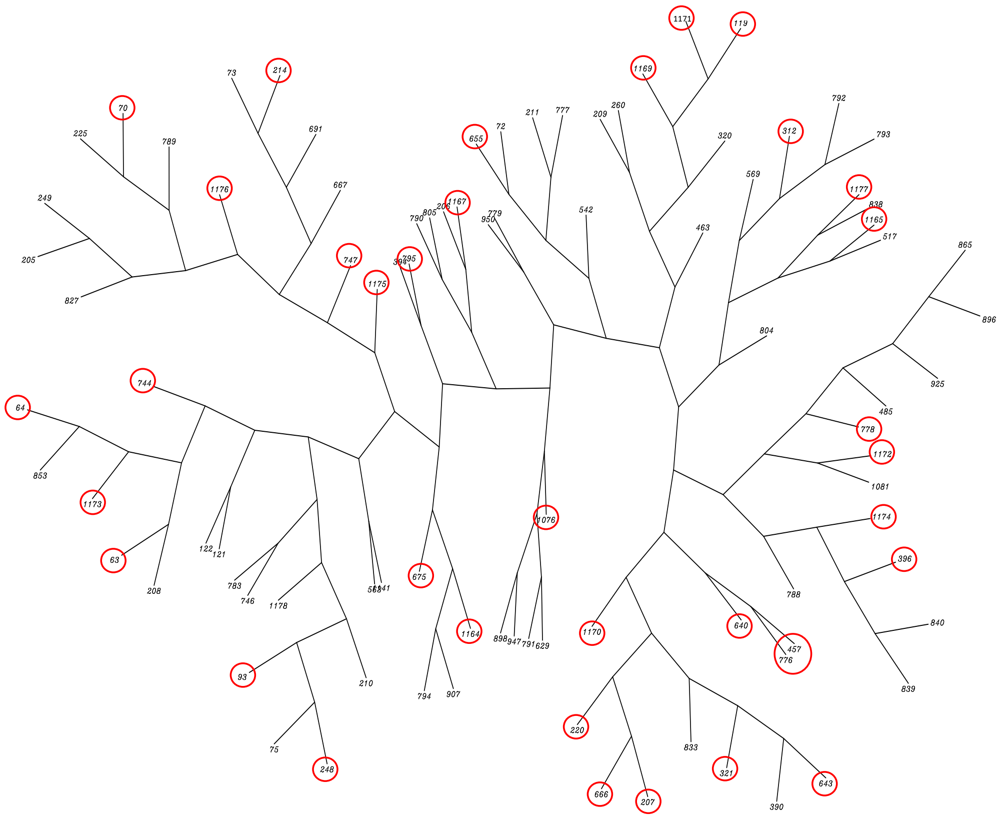

A. pittii

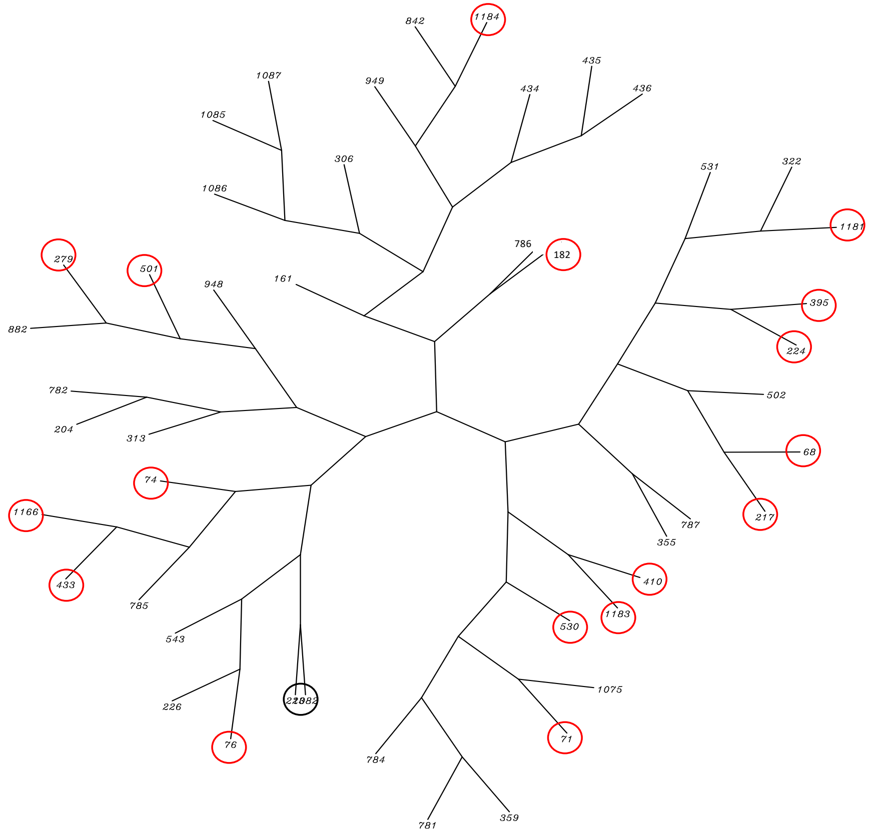

A. nosocomialis

Supplementary file 11. CdiA proteins in non ACB-complex Acinetobacter species
Identical proteins are highlighted

species	strain	GenBank ID	type	size (aa)	notes	modules	recognized CT domains
A. gerneri	MTCC 9824	ASYY01000035.1	II	2010	truncated	PT-LPEN	bacteriocin (pfam12639)
	DSM 14967	APPN01000054.1	II	>4000	truncated		
	KCTC 12415	BBLI01000056.1	II	2228	truncated		
A. guillouiae	CIP 63.46	APOS01000036.1					
	KCTC 23200	BBRY01000027.1	I	2113		DUF 637	
	MSP4-18	ASQG01000044.1					
	NIPH 991	APPJ01000002.1	II	1196	truncated		
A. junii	CIP 107470	APPS01000060.1					
	KCTC 12416	BBSD01000040.1	II	4136	*cdi*C gene between *cdi*B and *cdi*A genes	PT-VENN/SWR	endoU (pfam14436)
	MTCC 11364	ASYZ01000005.1					
A. haemolyticus	TJS01	NZ_CP018871.1	I	2246		DUF-637	HINT (cl25980)
	UBA4199	DFTZ01000108.1	I	2199		DUF-637	
A. bereziniae	XH901	NZ_CP018259.1					
	KCTC23199	BBLJ01000001.1	II	4155			
	LMG1003	APQG01000036.1					
	NIPH 3	APPK01000024.1	II	4662			ribonuclease (pfam00545)
A. soli	GFJ2	NZ_CP016896.1	II	3552			
	NIPH2899	APPV01000010.1	II	4024		PT-VENN	endoU (cl16881)
